# Safety and Immunogenicity of an Inactivated COVID-19 Vaccine, WIBP-CorV, in Healthy Children: Interim Analysis of a Randomized, Double-Blind, Controlled, Phase 1/2 Trial

**DOI:** 10.3389/fimmu.2022.898151

**Published:** 2022-06-24

**Authors:** Shengli Xia, Kai Duan, Yuntao Zhang, Xiaoqing Zeng, Dongyang Zhao, Huajun Zhang, Zhiqiang Xie, Xinguo Li, Cheng Peng, Wei Zhang, Yunkai Yang, Wei Chen, Xiaoxiao Gao, Wangyang You, Xuewei Wang, Zejun Wang, Zhengli Shi, Yanxia Wang, Xuqin Yang, Qingliang Li, Lili Huang, Qian Wang, Jia Lu, Yongli Yang, Jing Guo, Wei Zhou, Xin Wan, Cong Wu, Wenhui Wang, Shihe Huang, Jianhui Du, Xuanxuan Nian, Tao Deng, Zhiming Yuan, Shuo Shen, Wanshen Guo, Jia Liu, Xiaoming Yang

**Affiliations:** ^1^ Vaccine Clinical Research Center, Henan Center for Disease Control and Prevention, Zhengzhou, China; ^2^ National Engineering Technology Research Center for Combined Vaccines, Wuhan Institute of Biological Products Co Ltd, Wuhan, China; ^3^ Clinical Medical Center, China National Biotec Group Company Limited, Beijing, China; ^4^ Department of Infectious Diseases, Union Hospital, Tongji Medical College, Huazhong University of Science and Technology, Wuhan, China; ^5^ Chinese Academy of Sciences Key Laboratory of Special Pathogens, Wuhan Institute of Virology, Center for Biosafety Mega-Science, Chinese Academy of Sciences, Wuhan, China; ^6^ Department of Epidemiology and Biostatistics, College of Public Health, Zhengzhou University, Zhengzhou, China

**Keywords:** COVID-19, SARS-CoV-2, inactivated vaccine, clinical trial, children, safety, immunogenicity

## Abstract

Safe and effective vaccines against SARS-CoV-2 for children are urgently needed. Here we aimed to assess the safety and immunogenicity of an inactivated COVID-19 vaccine candidate, WIBP-CorV, in participants aged 3-17 years. A randomized, double-blind, placebo-controlled, phase 1/2 clinical trial was conducted in Henan Province, China, in healthy children aged 3-17 years. 240 participants in phase 1 trial and 576 participants in phase 2 trial were randomly assigned to vaccine or control with an age de-escalation in three cohorts (3-5, 6-12 and 13-17 years) and dose-escalation in three groups (2.5, 5.0 and 10.0μg/dose), and received 3 intramuscular injections at day 0, 28, and 56. WIBP-CorV showed a promising safety profile with approximately 17% adverse reactions within 30 days after injection and no grade 3 or worse adverse events. The most common adverse reaction was injection site pain, followed by fever, which were mild and self-limiting. The geometric mean titers of neutralizing antibody ranged from 102.2 to 1065.5 in vaccinated participants at 28 days after the third vaccination, and maintained at a range of 14.3 to 218.2 at day 180 after the third vaccination. WIBP-CorV elicited significantly higher titers of neutralizing antibody in the cohort aged 3-5 years than the other two cohorts. There were no detectable antibody responses in all alum-only groups. Taken together, our data demonstrate that WIBP-CorV is safe and well tolerated at all tested doses in participants aged 3-17 years, and elicited robust humoral responses against SARS-CoV-2 lasted for at least 6 months after the third vaccination. This study is ongoing and is registered with www.chictr.org.cn, ChiCTR2000031809.

## Introduction

As of early January 2022, the coronavirus disease 2019 (COVID-19) pandemic has caused over 289 million confirmed cases of severe acute respiratory syndrome coronavirus 2 (SARS-CoV-2) infection and over 5.4 million deaths globally (1). Children are susceptible to SARS-CoV-2 infection, and the number of COVID-19 children cases remains exceptionally high in some countries. As of December 2021, nearly 7.9 million children have tested positive for SARS-CoV-2 and children represented 17.4% of total cumulated COVID-19 cases in the United States of America (2). The incidence of COVID-19 among children raises the possibility of transmission among family members, and risk to elderly members who are more vulnerable to disease (3, 4). Moreover, although SARS-CoV-2 infection in children are mainly mild or asymptomatic, a number of children, especially those with underlying health comorbidities, might still be at risk for severe COVID-19 and serious complications, including multisystem inflammatory syndrome, myocardial dysfunction, shock, and respiratory failure requiring intensive care after the primary infection (5–8). Therefore, testing the effectiveness of COVID-19 vaccines in children is important for curbing the COVID-19 pandemic.

We have previously reported that an inactivated vaccine against COVID-19 WIBP-CorV (Wuhan Institute of Biological Products, Wuhan, China) is generally safe and has induced antibody responses in adults 18 years and older in a phase 1/2 trial (9). It has also been shown in a prespecified interim analysis of a double-blind, randomized, phase 3 trial that WIBP-CorV could significantly reduce the risk of symptomatic COVID-19 in adults (10). Thus, in this study, we further report the full safety set and immunogenicity data for WIBP-CorV in a phase 1/2 trial among healthy people younger than 18 years in China.

## Methods

### Study Design and Participants

This double-blind, randomized, placebo-controlled phase 1 and 2 trial were designed by the Wuhan Institute of Biological Products Co Ltd and Henan Provincial Center for Disease Control and Prevention (CDC). The study protocol, available in **Supplement 1**, was approved by the institutional review board of Henan Provincial CDC. Written informed consents were obtained from the parents of all participants. Participants aged 9–17 years also provided written assents before enrollment. The ongoing trial is being performed and data collected by the investigators at the CDC of Wuzhi Country, Henan Province, beginning on July 28, 2020. An independent data and safety monitoring board is monitoring the safety data and evaluating the risks among the participants during the trial.

Healthy children, aged 3 to 17 years, without history of SARS-CoV-2 infection (via on-site inquiry, serological and nucleic acid test) were eligible for enrollment in the study, and details of the inclusion and exclusion criteria are provided in **Supplement 2**. Participants were stratified by age (3-5 years, 6-12 years or 13-17 years) and were randomly assigned to receive intramuscular injections of 2.5μg, 5.0μg, 10.0μg dose, or alum control (1:1:1:1). Within each dose-escalating group of each age cohort in phase 1 and 2, the participants were sequentially assigned a computer-generated randomization number, and stratified block randomization (block size, 8) by subgroups was adopted. Within each randomization block, the ratio of vaccine vs placebo was 3:1. All vaccines used for inoculation were distributed in identical packages with serial numbers to ensure masking of participants. The safety evaluation and group allocation were masked from participants, investigators, and outcome assessors for the duration of the study. Four milliliters venous blood from elbow was collected before and 4 days after each vaccination from all participants.

### Vaccine

WBIBP-CorV was developed by Wuhan Institute of Biological Products (Wuhan, China), and manufactured as described previously (9). A SARS-CoV-2 strain (WIV04 strain, National Genomic Data Center of the Chinese Academy of Science accession No.SAMC133237, and GenBank accession number MN996528) was isolated from a patient in the Jinyintan Hospital, Wuhan. The virus was cultivated in a qualified Vero cell line for propagation, and the supernatant of the infected cells was inactivated with β-propiolactone (1:4000 vol/vol at 2 to 8°C for 48 hours. Following clarification of cell debris and ultrafiltration, the second β-propiolactone inactivation was performed in the same conditions as the first inactivation. The vaccine was adsorbed to 0.5-mg alum and packed into prefilled syringes in 0.5-mL sterile phosphate-buffered saline without preservative. The placebo group contained only sterile phosphate buffered saline and alum adjuvant. All the vaccines and placebos were approved by the National Institutes for Food and Drug Control of China.

### Outcomes

The primary outcome for safety was the occurrence of adverse reactions within 7 days after each vaccination. Adverse reactions within 30 days after whole vaccination procedure across all study groups were analyzed as secondary safety endpoints. The outcome for safety was the occurrence of all adverse reactions, including solicited and unsolicited adverse events, from the first dose to 30 days after the full course of vaccinations were collected by the investigator’s active visits and spontaneous reports. The participants were asked to record any injection site specific adverse reactions (eg, pain, redness, and swelling) and systemic adverse reactions (eg, fever, headache, and fatigue) on diary cards within 7 days of each injection, which were summed and considered as the primary safety outcome in both phases. Any other unsolicited symptoms were also recorded on the contact card during a 28-day follow-up period after each injection, and were considered as the secondary safety outcome. In the phase 1 trial, laboratory safety values (including routine blood tests, liver enzymes, total bilirubin, creatinine, urea nitrogen, urine protein, urine sugar, and urinary occult blood) were measured before and 4 days after each injection to assess any toxic effects after vaccination. The hematologic and biochemical markers were considered secondary safety measures in the phase 1 trial, and detailed methods for the laboratory measures are described in **Supplement 2**. The grading criteria of adverse reactions or events and the relationship with receiving injections were decided by the investigators before unblinding according to the standard guidelines issued by the National Medical Products Administration of China, and details are shown in **Supplement 2**.

The primary humoral immunogenicity outcomes included the geometric mean titers (GMTs) and seroconversion of neutralizing antibody and the specific IgG-binding antibody titers at 28 days after the second vaccination, and 28, 90 and 180 days after the third vaccination, respectively. The seroconversion rates of participants were defined as an increase of at least four-times post-vaccination titer from baseline. The neutralization capacity induced by vaccine against live SARS-CoV-2 (BetaCoV/Wuhan/AMMS01/2020 activated) was analyzed in triplicate by plaque reduction neutralization test (PRNT), and the PRNT50 values were reported as a measure to determine the extent to which serum can be diluted and still reduce SARS-CoV-2 plaque formation by 50%. The total specific IgG antibody responses were measured with an in-house–developed enzyme-linked immunosorbent assay kit, which used the inactivated whole SARS-CoV-2 as coating antigen. Details of the immunogenicity assays are provided in **Supplement 2**. The lower limit of detection was 5 for the neutralizing antibody test and 10 for the specific IgG antibody test, and those below the detection limit (eg, all baseline values) were assigned to 5 or 10, respectively, for further analysis. Seroconversion rate, as a secondary immunogenicity outcome, was defined as at least a 4-fold increase of antibody titers over baseline. Details of the immunogenicity assays are provided in **Supplement 2**.

### Statistical Analysis

The safety analysis was performed on data from all participants who received at least 1 dose. The number and proportion of participants with adverse reactions or events and the detailed safety profiles were compared across groups. The immunogenicity analysis was performed on data from the full analysis set of participants who received at least 1 dose and had results of any blood biomarker measurements before or after injections. The χ2 test or Fisher exact test (when data were sparse) was used to analyze categorical data, and the t test or the Mann-Whitney U test (for non-normally distributed data) was used to analyze log-transformed antibody titers between vaccine and alum-only groups. Differences across groups at different time points were analyzed by analysis of variance. Analyses were conducted using SPSS software, version 25.0 (IBM SPSS Inc). Hypothesis testing was 2-sided with p values of 0.05or less. The detailed statistical analysis plan is included in **Supplement 2**.

## Results

### Study Participants

**Figure 1:**
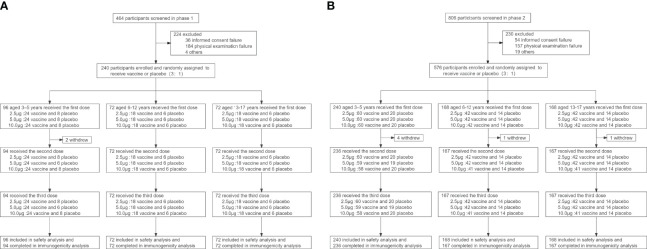
Trial profile for phase 1 **(A)** and phase 2 **(B)**.

Between July 28 and August 31, 2020, a total of 1270 participants were screened, and 240 were included in the phase 1 trial and 576 in the phase 2 trial (**Figure 1**). In the phase 1 trial, 96 aged 3-5 years, 72 aged 6-12 years and 72 aged 13-17 years were distributed over four cohorts (2.5μg, 5.0μg, 10.0μg and alum control cohorts). A total of 576 participants were recruited in phase 2 trial, including 240 aged 3–5 years, 168 aged 6–11 years, and 168 aged 12–17 years. All participants, but 2 in the phase 1 trial and 6 in the phase 2 trial, completed immunizations and scheduled visits within the prescribed time (**Figure 1**). Detailed demographic characteristics of the participants are listed in **Table 1**.

**Table 1:** Baseline characteristics of the study participants.

Table 1. Baseline Characteristics of the Study Participants in the Phase 1 Clinical Trial^a^															
Characteristic	3–5 years					6-12 years					13-17 years				
	Low dose	Medium dose	High dose	control	P	Low dose	Medium dose	High dose	control	P	Low dose	Medium dose	High dose	control	P
	(n=24)	(n=24)	(n=24)	(n=24)		(n=18)	(n=18)	(n=18)	(n=18)		(n=18)	(n=18)	(n=18)	(n=18)	
Age, years															
M(P25, P75)	4.5(4.0,5.4)	4.5 (3.8,5.4)	4.6(4.1,4.9)	4.5(3.9,5.5)	0.975	10.1(8.2,11.1)	9.6 (7.4,11.5)	10.1(9.0,11.2)	9.7(7.8,10.8)	0.912	14.6(13.8,15.9)	15.4(13.7,16.5)	14.6(13.3,15.8)	14.8(13.5,15.8)	0.551
X̄(SD)	4.6(0.8)	4.6 (0.8)	4.5(0.7)	4.6(0.9)		9.8 (1.7)	9.4 (2.1)	9.7(1.8)	9.5 (1.9)		14.9 (1.2)	15.4(1.4)	14.7(1.3)	15.0(1.5)	
Sex(%)															
Male	17 (70.8)	16 (66.7)	13 (54.2)	13 (54.2)	0.524	9 (50.0)	7 (38.9)	5 (27.8)	8 (44.4)	0.568	11(61.1)	11(61.1)	10(55.6)	13(72.2)	0.771
Female	7 (29.2)	8 (33.3)	11 (45.8)	11 (45.8)		9 (50.0)	11 (61.1)	13 (72.2)	10 (55.6)		7(38.9)	7(38.9)	8(44.4)	5(27.8)	
*^a^* *The low, medium, and high doses represent 2.5, 5.0, and 10.0μg/dose, respectively.*															
Table 1.2 Baseline Characteristics of the Study Participants in the Phase 2 Clinical Trial^a^															
**Characteristic**	**3–5 years**					**6-12 years**					**13-17 years**				
	**Low dose**	**Medium dose**	**High dose**	**control**	**P**	**Low dose**	**Medium dose**	**High dose**	**control**	**P**	**Low dose**	**Medium dose**	**High dose**	**control**	**P**
	**(n=60)**	**(n=60)**	**(n=60)**	**(n=60)**		**(n=42)**	**(n=42)**	**(n=42)**	**(n=42)**		**(n=42)**	**(n=42)**	**(n=42)**	**(n=42)**	
Age, years															
M(P25, P75)	4.7(3.8,5.4)	4.4 (3.8,4.9)	4.7(3.9,5.2)	4.7(3.7,5.4)	0.303	9.8(8.6,11.0)	10.0(8.5,11.6)	8.8(7.7,9.8)	8.8(7.3,10.7)	0.028	14.1(13.5,15.4)	15.1(14.1,16.3)	15.0(14.1,15.7)	14.7(13.7,15.8)	0.095
X̄(SD)	4.6 (0.9)	4.3(0.7)	4.6(0.8)	4.6(0.9)		9.8(1.7)	9.8(1.8)	9.0(1.6)	9.0 (1.8)		14.6 (1.3)	15.2 (1.2)	15.1(1.1)	14.8 (1.2)	
Sex(%)															
Male	26(43.3%)	25 (41.7%)	30(50.0%)	30(50.0%)	0.708	22(52.4%)	27 (64.3%)	24(57.1%)	22(52.4%)	0.654	26 (61.9%)	15 (35.7%)	21(50.0%)	29 (69.0%)	0.013
Female	34(56.7%)	35 (58.3%)	30(50.0%)	30(50.0%)		20(47.6%)	15(35.7%)	18(42.9%)	20(47.6%)		16 (38.1%)	27 (64.3%)	21(50.0%)	13 (31.0%)	

a The low, medium, and high doses represent 2.5, 5.0, and 10.0μg/dose, respectively.

### Safety Outcomes

The safety data of the phase 1 and phase 2 trial were combined for analysis because the same batches of the vaccine and aluminum hydroxide adjuvant and the same safety observation method were used. 139 (17.0%) of 816 participants reported at least one adverse reaction within 7 days of either vaccination. In an exploratory analysis by dosage, the number of participants reporting adverse reactions was 38 (18.6%), 32 (15.7%), 27 (13.2%), 42(20.6%) in 2.5μg, 5.0μg, 10.0μg and alum control cohorts within 7 days of either vaccination, respectively (**Table 2**, appendix 24 in **Supplement 3**). The most common adverse reaction was mild (grade 1) pain at the injection site, which was reported within 30 days after all three vaccinations by 10 (4.0%) of 252 participants in the vaccination groups, and 6 (7.1%) of 84 participants in the control group of the 3-5 years old cohort; 13 (7.2%) of 180 participants in the vaccination groups, and 7 (11.7%) of 60 participants in the control group of the 6-12 years old cohort; and 23 (12.8%) of 180 participants in the vaccination groups, and 12 (20.0%) of 60 participants in the control group of the 13-17 years old cohort (**Table 2**, appendix 24 in **Supplement 3**). A significant difference in the incidence of local adverse reactions among different dose vaccination groups and the control group was observed (p=0.021, **Table 2**). The second most common reaction was fever (grade 1 or 2), which was reported by 19 (7.5%) of 252 participants in all three dose groups, and by five (6.0%) of 84 participants in the control group of the 3-5 years old cohort; two (1.1%) of 180 participants in all three dose groups, and one (1·7%) of 60 participants in the control group of the 6-12 years old cohort; 12 (6.7%) of 180 participants in all three dose groups, and four (6·7%) of 60 participants in the control group of the 13-17 years old cohort. All adverse reactions were mild (grade 1 or 2), transient, and self-limiting, and did not require any treatment. No adverse reactions were reported between days 8 and 30 after injection. Unsolicited adverse events (regardless of relations with the immunization) are shown in **Supplement 2**. Two severe adverse events (grade 3) occurred in the phase 1 trial and eight severe adverse events (grade 3) occurred in the phase 2 trial during the follow-up but all were unrelated to the immunization.

**Table 2:** Adverse reactions after 3 doses in the phase 1/2 trial^a^.

Adverse reaction	Phase 1 clinical trial						Phase 2 clinical trial						Combination with phase 1 and phase 2 clinical trial				
	Low dose	Medium dose	High dose	control	P		Low dose	Medium dose	High dose	control	P		Low dose	Medium dose	High dose	control	P
	(n=60)	(n=60)	(n=60)	(n=60)			(n=144)	(n=144)	(n=144)	(n=144)			(n=204)	(n=204)	(n=204)	(n=204)	
**0-7d**																	
Total adverse reactions	19 (31.7)	14 (23.3)	10 (16.7)	18 (30)	0.216		19 (13.2)	18 (12.5)	17 (11.8)	24 (16.7)	0.632		38 (18.6)	32 (15.7)	27 (13.2)	42 (20.6)	0.209
Systemic adverse reactions	7 (11.7)	8 (13.3)	8 (13.3)	6 (10)	0.934		9 (6.3)	9 (6.3)	10 (6.9)	12 (8.3)	0.886		16 (7.8)	17 (8.3)	18 (8.8)	18 (8.8)	0.982
Hypersensitivity	0	0	0	1 (1.7)	>0.999		0	0	0	0	NA		0	0	0	1 (0.5)	>0.999
Fever	4 (6.7)	5 (8.3)	6 (10)	2 (3.3)	0.529		7 (4.9)	3 (2.1)	8 (5.6)	8 (5.6)	0.434		11 (5.4)	8 (3.9)	14 (6.9)	10 (4.9)	0.606
Diarrhea	1 (1.7)	0	0	0	>0.999		0	1 (0.7)	0	2 (1.4)	0.623		1 (0.5)	1 (0.5)	0	2 (1)	0.57
Joint pain	0	0	1 (1.7)	0	>0.999		0	0	0	0	NA		0	0	1 (0.5)	0	>0.999
Myalgia	0	0	0	0	NA		0	1 (0.7)	0	0	>0.999		0	1 (0.5)	0	0	>0.999
Coughing	2 (3.3)	4 (6.7)	2 (3.3)	2 (3.3)	0.741		2 (1.4)	4 (2.8)	1 (0.7)	3 (2.1)	0.565		4 (2)	8 (3.9)	3 (1.5)	5 (2.5)	0.412
Vomiting	0	0	1 (1.7)	0	>0.999		0	0	0	0	NA		0	0	1 (0.5)	0	>0.999
Loss of appetite	0	0	1 (1.7)	0	>0.999		0	0	1 (0.7)	0	>0.999		0	0	2 (1)	0	0.249
Headache	0	0	0	1 (1.7)	>0.999		0	0	1 (0.7)	0	>0.999		0	0	1 (0.5)	1 (0.5)	>0.999
Local adverse reactions	13 (21.7)	6 (10)	3 (5)	14 (23.3)	0.01		11 (7.6)	10 (6.9)	9 (6.3)	15 (10.4)	0.572		24 (11.8)	16 (7.8)	12 (5.9)	29 (14.2)	0.021
Erythema	1 (1.7)	2 (3.3)	0	3 (5)	0.331		1 (0.7)	3 (2.1)	0	2 (1.4)	0.338		2 (1)	5 (2.5)	0	5 (2.5)	0.107
Pain	12 (20)	6 (10)	3 (5)	12 (20)	0.036		9 (6.3)	8 (5.6)	8 (5.6)	13 (9)	0.59		21 (10.3)	14 (6.9)	11 (5.4)	25 (12.3)	0.056
Swelling	0	0	0	1 (1.7)	>0.999		1 (0.7)	1 (0.7)	1 (0.7)	1 (0.7)	>0.999		1 (0.5)	1 (0.5)	1 (0.5)	2 (1)	0.896
Other adverse reactions	2 (3.3)	0	0	0	0.247		1 (0.7)	0	0	0	>0.999		3 (1.5)	0	0	0	0.062
Abdominal pain	0	0	0	0	NA		1 (0.7)	0	0	0	>0.999		1 (0.5)	0	0	0	>0.999
Laryngeal pain	1 (1.7)	0	0	0	>0.999		0	0	0	0	NA		1 (0.5)	0	0	0	>0.999
Oral mucosal blister	1 (1.7)	0	0	0	>0.999		0	0	0	0	NA		1 (0.5)	0	0	0	>0.999
Runny nose	1 (1.7)	0	0	0	>0.999		0	0	0	0	NA		1 (0.5)	0	0	0	>0.999
Pharyngeal swelling	1 (1.7)	0	0	0	>0.999		0	0	0	0	NA		1 (0.5)	0	0	0	>0.999
**0-30d**																	
Total adverse reactions	19 (31.7)	14 (23.3)	10 (16.7)	18 (30)	0.216		19 (13.2)	18 (12.5)	17 (11.8)	24 (16.7)	0.632		38 (18.6)	32 (15.7)	27 (13.2)	42 (20.6)	0.209

NA, not applicable. ^a^The safety set included all participants who received at least 1 dose. The low, medium, and high doses represent 2.5, 5.0, and 10.0μg/dose, respectively. Data are shown as No. of participants with event (%).A participant was only counted once in the specific reaction category even though a participant could have more than 1 adverse reaction. For example, a participant who had the same symptom (eg, injection site pain) after each dose was counted once in the symptom category. Similarly, if a participant had more than 1 symptom in the reaction class (total, systemic, and local), they were only counted once in that adverse reaction class.

### Immunogenicity Outcomes

**Figure 2:**
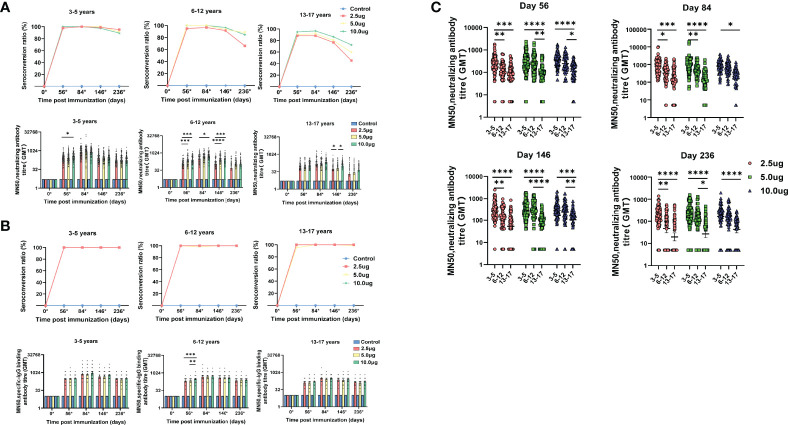
Antibody responses at different time points among different age cohorts in the phase 1/2 trial. **(A)** Seroconversion rates and geometric mean titers (GMTs) of neutralizing antibody to SARS-CoV-2 at different time points among different groups. **(B)** Seroconversion rates and GMTs of specific-IgG binding antibody to SARS-CoV-2 at different time points among different groups. **(C)** Titers of neutralizing antibody to SARS-CoV-2 were compared among different age cohorts at different time points after vaccination. Circles show the individual antibody titers and bars represent the geometric mean titers of antibodies. Error bars refer to the 95% CI. Seroconversion was defined as an increase of at least four-times post-vaccination titer from baseline. *, P < 0.05; **, P < 0.01; ***, P < 0.001; ****, P < 0.0001.

None of the participants had any detectable neutralizing antibody or specific-IgG binding antibody response against SARS-CoV-2 at baseline and remained negative throughout the studies in the control groups. In the cohort aged 3-5 years, seroconversion rates of neutralizing antibody reached 100% at day 84 (28 days after the third vaccination) in all three dose levels. In the cohort aged 6-12 years, 96.7% of participants in the 2.5μg group, and 100% of participants in the 5.0μg group and the 10.0μg group were neutralizing antibody seroconverted at day 84. In the cohort aged 13-17 years, 88.3% of participants in the 2.5μg group, 91.7% of participants in the 5.0μg group and 96.6% of participants in the 10.0μg group were neutralizing antibody seroconverted at day 84. In the cohort aged 3-5 years and the cohort aged 6-12 years, seroconversion rates of neutralizing antibody maintained at relatively high levels during the long-term follow-up. At day 236 (180 days after the third vaccination), the seroconversion rates of neutralizing antibody in the 2.5μg, 5.0μg and 10.0μg groups of the cohort aged 3-5 years were 95.2%, 91.5% and 89.3%, and in the cohort aged 6-12 years were 66.1%, 88.3% and 84.7%, respectively. In contrast, the seroconversion rates of neutralizing antibody in the cohort aged 13-17 years declined to 45%, 59.3% and 72.4% at day 236 (**Figure 2A**, **Table 3**). All participants but three in the 5.0μg group of the cohort aged 13-17 years were specific-IgG binding antibody seroconverted at day 56 (28 days after the second vaccination). All participants but one in the 5.0μg group of the cohort aged 6-12 years were specific-IgG binding antibody seroconverted at day 84. Seroconversion rates reached 100% at day 146, in all three dose levels of all three age cohorts, and were maintained at 100% at day 236, with the exception of one participant in the 5.0μg group of the cohort aged 13-17 years (**Figure 2B**, **Table 3**, appendix 77 in **Supplement 3**).

**Table 3:** Antibody responses in the phase 1/2 trial^a^.

Timepoints(days)	3-5 years					6-12 years					13-17 years					3-17 years				
	Low dose	Medium dose	High dose	control		Low dose	Medium dose	High dose	control		Low dose	Medium dose	High dose	control		Low dose	Medium dose	High dose	control
	(n=84)	(n=84)	(n=84)	(n=84)		(n=60)	(n=60)	(n=60)	(n=60)		(n=60)	(n=60)	(n=60)	(n=60)		(n=204)	(n=204)	(n=204)	(n=204)
**GMT of neutralizing antibodies to Live SARS-CoV-2, (95% CI)^b^**																				
**56**	237.8 (200.3~282.5)	268.4 (225.7~319)	383.4 (321.5~457.3)	5 (4.2~6)		108.7 (88.7~133.2)	223.6 (182.4~274)	248.6 (202.5~305.3)	5 (4.1~6.1)		71.8 (56.2~91.6)	78.7 (61.6~100.5)	112.7 (88.1~144.1)	5 (3.9~6.4)		132.8 (116.4~151.5)	176.9 (155~201.9)	234 (204.7~267.5)	5 (4.4~5.7)
**84**	794.3 (672.4~938.4)	901 (761.9~1065.5)	825.1 (694~980.9)	5 (4.2~5.9)		325.2 (264.8~399.3)	494.1 (401.7~607.8)	570.3 (463.7~701.6)	5 (4.1~6.1)		147.7 (110.7~197.2)	136.5 (102.2~182.2)	216.3 (161.6~289.5)	5 (3.7~6.7)		372.4 (320.9~432.3)	431.6 (371.6~501.3)	493.4 (423.8~574.4)	5 (4.3~5.8)
**146**	266.6 (223.6~317.9)	292.4 (245.0~349.1)	294.0 (244.6~353.3)	5 (4.2~6.0)		109.3 (83.3~143.5)	250.2 (191.1~327.7)	256.0 (195.1~336.0)	5 (3.8~6.5)		53.5 (39.3~72.9)	62.7 (46.1~85.4)	105.5 (77.3~144.0)	5 (3.7~6.8)		128.7 (106.3~155.7)	177.2 (147~213.6)	207.6 (175.3~246)	5 (5.0~5.0)
**236**	171.7 (135.1~218.2)	170.4 (133.7~217.3)	167.7 (130.1~216.2)	5 (3.9~6.4)		46.6 (33.3~65.3)	110.2 (78.9~153.9)	104.6 (74.7~146.5)	5 (3.6~7.0)		19.9 (14.3~27.9)	28.0 (20.0~39.3)	43.9 (31.3~61.8)	5 (3.6~7.0)		62.5 (49.3~79.1)	87.4 (70.1~109.0)	98.3 (79.6~121.4)	5 (5.0~5.0)
**Seroconversion rate of neutralizing antibodies to Live SARS-CoV-2, % (95% CI)^b^**																				
**56**	97.6 (91.7~99.7)	97.6 (91.6~99.7)	100	0		95.0 (86.1~99.0)	100	100	0		88.3 (77.4~95.2)	90.0 (79.5~96.2)	94.9 (85.9~98.9)	0		94.1 (90.0~96.9)	96.1 (92.4~98.3)	98.5 (95.6~99.7)	0
**84**	100	100	100	0		96.7 (88.5~99.6)	100	100	0		88.3 (77.4~95.2)	91.7 (81.6~97.2)	96.6 (88.3~99.6)	0		95.6 (91.8~98.0)	97.5 (94.3~99.2)	99.0 (96.4~99.9)	0
**146**	98.8 (93.5~100)	100	97.4 (90.9~99.7)	0		91.5 (81.3~97.2)	93.3 (83.8~98.2)	96.6 (88.3~99.6)	0		76.7 (64~86.6)	80 (67.7~89.2)	86.4 (75~94)	0		90.2 (86.1~94.3)	92.1 (88.4~95.9)	94.0 (90.7~97.3)	0
**236**	95.2 (88.3~98.7)	91.5 (83.2~96.5)	89.3 (80.1~95.3)	0		66.1 (52.6~77.9)	88.3 (77.4~95.2)	84.7 (73.0~92.8)	0		45.0 (32.1~58.4)	59.3 (45.7~71.9)	72.4 (59.1~83.3)	0		72.1 (65.8~78.3)	72.1 (65.8~78.3)	83.2 (78.0~88.5)	0
**GMT of specific IgG binding responses to SARS-CoV-2, (95% CI)^b^**																				
**56**	282.7 (248.9~321.2)	289.5 (254.6~329.1)	331.3 (290.7~377.5)	10 (8.8~11.4)		211.1 (183.4~243.1)	226.3 (196.5~260.5)	301.7 (261.8~347.8)	10 (8.7~11.5)		128.5 (110.6~149.2)	133 (114.5~154.4)	160 (137.6~186)	10 (8.6~11.6)		205.7 (188.4~224.7)	213.9 (195.8~233.7)	259.4 (237.2~283.7)	10 (9.1~10.9)
**84**	754.8 (632.2~901.3)	731.5 (612~874.4)	1015.9 (845.2~1221.2)	10 (8.3~12)		463.1 (389.8~550.3)	488.5 (410.5~581.2)	512 (430.2~609.2)	10 (8.4~11.9)		298.6 (257.3~346.5)	259.9 (224~301.6)	298.2 (256.7~346.5)	10 (8.6~11.6)		497.7 (445.1~556.5)	478.1 (427.4~534.8)	571.5 (510~640.5)	10 (8.9~11.2)
**146**	452.5 (391.0~523.7)	462.1 (398.9~535.2)	622.9 (534.8~725.6)	10 (8.7~11.5)		395.4 (340.1~459.5)	417.4 (359.5~484.5)	400.0 (344.2~465.0)	10 (8.6~11.6)		239.7 (207.8~276.6)	213.6 (185.1~246.4)	253.0 (219.0~292.2)	10 (8.6~11.7)		359.2 (322.6~400.0)	356.9 (320.1~398.1)	412.1 (369.9~459.1)	10 (10~10)
**236**	285.1 (250.1~325)	268.0 (234.7~306)	335.1 (291.7~385)	10 (8.7~11.5)		224.9 (194.0~260.9)	266.0 (229.6~308.1)	250.0 (215.6~290)	10 (8.6~11.6)		139.3 (121.3~159.9)	129.5 (112.7~148.8)	160.0 (139.0~184.1)	10 (8.7~11.5)		215.0 (194.6~237.7)	213.9 (191.8~238.4)	244.9 (222.0~270.1)	10 (10~10)
**Seroconversion rate of specific IgG binding responses to SARS-CoV-2, % (95% CI)^b^**																				
**56**	100	100	100	0		100	100	100	0		100	95.0 (86.1~99.0)	100	0		100	98.5 (95.7~99.7)	100	0
**84**	100	100	100	0		100	98.3 (90.9~100)	100	0		100	100	100	0		100	99.5 (97.3~100.0)	100	0
**146**	100	100	100	0		100	100	100	0		100	100	100	0		100	100	100	0
**236**	100	100	100	0		100	100	100	0		100	98.3 (90.9~100)	100	0		100	99.0 (97.6~100.4)	100	0

SARS-CoV-2, severe acute respiratory syndrome coronavirus 2.^a^Immunogenicity population is defined as randomized participants who received at least 1 dose injection with nonmissing immunogenicity data before or after injections. ^b^95% CI=95% confidence interval. The low, medium, and high doses represent 2.5, 5.0, and 10μg/dose, respectively. Seroconversion was defined by at least a 4-fold increase in postinjection titer from baseline. The baseline values were imputed by the lower limit of detection of the assays, which was 5 for the neutralizing antibody measurement and 10 for the specific-IgG binding antibody measurement.

The GMTs of neutralizing antibody and specific-IgG binding antibody elicited by WIBP-CorV at day 56, 84, 146 and 236 are listed in **Table 3**. The GMTs of neutralizing antibody detected at day 84 were approximately 2- to 3-fold higher than those at day 56 across all three dose levels of all three age cohorts, suggesting a third dose of vaccination could strongly boost the established neutralizing antibody response. During the long-term follow-up, the titers of neutralizing antibody started to decline in all three dose levels of all three age cohorts. The decline of GMTs of neutralizing antibody between day 84 and 146 was approximately 2- to 3-fold, and between day 84 and 236 was approximately 5- to 7-fold (**Figure 2A**, **Table 3**). Similar dynamics was also observed for specific-IgG binding antibody in all three dose levels of all three age cohorts (**Figure 2B**, **Table 3**).

Dose-level dependent immunogenicity was observed in all three age cohorts at different time points after the second or third vaccination. In the cohort aged 3-5 years, the neutralizing antibody GMTs of the 10.0μg group were significantly higher than that of the 2.5μg group at day 56 (p=0.0399). In the cohort aged 6-12 years, the GMTs of the 5.0μg group were significantly higher than that of the 2.5μg group at day 56 (p=0.0003), and day 146 (p<0.0001). The GMTs of the 10.0μg group were significantly higher than that of the 2.5μg group at day 56 (p=0.0002), day 86 (p=0.0203) and day 146 (p=0.0006). In the cohort aged 13-17 years, the GMTs of the 10.0μg group were significantly higher than that of the 2.5μg group (p=0.028) and the 5.0μg group (p=0.0278) at day 146 (**Figures 2A, B**).

Interestingly, different immunogenicity of WIBP-CorV was also observed among the three age cohorts. At all analyzed time points, the 3-5 years old cohort showed significant higher neutralizing antibody GMTs than the 13-17 years old cohort in all three dose groups (day 56: 2.5μg, p=0.0001; 5.0μg, p<0.0001; 10.0μg, p<0.0001; day 84: 2.5μg, p=0.0004; 5.0μg, p<0.0001; 10.0μg, p=0.0123; day 146: 2.5μg, p<0.0001; 5.0μg, p<0.0001; 10.0μg, p=0.0001; day 236: 2.5μg, p<0.0001; 5.0μg, p<0.0001; 10.0μg, p<0.0001). The 3-5 years old cohort also showed significant higher GMTs than the 6-12 years old cohort in the 2.5μg group at all analyzed time points (day 56: p=0.0042; day 84: p=0.014; day 146: p=0.0051; day 236: p=0.0051), and in the 5.0μg group at day 84 (p=0.0012). The 6-12 years old cohort showed significant higher GMTs than the 13-17 years old cohort in the 5.0μg group at day 56 (p=0.0017), day 146 (p<0.0001) and day 236 (p=0.0161), and in the 10.0μg group at day 56 (p=0.0113) and day 146 (p=0.0063) (**Figure 2C**).

Previous studies have demonstrated that SARS-CoV-2 vaccines could induce more robust SARS-CoV-2-specific humoral responses in females than males in adults (11) (12, 13). Therefore, we next analyzed whether gender has an impact on WIBP-CorV-induced antibody responses in children. In the cohort aged 3-5 years, the neutralizing antibody GMTs of the 2.5μg group were significantly higher in males than females at day 146 (p=0.038) and day 236 (p=0.029). However, this difference in neutralizing antibody titers between males and females was not observed in any other dose groups or in any other age cohorts (Appendix 79 in **Supplement 3**).

## Discussion

In this interim report of the phase 1/2 clinical trial, we investigated the safety and immunogenicity of the WIBP-CorV inactivated vaccine in participants aged 3-17 years. Our data showed that the three-dose regimen of WIBP-CorV had an acceptable safety profile, and was able to elicit robust humoral response against SARS-CoV-2 in children. Local and systemic adverse reactions were mostly mild to moderate in severity. Fever and injection-site pain were the most reported systemic and local adverse reactions and were transient. None of the serious adverse events reported during the trial was related to vaccination. These results were similar to our observation in adults (9). The incidence of systemic adverse reactions in different dose groups was similar, but significant differences in the incidences of local and other adverse events were observed. The higher injection-site pain reported was the main reason for the higher incidence of local adverse reactions in the 2.5μg group compared with the other two dose groups.

Several clinical trials of vaccine candidates on different platforms (inactivated (14, 15), mRNA (16, 17), and vector based (18)) have been performed to investigate their safety and immunogenicity against SARS-CoV-2 in children. Two mRNA COVID-19 vaccines, BNT162b2 (tozinameran; Pfizer-BioNTech) and mRNA-1273 (Moderna, Cambridge, MA, USA), had only been characterized in participants aged 5-15 years and 12-17 years, respectively. Both mRNA vaccines were reported to be tolerated and highly effective against COVID-19 after the second dose in participants (16) (17). The safety and immunogenicity of two inactivated vaccines, CoronaVac (Sinovac, Beijing, China) and BBIBP-CorV (Beijing Institute of Biological Products, Beijing, China), are being tested in phase 1/2 clinical trials in recipients including younger populations under 12 years. These two inactivated vaccines also demonstrated favorable safety profiles and elicited robust humoral responses against SARS-CoV-2 infection in children (14, 15). A two-dose vaccination regimen was utilized in the phase 2 trial of CoronaVac in participants aged 3-17 years, and the GMTs of neutralizing antibody elicited by CoronaVac 28 days after the second vaccination ranged from 78.3 to 146 (14). In comparison, the GMTs of neutralizing antibody elicited by WIBP-CorV 28 days after the second vaccination ranged from 118.3 to 266.7, which were about 1.5-fold higher than those elicited by CoronaVac. The vaccination regimen of BBIBP-CorV in phase 1/2 trial was identical to the current study (15). By 28 days after the third vaccination, the GMTs of neutralizing antibody elicited by BBIBP-CorV ranged from 143.5 to 224.4 in the cohort aged 3-5 years, 127 to 184.8 in the cohort aged 6-12 years, and 150.7 to 199 in the cohort aged 13-17 years. In comparison, the GMTs of neutralizing antibody elicited by WIBP-CorV ranged from 672.4 to 1065.5 in the cohort aged 3-5 years, 264.8 to 701.6 in the cohort aged 6-12 years, and 102.2 to 289.5 in the cohort aged 13-17 years, which were also significantly higher than those elicited by BBIBP-CorV, especially in the cohort aged 3-5 years and 6-12 years. Moreover, the GMTs of neutralizing antibody elicited by WIBP-CorV with the three-dose regimen in children were also significantly higher than those in adult participants aged 18-59 years, which ranged from 123 to 457 as we previously noted in the phase 1 trial (9). Thus, our current study documented a significant age-related difference in immunogenicity of WIBP-CorV, and suggests that WIBP-CorV may possess an advantage in eliciting SARS-CoV-2-specific humoral responses in young children. Age is well known as an important factor that influences vaccine responses. Actually, it has been documented that some other SARS-CoV-2 vaccines, including Corona Vac, BNT162b2 and an adenovirus-vectored COVID-19 vaccine, induced higher antibody titers in children and adolescents than in adults and the elderly (14, 16, 18, 19). Moreover, the association of age with SARS-CoV-2 antibody responses was also observed in nature SARS-CoV-2 infection. SARS-CoV-2 IgG and total antibody levels, neutralizing activity, and avidity was found negatively correlated with age in patients aged 1 to 24 years (4). Another community-based study of household clusters of mild COVID-19 found that children aged <6 years, and, in particular, those aged <3 years, developed higher long-lasting levels of NAbs compared with older siblings and/or adults (20). Consistently, a very recent study reported that young children aged 0–4 years generally develop significantly higher titers of neutralizing antibody than adults. This study also documented a stepwise downward progression in binding-to-neutralizing (B/N) antibody titer ratio with age: children aged 0–4 years had the highest ratio of B/N antibody, children aged 5–17 years had an intermediate level, and adults had the lowest (21). So far, the exact mechanisms underlying the different SARS-CoV-2 antibody responses based on age remain unclear. A few possibilities have been suggested. First, it has been proposed that children have strong innate immune response due to trained immunity by other vaccines, in particular by live-attenuated vaccines such as measles, mumps, and rubella (22), which may thus confer a better immune environment for developing anti-SARS-CoV-2 humoral immune responses. Second, it has been shown that only one-third of the dose of BNT162b2 vaccine given to adults is needed for children aged 5–11 years to develop comparable SARS-CoV-2 antibody responses to adults (23), suggesting the effect of inducing higher antibody levels in children with younger age by same dose of COVID-19 vaccines could be similar to a dose dependent effect of vaccination. Third, the phenomenon could be due to the onset of cross-reactivity among the different beta coronaviruses. This cross-immunity given that multiple putative epitopes for B and T cells, which are conserved among SARS-CoV-2 and the human coronavirus 0C43 and HKU1 (4, 24). Future studies are needed to further characterize the potential mechanisms.

In this study, the antibody responses elicited by WIBP-CorV were characterized up to 180 days after full vaccination. To our knowledge, this is the first report of long-term immunogenicity of COVID-19 candidate vaccine among children as low as 3 years old. Our data demonstrate that the titers of neutralizing antibody to SARS-CoV-2 peak at one month after three doses of vaccination and start to decline within the next two months to less than 50% of the peak titers, and further decline to about 20% of the peak titers 6 months after full vaccination. Our result suggests that there may be a need for booster vaccinations in children long-term post inoculating COVID-19 inactivated vaccines.

There are several limitations of our study. Firstly, cellular immunity elicited by WIBP-CorV, which was also important for controlling SARS-CoV-2 infection (25), was not evaluated. Secondly, participants in this study had limited racial and ethnic diversity as compared with the general population. Lastly, whether the neutralizing antibody elicited by WIBP-CorV could inhibit currently dominant SARS-CoV-2 variant Omicron was not characterized. Actually, the cross-neutralizing immunity against Omicron variant induced by inactivated COVID-19 vaccines were intensively characterized recently (26–31). These studies consistently demonstrated sharp reductions in virus neutralizing activity in the serum of individuals who received only two-dose inactivated COVID-19 vaccines. However, a third dose of homologous or heterologous booster vaccination could significantly increase the positive neutralization activity against Omicron, albeit the titers of neutralizing antibody to Omicron were still significantly lower than those to SARS-CoV-2 prototype (28–30). These data suggest the necessity of a third dose boost vaccination in children to elicit neutralizing immunity that helps reduce the Omicron variant escape and improve the protection.

Taken together, we found that the inactivated COVID-19 vaccine WIBP-CorV is tolerable and immunogenic in individuals aged 3-17 years. Our results support the evaluation of this vaccine candidate in phase 3 trials with populations aged 3-17 years to further ascertain its safety and protection efficacy against SARS-CoV-2. Our current trial, along with other clinical trials of different COVID-19 vaccines, will further address the feasibility of safe and efficacious vaccines in preventing SARS-CoV-2 in children.

## Data Availability Statement

The original contributions presented in the study are included in the article/**Supplementary Material**. Further inquiries can be directed to the corresponding authors.

## Ethics Statement

The studies involving human participants were reviewed and approved by Henan Provincial Center for Disease Control and Prevention. Written informed consent to participate in this study was provided by the participants’ legal guardian/next of kin.

## Author Contributions

XMY and SX had full access to all of the data in the study and take responsibility for the integrity of the data and the accuracy of the data analysis. SX, KD and YZ, and XZ contributed equally and are joint first authors. SS and ZY and WG are joint last authors. Concept and design: SX, SS, WG, KD, ZY, YZ, XL, YY, WC, CW, XMY. Acquisition, analysis, or interpretation of data: SX, DZ, HZ, XZ, ZX, CP, WZha, XG, WY, XWW, ZW, ZS, YW, XQY, LH, QW, JLu, YLY, JG, WZhou, XW, WW, SH, JD, QL, XZ, XN, TD, JLi. Drafting of the manuscript: SX, XZ, SH, JD, XN, TD, JLi. Critical revision of the manuscript for important intellectual content: SX, SS, WG, KD, ZY, YZ, DZ, HZ, ZX, XL, CP, WZha, YKY, WC, XG, WY, XWW, ZW, ZS, YW, XQY, QL, LH, QW, JLu, YLY, JG, WZhou, XW, CW, WW, XMY. Statistical analysis: XZ, ZW, YLY, JLi. Obtained funding: SX, SS, WG, KD, ZY, YZ, XWW, ZW, JLi, XMY. Administrative, technical, or material support: SX, HZ, YKY, WC, ZW. Supervision: SS, WG, KD, ZY, YZ, XMY. All authors contributed to the article and approved the submitted version.

## Funding

This study was supported by the National Program on Key Research Project of China (2020YFC0842100), Major Science and Technology Project of the National New Drug Development of China (2018ZX09734-004) and the National Natural Science Foundation of China (92169105, 82172256, 81861138044, and 91742114).

## Conflict of Interest

SX, WG, and KD, YZ, XWW, ZW, ZY, SS, and XMY reported receiving grants from the Ministry of Science and Technology of the People’s Republic of China during the conduct of the study. YZ, YKY, XWW, XQY, QW, and XMY reported being employees of the China National Biotec Group Co Ltd. KD, XL, ZW, JLu, JG, WZhou, XW, CW, WW, SH and SS; WC and QL reported being employees of the Wuhan Institute of Biological Products Co Ltd. JD, XN, TD reported being a student of the Wuhan Institute of Biological Products Co Ltd. YLY reported receiving personal fees from Wuhan Institute of Biological Products Co Ltd during the conduct of the study.

The remaining authors declare that the research was conducted in the absence of any commercial or financial relationships that could be construed as a potential conflict of interest.

The China National Biotec Group Co Ltd and the Wuhan Institute of Biological Products Co Ltd were the study sponsors and designed the trial, provided the study product, and oversaw all trial operations. The sponsors used contract clinical research organizations to coordinate interactions with regulatory authorities and oversee clinical site operations. Data were collected by the clinical site research staff, managed by a blinded contract research organization data management team, monitored by a contract research organization, and overseen by the sponsor and an independent data and safety monitoring board. The interim analysis was performed by an independent statistician who was not involved in the trial after the data were collected, checked, and locked for the specific groups before unblinding. Manuscript preparation was performed by the study authors and the decision to submit the manuscript for publication was made by the study authors.

## Publisher’s Note

All claims expressed in this article are solely those of the authors and do not necessarily represent those of their affiliated organizations, or those of the publisher, the editors and the reviewers. Any product that may be evaluated in this article, or claim that may be made by its manufacturer, is not guaranteed or endorsed by the publisher.
