# Supplementary material for: Safety and Immunogenicity of an Inactivated COVID-19 Vaccine, WIBP-CorV, in Healthy Children: Interim Analysis of a Randomized, Double-Blind, Controlled, Phase 1/2 Trial
Source: Front Immunol. 2022 Jun 24;13:898151. doi: 10.3389/fimmu.2022.898151 (PMC9265248; doi:10.3389/fimmu.2022.898151)
Supplement: Supplementary file 1 [file DataSheet_1.pdf]

Supplemental Online Content

Supplement to: Guo W, Duan K, Zhang Y, et al. Safety and immunogenicity of an inactivated COVID-19 vaccine, WIBP-CorV, in healthy children: interim analysis of 2 randomized clinical trials up to 180 days after full vaccination.

Supplement 1. Trial protocol

This supplemental material has been provided by the authors to give readers additional information about their work.

Section 1: **Protocol**

PROTOCOL COVER PAGE

Brief title: Phase 1/2 clinical trials for an inactivated vaccine (Vero cell) for the COVID-19

Protocol title: Evaluation of the safety and immunogenicity of an inactivated vaccine (Vero cell) for the COVID-19 in in healthy children aged 3-17 years: randomized, double-blind, placebo-controlled phase 1/2 clinical trials

Product name: An inactivated vaccine (Vero cell) for the COVID-19

Specifications: Low dose: 100 WU/dose;

Medium dose: 200 WU/dose;

High dose: 400 WU/dose

Protocol No.: WIBP2020001SQ

Version: Version 3.1(final)

Date: June 14, 2020

Sponsor: Wuhan Institute of Biological Products Co., Ltd;

Investigator: Henan Provincial Center for Disease Control and Prevention;

Researcher: Wuzhi county Center for Disease Control and Prevention, Jiaozuo, Henan

Statistical Party: Teaching and Research Unit of Biostatistics, School of Public Health, Zhengzhou University, Zhengzhou, Henan Province, China

Document history

Version No.	Version Date		Amendment
1.0	March 19, 2020		N/A
2.0	April 9, 2020		1st Amendment
2.1	April 28, 2020		2nd Amendment
3.0	May 20, 2020		3rd Amendment
3.1	June 14, 2020		4thAmendment
**Information of the 1st Amendment**			
Contents in Original Version (1.0)		Contents in Altered Version (2.0)	
Chapter	Original Contents	Page/Row	Altered Contents
Chapter 5	Lack of preclinical study in version 1.0	Chapter 5	Addition of some results from preclinical studies
Protocol summary, and Chapter 7	In the phase 2 trial, the sample sizes among the three-dose groups were 30 in the low-, medium-, and high-dose groups, 10 in the corresponding placebo groups (E1-E6 in the Table 4 in the Protocol Summary and Table 9 in the Chapter 7)	Protocol summary (Table 4), and Chapter 7 (Table 9)	In the phase 2 trial, the sample sizes in the three-dose groups have been changed to 60 in the low-, medium-, and high-dose groups, 20 in the corresponding placebo groups (E1-E6 in the Table 4 and Table 9)
**Information of the2nd Amendment**			
**Information of the 3rd Amendment**			
Contents in Original Version (2.1)		Contents in Altered Version (3.0)	
Chapter	Original Contents	Page/Row	Altered Contents
Chapter 5	None	Chapter 5 (5.2.3.2 Monkeys)	Adding the results of long-term safety experiment among monkeys
**Information of the 4th Amendment**			
Contents in Original Version (3.0)		Contents in Altered Version (3.1)	
Chapter	Original Contents	Page/Row	Altered Contents
Chapter 7	Considering the urgent COVID-19 epidemic, partial unblinding could be conducted in certain dose groups. Blinding codes from different subgroups are enclosed separately, which could facilitate the local unbinding. For example, when we get the results of immunogenicity test on day	Chapter 7 (7.10.5 Unblinding and reporting)	Considering the urgent COVID-19 epidemic, partial unblinding could be conducted in certain dose groups. Blinding codes from different subgroups are enclosed separately, which could facilitate the local unbinding. For example, when we get the results of humoral and cellular immunogenicity tests on day 14 after the second inoculation in the phase 1 and 2 trials, partial

	28 after the whole-course immunization and safety evaluation after one month from the end of the whole-course immunization in the low-dose group, partial unblinding in the low-dose group can be carried out after inspection and data cleaning.		unblinding in those groups can be carried out after inspection and data cleaning.

Sponsor information

Protocol title	Evaluation of the safety and immunogenicity of an inactivated vaccine (Vero cell) for the COVID-19 in healthy children aged 3-17 years: randomized, double-blind, placebo-controlled phase 1/2 clinical trials
Protocol No.	WIBP2020001SQ
Date	June 14, 2020
Version	Version 3.1 (final)
Sponsor	Wuhan Institute of Biological Products Co., Ltd
Project leader of the sponsor	Name: Kai Duan<br>Institution: Wuhan Institute of Biological Products Co., Ltd;<br>Address: No.1, Golden Industrial Park Road, Zhengdian, Jiangxia District, Wuhan<br>Postcode: 430207<br>Telephone number: 18995586168<br>Fax: 027-86637111<br>E-mail：duankai@sinopharm.com

Statement of the principal investigator

Protocol No.	WIBP2020001SQ
Date	June 14, 2020
Version	Version 3.1

I agree:

- To undertake the responsibility of correctly guiding the clinical research in this area.
- To ensure that this study is conducted in accordance with the trial protocol and the standard operating procedures of clinical research.
- To ensure that the personnel involved in the project fully understand the research product information and other research-related responsibilities and obligations specified in the protocol.
- To ensure that the protocol will not be changed without the review and written approval of the sponsor and the Ethics Committee (IEC), unless there are immediate hazards to the participants needed to be eliminated or requirements of the registration authority (e.g. administrative aspects of the project).
- I am fully familiar with the correct use of the vaccine described in the trial protocol and fully understand other information provided by the sponsor, including but not limited to the current investigator's Manual (IB) or equivalent documents and relevant supplements
- I am familiar with and will comply with the *Good Clinical Practice* (GCP), *the guideline for vaccine clinical trial quality management (Trial),* and all current regulatory requirements.

Name of the principal investigator: Shengli Xia

Signature of the principal investigator: ______________ Date： ______________

Content

Document history 3

Sponsor information 5

Statement of the principal investigator 6

Content 7

List of abbreviations 12

Glossary of terms 14

Research team 17

Protocol Summary 20

1. Introduction 30

2. Responsibilities of related parties in the clinical trial 30

2.1 Sponsor 30

2.2 Investigator 31

2.3 Researchers and clinical trial site 32

2.4 Laboratory measurement units 33

2.5 Contract Research Organization 34

2.6 Data management unit 36

2.7 Statistical party 37

2.8 Data and safety monitoring board (DSMB) 37

3. Background and principle 37

3.1 Disease background 38

3.2 pathogen background 39

3.3 Vaccine background......40
4. Product information......45
4.1 Characteristics of the experimental vaccine......45
4.2 Production process......45
4.3 Vaccination route and immunization procedure......47
4.4 Preservation and transportation of vaccines......47
5. Preclinical study......47
5.1 Stability study......47
5.2 Safety study......48
5.3 Immunogenicity......54
5.4 Animal protection effect......57
5.5 Summary of preclinical research......58
6. Research objectives......59
6.1 Main purpose......59
6.2 Secondary purpose......59
7. Study design......60
7.1 Procedures and methods......60
7.2 Control group......63
7.3 Investigational vaccine......64
7.4 Endpoints......65
7.5 Safety assessment......67
7.6 Evaluation of immunogenicity......77

7.7 Study hypothesis 78

7.8 Selection of the best immune procedure and dosage 78

7.9 Sample size 78

7.10 Randomization and blinding 80

7.11 Trial suspension or early termination criteria 86

7.12 Violation and deviation of protocol 87

7.13 Study duration 88

8. Participants 88

8.1 Inclusion criteria 88

8.2 Exclusion criteria for the first dose 88

8.3 Exclusion criteria for second and third doses 90

8.4 Early withdrawal of the participants 91

9. Procedures and methods of the trial 91

9.1 Recruitment 91

9.2 Informed consent 91

9.3 Inclusion/exclusion criteria of physical examination and examination 92

9.4 Study number allocation 92

9.5 Blood sample collection before immunization (phase 2 clinical trial) 92

9.6 Vaccine inoculation 93

9.7 Clinical observation after inoculation in the field 94

9.8 Safety observation and follow-up 94

9.9 The second and third vaccinations 96

9.10 Sample collection 96

9.11 Concomitant drug use 97

9.12 Closure of the field 98

9.13 Criteria for ending the clinical trial 98

10. Data management and statistical analysis plan 99

10.1 Data management 99

10.2 Contents of statistical analysis 100

11. Monitoring of clinical trials 107

11.1 Sponsor 107

11.2 Investigators and researchers 108

11.3 DSMB 109

11.4 Personnel training 110

11.5 Inspectors 110

11.6 Safety of the participants 110

11.7 Measures for improving compliance 117

11.8 Blood sample management and numbering rules 118

11.9 Vaccine management 119

11.10 Calibration and standardization of instruments and equipment 121

11.11 Quality control management of clinical trial data 121

11.12 Relevant documents 123

12. Ethics committee 126

12.1 Review and approval 126

12.2 Inspection in the research field 126

13. References 128

14. Accessory: informed consent 131

14.1 Informed consent for the phase 1 trial 131

14.2 Informed consent for the phase 2 trial 143

List of abbreviations

ACE2	angiotensin converting enzyme 2
ADE	antibody dependence enhancement
AE	adverse event
CI	confidence interval
CDC	Centers for Disease Control and Prevention
CoV	coronavirus
COVIV	coronavirus inactivated vaccine
DSMB	Data Safety and Monitoring Board
eCRF	electronic Case Report Form
EDC	Electronic Data Capture System
FAS	full analysis set
GCP	Good Clinical Practice
GMP	Good Manufacturing Practice
GMT	geometric mean titer
GMI	geometric mean increase
IB	investigator's handbook
ICF	informed consent form
IEC	independent ethics committee
MERS	Middle East respiratory syndrome

NMPA	National Medical Products Administration
PPS	per-protocol set
SAE	serious adverse events
PBMC	peripheral blood mononuclear cell
SARS	severe acute respiratory syndrome
SOP	standard operating procedure
SS	safety set
SUSAR	suspicious and unexpected severe adverse reactions
VDE	vaccine enhanced disease
WHO	World Health Organization

Glossary of terms

Trial protocol: the document describing the background, theoretical basis, purpose, design, method, and implementation of the trial, including statistical analyses, execution of the trial, and termination requirements.

Randomization: the process of randomly assigning treatment to participants to reduce selection bias.

Ethics Committee: an independent organization composed of medical professionals, legal experts, and non-medical personnel, whose responsibilities are to verify whether the clinical trial protocol and supplementary materials are ethical, and to provide public guarantee and ensure the safety, health, and rights of the participants are protected. The composition and all activities of the committee should not be interfered or influenced by the clinical trial sponsors and investigators.

Investigator's Manual: the clinical and non-clinical research materials for the investigators to implement the clinical trial.

Investigational drug: a drug with active ingredients that is going to be evaluated in clinical trials or placebo used as a control drug, including those approved for marketing but for indications that were not previously approved, or for collecting more information about the approved content.

Investigator(s): researcher(s) in charge of the implementation and quality of clinical trials, as well as the safety and rights of participants. The researchers must pass the qualification examination and have the specialty, qualification, and ability needed in clinical trials.

Sponsor: the company, institution, or organization that initiates a clinical trial and is responsible for its initiation, management, finance, and supervision.

Supervisor: the person with relevant knowledge who is appointed by the sponsor and responsible for the sponsor, whose task is to monitor and report the process of the trial and collate the data.

Contract research organization: an individual or organization who signed a contract with the sponsor and conducted certain tasks and work for the sponsor in the clinical trial.

Participant: the individual who receives investigational drugs or placebo in the clinical trial, and the researchers have contacted the participants or their parents or guardian about their participation in the clinical research.

Participant ID: a unique identification code that can be linked to the participant's documents in the clinical trials. When adverse events and other data related to the trial are reported, the privacy information of the participant is kept confidential, and the ID can be used to replace the name of the participant; the participant can use his/her original medical record number as the ID to facilitate tracing all clinical data.

Informed consent: the process that the participants voluntarily confirm their participation in the clinical trial after being informed of all aspects of the trial, which must be evidenced by the signed and dated informed consent.

Written informed consent: it is a documentary proof that each participant has expressed the willingness to participate in a certain experiment. The researcher should explain the nature and purpose of the trial, the possible benefits and risks, alternative treatment methods, and the rights and obligations of the participants in line with the declaration of Helsinki, so that the participants can fully understand and express their consent.

Case report form: a document designed according to the trial protocol to record the data of each participant during the trial.

Standard operation procedure (SOP): the detailed and standard procedure of certain work to complete the work uniformly.

Supervision: to supervise and review the progress and process of clinical trials, and to ensure that clinical trials are implemented, recorded, and reported following the trial protocol, SOP, good clinical practice (GCP),

and relevant laws and regulations related to drug clinical trials.

Inspection: a systematic inspection conducted by personnel not directly involved in the trial to evaluate whether the implementation of the trial and data recording and analysis comply with the requirements of the trial protocol, SOP, and relevant regulations related to drug clinical trials.

Adverse event: the unexpected medical events in participants during the clinical trial, which may not necessarily have a causal relationship with the investigational drug.

Adverse reactions: unexpected or harmful reactions occurred during the process of vaccination according to the prescribed dose and procedure, which are usually related to vaccination.

Serious adverse events: the events occurred during the clinical trial, including hospitalization, prolonged hospitalization, disability, work limitation, congenital malformation, threat to life or death, et al.

Research team

Sponsor

Company name: Wuhan Institute of Biological Products Co., Ltd;

Address: 1 Golden Industrial Park Road, Zhengdian, Jiangxia District, Wuhan;

Contact person: Wei Chen.

Investigator (institution in charge of clinical trials)

Organization name: Henan Provincial Center for Disease Control and Prevention;

Address: Nongye East Road, Zhengdong New District, Zhengzhou, Henan Province

Contact person: Shengli Xia.

Principal Investigator

Name: Shengli Xia;

Department: Vaccine clinical research center; Henan Provincial Center for Disease Control and Prevention.

Researchers and clinical trial site

Organization name: Wuzhi County Center for Disease Control and Prevention, Jiaozuo, Henan Province;

Address: 63 Wenhua Road, Wuzhi County, Jiaozuo City, Henan Province;

Person in charge: Xueli Li.

Clinical trial inspection unit

Company name: Beijing Zhongsheng Hengyi Pharmaceutical Technology Co., Ltd;

Address: Room 127, Floor 1, Building 71, 30 Litang Road, Dongxiaokou Town, Changping District, Beijing;

Contact person: Qi Guo.

Data management unit

Company name: Beijing Zhongsheng Hengyi Pharmaceutical Technology Co., Ltd;

Address: Room 127, Floor 1, Building 71, 30 Litang Road, Dongxiaokou Town, Changping District, Beijing;

Contact person: Mengru Zhao.

Clinical trial laboratory testing unit

Institution 1: National Institutes for Food and Drug Control, China;

Address: 31 Huatuo Road, Daxing District Biomedical Industry Park, Beijing;

Contact person: Changgui Li.

Institution 2: Wuhan Institute of Viruses, Chinese Academy of Sciences;

Address: No. 44, middle Xiaohongshan District, Wuchang District, Wuhan, Hubei Province;

Contact person: Zhiming Yuan.

Institution 3: People's Hospital of Wuzhi County;

Address: 519 Chaoyang 2nd Road, Longquan Street, Wuzhi County, Jiaozuo City, Henan Province;

Contact person: Chun Wang.

Institution 4: Zhengzhou Jinyu Clinical Test Center Co., Ltd

Address: Zhengzhou Pilot Free Trade Zone, Henan Province;

Contact person: Xu Zhao.

Institution 5: Shenzhen Huada Life Science Research Institute

Address: 6/F, National Gene Library, Jinsha Road, Dapeng New District, Shenzhen;

Contact person: Longqi Liu.

Statistical party

Organization name: Teaching and Research Office of Biostatistics, School of Public Health, Zhengzhou University, Zhengzhou, Henan Province;

Address: 100 Science Avenue, High Tech Development Zone, Zhengzhou City, Henan Province

Contact person: Yongli Yang.

Protocol Summary

**Research title**	Evaluation of the safety and immunogenicity of an inactivated vaccine (Vero cell) for the COVID-19 in healthy children aged 3-17 years: randomized, double-blind, placebo-controlled phase 1/2 clinical trials
**Product characteristics**	The inactivated vaccine (Vero cell) is produced by inoculating COVID-19 WIV04 strain in Vero cells, which is then cultured, harvested, inactivated, clarified, concentrated, secondary inactivated, purified, and finally made with the adjuvant of aluminum hydroxide.
**Study population**	Healthy individuals aged 3-17 years old will be recruited.
**Research objective**	To evaluate the safety and immunogenicity of different doses and administration timing of an inactivated vaccine (Vero cell) for COVID-19 in healthy children aged 3-17 years.
**Investigational vaccine**	**Investigational vaccine 1:**<br>Low-dose inactivated vaccine (Vero cells) for COVID-19;<br>Production unit: Wuhan Institute of Biological Products Co., Ltd; Wuhan Institute of Virology, Chinese Academy of Sciences;<br>Specification: 100 WU/dose per time for human use, 0.5 ml/dose;<br>Route of inoculation: intramuscular injection;<br>Storage conditions: 2-8 °C;<br>Vaccine batch No.: 202003001; expiry date: 2023/03/12;<br><br>**Investigational vaccine 2:**<br>Medium-dose inactivated vaccine (Vero cells) for COVID-19;<br>Production unit: Wuhan Institute of Biological Products Co., Ltd; Wuhan Institute of Virology, Chinese Academy of Sciences;<br>Specification: 200 WU/dose per time for human use, 0.5 ml/dose;<br>Route of inoculation: intramuscular injection;<br>Storage conditions: 2-8 °C;<br>Vaccine batch No.: 202003002; expiry date: 2023/03/12;

	**Investigational vaccine 3:**<br>High-dose inactivated vaccine (Vero cells) for COVID-19;<br>Production unit: Wuhan Institute of Biological Products Co., Ltd; Wuhan Institute of Virology, Chinese Academy of Sciences;<br>Specification: 400 WU/dose per time for human use, 0.5 ml/dose;<br>Route of inoculation: intramuscular injection;<br>Storage conditions: 2-8 °C;<br>Vaccine batch No.: 202003001; expiry date: 2023/03/12;
**Placebo control**	Name: aluminum hydroxide adjuvant<br>Active ingredient: none; virus content: none;<br>Adjuvant: aluminum hydroxide<br>Auxiliary materials: sodium chloride, disodium hydrogen phosphate, potassium chloride, potassium dihydrogen phosphate;<br>Manufacturer: Wuhan Institute of Biological Products Co., Ltd;<br>Specification: 0.5 ml/tube, 0.5 ml/time for human use;<br>Route of inoculation: intramuscular injection;<br>Storage conditions: 2-8 °C;<br>Vaccine batch No.: 202003001; expiry date: 2023/03/12;
**Research design**	The phase 1/2 clinical trials are 2 randomized, double-blind and placebo-controlled trials among healthy children aged 3-17 years. The participants are divided into three groups, i.e. low-dose, medium-dose, and high-dose groups. In the phase 1 trial, 3 doses of vaccine or placebo are administered to participants on day 0, day 28, and day 56, and the number of participants in each dose group is three times as many as the number of participants in the placebo group (3:1 ratio). In the phase 2 trial, three doses of vaccine (in low, medium, and high doses, respectively) or placebo are administered to participants on day 0, day 28, and day 56 with increased sample sizes but same 3:1 ratio.<br>The data safety and monitoring board (DSMB) is set up to assess the risk of clinical trial, so as to ensure the trial could be conducted safely according to the protocol and standards.<br>The vaccines are administered in the low-dose group among those aged 6 ~ 17 years old group

first, followed by the medium-dose and high-dose group. (The 6-17-year-old group is divided into two parts, and the sample size is 50% of the sample size of the dose group. The first part is the 13-17-year-old group in the low-dose group, followed by the medium-dose and high-dose group. Meanwhile, after the safety assessment on the 8th day after the first dose among adolescents aged 13-17 years, the vaccines are administered to those aged 6-12 years in the low-dose group first, followed by the medium-dose and high-dose group.) The later 3-5-year-old group will enter the group from low dose to high dose. In the phase 1 trial, laboratory tests are conducted before each inoculation and on the 4th day after the inoculation. After the safety assessment on the 8th day post the first low dose in the phase 1 trial, the same dose in the older children in the phase 1 trial and same dose in the younger children in the phase 2 trial, as well as the next higher dose group in the younger children in phase 1 trial could be inoculated if the preliminary assessment of safety meets the criteria of the protocol. Given the urgency, the phase 2 trial can be timely carried out according to the real situation at the clinical site.

The chronological order of the first-dose inoculation in different dose groups is shown in Table 1.

Table 1. The chronological order of the first-dose inoculation in different dose groups in phase 1 and 2 clinical trials

Age group	13-17			6-12			3-5		
Dose group	Low	Medium	High	Low	Medium	High	Low	Medium	High
Day 0	◆I								
Day 8	◆II	◆I		◆I					
Day 16		◆II	◆I	◆II	◆I		◆I		
Day 24			◆II		◆II	◆I	◆II	◆I	
Day 32						◆II		◆II	◆I
Day 40									◆II

Table 2. Sample size and procedure of phase 1 clinical trial

Age group	Group	Dose	Timing of inoculation	N	Safety assessment	Immunogenic blood collection

6-17	A1	L	Days 0, 28, 56	36	1. Blood and urine samples were collected before and 4 (+ 3 days) after each dose of immunization for blood biochemistry, blood routine and urine routine tests; 2. Collect AE and SAE within 0-7 days and 8-28 / 30 days after each dose of immunization; 3. Collect SAE within 12 months after the whole process exemption.	The first dose: (1) before; (2) the 4th day after; (3) the 14th day after; The second dose: (4) before; (5) the 4th day after; (6) the 14th day after; The third dose: (7) before; (8) the 4th day after; (9) the 28th day after; (10) the 90th day after; (11) the 180th day after; (12) the 360th day after.
	A2	P		12		
	A3	M		36		
	A4	P		12		
	A5	H		36		
	A6	P		12		
3-5	E1	L	Days 0, 28, 56	24		
	E2	P		8		
	E3	M		24		
	E4	P		8		
	E5	H		24		
	E6	P		8		

Note: (1) the blood collection window period is +3 days on the 4th day after each dose of inoculation, +7 days on the 14th and 21st day after the first dose of inoculation, +7 days on the 14th day after the 2nd and 3rd dose of inoculation, +10 days on the 28th day after inoculation, and +20 days on the 90th, 180th and 360th day after the whole dose of inoculation;

(2) Before and on the 4th day after each dose of inoculation, in addition to antibody test, blood routine test and biochemical test are still needed;

(3) The participants use the diary card to record the adverse events 0-7 days after the inoculation and the contact card to record the adverse events 8-28/30 days after the inoculation;

(4) Vaccine inoculation window period +7 days.

(5) L: low dose; M: medium dose; H: high dose

In the phase 1 trial, all participants are tested for humoral immune antibody titer according to the schedule in the Table 2, and parameters related to cellular immunity safety outcomes (Table 3) are tested according to the schedule in the Table 2.

Table 3. Parameters related to cellular immunity safety

Lymphocyte count	Cytokine
(1) Detection of lymphocyte subsets absolute count and relative ratio (T/B/NK cells);	IL-1β、 IL-2、 IL-4、 IL-5、 IL-6、IL-8、 IL-10、

(2) Activated monocyte;<br>(3) NK cell subpopulation (mature NK cell, immature NK fine);<br>(4) Regulatory T cell subsets	IL-12p70、IL-17A、IL-17F、IL-21、IFN -γ、TFN -α、TFN -β;

The sample size and procedures of the phase 2 trial are shown in the Table 4.

Table 4. Sample size and procedure of the phase 2 trial

Groups	Dose	Procedure	N	Safety assessment	Immunogenic blood collection
A1	L	Day 0, 28, and 56	84	1. Collect AE and SAE within 0-7 days and 8-14 / 21 / 28 / 30 days after each dose of immunization;<br>2. Collect SAE within 12 months after the whole process exemption.	① before the first dose; ② before 2 doses; 3 doses ③ before immunization and ④ on the 28th day after immunization; The 90th day ⑤ 180th day ⑥ 360th day ⑦ after the whole process free
A2	P		28		
A3	M		84		
A4	P		28		
A5	H		84		
A6	P		28		
E1	L	Day 0, 28, and 56	60		
E2	P		20		
E3	M		60		
E4	P		20		
E5	H		60		
E6	P		20		

Note: (1) the window period of blood collection on the 28th day after inoculation is +10 days, and the window period of blood collection on the 90th, 180th and 360th day after inoculation is +20 days;

(2) The participants use the diary card to record the adverse events between day 0 and day 7 after the inoculation, and use the contact card to record the adverse events on days 8-14/21/28/30 after the inoculation;

	(3) Vaccine inoculation window period is +7 days.
**Inclusion Criteria**	— Age range: healthy children aged 3-17 years<br>— General good health as established by medical history and physical examination;<br>— Since December 2019, the participant has not gone to Hubei province, overseas, or to a village/community where there had COVID-19 cases, have not contacted with confirmed or suspected cases, are not in the quarantine period, and are not from a village/community where there were confirmed or suspected cases.<br>— Women who have reached menarche are not pregnant (negative urine pregnancy test), are not breastfeeding, do not have pregnancy plan within the three months after enrollment, and have taken effective contraceptive measures two weeks before enrollment;<br>— Participants are able and willing to complete the whole research procedure in about 14 months;<br>— Participants have the ability to understand the research procedures, to sign the informed consent voluntarily after explanation, and can comply with the requirements of the clinical research program.
**Exclusion criteria for the first dose**	— Confirmed, suspected, or asymptomatic COVID-19 cases;<br>— Those with positive antibody tests of the COVID-19;<br>— History of SARS virus infection (identified through self-report or on-site inquiry)<br>— Those with fever (axillary temperature >37.0 °C), dry cough, fatigue, nasal obstruction, runny nose, sore throat, myalgia, diarrhea, shortness of breath, and dyspnea within 14 days before inoculation;<br>— Those with clinically abnormal parameters from blood biochemical, blood routine, and urine routine before inoculation (only for phase 1 clinical trial);<br>— Axillary temperature >37.0 °C before inoculation;<br>— Those who have experienced severe allergic reactions (such as acute anaphylaxis, urticaria, eczema, dyspnea, neurovascular edema, or abdominal pain), or those who are allergic to the known gradients of COVID-19 inactivated vaccine;

	— Those with history or family history of convulsion, epilepsy, encephalopathy, or mental illness;<br>— Those with congenital malformation, developmental disorder, genetic defect, or severe malnutrition, etc;<br>— Those with severe hepatorenal diseases, uncontrolled hypertension (systolic blood pressure ≥140 mmHg, diastolic blood pressure ≥90 mmHg), diabetes complications, malignant tumors, or various acute or chronic diseases (acute attack stage);<br>— Those diagnosed with congenital or acquired immunodeficiency, HIV infection, lymphoma, leukemia, or other autoimmune diseases;<br>— Those with confirmed or suspected serious respiratory diseases, serious cardiovascular diseases, hepatorenal diseases, and malignant tumors;<br>— Those with a history of abnormal coagulation (such as lack of coagulation factors or coagulation diseases);<br>— Those receiving anti-TB treatment;<br>— Those receiving immunoenhancement or inhibitor treatment (p.o. or gtt.) within 3 months (continuous oral or infusion for more than 14 days);<br>— Those receiving live attenuated vaccines within one month before inoculation or other vaccines within 14 days before inoculation;<br>— Those receiving blood products within 3 months before inoculation;<br>— Those receiving other study drugs within 6 months before inoculation;<br>— Those under other conditions not suitable for the clinical trial (evaluated by researchers).
**Exclusion criteria for the second and third doses**	— Women who have reached menarche with positive urine pregnancy tests;<br>— Those with high fever (axillary temperature ≥39.0 °C) lasting for three days or severe allergic reaction after the previous inoculation;<br>— Serious adverse reactions related to the previous inoculation;<br>— If inconformity with inclusion criteria or conformity with exclusion criteria for the first dose occurs or is newly found after the previous inoculation, researchers should decide whether the participants could continue to participate in the study;<br>— Other reasons for exclusion evaluated by researchers.

	Participants are not required to stop the trial if<br>— non-specific immunoglobulins are used during the study;<br>— steroid hormones have been given orally or intravenously for 14 days.
**Criteria for early withdrawal of participants**	Early withdrawal refers to the process that the participants fail to complete the vaccination and blood collection procedures according to the protocol, and the researchers decide whether to continue the subsequent procedures. Early withdrawal is needed if any of the following issues occur.<br>— The participant or the guardian of the participant requests to withdraw from the clinical trial;<br>— There are intolerable adverse events, no matter if they are related to the experimental vaccine or placebo;<br>— It is inappropriate for the participant to continue the trial due to his/her health condition;<br>— Participants are vaccinated with other experimental vaccines during the study period;<br>— Any other reasons decided by researchers.
**Trial suspension or early termination criteria**	If there are any of the following situations, the trial should be suspended, and the investigator, the sponsor, DSMB and the ethics committee should jointly hold a meeting to decide whether to terminate the clinical trial in advance:<br>■ Any adverse reaction of level 4 or SUSAR causally related to vaccination in any group;<br>■ In any subgroup, the number of participants with adverse reactions of level 3 exceeds 15% of the total number of participants in that subgroup after each dose;<br>■ The DSMB evaluates the clinical trial and believes there is a potentially substantial safety risk.<br>The clinical trial should be terminated in advance if there are any of the following situations.<br>■ The sponsor find that the vaccine has potential safety hazards or the research has quality problems and requires the trial to be terminated completely;<br>■ The ethics committee call for the termination of the trial because it is unethical;<br>■ The government authorities request the termination of the trial.
**Endpoints**	**Phase 1:**<br>**Safety endpoints:**

	● The primary safety endpoint is the incidence of adverse reactions within 7 days after each injection;<br>The secondary safety endpoints include:<br>● Incidence of any adverse reactions/events within 30 minutes after each injection;<br>● Incidence of abnormal hepatorenal function and abnormal parameters of blood and urine routine before and on the 4th day after each injection;<br>● Lymphocyte subsets and cytokine measures before each injection, on day 14 after the first and second injection, before the third injection, and on days 28, 180, and 360 after the third injection;<br>● Incidence of adverse events from day 8 to 28/30, and from days 0 to 28/30 after each injection;<br>● Incidence of serious adverse events (SAE) within 12 months.<br>**Humoral immunogenicity endpoints:**<br>● Specific ELISA antibody titers to whole SARS-CoV-2 virus, and the neutralizing antibody amounts against live SARS-CoV-2 virus. A positive antibody response (seroconversion) is defined as at least a four-fold increase in post-injection titer from baseline.<br>● Seroconversion and antibody titers (GMT, GMI) before each injection, on the 4th and 14th days (except for the third injection among those aged 3-17 years) after each vaccination, and on the 28th, 90th, 180th, and 360th days after the whole-course immunization.<br><br>**Phase 2:**<br>**Safety endpoints:**<br>● The primary safety endpoint is the incidence of adverse reactions observed within 7 days after each injection;<br>The secondary safety endpoints include:<br>● Incidence of any adverse reactions/events within 30 minutes after each injection;<br>● Incidence of adverse reactions observed from day 8 to 14/21/28/30, and from days 0 to

	14/21/28/30 after each injection;<br>● Incidence of serious adverse events (SAE) within 12 months.<br>**Humoral immunogenicity endpoints:**<br>● Specific ELISA antibody titers to whole SARS-CoV-2 virus, and the neutralizing antibody amounts against live SARS-CoV-2 virus. A positive antibody response (seroconversion) is defined as at least a four-fold increase in post-injection titer from baseline.<br>● Seroconversion and antibody titers (GMT, GMI) before each injection, and on days 28, 90, 180, and 360 after the whole-course immunization.

Detailed protocol for the phase 1/2 clinical trials of an inactivated vaccine (Vero cell) for the COVID-19

1. Introduction

The inactivated vaccine (Vero cell) of the COVID-19 (COVIV for short hereafter), developed by Wuhan Institute of Biological Products Co., Ltd (WIBP), is used for the immunoprophylaxis of diseases caused by COVID-19. According to the *Drug Administration Law of the People's Republic of China*, *Vaccine Administration Law of the People's Republic of China*, and *drug registration administration measures*, the National Medical Products Administration (NMPA) approved the vaccine trials. WIBP entrusted Henan Provincial Center for Disease Control and Prevention (HNCDC, as the investigator) with the vaccine trial.

A randomized double-blind, placebo-controlled trial is planned to be conducted in healthy individuals to evaluate the safety and immunogenicity of COVIV. This clinical trial protocol is formulated in accordance with the *Provisions for Drug Registration*, *Good Clinical Practice of Pharmaceutical Products* (GCP), *Technique Guideline for Clinical Trials of Vaccines*, and *Technique Guideline for Clinical Trials of Vaccines (Trial)*.

2. Responsibilities of related parties in the clinical trial

2.1 Sponsor

The sponsor: Wuhan Institute of Biological Products Co., Ltd (WIBP);

Responsibility:

- Provide the preliminary trial protocol, review and approve the final protocol and related forms and materials;
- Provide on-site application documents such as clinical trial notice and investigator's Manual (including chemical, pharmaceutical, toxicological, pharmacological and clinical data and data of

the experimental drugs);

- Provide the vaccine for clinical trials, and provide the qualified inspection report or the certificate of biological product batch issuance;
- Provide vaccine delivery to ensure safe storage and delivery of experimental vaccines;
- Provide clinical research funds;
- Designate full-time personnel to be responsible for the management of clinical trial safety information monitoring and SAE report, monitor the latest status of the whole clinical trial safety information, and timely report to all researchers, relevant parties and regulatory authorities involved in the trial;
- Participate in the investigation and treatment of adverse reactions and adverse events, and is responsible for providing medical treatment and relevant compensation fees for adverse reaction cases and adverse event cases clinically proven to be related to vaccines according to relevant regulations;
- Be responsible for sending qualified supervisors or entrusted contract research organizations to evaluate, select, and approve the clinical trial site. During the test, perform the supervision duties according to the requirements of GCP, and verify the research data;
- Organize the audit of clinical trials to ensure the quality, ensure that the clinical trials are conducted in accordance with the requirements of GCP and clinical trial regulations, and take the ultimate responsibility for the quality of clinical trials.

2.2 Investigator

Henan Province Center for Disease Control and Prevention (HNCDC);

Responsibility:

- Participate in the formulation of clinical trial protocol and is responsible for the implementation;
- Prepare and review informed consent, diary cards, electronic case report forms (ECRF), and field application forms, etc.;
- Submit the ethical review materials to the ethics committee and obtain the approval certificate;
- Establish vaccine clinical trial organization management system and quality management system, write SOP, and conduct training;
- Select the clinical trial site, organize and assist in the standardized operation of the trial site, and complete the relevant trial registration;
- Establish the emergency management mechanism and measures to prevent and deal with emergencies in vaccine trials, establish the expert team of SAE emergency treatment and obtain the technical ability to deal with SAE;
- Ensure the safe storage and delivery of vaccines and biological samples for laboratory tests;
- Recruit and enroll participants, implement the vaccination inoculation, and on-site clinical observation;
- Follow-up and collect data of adverse events from all participants, and establish a team for the report, investigation, and treatment of adverse events;
- Be responsible for collecting and completing all forms and electronic case report forms;
- Confirm the archives of the data;
- Write the clinical trial summary report;
- Manage and save trial-related materials according to GCP requirements until 5 years after the trial.

2.3 Researchers and clinical trial site

Wuzhi County Center for Disease Control and Prevention, Henan Province;

Responsibility:

- Establish a qualified team of field researchers, and provide environment and facilities that meet the requirements of clinical trials;
- Recruit and enroll the participants who meet the requirements of the protocol;
- Complete vaccination inoculation, collect biological samples, and follow-up for safety events;
- Manage adverse events and report serious adverse events as required;
- Collect the original data and input the data to electronic case report forms;
- Manage trial vaccines and biological samples in accordance with GCP requirements;
- Manage and save trial-related data according to GCP requirements until 5 years after the trial.

2.4 Laboratory measurement units

2.4.1 National Institutes for Food and Drug Control, China

Responsibility:

- Complete the blind detection of binding antibody responses in blood samples against whole SARS-CoV-2 virus with ELISA kits;
- Provide the measurement method, reference value, test standard, and provide method verification certificate if necessary.

2.4.2 Wuhan Institute of Virology, Chinese Academy of Sciences

Responsibility:

- Complete the blind detection of neutralizing antibody titers in blood samples;
- Provide the measurement method, reference value, test standard, and provide method verification certificate if necessary.

2.4.3 People's Hospital of Wuzhi County

Responsibility:

- Complete the laboratory tests for blood and urine routines on day 0 before each inoculation and day 4 after each inoculation;
- Provide the measurement method, reference value, test standard, and provide method verification certificate if necessary.

2.4.4 Zhengzhou Jinyu clinical test center Co., Ltd

Responsibility:

- Complete the cellular immune tests;
- Provide test method, reference value, test standard, and certificate of method verification if necessary.

2.4.5 Shenzhen Huada Life Science Research Institute

Responsibility: to sequence the samples collected at different time points for high-throughput single cell sequencing, analyze the changes of the type and proportion of immune cells in PBMC before and after the inoculation, as well as the expression spectrum of immune-related genes in various subpopulations of cells, and explore the host immune response, TCR characteristics of potential effector T cells and BCR characteristics of B cells.

2.5 Contract Research Organization

Beijing Zhongsheng Hengyi Pharmaceutical Technology Co., Ltd

Responsibility:

- Carry out clinical trial audit according to GCP, clinical trial protocol and SOP;
- Assist the sponsor to confirm that the investigator and site unit have the appropriate conditions to complete the trial, including personnel allocation and training, first aid room and other functional

divisions, all clinical laboratories are fully equipped and well operated, and have various conditions related to the trial

- Verify the delivery, storage, distribution, use, return and treatment of the test vaccine according to the protocol requirements during the whole process, and check the dose change and concomitant drug use of each participant;
- Confirm that all participants have signed the written informed consent and the date is signed before the trial, and confirm that the included participants are qualified;
- Audit the whole process, including the reception, informed consent, physical examination, randomization, sample collection, vaccination inoculation, post-inoculation observation, vaccine management, biospecimen management, study material management, archives management and other documents to ensure that they are implemented in accordance with the relevant requirements of GCP and the protocol;
- Confirm that the investigator receives the latest version of manual, protocol, all trial-related documents, and supplies, and implement the protocol according to the regulations. Verify that the researchers are trained and received authorization before the study;
- Make sure that the researchers keep the necessary documents according to the requirements of GCP, and the records and documents are updated in real time and well preserved;
- Determine the deviation of clinical trial from the protocol, SOP, GCP, and relevant regulations, communicate with researchers in time, and take appropriate measures to prevent future deviations. Verify that the withdrawal and loss of follow-up of the participants have been recorded in the case report form;
- The inspector should make a written report after each visit and deliver it to the sponsor, and state

the corrective measures taken or to be taken for the problems found in the audit. Truthfully record the follow-up, procedures, and measurements that the researchers fail to do, and whether the errors and omissions are corrected.

2.6 Data management unit

Beijing Zhongsheng Hengyi Pharmaceutical Technology Co., Ltd

Responsibility:

- Develop data management plan and data verification plan according to the protocol, and manage the data during the trial;
- Provide EDC and other related online services;
- Carry out data management in accordance with technical guidelines for clinical trial data management. Confirm that all data records and reports are correct and complete, all eCRFs are filled in correctly and consistent with the original data, verify that all medical reports, records, and documents provided by the researchers are accurate, complete, timely, legible, dated with test number, verify that the corrections, additions or deletions made by the logarithm data are correct, dated and justified, and have been signed by the researchers. Confirm that all adverse events are recorded, and serious adverse events are reported and recorded within the specified time;
- Clean the data, mainly including vacancy value, logic check, question management, SAE consistency check, concomitant drug use check, external data consistency check, etc.;
- Inquiry about the data, assist the researchers to verify and clarify the data; after the clarification, sort out the decision of statistical population classification according to the opinions of researchers, sponsors, statisticians, etc. Lock the database and deliver the data to the statistician for statistical analysis;

- Write data management report.

2.7 Statistical party

Teaching and Research Office of Biostatistics, School of public health, Zhengzhou University

Responsibility:

- Responsible for the preparation of randomization, sample size calculation, and statistical analysis plan;
- Write statistical analysis plan according to the protocol;
- Implement randomization and blinding;
- Check data blindly;
- Implement statistical analyses according to the proposed plan, and write the analysis report.

2.8 Data and safety monitoring board (DSMB)

The sponsor has set up a DSMB to supervise the whole process of clinical trial. DSMB is established to ensure the scientifical integration of the research, ensure the reliability of the data and avoid the conflict of interest, protect the rights and health of the participants, and provide an independent evaluation on the rationality and safety of the research project to ensure the research follows the scientific and ethical standards to the maximum extent. DSMB members are experts from different disciplines, including experts in clinical medicine, statistics, and clinical trial management.

Responsibility: DSMB will review the safety report data of the participants in accordance with the committee's regulations and audit plan. If the risk of participants is found to be increased during the study, DSMB needs to promptly inform the main investigator and the sponsor.

3. Background and principle

3.1 Disease background

Coronavirus belongs to the family coronaviridae, which is a pathogen that can spread across races and easily cause respiratory diseases.[1] Coronaviruses are divided into four genera, namely Alphacoronavirus, Betacoronavirus, Gammacoronavirus, and Deltacoronavirus. According to the gene sequence, Betacoronavirus are divided into four subgroups: A, B, C, and D coronaviruses. In 2018, the World Health Organization (WHO) further classified Betacoronavirus into five major subgroups: Embedovirus (previous A), Sarbecovirus (previous B), Merbecovirus (previous C), Nobecovirus (previous D) and Hibecovirus. The first two subpopulations mainly infect mammals, and HCoV-OC43, HCoV-229E, SARS-CoV, HCoV-NL63, HCoV-HKU1, MERS-CoV, and SARS-CoV-2 (also known as 2019-nCoV) can infect humans.[2] The SARS-CoV-2 belongs to Sarbecovirus subpopulation.[1] In 2003, severe acute respiratory syndrome (SARS) infected more than 8000 people, with a mortality rate of nearly 10%; in 2012, Middle East respiratory syndrome (MERS) infected nearly 2500 people, with a mortality rate of 37%.[2] In December 2019, an outbreak of pneumonia of unknown cause occurred in Wuhan, Hubei Province, China.[3] The patient's manifestations included fever, cough, dyspnea, and the imaging changes of pulmonary patchy and diffuse infiltration.[4] A novel coronavirus was identified from the lower respiratory tract bronchoalveolar lavage fluid samples from patients with unknown pneumonia in Wuhan. The virus was named 2019 novel coronavirus (2019-nCoV, and later as SARS-CoV-2), and the virus could cause COVID-19. The WHO listed the outbreak as a public health emergency of international concern,[5] and later claimed it as a global pandemic.

The main route of transmission is through respiratory droplets and close contact. Under the condition of exposure to high concentration aerosols in relatively closed environment, it is possible to propagate through aerosols. Because the virus can be isolated from feces and urine, attention should also be paid to the spread of aerosols or contacts caused by environmental pollution by feces and urine. All people are

susceptible. The elderly and those with chronic diseases are at a higher risk of poor prognosis. The symptoms of children are relatively mild.[6] By April 8, 2020, there were more than 1.4 million confirmed cases globally, among which more than 80 000 were from China and there were 80 000 deaths around the world.[7] The crude mortality rate is lower than that of SARS and MERS, but the number of infected persons and the total deaths are significantly higher than that of SARS and MERS.[7] Therefore, the research and development of an effective vaccine is urgent.

The preclinical studies of SARS-CoV and MERS-CoV vaccines show that some of the candidate inactivated and recombined coronavirus vaccines have certain immune pathological reactions and lung tissue damage after inoculation in some experimental animals, and may induce antiviral dependency enhancement (ADE); however, some of the candidate vaccines do not.[8] This is a potential safety issue that should be addressed in all preclinical and clinical researches on all kinds of novel coronavirus vaccines.

3.2 Pathogen background

The SARS-CoV-2 belongs to the family of coronaviruses of the order Nidovirales, with a diameter of 80-120 nm.[9] It is a non-enveloped virus with a positive-sense single-stranded RNA of 30 000 bases. The 5 'end of the RNA chain has a methylation cap and the 3' end has a PolyA tail structure.[9] This structure is very similar to eukaryotic mRNA, and it is also the important structural basis for its function of translation template. The SARS-CoV-2 first expresses RNA polymerase using viral RNA as template, and then RNA polymerases complete the transcription synthesis of negative-strand RNA, the synthesis of various structural protein mRNA, and the replication of viral genomic RNA. New coronavirus particles are assembled in the endoplasmic reticulum and secreted out of the cells through Golgi apparatus, which forms a viral replication period. The homology of its genome with that of bat-SL-CoVZC45 (MG772933.1), human SARS virus, and MERS was 86.9%, 78%, and 50%, respectively.[10] The SARS-CoV-2 is characterized as being able to use

itself as a template to guide the synthesis of virus-related proteins. Coronavirus is composed of nucleocapsid protein (N protein), spike protein (S protein), envelope protein (E protein), membrane protein (M protein), haemagglutinin esterase (HE protein), and ribonucleic acid (RNA).[2]

S protein makes the virus look like a crown, and plays a key role in recognizing and binding receptor on the surface of host cells, and mediating the fusion of virus envelope and cell membrane. Considering the amino acid sequences between SARS-CoV-2 and SARS-CoV receptor-binding domain are highly similar, the predicted protein structure shows that SARS-CoV-2 can effectively use human angiotensin converting enzyme II (ACE-2) as a receptor to enter the cell, which potentially promotes human to human transmission.[11] The results of surface plasmon resonance (SPR) analysis showed that the equilibrium dissociation constant KD (15 nm) of S-protein binding to human ACE2 of SARS-CoV-2 was much smaller than that of SARS virus (325.8 nm), which indicated that the affinity of ACE2 protein to SARS-CoV-2 was about 20 times that of SARS-CoV and explained the high transmissibility of SARS-CoV-2;[12] M-protein participated in the formation and budding process of virus envelope and transportation of nutrients; HE protein is the short bulge of the envelope, which may be related to the early adhesion of coronavirus. HE protein of some coronaviruses can cause the agglutination of red blood cells and adhesion to red blood cells.[2] Based on the current research and knowledge, SARS-CoV-2 is sensitive to UV rays and heat, and it can be effectively inactivated in 56 °C for 30 min, 75% ethanol, chlorine-containing disinfectant, peracetic acid, and chloroform. However, chlorhexidine cannot effectively inactivate the virus.[13]

3.3 Vaccine background

With the development of the COVID-19 pandemic, vaccines, small molecule drugs, serum of convalescent patients, neutralizing antibodies, and other drugs have become the mainstream means of fighting against the pandemic.[14] Vaccine experts from WHO said that there had been no vaccine against other

coronaviruses that can produce cross immunity protection against the novel coronavirus, and they were not sure about the capacity of clinical development and large-scale production of different SARS-CoV-2 vaccines. Experts suggested that, in view of the current knowledge and situation, the technical routes adopted by other coronavirus vaccines (i.e., SARS and MERS) should be prioritized for SARS-CoV-2 vaccine development, and further amelioration are warranted over time.[8]

Among all technical routes under development, nucleic acid vaccine (mRNA and DNA) and virus vector vaccine (such as MVA, VSV, Ad/Ch Ad) currently represent the verified options of technical routes theoretically. Among them, some technical routes may produce alternative vaccines on a large scale easier and faster, while some technical routes may stimulate immune protection of organisms faster and more powerfully. Other technical routes, such as subunit proteins, can also be considered, but more detailed research data are needed.[8]

Since the outbreak of COVID-19, domestic and overseas research institutions have raced to the development of effective vaccines based on the research experience in SARS and MERS virus vaccine. The China Vaccine Industry Association previously announced that 18 companies, including China Biotechnology Co., Ltd., Kangxinuo Biology Co., Ltd., Institute of Medical Biology of the Chinese Academy of Medical Sciences, and Hualan Bioengineering Co., Ltd, etc., had started the development of novel coronavirus vaccine. Several technical routes were used, such as inactivated vaccine, subunit vaccine, virus vector vaccine, DNA vaccine, and mRNA vaccine, etc. In addition to the projects funded by the Epidemic Preparedness Innovations (CEPI), most candidates may need about one year to start phase 1 clinical trials. Some of the institutions have finished early studies and started animal experiments. It is reported that the novel coronavirus vaccine mRNA-1273 developed by Moderna, a United States biotechnology company, and the National Institutes of Health launched the phase 1 clinical trial on March 16, 2020. At the same time,

the recombinant novel coronavirus vaccine (adenovirus type 5 vector) developed by Dr. Wei Chen and her colleagues from the Academy of Military Medical Sciences, PLA of China, also started the phase 1 clinical trial.[8] The current situation of the vaccine researches is shown in the Table 5.

Table 5. Current SARS-CoV-2 vaccine researches

Organization/Enterprise	Vaccine type	Latest progress
Siwei/Chinese Center for Disease Control and Prevention/ Shanghai Tongji University Medical College	mRNA vaccine	Animal experiments
Institute of Microbiology, Chinese Academy of Sciences	Recombinant protein vaccine	Animal experiments
Moderna/NIAID	mRNA vaccine	Phase 1
Adweixin/Inovio/Kangtai biology	DNA vaccine	Animal experiments
Imperial College London	undisclosed	Animal experiments
Clover biopharmaceutical	Recombinant protein vaccine	Animal experiments
University of Saskatchewan	undisclosed	Animal experiments
GSK/University of Queensland	Recombinant protein vaccine	Animal experiments
CanSinoBIO	Virus vector vaccine, mRNA vaccine	Phase 1
Zhi Fei Biological	Recombinant protein vaccine	Early R & D

Tianyuan biology	undisclosed	Early R & D
Janssen Pharmaceutical	Virus vector vaccine	Early R & D
CureVac	mRNA vaccine	Early R & D
Wuhan bowo/geovaxlabs	Virus vector vaccine	Early R & D
Li Ka Shing Faculty of Medicine	Recombinant protein vaccine	Early R & D
Yuzhibo biology	Recombinant protein vaccine	Early R & D
China National Biotec Group Company Limited	Inactivated vaccine/recombinant vaccine	Early R & D

3.3.1 Inactivated vaccine

Inactivated vaccines are produced by inactivating and purifying the cultured SARS-CoV-2, which are composed of the whole virus or its fragments. In terms of a novel infectious disease, the research and development process of inactivated vaccine is relatively mature, has clear quality control and evaluation methods, is easy to initiate the large-scale production process, and has a high safety capacity.[8] Many research institutions have isolated the virus strains. Sinopharm Wuhan Institute of Biological Products Co., Ltd. and Codagenix companies are committed to the research and development of inactivated SARS-CoV-2 vaccine.

3.3.2 Recombinant protein vaccine

Recombinant protein vaccines are vaccines produced by purification of proteins or peptides which are expressed by the target gene of the virus in cells or microorganisms. A team from the Institute of Microbiology, Chinese Academy of Sciences, is undertaking the research and development of SARS-CoV-

2 recombinant protein vaccine and has completed the design of vaccine products. The team is currently carrying out the animal experiments to evaluate the effects of the vaccine product and work related to process research and development. Specific antibodies against S-antigen of SARS-CoV-2 have been detected in the sera of many rehabilitated patients infected by 2019-nCoV.[15] Based on the S-protein of novel coronavirus, Clover pharmaceutical company has developed the "S-protein-trimer" antigen vaccine, which has been used for in vivo test. The University of Queensland has developed the first candidate COVID-19 vaccine in three weeks using this technology, aiming to conduct clinical trials in the middle of this year.[8]

3.3.3 Recombinant adenovirus vector vaccine

The recombinant adenovirus vaccine encoding MERS-CoV S1 subunit can induce potentially protective antigen-specific humoral and cellular immune responses in mice.[16] Merck's Ebola vaccine has been approved in the European Union and the United States.[17,18] In October 2017, the Ebola vaccine developed by China Kangxinuo biological Co., Ltd. using the adenovirus platform obtained the new drug registration application of China food and drug administration.[19] Now, the company has used mature adenovirus platform to develop recombinant adenovirus vector 2019-nCoV vaccine and started phase 1 clinical trials.

3.3.4 DNA vaccine

For DNA vaccines, the S-protein-based DNA vaccine developed by Inovio pharmaceutical, Adnexine, Kangtai Biology, and other companies has been tested in animal experiments. It is said that the Innovio technology platform can design INO-4800 gene vaccines in three hours, has completed the sequence research and development of DNA vaccines, and planned to start clinical trials in the United States in April, followed by in China and South Korea. Inovio has previously carried out vaccine research and development of Zika virus, Ebola virus, MERS, and other viruses, and experiments in mice showed the vaccine’s effects are promising. For the COVID-19 vaccine, Inovio expected to use existing resources to provide 1 million doses

of vaccines by the end of 2020.[8]

3.3.5 mRNA vaccine

The mRNA vaccines are related sequences of virus mRNA synthesized in vitro, which are transferred to human cells and form immunity. The production of mRNA vaccines does not depend on the process of cell expansion, so it is easier to be produced on a large scale. Moreover, the early synthesis is fast, and subsequent researches could be conducted once the effective antigens are found during animal experiments.[8] For mRNA vaccines, Stemirna Therapeutics Co., Ltd. and American Moderna announced to carry out the research work of SARS-CoV-2 mRNA vaccine almost at the same time. Stemirna Therapeutics Co., Ltd. announced that the vaccines started to be tested in animals on February 7,[20] and the Wall Street Journal reported that Moderna had launched the phase 1 clinical trial of SARS-CoV-2 mRNA vaccine on March 16.[21]

4. Product information

4.1 Characteristics of the experimental vaccine

The novel coronavirus inactivated vaccine (Vero cell line; COVIV) is produced by inoculating SARS-CoV-2 WIV04 strain in Vero cells, which is then cultured, harvested, inactivated, purified, concentrated, secondary inactivated, purified, and finally made with the adjuvant of aluminum hydroxide added. After inoculation with the vaccine, the body could produce immune response and is expected to prevent the development of COVID-19.

This product is suitable for healthy people aged 3 years and above.

4.2 Production process

4.2.1 Virus strains

A novel coronavirus strain was successfully isolated from a patient from Jinyintan Hospital in late December 2019, which was named as 2019-nCoV WIV04 strain. The novel coronavirus strain was identified, detected, and analyzed by cytopathology, immunofluorescence, virus proliferation, morphological examination, deep sequencing, evolutionary analysis, and sequence alignment, etc. Meanwhile, in the continuous passage adaptation studies, the results regarding viral cytopathic features, viral titers and antigenicity, immunogenicity, and passage stability showed that WIV04 strain had good biological characteristics and could be used for vaccine production.

4.2.2 Culture and inactivation process

The developed processes of the vaccine preparation and quality control methods were submitted for patent applications. DMEM high glucose virus maintenance solution is used for virus culture. The MOI is 0.01-0.001 for virus inoculation. After 72-96 hours of virus culture, the virus is harvested, followed by β-propiolactone-inactivation (1:4000 (v/v) at 2-8 °C for 48 hours. The inactivated virus collection solution is clarified by microfiltration and centrifugation with different particle sizes. Then, the 300KD ultrafiltration membrane is used to concentrate the harvested virus, meanwhile, small molecular proteins are partially removed. A 2nd β-propiolactone-inactivation procedure is carried out, followed by gel-chromatography, ion-exchange chromatography, sterile filtration, formulation with buffer and Aluminum hydroxide (Alum), filling, packaging and labeling. Inactivation was validated by passaging the treated samples 3 generations without appearance of cytopathic effects. The in-process quality controls were established and applied. Production was performed in good manufacturing practice (GMP) manufacturing facilities and well documented in detail. The preliminary stability of the finished products was also tested at different temperatures. The persistency of quality and quantity of each bulk and lot of preparations was determined. The residues of host Vero cell proteins, DNAs and additives in cell culture were determined by dot-blotting,

quantitative Real-Time PCR and other applicable methods.

4.3 Vaccination route and immunization procedure

Route of inoculation: lateral deltoid of upper arm, intramuscular injection**.**

Procedure: In the phase 1 and 2 trials, all participants in the low-dose, medium-dose, and high-dose groups received three doses of vaccines on 0th, 28th, and 56th days.

4.4 Preservation and transportation of vaccines

The vaccine should be stored and transported in dark place and at 2-8 °C to prevent freezing. It is necessary to monitor and record the temperature during the transportation and storage of vaccines. If the storage and transportation temperature conditions exceed the specified range, the field researchers should immediately contact the investigator and the sponsor to determine whether the vaccine can be used.

5. Preclinical study

5.1 Stability study

Wuhan Institute of Biological Products Co., Ltd established the protocol of stability study of the SARS-CoV-2 inactivated vaccine (Vero cell), including stability study at 2-8 °C, accelerated stability study at 20-25 °C and 37 °C.

For stability study at 2-8 °C, the vaccine is planned to be placed at 2-8 °C for 42 months, and the appearance, pH value, antigen content (in vitro relative potency test), in vivo relative potency test, and other indicators would be measured. This study aims to determine the vaccine’s stability and expiring date under normal storage temperature.

For accelerated stability study at 20-25 °C, the vaccine is planned to be placed at 20-25 °C for 6 weeks, and the antigen content and/or protein concentration of the vaccine stock solution and vaccine would be measured to test their stability at accelerated temperature.

For accelerated stability study at 37 °C, the vaccine is planned to be placed at 37 °C for 2 weeks, and the antigen content and/or protein concentration of the vaccine stock solution and vaccine would be measured to test their stability at accelerated temperature.

The results are as follows.

The inactivated vaccine was placed at 25 °C and 37 °C for 2 weeks. After the simultaneous desorption of vaccines used in stability study and reference vaccines, the ratio of antigen content was not changed, indicating that the antigen of the inactivated vaccine remained stable at 25 °C or 37 °C for 2 weeks.

5.2 Safety study

5.2.1 Animal allergy test

Thirty-six SPF male Hartley guinea pigs were randomly divided into four groups, i.e. high-dose group, low-dose group, negative control group, and positive control group. Vaccines were injected intramuscularly on days 1, 3, and 5 for sensitization and were injected intravenously on day 19 and/or day 26 for hypersensitive response. Vaccines or control substances were intramuscularly injected through hind limbs into all animals on Days 1, 3, and 5. On day 19, vaccines or control substances were intravenously injected into the first three animals in each group, and the animals were observed for at least 30 minutes after injection. If there were animals showing allergic reaction symptoms in each group, vaccines or control substances were injected into the remaining animals in each group on the same day. The remaining animals in the negative control group, positive control group, and groups where there were no animals showing allergic reaction symptoms would receive intravenous injection on day 26. During the test, the substances causing allergic reaction for the first time need to be tested twice.

The results are as follows.

The SARS-CoV-2 inactivated vaccine (Vero cells) was respectively intramuscularly injected by 0.1 and

1 dose per time for sensitization, and was then respectively intravenously injected by 0.2 and 2 doses per time. The injections did not cause active systemic anaphylaxis.

5.2.2 Animal acute toxicity test

Twenty SD rats (10 male and 10 female rats) were used in the experiment. They were randomly divided into two groups according to their sex, i.e. negative control group and vaccine test group, and there were 5 male and 5 female rats in each group. Each rat in negative control group received 2 mL of sodium chloride injection intramuscularly, while each rat in vaccine test group received 2 mL/4 doses of inactivated vaccine (Vero cell). During the experiment, acute toxic reaction symptoms were observed for four hours after injection among all animals and also observed once in the morning as well as once in the afternoon for 14 days; body weight and food intakes of rats were also measured regularly. At the end of the observation period (day 15), the animals were euthanized and observed by anatomy.

During the test, no animals died or were dying, and no obviously abnormal clinical reactions were found. Compared with the animals from negative control group of same sex in the same duration, there was no statistical difference in body weight and food intakes in vaccine group ($P > 0.05$). The gross anatomy of pathology showed that there was no abnormal change in main organs and tissues of each group. Thus, when the inactivated vaccines (Vero cells) were intramuscularly injected into rats by 4 doses per time, SD rats showed no obviously abnormal reactions. The maximum tolerance dose (MTD) of animals was greater than 4 doses per rats.

5.2.3 Long term toxicity test

5.2.3.1 Rats:

Methods: 150 SD rats, half male and half female, were randomly divided into 7 groups according to their weight. There were 120 rats in the main experimental group (groups 1 to 4, 15 rats/sex/group) used for

toxicological study and 30 rats in the satellite group (groups 5 to 7, 5 rats/sex/group) used for determination of antibody.

The inactivated vaccines (Vero cells) were administered to the low-dose group by 1 dose/rat and the high-dose group by 3 doses/rat, and each dose contained 0.5 mL of vaccines. The negative control group received 1.5 mL/rat of sodium chloride injection, and the adjuvant control group received 1.5 mL/rat of control adjuvant. All animals were given intramuscular injection once a week for 2 consecutive weeks (a total of 3 times). Clinical observation, measurements of weight, food intakes, and body temperature, ophthalmology examination, clinical pathology (blood cell count, coagulation function, blood biochemistry, and urine analysis), and measurements of T lymphocyte subsets ($CD3^+$, $CD3^+CD4^+$, $CD3^+CD8^+$, and $CD3^+CD4^+/CD3^+CD8^+$), serum cytokines (IL-2, IL-6, IL-10, TNF alpha, and IFN- gamma), and SARS-CoV-2 specific antibody and detection of neutralization activity were performed.

The first 10 rats of each sex in each group were euthanized on day 18 (3 days after the last administration), and the rest animals were euthanized at the end of the two-week recovery period (on day 29). All animals were observed in gross anatomy, and the main organs were weighed to calculate the ratio of viscera to body and the ratio of viscera to brain. Pathological examination was also carried out for various tissues and organs.

Results: During the experiment, no rats died or were dying in each group, and no abnormal reactions related to vaccines were observed. Compared with the animals from negative control group of same sex in the same duration, there were no obvious or significant differences in body weight, food intakes, body temperature, ophthalmology examination, clinical pathology except for blood cell count, and T lymphocyte subsets ($CD3^+$, $CD3^+CD4^+$, $CD3^+CD8^+$, and $CD3^+CD4^+/CD3^+CD8^+$) in adjuvant control group, low-dose group, and high-dose group. Gross anatomy for euthanized rats showed no abnormalities.

Compared with the animals from negative control group of same sex in the same duration, after the recovery period, IL-6 level in low-dose and high-dose groups increased, and IL-6 levels were statistically different among the female rats, which might be related to the administration. In addition, there was no abnormal change of cytokines (IL-2, IL-10, TNF-α, and IFN-γ) in each group. On the third day after the last administration (day 18), no toxically pathological changes related to vaccines or adjuvants were observed using microscopes, except for the injection site. In the injection site, 20 animals respectively in the adjuvant control group, low-dose group, and high-dose group showed mild to moderate granulomatous inflammation (macrophage). Since the changes were observed in adjuvant control group, low-dose group, and high-dose group, and there were no significant differences in the incidence and the degree of lesions, we believed that the changes might be related to aluminum adjuvant instead of the vaccine.

Antibody tests showed the inactivated vaccine could induce the neutralization antibody and the specific antibody IgG in SD rats.

(1) For serum neutralizing antibody, after two vaccinations, all animals in the low-dose and high-dose groups produced neutralizing antibody, and the GMT of neutralizing antibody in the low-dose and high-dose groups were 505 and 1047. After the second and third vaccinations, the titer of neutralizing antibody in the low-dose and high-dose groups continued to rise, and the GMT of neutralizing antibody in the high-dose group was 2177. Before the end of the recovery period (day 28), no significant reduction was observed for the GMT of neutralizing antibody in the low-dose and high-dose groups.

(2) For serum binding antibody (specific IgG antibody), all animals in the low-dose and high-dose groups produced binding antibody before the second vaccination. After the second and third vaccinations, the titer of binding antibody in the low-dose and high-dose groups continued to rise until the end of the recovery period (day 28), and the GMT of binding antibody in the low-dose and high-dose groups did not

decrease significantly.

Conclusion: The inactivated vaccines (Vero cells) were intramuscularly injected into the SD rats by one dose and three doses per time. The injections were given once a week for two consecutive weeks (a total of three times). No systematic toxic reactions were observed 3 days after the last vaccination (day 18). Before the end of the recovery period (day 28), the binding antibody and neutralizing antibody increased significantly and showed obvious dose-effect and immunity enhancing effects. At the end of the 2-week recovery period, the antibody titer did not decrease. No pathological changes were observed except for local stimulation. During the experiment, compared with the animals from negative control group of same sex in the same duration, no obvious or significant changes of weight, food intakes, body temperature, ophthalmologic examination, clinical pathology (except blood count), and T lymphocyte subsets ($CD3^+$, $CD3^+$ $CD4^+$, $CD3^+$ $CD8^+$, and $CD3^+$ $CD4^+$/$CD3^+$ $CD8^+$) were observed in adjuvant control group, low-dose and high-dose groups.

5.2.3.2 Monkeys

Conventional Macacafascicularis (half males and half females) were given intravascular injection for 4 weeks continuously (a total of four times, and on days 1, 8, 15, and 29). During the experiment, the animals were observed routinely twice a day. The animals were taken out of the cage once a week for detailed clinical observations. The injection sites were observed once on the day before and after each injection as well as once a week. At the same time, weight, body temperature, ECG, blood pressures were monitored, and ophthalmic examinations, clinical pathological examinations, immunological tests, antibody examinations, and body and histopathological examinations were carried out.

Results: During the experiment, no animals died or were dying in each group. One male animal in the

negative control group was euthanized on the 10th day after the third vaccination (day 25), and gastrointestinal reaction and myocarditis were observed. There was no abnormal reaction related to vaccines. No erythema, hyperemia, swelling, ulcer, and induration were observed in injection sites.

During the experiment, there was no abnormality in the ophthalmic and urine examination in each group. Compared with the animals from negative control group of same sex in the same duration, there was no significant difference in weight, coagulation function, and complement in each group ($P > 0.05$). Body temperature, ECG parameters, blood pressure, blood oxygen saturation, blood cell count, blood biochemistry, lymphocyte subpopulation, cytokines, and C-reactive protein were observed to be statistically significantly different among some groups at some time points ($P \leq 0.05$). But when comparing them at different time points, the above differences were not clearly related to the dosage or administration time, so we believed the differences had no toxicological significance.

For serum antibody, the inactivated vaccine can induce corresponding new anti-coronavirus binding antibody (specific IgG antibody) and neutralizing antibody in Macaca Fascicularis. For serum binding antibody (specific IgG antibody), all animals in the low-dose and high-dose groups produced binding antibody, and the GMT of binding antibody in the high-dose group was 2111 after two vaccinations. Between the third (day 21) and fourth (day 28) vaccination, the titer of binding antibody kept rising in the low-dose and high-dose groups, and the GMTs of binding antibody in the high-dose group were 8445 and 16890. There were no significant reductions in GMT in the low-dose and high-dose groups until the end of the recovery period (day 43). For serum neutralizing antibody, all animals in the low-dose and high-dose groups produced neutralizing antibody, and the GMT of neutralizing antibody in the high-dose group was 780. After the third vaccination, the titer of neutralizing antibody in the low-dose and high-dose groups kept rising, and the GMT of neutralizing antibody in the high-dose group was 1640. Between the fourth vaccination and the

end of the recovery period (day 43), the neutralizing antibody levels increased significantly in the low-dose group and remained consistent in the high-dose group.

For pathological examination, on the third day after the last vaccination and at the end of the 2-week recovery period, there were no systematic changes of the organ weight/organ coefficient in the adjuvant control group, low-dose group, and high-dose group. There was no obvious systemic toxic pathological change in the gross anatomy and microscopic observation.

On injection sites, six animals respectively in the adjuvant control group, low-dose group, and high-dose group showed mild to moderate granulomatous inflammation (macrophage) on the third day after the last vaccination. We speculated that the changes might be related to aluminum adjuvant and were expected reactions caused by intramuscular injection of the vaccine containing aluminum adjuvant. At the end of the two-week recovery period (day 43), 4 animals respectively in the adjuvant control group, low-dose group, and high-dose groups showed macrophage granulomatous inflammation, suggesting that the topical stimulation of the drug administration has not yet fully recovered.

Conclusions: the inactivated vaccines (Vero cells) were injected into Macaca Fascicularis by 1 dose and 4 doses each time. The injections were given weekly or biweekly for four consecutive weeks (a total of four times). The animals had no obvious systemic toxic reaction during the period between vaccinations and the end of two-week recovery period, thus we thought that the no observed adverse effect level (NOAEL) was 4 doses per animal.

During the period between vaccinations and the end of two-week recovery period, there were irritative effects related to aluminum adjuvants on injection sites, and specific IgG antibody and neutralizing effects were detected in animals; and immune toxicity was not observed.

5.3 Immunogenicity

The immunogenicity studies on the vaccine in mice, rats, guinea pigs, rabbits, and rhesus monkeys have been completed.

5.3.1 Immunogenicity in mice

Three dose groups (i.e. 40 WU/dose, 200 WU/dose, and 1000 WU/dose) were selected, and each dose group was divided into two immune procedure groups. Vaccinations were inoculated on days 0, 7, and 14 through intraperitoneal injection in three-dose groups, and on days 0 and 14 through intraperitoneal injection in two-dose groups. There were five mice in each group. Blood samples were collected on days 0, 14, and 21 in three-dose groups and on days 0, 14, and 28 in two-dose groups. The results showed that the vaccines could produce specific binding antibody and neutralizing antibody when using different immune procedures, indicating that the antigen purified by the current process had good immunogenicity. However, the immune response levels were different when using different immune procedures, and an interval of 14 days (S14) is better than an interval of 7 days (S7), indicating that vaccines can stimulate the production of neutralizing antibodies in more mice and higher titers of binding and neutralizing antibodies when the interval between injections was prolonged.

5.3.2 Immunogenicity in rats

Two dose groups (i.e. 200 WU/dose and 1000 WU/dose) were set, and each dose group was divided into two groups (with and without adjuvant). There were five mice in each group. Vaccinations were provided on days 0, 7, and 14 through intramuscular injection. Blood samples were collected on days 0, 7, 14, and 21. The results showed that the immune response level was higher in rats compared with that in mice (S7), and the titers of neutralizing antibodies were higher in rats when comparing the corresponding vaccination. Besides, the GMTs of serum neutralizing antibodies were higher in rats compared with that in mice, regardless of the adjuvant control group, medium-dose group, or high-dose group.

5.3.3 Guinea pigs and rabbits

5.3.3.1 Guinea pigs

Guinea pigs were inoculated subcutaneously with 3 doses (200 WU/dose) of vaccines on days 0, 7, and 14. Blood samples were collected on days 0, 7, 14, and 21. There were 5 guinea pigs in each group.

The results of immunogenicity in guinea pigs were basically similar to those in mice and rats.

(1) The neutralizing antibody titer increased significantly with more vaccinations were provided.

(2) Three vaccinations on days 7, 14, and 21 showed a good immune enhancing effect.

(3) There were significant differences in neutralizing antibody levels between the second (day 14) and first (day 7) vaccination as well as between the third (day 21) and second (day 14) vaccinations.

5.3.3.2 Rabbit

Rabbits were inoculated subcutaneously by 1000 WU/dose of vaccines. Rabbits in the three-dose group were injected on days 0, 7, and 14, and rabbits in the two-dose group were injected on days 0 and 14. Blood samples were collected on days 0, 7, 14, and 21 in the three-dose group and on days 0, 14, and 28 in the two-dose group. There was one rabbit in each group.

Results showed that the neutralizing antibody titer increased significantly with more vaccinations were provided in both groups. Basically, the results of immunogenicity in rabbits were similar to those in mice, and vaccines can stimulate higher production of neutralizing antibodies when the interval between injections was prolonged.

5.3.4 Immunogenicity in rhesus monkeys

Three groups (i.e. 200 WU/dose, 1000 WU/dose, and adjuvant control group) were set. Vaccinations were conducted on days 0 and 14 via intravascular injections. There were two monkeys in the adjuvant control group and three monkeys in other groups. Blood samples were was collected on days 0, 7, 14, and

21.

The results showed that the vaccine had good immunogenicity and could stimulate high production of neutralizing and binding antibodies in rhesus monkeys.

In conclusion, the inactivated vaccine has good immunogenicity in different types of animals. Neutralizing antibodies and binding antibodies could be produced on the 7th day after the first vaccination. Another vaccination on day 7 or 14 after the first vaccination could stimulate a high production of neutralizing antibodies and binding antibodies. Various strands of novel coronavirus have been isolated, and the results of the cross neutralization test showed that vaccine-immuned serum can effectively neutralize other isolated strains.

5.4 Animal protection effect

(1) Experimental animals

Rhesus monkeys, a non-human primate experimental animal, were selected for the experiment. They were 6-8 years old, half male and half female. There were three in each group of the experimental group and two in the control group.

(2) Inactivated vaccine preparation

The inactivated vaccine (Vero cell) was developed by the Wuhan Institute of Biological Products Co., Ltd and the Wuhan Institute of Virology of the Chinese Academy of Sciences. The aluminum hydroxide adjuvant was derived from CrodaDenmark Company.

(3) Immune procedure

The control group was inoculated with 0.5 mL of aluminum hydroxide adjuvant on days 0 and 14,

respectively. Vaccines with aluminum hydroxide adjuvants were inoculated to low-dose and high-dose groups on days 0 and 14, respectively.

(4) Method and doses of virus in challenge test

The experimental animals were transferred to the fourth level biosafety laboratory on the 21st day after vaccination. On the 24th day after vaccination, all animals in the control group, high-dose group, and low-dose group received 1×10^6 $TCID_{50}$ of virus (1 mL) by tracheal intubation.

(5) Results

The inactivated vaccine can induce specific antibody and neutralizing antibody in experimental animals, which showed certain dose-effect relationship. After receiving 1×10^6 $TCID_{50}$ novel coronavirus through tracheal intubation, proliferation of virus was observed in lungs of animals in the control group. In the high-dose and low-dose groups, antibodies could completely inhibit the replication of virus in animals when the serum neutralizing antibody level was high, while antibodies can only partially inhibit the replication of virus in animals when the serum neutralizing antibody level was low, indicating that the inactivated virus vaccine had a protective effect on preventing and reducing the replication of virus in animal bodies.

High dose of virus infection could lead to moderate to severe viral pneumonia and acute alveolar injury in the control group, while the lung injury was mild or mild to moderate in low-dose and high-dose groups, which was weaker than that in control group and could heal later.

No ADE was found in this experiment.

5.5 Summary of preclinical research

Through preclinical researches, the preparation and quality standard of the inactivated vaccine (Vero cell) have been established. The allergy experiment and acute toxicity experiment in animals were completed, and we are performing the interim analysis of long-term toxicity tests in the SD rats. There are no

abnormalities observed in the monkeys (complete report will be released later), and the current results of safety assessment suggested that the vaccine is safe.

The results of immunogenicity test showed that the vaccine had good immunogenicity in different animals. Neutralizing antibodies and binding antibodies could be produced on the 7th day after the first vaccination. Another vaccination on day 7 or 14 after the first vaccination could stimulate a high production of neutralizing antibodies and binding antibodies. According to the current results of safety and immunogenicity studies, the present inactivated vaccine met the requirements of the *new coronavirus inactivated vaccine (Vero cell) verification regulation (Draft)*, and the quality of vaccines is manageable, safe, and effective.

The results are to be submitted.

6. Research objectives

To evaluate the safety of the inactivated vaccine (Vero cell) in healthy children aged 3-17 years old by inoculating different doses of vaccines, and to initially explore the immunogenicity and tolerability of the vaccine.

6.1 Main purpose

To evaluate the safety of an inactivated vaccine (Vero cells) for the COVID-19 with different doses and different immune procedures in healthy children aged 3-17 years old.

6.2 Secondary purpose

To investigate the immunogenicity and tolerability of an inactivated vaccine (Vero cells) for the COVID-19 with different doses and different immune procedures in healthy children aged 3-17 years old.

7. Study design

Two randomized, double-blind, placebo-controlled phase 1/2 trials.

7.1 Procedures and methods

The phase 1/2 clinical trials are 2 randomized, double-blind and placebo-controlled trial. Participants are divided into three dose groups, i.e. low-dose, medium-dose, and high-dose groups. In the phase 1 trial, 3 doses of vaccine or placebo are administered to participants on day 0, day 28, and day 56, and the number of participants in each dose group is three times as many as the number of participants in the placebo group (3:1 ratio). In the phase 2 trial, three doses of vaccine (in low, medium, and high doses, respectively) or placebo are administered to participants on day 0, day 28, and day 56 with increased sample sizes, but same 3:1 ratio.

The DSMB has been set up to assess the risk of clinical trial, so as to ensure the trial could be conducted safely according to the protocol and standards.

The vaccines are administered in the low-dose group among those aged 3-17 years first, followed by the medium-dose and high-dose group. Meanwhile, after the safety assessment on the 8th day after the first dose among healthy children aged 3-17 years in the low-dose group first, followed by the medium-dose and high-dose group. Among all participants in the phase 1 trial, laboratory routine tests are conducted before each inoculation and on the 4th day after the inoculation. After the safety assessment on the 8th day after the first dose, the next higher dose in phase 1 trial as well as the corresponding dose group in phase 2 trial could be inoculated if the preliminary assessment of safety meets the criteria of the protocol. Given the urgency, the phase 2 trial can be timely carried out according to the real situation at the clinical site.

The chronological order of the first-dose inoculation in different dose groups is shown in the Table 6.

Table 6. The chronological order of the first-dose inoculation in different dose and age groups in phase 1 and II clinical trials during the first stage

Age group	13-17			6-12			3-5		
Dose group	Low	Medium	High	Low	Medium	High	Low	Medium	High
Day 0	◆I								
Day 8	◆II	◆I		◆I					
Day 16		◆II	◆I	◆II	◆I		◆I		
Day 24			◆II		◆II	◆I	◆II	◆I	
Day 32						◆II		◆II	◆I
Day 40									◆II

Table 7. Sample size and procedure of the phase 1 trial

Age group	Group	Dose	Timing of inoculation	N	Safety assessment	Immunogenic blood collection	
6-17	A1	L	Days 0, 28, 56	32	1. Blood and urine samples were collected before and 4 (+ 3 days) after each dose of immunization for blood biochemistry, blood routine and urine routine tests; 2. Collect AE and SAE within 0-7 days and 8-28 / 30 days after each dose of immunization; 3. Collect SAE within 12 months after the whole process exemption.	The first dose: (1) before; (2) the 4th day after; (3) the 14th day after; The second dose: (4) before; (5) the 4th day after; (6) the 14th day after; The third dose: (7) before; (8) the 4th day after; (9) the 28th day after; (10) the 90th day after; (11) the 180th day after; (12) the 360th day after.	/
	A2	P		12			
	A3	M		32			
	A4	P		12			
	A5	H		32			
	A6	P		12			
3-5	E1	L	Days 0, 28, 56	24			
	E2	P		8			
	E3	M		24			
	E4	P		8			
	E5	H		24			
	E6	P		8			

Note: (1) the blood collection window period is +3 days on the 4th day after each dose of inoculation, +7 days on the 14th after the first dose of inoculation, +7 days on the 14th day after the 2nd and 3rd dose of inoculation, +10

days on the 28th day after inoculation, and +20 days on the 90th, 180th and 360th day after the whole dose of inoculation.

(2) Before and on the 4th day after each dose of inoculation, in addition to antibody test, blood routine test and biochemical test are still needed;

(3) The participants use the diary card to record the adverse events 0-7 days after the inoculation and the contact card to record the adverse events 8-28/30 days after the inoculation;

(4) Vaccine inoculation window period +7 days.

(5) L: low dose; M: medium dose; H: high dose

Table 8. Parameters related to cellular immune safety outcomes

Lymphocyte count	Cytokine
(1) Detection of lymphocyte subsets absolute count and relative ratio (T/B/NK cells);<br>(2) Activated monocyte;<br>(3) NK cell subpopulation (mature NK cell, immature NK fine);<br>(4) Regulatory T cell subsets	IL-1β、IL-2、IL-4、IL-5、IL-6、IL-8、IL-10、IL-12p70、IL-17A、IL-17F、IL-21、IFN -γ、TFN -α、TFN -β;

The sample size and procedures of the phase 2 trial are shown in the Table 4.

Table 9. Sample size and procedure of the phase 2 trial

Groups	Dose	procedure	N	Safety assessment	Immunogenic blood collection
A1	L	Day 0, 28, and 56	84	1. Collect AE and SAE within 0-7 days and 8-14 / 21 / 28 / 30 days after each dose of immunization; 2. Collect SAE within	① before the first dose; ② before 2 doses; 3 doses ③ before immunization and ④ on the 28th day after immunization; The 90th day ⑤ 180th day ⑥ 360th day ⑦ after the whole process free
A2	P		28		
A3	M		84		
A4	P		28		
A5	H		84		

A6	P		28	12 months after the whole process exemption.	
E1	L	Day 0, 28, and 56	60		
E2	P		20		
E3	M		60		
E4	P		20		
E5	H		60		
E6	P		20		

Note: (1) the window period of blood collection on the 28th day after inoculation is +10 days, and the window period of blood collection on the 90th, 180th and 360th day after inoculation is +20 days；

(2) The participants use the diary card to record the adverse events between day 0 and day 7 after the inoculation, and use the contact card to record the adverse events on days 8-14/21/28/30 after the inoculation;

(3) Vaccine inoculation window period is +7 days.

In the phase 2 trial, in addition to the blood sample collection and antibody measurements shown in the Table 9, a block of eight people will be respectively selected from the middle-dose group (Group A3 and A4) to collect additional 10ml of blood samples before and 14 days after immunization, and on the 28th, 90th, 180th, and 360th days after the whole process in order to isolate PBMC for high-throughput single cell sequencing. The window period of blood collection on day 14 after each dose of inoculation is +10 days, and the window period of blood collection on other time points is consistent with Table 7. The results could be used to explore the immune response of hosts, TCR characteristics of potential effector T cells, and BCR characteristics of B cells.

7.2 Control group

At present, no COVID-19 vaccine is available worldwide. In order to ensure the scientific evaluation of the vaccine and considering that the final products contain adjuvant, we choose the same dose of aluminum hydroxide

adjuvant as placebo control.

7.3 Investigational vaccine

7.3.1 Different doses

Investigational vaccine 1:

COVID-19 inactivated vaccine (Vero cells) (low dose);

Manufacturer: Wuhan Institute of Biological Products Co., Ltd; Wuhan Institute of Virology, Chinese Academy of Sciences;

Specification: 100 WU/dose per time for human use, 0. 5 mL/dose;

Storage conditions: 2-8 °C;

Vaccine batch No.: 202003001, expiry date: 2023/03/12;

Investigational vaccine 2:

COVID-19 inactivated vaccine (Vero cells) (medium dose);

Manufacturer: Wuhan Institute of Biological Products Co., Ltd; Wuhan Institute of Virology, Chinese Academy of Sciences;

Specification: 200 WU/dose per time for human use, 0.5 mL/dose;

Storage conditions: 2-8 °C;

Vaccine batch No.: 202003002, expiry date: 2023/03/12;

Investigational vaccine 3:

COVID-19 inactivated vaccine (Vero cells) (high dose);

Manufacturer: Wuhan Institute of Biological Products Co., Ltd; Wuhan Institute of Virology, Chinese Academy of Sciences;

Specification: 400 WU/dose per time for human use, 0.5 mL/dose;

Storage conditions: 2-8 °C;

Vaccine batch No.: 202003001, expiry date: 2023/03/12;

7.3.2 Placebo control

COVID-19 inactivated vaccine (Vero cell) aluminum adjuvant

Active ingredient: none;

Virus content: None;

Adjuvant: aluminum hydroxide

Auxiliary materials: sodium chloride, disodium hydrogen phosphate, potassium chloride, potassium dihydrogen phosphate;

Manufacturer: Wuhan Institute of Biological Products Co., Ltd;

Storage conditions: 2-8 °C;

Specification: 0.5 mL per time for human use, without SARS-CoV-2 antigen.

Batch number and expiry date: details are reported in the *inspection report* of CFDA

7.4 Endpoints

7.4.1 Endpoints of the phase 1 trial

Safety endpoints:

- The primary safety endpoint is the incidence of adverse reactions within 7 days after each injection;

  The secondary safety endpoints include:

- Incidence of any adverse reactions/events within 30 minutes after each injection;
- Incidence of abnormal hepatorenal function and abnormal parameters of blood and urine routine before and on the 4th day after each injection;

- Lymphocyte subsets and cytokine measures before each injection, on day 14 after the first and second injection, before the third injection, and on days 28, 180, and 360 after the third injection;
- Incidence of adverse events from day 8 to 28/30, and from days 0 to 28/30 after each injection;
- Incidence of serious adverse events (SAE) within 12 months.

Humoral immunogenicity endpoints:

- Specific ELISA antibody titers to whole SARS-CoV-2 virus, and the neutralizing antibody amounts against live SARS-CoV-2 virus. A positive antibody response (seroconversion) is defined as at least a four-fold increase in post-injection titer from baseline.
- Seroconversion and antibody titers (GMT, GMI) before each injection, on days 4 and 14 (except for the third injection among those aged 3-17 years) after each injection and on days 28, 90, 180, and 360 after the whole-course immunization.

7.4.2 Endpoints of the phase 2 trial

Safety endpoints:

- The primary safety endpoint is the incidence of adverse reactions observed within 7 days after each injection;

  The secondary safety endpoints include:
- Incidence of any adverse reactions/events within 30 minutes after each injection;
- Incidence of adverse reactions observed from day 8 to 14/21/28/30, and from days 0 to 14/21/28/30 after each injection;
- Incidence of serious adverse events (SAE) within 12 months.

Humoral immunogenicity endpoints:

- Specific ELISA antibody titers to whole SARS-CoV-2 virus, and the neutralizing antibody amounts against

live SARS-CoV-2 virus. A positive antibody response (seroconversion) is defined as at least a four-fold increase in post-injection titer from baseline.

- Seroconversion and antibody titers (GMT, GMI) before each injection, and on days 28, 90, 180, and 360 after the whole-course immunization.

7.5 Safety assessment

7.5.1 Parameters

(1) Adverse events between days 0 and 7

Adverse events on injection sites (topical) include pain, induration, swelling, rash, flush, and pruritus.

Adverse events on other sites (systemic) include fever, diarrhea, constipation, dysphagia, anorexia, vomiting, nausea, myalgia, arthralgia, headache, coughing, dyspnea, pruritus, mucocutaneous abnormalities, acute allergic reaction, and fatigue.

Other adverse events include any adverse events or medical events other than the aforementioned events during the clinical trial, such as acute illness, accidental injury, etc.

(2) Adverse events between days 8 and 30

Any medical events which occur between days 8 and 30, such as acute illness, accidental injury, etc.

(3) Laboratory tests

- Blood biochemical parameters include ALT, AST, total bilirubin, creatinine, and urea nitrogen.
- Blood routine parameters include hemoglobin, leukocyte count, lymphocyte, neutrophil, and platelet.
- Urine routine parameters include urine protein, urine sugar, and urine blood cells (microscopic examination).

- Lymphocyte subsets measures included lymphocyte subsets absolute count and relative ratio test (T/B/NK cells), activated monocytes, NK cell subsets (mature NK cells and immature NK cells), and regulatory T cell subsets. The cytokine measures included IL-1β、IL-2、IL-4、IL-5、IL-6、IL-8、IL-10、IL-12p70、IL-17A、IL-17F、IL-21、IFN -γ、TFN -α、TFN -β.

7.5.2 Classification of adverse events

According to the *Guiding Principles for Classification of Adverse Events in Clinical Trials of Preventive Vaccines* (No. 102 announcement released by National Medical Products Administration in 2019), topical adverse events, systemic adverse events, vital signs, and parameters of laboratory tests are determined after vaccination.

Table 10. Grading of adverse events on injection sites (topical)

Symptoms		Level 1	Level 2	Level 3	Level 4
Pain		No or slight impact on physical activity	Affect physical activity	Affect daily life	Loss of basic self-care ability or hospitalization
Induration* and swelling**#	>14 years old	Diameter of 2.5 ~ <5 cm or area of 6.25 ~ <25 cm^2, not or slightly affect daily life	Diameter 5 ~ <10 cm or area 25 ~ <100 cm^2 or affect daily life	Diameter ≥10 cm or area ≥100 cm^2 or ulceration or secondary infection or phlebitis or aseptic abscess or wound drainage or serious impact on daily life	Abscess, exfoliative dermatitis, dermal or deep tissue necrosis
	≤14 years	Diameter <2.5 cm	Diameter ≥2.5	Area ≥50% of the inoculated	Abscess,

	old		cm, and area <50% of the inoculated limb (anatomically, the limb of the inoculated site, such as upper arm or thigh)	limb, or ulceration or secondary infection or phlebitis or wound drainage	exfoliative dermatitis, dermal or deep tissue necrosis
Rash * and erythema**#	>14 years old	Diameter of 2.5 ~ <5 cm or area of 6.25 ~ <25 cm^2, not or slightly affect daily life	Diameter 5 ~ <10 cm or area 25 ~ <100 cm^2 or affect daily life	Diameter ≥10 cm or area ≥100 cm^2 or ulceration or secondary infection or phlebitis or aseptic abscess or wound drainage or serious impact on daily life	Abscess, exfoliative dermatitis, dermal or deep tissue necrosis
	≤14 years old	Diameter <2.5 cm	Diameter ≥2.5 cm and area <50% of the inoculated limb (referring to the limb of the inoculated part in anatomical sense,	Area ≥50% of inoculated limb or ulceration or secondary infection or phlebitis or wound drainage	Abscess, exfoliative dermatitis, dermal or deep tissue necrosis

			such as upper arm or thigh)		
Pruritus		Pruritus on the inoculation site, relieved by itself or within 48 hours after treatment	Pruritus on inoculation site did not relieve within 48 hours after treatment	Affect daily life	NA

Note: *In addition to direct measurement of diameter for grading, the progress and change of measurements should also be recorded.

**The maximum measured diameter or area should be used.

#The evaluation and grading of induration and swelling, or rash and redness should be based on the functional grade and the actual measurements, and the indexes with higher grading should be selected.

Table 11. Grading of adverse events on other sites (systemic)

Symptoms		Level 1	Level 2	Level 3	Level 4
Fever * (axillary temperature ℃)	>14 years	37.3 ~ <38.0	38.0 ~ <38.5	38.5 ~ <39.5	≥39.5, lasting for more than 3 days
	≤14 years	37.3 ~ <38.0	38.0 ~ <39.5	≥39.5	≥39.5, lasting for more than 5 days
Diarrhea		Slight or transient, 3-4 times/day, abnormal fecal characteristics, or slight diarrhea lasting	Moderate or persistent, 5-7 times/day, abnormal fecal characteristics,	>7 times/day, abnormal fecal characteristics, or hemorrhagic diarrhea, orthostatic hypotension,	Hypotension shock, requiring hospitalization

	less than 1 week	or diarrhea > 1 week	electrolyte imbalance, need intravenous infusion > 2L	
Constipation*	Need stool softener and diet adjustment	Need defecation medicine	Intractable constipation needs manual dredging or enema	Toxic megacolon or intestinal obstruction
Dysphagia	Mild discomfort when swallowing	Restricted diet	Limited diet and conversation; unable to eat solid food	Unable to eat liquid food; requiring intravenous nutrition
Anorexia	Decreased appetite, but not reduced food intake	Decreased appetite and food intake, but no significant weight loss	Loss of appetite and weight	Interventions needed (e.g. tube feeding, parenteral nutrition)
Vomiting	1-2 times/24 hours without affecting the activity	3-5 times/24 hours or limited activity	More than 6 times in 24 hours or need intravenous rehydration	Need to be hospitalized or other ways of nutrition due to hypotension shock
Nausea	Transient (<24 hours) or intermittent and food intake is basically normal	Continuous nausea leads to reduced food intake (24-48 hours)	Persistent nausea results in almost no food intake (>48 hours) or need intravenous rehydration	Life threatening (such as hypotension shock)
Myalgia (on non-injection sites)	No impact on daily activities	Slightly affect daily activities	Severe muscle pain, serious impact on daily	Emergency or hospitalization

			activities	
Arthralgia	Mild pain, no impairment of function	Moderate pain; requires painkillers and/or pain impairs function but does not affect daily activities	Severe pain; need painkillers and/or pain affects daily activities	Disability pain
Headache	No impact on daily activities, no need for treatment	Temporary, slight impact on daily activities, may need treatment or intervention	Seriously affecting daily activities, requiring treatment or intervention	Intractable, requiring emergency treatment or hospitalization
Coughing	Transient, no treatment required	Persistent cough, effective treatment	Paroxysmal cough, uncontrollable by treatment	Emergency or hospitalization
Dyspnea	Dyspnea during exercise	Dyspnea due to normal activities	Dyspnea at rest	Dyspnea, requiring oxygen therapy, hospitalization or assisted respiration
Pruritus on non-injection site (no skin damage)	Mild, not or slightly affect daily life	Pruritus affects daily life	Pruritus leads to carry out daily life	NA
Mucocutaneous	Erythema/pruritus/colo	Diffuse	Blister/exudation/desquam	Exfoliative dermatitis involving

abnormalities	r change	rash/macula/dryness /desquamation	ation/ulcer	mucosa, erythema multiforme, or Stevens Johnson syndrome
Acute allergic reaction**	Local urticaria (blister), no treatment required	Local urticaria, requiring treatment or mild angioedema, not requiring treatment	Extensive urticaria or angioedema requiring treatment or mild bronchospasm	Anaphylactic shock or life-threatening bronchospasm or laryngeal edema
Fatigue	No impact on daily activities	Affect normal daily activities	Seriously affect daily activities, unable to work	Emergency or hospitalization

Note: *Axillary temperature is commonly used in China, and it could be converted to oral temperature and anal temperature if necessary. Generally, oral temperature = axillary temperature + 0.2 °C, and anal temperature = axillary temperature + (0.3 ~ 0.5 °C). The causes of high fever should be determined as soon as possible.

**For constipation, we should pay attention to the changes before and after vaccination.

Table 12. Grading according to blood biochemical examination

Parameters	Level 1	Level 2	Level 3	Level 4
Liver function (elevated ALT and AST)	1.25 ~ <2.5 × ULN	2.5 ~ <5.0× ULN	5.0 ~ <10× ULN	≥10×ULN
Elevated total bilirubin (mg/dl; μ mol/L)	1.1 ~ <1.6× ULN	1.6 ~ <2.6× ULN	2.6 ~ 5.0× ULN	≥5.0×ULN
Creatinine	1.1 ~ 1.5× ULN	1.6 ~ 3.0× ULN	3.1 ~ 6× ULN	>6× ULN
Urea nitrogen (BUN)	1.25 ~ 2.5× ULN	2.6 ~ 5× ULN	5.1 ~ 10× ULN	>10× ULN

Note: ULN refers to the upper limit of normal value range.

Table 13. Grading according to blood routine examination

Parameters	Level 1	Level 2	Level 3	Level 4
Increased leukocytosis (WBC, 10^9/L)	11 ~ <13	13 ~ <15	15 ~ <30	≥30
Decreased leukopenia (WBC, 10^9/L)	2.000 ~ 2.499	1.500 ~ 1.999	1.000 ~ 1.499	<1.000
Low hemoglobin (g/dL)				
≥ 13-year-old male	10.0 ~ 10.9	9.0 ~ <10.0	7.0 ~ <9.0	<7.0
≥ 13-year-old female	9.5 ~ 10.4	8.5 ~ <9.5	6.5 ~ <8.5	<6.5
57 days old to < 13 years old	9.5 ~ 10.4	8.5 ~ <9.5	6.5 ~ <8.5	<6.5
Decreased lymphocytopenia (LY, 10^9/L)	0.75 ~ 1.00	0.5 ~ 0.749	0.25 ~ 0.49	<0.25
Decreased neutropenia (ANC, 10^9/L)	0.800 ~ 1.000	0.600 ~ 0.799	0.400 ~ 0.599	<0.400
Decreased platelet (PLT, 10^9/L)				
>12 years old	125 ~ 140	100 ~ 124	25 ~ 99	<25
3 months to 12 years old	NA	50 ~ 75	25 ~ 49	<25

Hyperglycemia (Glu, mmol/L)				
Fasting	6.11 ~ <6.95	6.95 ~ <13.89	13.89 ~ <27.75	≥27.75
Non-fasting	6.44 ~ <8.89	8.89 ~ <13.89	13.89 ~ <27.75	≥27.75
Hypoglycemia (Glu, mmol/L)	3.05 ~ <3.55	2.22 ~ <3.05	1.67 ~ <2.22	<1.67

Table 14. Grading according to urine routine examination

Parameters	Level 1	Level 2	Level 3	Level 4
Urine protein (PRO) (urine test paper detection)	1+	2+	3 + or higher	NA
Urine sugar (urine test paper test)	Trace ~ 1 + or ≤ 250 mg	>2 or >250 ~ ≤500 mg	>2 + or >500 mg	NA
RBC (microscopic examination) [RBC/HPF per high power field (excluding female menstrual period))	6 ~ <10	≥10	Macroscopic hematuria with or without blood clots; or tubular urine red blood cells; or requiring treatment	Emergency or hospitalization

For the clinical abnormalities not involved in the above tables, the intensity of adverse reactions should be graded according to the following standards.

Level 1	Level 2	Level 3	Level 4	Level 5
Mild: short time (< 48h) or slight discomfort, no impact on activity, no need for treatment	Moderate: mild or moderate activity restriction, may require medical treatment, no or only mild treatment	Severe: obviously limited activity, need to see a doctor and receive treatment, may need to be hospitalized	Critical: may be life-threatening, severely restricted, requiring intensive care	death

7.5.3 Relationship between adverse events and experimental vaccine

Definitely not: the adverse events are caused by other factors, and there is sufficient evidence to prove that the adverse reactions/events are caused by other reasons, but not related to vaccination.

Possibly unrelated: the occurrence of adverse events may be caused by other factors, such as the clinical condition of the participant, other treatment or concomitant medication, which is inconsistent with the known adverse reactions of vaccination.

Possibly related: adverse events are consistent with known trial vaccine information, have a reasonable time sequence with vaccination, and/or have occurred for vaccination. There is a causal relationship with the trial vaccine, but it may also be related to other factors.

It is likely to be related: the adverse event is consistent with the known information of the trial vaccine, has a causal relationship with the trial vaccine, and cannot be explained by other factors, such as the clinical situation of the participant, other treatment or concomitant medication.

Definitely relevant: the adverse events are consistent with the known trial vaccine information and have a causal relationship with the trial vaccine, and this relationship cannot be explained by other factors, such as the clinical situation of the participant, other treatment or concomitant medication. In addition, adverse events recur when the trial vaccine is used again.

7.6 Evaluation of immunogenicity

Antibodies (Specific ELISA antibody titers to whole SARS-CoV-2 virus, and the neutralizing antibody amounts against live SARS-CoV-2 virus) are detected in all participants before the first vaccination and after each vaccination. Detailed time points of blood collection are as follows.

Phase 1 trial:

Blood samples are collected from all participants before each vaccination and on the 4th and 14th days (except for the third injection among those aged 3-17 years) after each vaccination and on the 28th, 90th, 180th, and 360th days after the whole-course immunization.

Phase 2 clinical trial:

Blood samples are collected from all participants before each vaccination and on the 28th, 90th, 180th, and 360th days after the whole-course immunization.

7.6.1 Parameters related to immunogenicity

Seroconversion (at least a four-fold increase in post-injection titer from baseline), antibody titers (GMT, GMI), and IgG antibody isotyping.

7.6.2 Evaluation standard

A positive antibody response (seroconversion) is defined as at least a four-fold increase in post-injection

titer from baseline. Since no antibody response was present at baseline, the GMT was imputed using the low limit of detection of the corresponding assay.

7.7 Study hypothesis

For safety, we hypothesize that there is no serious vaccine-related adverse reaction of level 4 or SUSAR, and the number of participants with adverse reactions of level 3 is less than 15% among participants who receive the active vaccines.

7.8 Selection of the best immune procedure and dosage

According to the test results of humoral immunity and safety, the investigator, sponsor, and statisticians jointly make the choice of best immune procedure and dosage.

7.9 Sample size

According to the *Technique Guideline for Clinical Trials of Vaccines*, phase 1 clinical trials are usually carried out in healthy children with normal immune function. If the vaccinated objects are children or other special groups, a phase 1 clinical trial among healthy children should be first carried out, and then inoculations in small-scale target groups could be carried out. Besides, when there are some differences in different dosages, vaccination time, inoculation routes, and diseases, high-dose, medium-dose and low-dose groups (8-10 participants in each group) could be set to observe the clinical tolerance.

In phase 1 clinical trial, 18 participants are respectively enrolled in the low-dose, medium-dose, and high-dose groups, and six participants are enrolled in the placebo control group in each of the dose groups thus there are a total of 18 participants in the placebo group in the cohorts aged 6-12 years and 13-17 years.

24 participants are respectively enrolled in the low-dose, medium-dose, and high-dose groups, and eight participants are enrolled in the placebo control group in each of the dose groups thus there are a total of 24 participants in the placebo group in the cohorts aged 3-5 years.

Phase 2 clinical trial is a randomized, double-blind, placebo-controlled trial, and the main purpose is to explore the optimal dosage and immune procedure and to provide the basis for the establishment of the dosage and immune program used in phase 3 clinical trial. Given that the results of the phase 1 trial is not available, the sample size in the phase 2 trial is mainly determined based on previous research experience of vaccine trials, and the sample size should be about 3-fold of the phase 1 trial.

For the procedure where vaccinations are provided on days 0, 28, and 56, data on immunogenicity in phase 1/2 clinical trials can be merged for the final analyses. Based on the experience of similar clinical trials, the immunogenicity in the high-dose group will not be lower than that in the low-dose group, so single-sided p value below 0.05 is considered as statistically significant. Based on the Two Independent Proportions: inequality (difference) in the PASS software, the power of test is 90% to detect 10% of between-group difference.

For the one-dose high-dose group and the two-dose medium-dose groups, there are 84 participants in each group (estimated loss-to-follow-up rate 10%), and 28 participants in the corresponding placebo group. PASS software is used to calculate sample size. Single-sided p value below 0.05 is considered as statistically significant and the power of test is 80% to detect 15% of between-group difference.

Again, based on results from the preclinical studies, the difference between vaccine group and placebo group is more than 10% or 15% (no antibody responses were observed in the placebo group in the preclinical trials), therefore, the power calculation should be interpreted cautiously.

7.10 Randomization and blinding

7.10.1 Vaccine blinding

The qualified investigational vaccines and placebo are provided by the sponsor. After being blinded by the third party organization, the random sequence is generated using Stata 12.0 software, and the investigational vaccines and placebos are randomly coded (vaccines for each participant have a unique identification number). Considering the need for partial unblinding in case of the emergency of COVID-19 epidemic, blinding is respectively conducted in dose-specific group and immune-procedure-specific group (among those aged 3-17 years in phase 2 clinical trial only). In phase 1 clinical trial, there are 3 groups with different random seeds for each age group; in phase 2 clinical trial, there are respectively 3 groups with different random seeds among those aged 3-17 years. The sample size and corresponding random sequence in each age group are as follows.

Table 15. Random sequence of each group in the phase 1 clinical trial

Group		13 -17 years old (days 0, 28, and 56)	6-12 years old (days 0, 28, and 56)	3-5 years old (days 0, 28, and 56)
Low	Number range	I193-I208	Ⅰ241-Ⅰ256	I193-I208
	Group code	F1,F2	H1,H2	F1,F2
Medium	Number range	I209-I224	Ⅰ257-Ⅰ272	I209-I224
	Group code	F3,F4	H3,H4	F3,F4
High	Number range	I225-I240	Ⅰ273-Ⅰ288	I225-I240
	Group code	F5,F6	H5,H6	F5,F6
Vaccine number		IXXX-1<br>IXXX-2<br>IXXX-3	IXXX-1<br>IXXX-2<br>IXXX-3	IXXX-1<br>IXXX-2<br>IXXX-3

Table 16. Random sequence of each group in the phase 2 clinical trial

Group		13 -17 years old (days 0, 28, and 56)	6-12 years old (days 0, 28, and 56)	3-5 years old (days 0, 28, and 56)
Low	Number range	Ⅱ0929-Ⅱ968	Ⅱ1049-Ⅱ1088	Ⅱ1169-Ⅱ1248
	Group code	F1,F2	H1,H2	M1、M2

Medium	Number range	Ⅱ969- Ⅱ1008	Ⅱ1089- Ⅱ1128	Ⅱ1249- Ⅱ1328
	Group code	F3,F4	H3,H4	M3、M4
High	Number range	Ⅱ1009- Ⅱ1048	Ⅱ1129- Ⅱ1168	Ⅱ1329- Ⅱ1408
	Group code	F5,F6	H5,H6	M5、M6
Vaccine number		IIXXXX-1<br>IIXXXX-2<br>IIXXXX-3	IIXXXX-1<br>IIXXXX-2	IIXXXX-1<br>IIXXXX-2

7.10.2 Vaccine packaging and labeling

Each dose of vaccine used in the trial is packaged in the same shape, and the random sequence is labeled on the packing box. The random sequence is the study identification number. The Roman number I is added in front of the identification number in phase 1 clinical trial, and the Roman number II is added in front of the identification number in phase 2 clinical trial. For vaccines used for each participant, different doses are distinguished by a suffix in sequences, i.e. IXXX-1 represents the first dose for participant XXX in phase 1 clinical trial, IXXX-2 represents the second dose for participant XXX, IXXX-3 represents the third dose for participant XXX.

Vaccine labeling

(1) Each dose of vaccine is packaged separately and labeled with an identification number;

(2) After the vaccine is put into use, the name abbreviation of the participant and the date of vaccination should be filled in the outer packing box, and the activity label should be pasted on the original record sheet. Personnel should check all information before injection. During the trial, all outer packing boxes should be reserved for inspection.

All labels are displayed as follows, and the vaccine No. is identification number for each participant which is randomly generated. Vaccine No. also indicated different doses for each participant, which is identified by a suffix.

Labels used in phase 1 clinical trial

(1) Labels on outer packing box:

A novel coronavirus inactivated vaccine (Vero cell) (for clinical research)
Vaccine No.: I 001-x
Name abbreviation: Date of inoculation: MM/DD/YYYY
Batch No.: XXXXXX Valid until:
[illegible]

(2) Labels on vaccine bottle:

A novel coronavirus inactivated vaccine (Vero cell) (for clinical research)
Vaccine No.: I 001-x

(3) Activity tag:

Vaccine No.: I 001-x
Name abbreviation:

(4) Labels on big box

A novel coronavirus inactivated vaccine (Vero cell) (for clinical research)
Dose X
Vaccine No.: I 001-x
Name abbreviation: Date of inoculation: MM/DD/YYYY
Batch No.: XXXXXX Valid until:

The difference of labels used in phase 1 and II clinical trials is the different length of identification number.

7.10.3 Backup vaccines

Backup vaccines would be used if original vaccines could not be used (for example, vaccines are

damaged, including packages are damaged, or there are sediments or abnormal turbidity even after being shaken). Ten percent of each prepared dose should be prepared for backup. The online electronic system is used to obtain the identification number of backup vaccines. When the field researchers find abnormal vaccines, they should immediately suspend the vaccination and apply backup vaccines through online system. With the approval of the leader of the field, backup vaccines could be used with relevant identification numbers. Meanwhile, the access activity of backup vaccine would be sent to the sponsor, PI, and inspectors. The labels of the backup vaccines and original vaccines should be pasted on the CRFs.

7.10.4 Preservation and use of blinding codes

Statisticians develop a program used for randomization and generate blinding codes. The blinding codes contain two parts. The first part is the group code, and different alphabets represented vaccines or placebos. The second part used different alphabets to identify the low-dose group, medium-dose group, high-dose group, and placebo group. Two copies of the program used for randomization and blinding codes are respectively enclosed, sealed, signed, and kept by investigators and the sponsor. Personnel who participated in generating blinding codes should not participate in clinical trials and should not disclose blinding-related information to personnel participating in clinical trials.

In the phase 1 trial, 3 small envelopes are respectively placed in the large envelope for the first and second part of blinding codes, which contain the programs and parameters used for blinding in 9 subgroups, including I193-I208, I209-I224, I225-I240, I241-I256, I257-I272, I273-I288, I289-I320, I321-I352 and I353-I384. In the phase 2 trial, 3 small envelopes are respectively placed in the large envelope for the first and second part of blinding codes, which contain the programs and parameters used for blinding codes, including II0929 - II0968, II0969 - II1008, II1009 - II1048 , II1049 - II1088 , II1089 - II1128 , II1129 - II1168、II1169 - II1248 , II1249-II1328 , II1329-II1408.When there are safety risks in certain

dose groups from certain age groups, partial unblinding would be needed in the age groups. Besides, partial unblinding is also needed in certain age groups under the urgency of COVID-19 epidemic. Thus, each small envelope is sealed, and when partial unblinding in certain groups is needed, blinding in other groups would not be violated.

7.10.5 Unblinding and reporting

Considering the preclinical animal safety evaluation is still unfinished, current animal evidence only supports two-dose immune procedures in clinical trials. Whether three-dose immune procedures could be used in the clinical trial needs subsequent results of animal studies. If animal experiments are completed and support the three-dose immune procedures before the third dose of vaccine gets injected, the third dose will be injected according to the protocol, and unblinding or partial unblinding in certain age groups could be performed after getting the results of immunogenicity test on day 28 after the third dose and safety evaluation after one month from the third vaccination. If animal experiments are not completed or the completed animal experiments oppose the three-dose immune procedures before the third dose of vaccine gets injected, the third vaccinations will not be carried out, and unblinding or partial unblinding in certain groups could be performed after getting the results of immunogenicity test on day 28 after the second dose and safety evaluation after one month from the second vaccination.

(1) Unblinding

When we get the results of immunogenicity test on day 28 after the whole-course immunization and safety evaluation after one month from the end of the whole-course immunization, unblinding could be conducted after inspection of blinding and data cleaning. After unblinding, evaluation of main safety and immunogenicity test will be conducted. Unblinding is carried out jointly by the sponsor, the main investigator, statisticians, DSMB members, and data managers, etc. Safety follow-up and immune

durability study after 12 months from the end of whole-course immunization would be carried out after unblinding, and relevant safety information will be recorded.

(2) Partial unblinding in certain groups

Considering the urgent COVID-19 epidemic, partial unblinding of certain groups is allowed. Whether to carry out partial unblinding in certain groups and the frequency of partial unblinding in certain groups should be decided according to the time when antibody test results are obtained. All personnel involved in unblinding procedure should sign a confidentiality agreement, and researchers in the field and the participants are still blinded of their group assignment. All procedures specified in the protocol should be continued up to 12 months after the end of the whole-course immunization. Main safety and immunogenicity evaluations could be conducted periodically in the unblinded groups.

When there are safety risks in certain dose groups (such as one case of vaccine-related serious adverse reactions of level 4 or SUSAR, or the number of participants with serious adverse reactions of level 3 exceeds 15% of the vaccinated participants in this group), the clinical trials in these groups should be suspended, and partial unblinding should be carried out. After unblinding, the adverse reactions of level 3 and 4 should be calculated in vaccine groups and placebo group, respectively. Investigators, the sponsor, DSMB, and the ethics committee should jointly decide whether to terminate the clinical trials in the dose groups in advance.

(3) Partial unblinding added to the protocol on June 14, 2020

Considering the urgent COVID-19 epidemic, partial unblinding is added to the protocol on June 14. The data are available for humoral immunogenicity tests on day 14 after the second inoculation in the phase 1 and 2 trials among those aged 3-17 for the following groups: 0/28/56 with low-dose, medium-dose and high-dose in the phase 1/2 trials. Partial unblinding in those groups can be carried out after inspection

and data cleaning. The reasons for unblinding those groups are mainly because results from those groups could provide basic information about safety and immunogenicity of the inactivated vaccine for 3 different doses (100, 200 and 400 WU/dose) and different injection schedules (0/28/56). The information will be used for the decision of continuing the current trial and design for the phase 3 trial.

immunogenicity of the inactivated vaccine for 3 different doses (100, 200 and 400 WU/dose) and different injection schedules (0/28/56). The information will be used for the decision of continuing the current trial and design for the phase 3 trial.

(4) Emergency unblinding

If there are emergencies (such as serious adverse events) in the field, emergency unblinding could be carried out after informing the ethics committee.

An on-line electronic system would be used for emergency unblinding, and a researcher in the field would be authorized. When there are emergencies (such as SAE related to vaccines), information on the dosage in this group should be obtained for timely treatment. The authorized researcher can log in to the personal authorization account on the system and apply for emergency unblinding. Then, the system will send the application of emergency unblinding to the sponsor, principal investigator, and inspector at the same time, and all authorized personnel would log in to the system and handle this application. The online system will inform the researcher in the field of the blinding code of this participant. When adverse events occurred in several participants or there are other situations that might interrupt the trial, the sponsor and investigators could decide to unblind in advance together.

7.11 Trial suspension or early termination criteria

If there are any of the following situations, the trial should be suspended, and the investigator, the sponsor, DSMB, and the ethics committee should jointly hold a meeting to decide whether to terminate the

clinical trial in advance.

- Any adverse reaction with level 4 occurred in any group;
- Any SUSAR related to vaccination occurred in any group;
- In any subgroup, the number of participants with adverse reactions of level 3 exceeds 15% of the total number of participants in that subgroup after each dose;
- The DSMB evaluates the clinical trial and believes there is a potentially great safety risk.

The clinical trial should be terminated in advance if there are any of the following situations.

- The sponsor finds that the vaccine has potential safety hazards or the research has quality problems and requires the trial to be terminated completely.
- The ethics committee calls for the termination of the trial because it is unethical
- The relevant government authorities request the termination of the trial.

7.12 Violation and deviation of protocol

7.12.1 Violation of protocol

The following situations (including but not limited to) indicate that the protocol is violated.

- Informed consent is not appropriately given to the participants;
- The participants do not meet the inclusion criteria, or those meeting exclusion criteria are enrolled in the study;
- The participant receives wrong research interventions (such as wrong vaccinations);
- Serious adverse events (SAEs) are not reported within a given time;
- Vaccines evaluated in other clinical trials are inoculated during the study.

7.12.2 Deviation of protocol

The following situations (including but not limited to) indicate the protocol is deviated.

- The vaccine is not inoculated in the window period;
- Biospecimen samples are not collected in the window period;
- The interval between vaccination and other vaccines is not long enough, except for emergency vaccination such as rabies vaccines.

7.13 Study duration

Early recruitment of participants: 1 month;

Enrollment + vaccination: 6 months;

Long term safety observation and immune durability observation: 12 months;

Database establishment + statistical analysis: 1 month;

Completion of summary report: 1 month

8. Participants

8.1 Inclusion criteria

- Age range: healthy children aged 3-17 years
- General good health as established by medical history and physical examination;
- Since December 2019, the participant has not gone to Hubei province, overseas, or to a village/community where there had COVID-19 cases, have not contacted with confirmed or suspected cases, are not in the quarantine period, and are not from a village/community where there were confirmed or suspected cases.
- Women who have reached menarche are not pregnant (negative urine pregnancy test), are not breastfeeding, do not have pregnancy plan within the three months after enrollment, and have taken effective contraceptive measures two weeks before enrollment;

- Participants are able and willing to complete the whole research procedure in about 14 months;
- Participants have the ability to understand the research procedures, to sign the informed consent voluntarily after explanation, and can comply with the requirements of the clinical research program.

8.2 Exclusion criteria for the first dose

- Confirmed, suspected, or asymptomatic COVID-19 cases;
- Those with positive antibody tests of the COVID-19;
- History of SARS virus infection (identified through self-report or on-site inquiry)
- Those with fever (axillary temperature >37.0 °C), dry cough, fatigue, nasal obstruction, runny nose, sore throat, myalgia, diarrhea, shortness of breath, and dyspnea within 14 days before inoculation;
- Those with clinically abnormal parameters from blood biochemical, blood routine, and urine routine before inoculation (only for phase 1 clinical trial);
- Axillary temperature >37.0 °C before inoculation;
- Those who have experienced severe allergic reactions (such as acute anaphylaxis, urticaria, eczema, dyspnea, neurovascular edema, or abdominal pain), or those who are allergic to the known gradients of COVID-19 inactivated vaccine;
- Those with history or family history of convulsion, epilepsy, encephalopathy, or mental illness;
- Those with congenital malformation, developmental disorder, genetic defect, or severe malnutrition, etc.;
- Those with severe hepatorenal diseases, uncontrolled hypertension (systolic blood pressure ≥140 mmHg, diastolic blood pressure ≥90 mmHg), diabetes complications, malignant tumors, or various acute or chronic diseases (acute attack stage);
- Those diagnosed with congenital or acquired immunodeficiency, HIV infection, lymphoma, leukemia, or other autoimmune diseases;

- Those with confirmed or suspected serious respiratory diseases, serious cardiovascular diseases, hepatorenal diseases, and malignant tumors;
- Those with a history of abnormal coagulation (such as lack of coagulation factors or coagulation diseases);
- Those receiving anti-TB treatment;
- Those receiving immunoenhancement or inhibitor treatment (p.o. or gtt.) within 3 months (continuous oral or infusion for more than 14 days);
- Those receiving live attenuated vaccines within one month before inoculation or other vaccines within 14 days before inoculation;
- Those receiving blood products within 3 months before inoculation;
- Those receiving other study drugs within 6 months before inoculation;
- Those under other conditions not suitable for the clinical trial (evaluated by researchers).

8.3 Exclusion criteria for second and third doses

- Women who have reached menarche with positive urine pregnancy tests;
- Those with high fever (axillary temperature ≥39.0 °C) lasting for three days or severe allergic reaction after the previous inoculation;
- Serious adverse reactions related to the previous inoculation;
- If inconformity with inclusion criteria or conformity with exclusion criteria for the first dose occurs or is newly found after the previous inoculation, researchers should decide whether the participants could continue to participate in the study;
- Other reasons for exclusion evaluated by researchers.

Participants are not required to stop the trial if

- non-specific immunoglobulins are used during the study;

— steroid hormones have been given orally or intravenously for 14 days.

8.4 Early withdrawal of the participants

Early withdrawal refers to the process that the participants fail to complete the vaccination and blood collection procedures according to the protocol, and the researchers decide whether to continue the subsequent procedures. Early withdrawal is needed if any of the following issues occur.

— The participant or the guardian of the participant requests to withdraw from the clinical trial;

— There are intolerable adverse events, no matter if they are related to the experimental vaccine or placebo;

— It is inappropriate for the participant to continue the trial due to his/her health condition;

— Participants are vaccinated with other experimental vaccines during the study period;

— Any other reasons decided by researchers.

9. Procedures and methods of the trial

9.1 Recruitment

After the review and approval of the ethics committee, the investigators and researchers will work with the local medical staff to publicize the recruitment of this clinical trial to the qualified volunteers, and will recruit and register the candidates who voluntarily participate in the trial. Recruitment progress should be adjusted according to the progress of the trial during the study, to ensure the numbers of male and female participants are relatively balanced.

9.2 Informed consent

Informed consent refers to the process that the participants voluntarily confirm their participation in the clinical trial after being informed of all aspects of the trial, which must be evidenced by the signed and dated informed consent. After the volunteers arrive at the field, informed consent must be given to them

first. Researchers informed the volunteers of the informed consent of the clinical trial in oral and written form. Volunteers or the entrusted person and the researchers should jointly sign the informed consent voluntarily. The informed consent should be in duplicate, and the volunteer or the entrusted person should keep a copy, and the researchers keep the other copy.

9.3 Inclusion/exclusion criteria of physical examination and examination

For volunteers who sign the informed consent, height, weight, body temperature, and blood pressure will be measured. Auscultation of heart and lung, skin examination, and observation of body development would also be performed. Women who have reached menarche should receive urine pregnancy tests before each vaccination.

For participants in phase 1 clinical trial, around 16 mL of fasting blood samples would be collected for blood routine examination, blood biochemistry test, COVID-19 antibody test, and humoral and cellular immunity test. Urine samples are collected for routine urine tests. The identification numbers of samples are identification number used for screening.

For participants in the phase 2 trial, two blood drops are collected from their fingertips for antibody tests.

Researchers inquire about volunteers' information according to inclusion and exclusion criteria in this protocol and decide whether the volunteers could be included in the clinical trial. Information on physical examination and inclusion/exclusion criteria of participants would be inputted into EDC system.

9.4 Study number allocation

Each eligible participant is assigned a unique study number in order of time. Once the study number is assigned, it would not be reassigned to other participants.

9.5 Blood sample collection before immunization (phase 2 clinical trial)

After all the participants are enrolled in the group, about 4 mL of venous blood samples are collected before the first vaccination, and serum is separated for antibody measurements. Within 24 hours, serum is separated and packed into three tubes (tube A and tube B for laboratory measurement, each tube contains no less than 0.5 mL of serum, and tube C for storage), and stored at - 20 °C or below for antibody measurement.

One block (eight participants) is selected from three-dose middle-dose groups (A3 and A4) among those aged 3-17 years. After enrollment, 10 mL of blood samples (added anticoagulant) are collected, and PBMC is isolated for high-throughput single cell sequencing.

9.6 Vaccine inoculation

9.6.1 Requirements of inoculation

- Before inoculation, the information of participants and experimental vaccines must be checked. After obtaining the identification number of vaccines from outer packing box, outer packing box could be opened. After the verification between the label on the outer packing box and the label on the bottle, the name abbreviation and vaccination date of the recipients should be filled in the label on the outer packing box, and the activity tag should be torn off and pasted on the corresponding position of the original data record sheet.
- The vaccine should be injected at deltoid muscle of upper arm.
- During the inoculation, vaccines and placebo should be kept at 2-8 °C (the temperature should be monitored and recorded every hour). The time between taking out vaccines from thermal insulation containers and completing inoculation should not be over 30 minutes.
- During the inoculation, if the vaccine is found abnormal, such as abnormal color, damage, and insufficient loading, etc., the inoculation should be stopped immediately, and the abnormalities should

be reported to the sponsor, inspectors, and the researcher who is in charge in the field. After confirming the damage of vaccines, the original vaccine should be abandoned according to the protocol, and backup vaccine should be used for inoculation.

- Input inoculation information into EDC system.

9.6.2 Vaccination window period

The window period of each dose is + 7 days. If the vaccination is postponed within the window period, the date of the next dose should be postponed accordingly.

9.7 Clinical observation after inoculation in the field

Adverse reactions should be observed for 30 minutes after each inoculation. Researchers should explain to the participants the methods of how to judge, measure, record, and report adverse reactions in the field. Diary card, scale, and thermometer should be given to the participants. Researchers should also teach the participants, guardians or trustees how to use the thermometer, observe adverse events, fill in the diary card, and make an appointment to return the diary card (return the diary card and get the contact card on the 8th day after vaccination).

9.8 Safety observation and follow-up

Phase 1 trial

(1) Topical and systemic adverse events are collected during 30 minutes after each inoculation;

(2) Researchers visit the participants once a day in the village or community within 0-7 days after the first inoculation to guide the participants to report the adverse events correctly and fill in the diary card;

- ✧ The researchers and the doctors should make use of the village/community health service center to actively monitor the adverse events of the participants, and instruct the participants to accurately report and record the topical and systemic adverse events.

✧ If there are respiratory tract infection symptoms such as fever and cough, researchers should make a comprehensive judgment, carefully identify the common symptoms after vaccination or the infection caused by other reasons, and closely observe the progress of the disease. If the disease gets worse, the doctors should report it to the lead researcher in the field as soon as possible. The participant should be taken care of immediately to ensure that the participant get timely diagnosis and treatment.

(3) After the second and third inoculation, a phone call should be made in 6 to 24 hours. A face-to-face visit should be made within 1 to 3 days to instruct the participants to report and record adverse events on the diary cards. Another phone call should be made within 4 to 7 days;

(4) On the 8th day after each inoculation, the researchers should collect and review diary cards of this period, and provide the participants with new contact cards;

(5) On the 8th to the 28th/30th days after each inoculation, the safety observation is carried out by weekly telephone follow-up and active reports from the participants. Contact cards should be collected on the 28th/30th day;

(6) When knowing that a participant has adverse reactions/events of level three or above, a face-to-face visit should be conducted within 24 hours;

(7) SAE should be observed by monthly telephone follow-up and active reports from the participants between the 31st day to the 12th month after the whole-course vaccination.

Phase 2 trial

(1) Topical and systemic adverse events are collected during 30 minutes after each inoculation;

(2) After each inoculation, a phone call should be made in 6 to 24 hours to inquire about adverse events;

(3) A face-to-face visit should be made within 1 to 3 days to instruct the participants to accurately report

and record the adverse events and fill in the diary cards;

(4) Another phone call should be made within 4 to 7 days;

(5) On the 8th day after each inoculation, the researchers should collect and review diary cards of this period, and provide the participants with new contact cards;

(6) On the 8th to the 14th/21st/28th/30th days after each inoculation, the safety observation is carried out by weekly telephone follow-up and active reports from the participants. Contact cards should be collected on the 14th/21st/28th/30th day;

(7) When knowing that a participant has adverse reactions/events of level three or above, a face-to-face visit should be conducted within 24 hours;

(8) SAE should be observed by monthly telephone follow-up and active reports from the participants between the 31st day to the 12th month after the whole-course vaccination.

9.9 The second and third vaccinations

Diary cards should be collected on the 8th day after the first inoculation, and the contact cards should be collected on days 28/30 after inoculation to review adverse events.

Before the second and third inoculation, exclusion criteria should be checked. Those meeting the inclusion criteria but not exclusion criteria should be inoculated, and the window period of each inoculation is +7 days.

9.10 Sample collection

Table 17. Schedule of blood collection in the phase 1 trial

No.	Blood collection time	Blood volume for biochemical blood and routine blood test (mL)	Blood volume for humoral immune antibody test (mL)	Blood volume for cellular immune safety outcome test (mL)	Total blood volume (mL)

1	Before 1st dose	5	4	7	16
2	4 days after 1st dose	5	4		9
3	14 days after 1st dose		4	7	11
4	Before 2nd dose	5	4	7	16
5	4 days after 2nd dose	5	4		9
6	Before 3rd dose	5	4	7	16
7	4 days after 3rd dose	5	4		9
8	14 days after 3rd dose		4		4
9	28 days after 3rd dose		4	7	11
10	90 days after 3rd dose		4		4
11	180 days after 3rd dose		4	7	11
12	360 days after 3rd dose		4	7	11

Table 18. Schedule of blood collection in the phase 2 trial

No.	Blood collection time	Program group			Blood volume for antibody test (mL)
		1-dose	2-dose	3-dose	
1	Before 1st dose	●	●	●	4
2	Before 2nd dose (28 days after 1st dose)	●	●	●	4
3	Before 3rd dose (28 days after 2nd dose)	●	●	●	4
4	28 days after 3rd dose	●	●	●	4
5	90 days after vaccination	●	●	●	4
6	180 days after vaccination	●	●	●	4
7	360 days after vaccination	●	●	●	4

9.11 Concomitant drug use

Within days 0-14/21/28/30, the adverse events of the participants are actively observed and recorded on diary/contact cards. Meanwhile, the drug uses of the participants within days 0-14/21/28/30 are recorded on the diary/contact cards.

Allowed drug use: During the clinical trial, if the participants have adverse events, necessary drug treatment and medical treatment should be allowed;

Allowed vaccine: Other vaccines are allowed to be inoculated during the trial; however, they should be inoculated 7 days after the trial vaccine is inoculated. Emergency vaccinations, such as rabies vaccines, are

unrestricted.

Drug use record: In order to understand the impact of drug use on vaccine safety and immunogenicity during the trial and to completely collect vaccine-related adverse events, researchers should instruct the participants to record all hospital visits and drug use in diary cards and/or contact cards and assist in collecting information on inpatient drug use records. When a SAE occurs, copies of corresponding medical records and medication records should be collected and kept. The following drugs need to be transcribed into the electronic case report form (ECRF):

(1) Hormone/steroid drugs and other immunosuppressants;

(2) Antiallergic drugs;

(3) Antipyretics/analgesics/NSAIDs;

(4) Preventive vaccine;

(5) Therapeutic biological products;

(6) Antibiotics;

(7) Antiviral;

(8) Chinese traditional medicine;

(9) Other medications determined by investigators.

9.12 Closure of the field

The field could be closed when (1) all participants complete the long-term safety observation 12 months after the last inoculation and the blood sample collection used for immune durability test 12 months after the whole-course immunization, (2) when all original data are collated, inputted, checked, and then submitted to the statisticians, and (3) when all questions about data confirmation are replied and solved.

9.13 Criteria for ending the clinical trial

(1) The blood samples have been sent to CFDA or Wuhan Institute of Virology of Chinese Academy of Sciences after each blood collection, and the test reports have been completed;

(2) All participants complete the required visits, and the original data and documents of the clinical trial are transferred to the data manager for filing and storage;

(3) The number of remaining vaccines is accurate, and the remaining vaccines are handed over to the sponsor;

(4) The statistical analysis report and summary report meet the requirements.

10. Data management and statistical analysis plan

10.1 Data management

10.1.1 Data record

The original records in clinical research include electronic records and paper records. The receptions, physical examinations, sample collections, sequence allocation, vaccinations, and 30-minute medical observations are electronic records, which could be generated in real time. Informed consent, diary cards, contact cards, SAEs, protocol violations/deviations, and emergency unblinding are paper records, which should be inputted into the vaccine clinical research system (EDC system) after being checked.

When researchers enter data, they should correct the wrong data and record the process. The process of the inspector's and data managers' questioning data and the researchers' responding to the questions should be completely recorded. Corrections should leave notations, which facilitate further rechecks. Researchers should keep the original records carefully.

10.1.2 Supervision of data records

Investigators, data administrators, and inspectors should regularly and irregularly monitor data records until all data are entered, verified, and cleaned. Data administrators are mainly responsible for checking

whether the data in the electronic case report form (eCRF) and the data in the original record are consistent, besides, they also should check if there are logical mistakes.

Any question found by data administrators and/or inspectors during the verification should be fed back to researchers through EDC system. Researchers should make verifications and reply to the queries, and data administrators and/or inspector should review the replies and end queries after confirmation. If queries are not resolved, new queries should be given out again until all questions are solved.

10.1.3 Data quality control

After the completion of all data entry, verification, and data cleaning, a certain number of cases should be randomly selected according to a certain proportion for quality control of the database, and the data in the electronic case report form (eCRF) and EDC system should be checked with the original records to ensure the consistency of the data.

10.1.4 Data locking

Before the clinical trial database can be locked, blind data review should be carried out to confirm all data queries, cases who lose to follow-up and violate or deviate protocol, concomitant drug use, adverse events, and the division of analysis data set.

After all data have been correctly input into the database, all data queries have been confirmed and solved, and all eCRFs have been signed by the principal investigator, the sponsor, researchers, statisticians, data administrators, and inspectors, etc. should confirm and approve the locking of the database. After the database is locked, the data editing authority of the database should be revoked.

10.2 Contents of statistical analysis

10.2.1 Statistical analysis plan

(1) Proposal

The personnel who undertake the task of statistical analysis participate in the whole process from experiment design, implementation, formulation of statistical analysis plan, to data summary. They should maintain the database and carry out statistical analysis, as well as write statistical analysis plan and complete clinical statistical report.

(2) Selection of datasets for analysis before unblinding (for safety analysis and immunogenicity analysis on day 28 after whole-course immunization)

Dataset for safety evaluation (SS): All participants who have received vaccines and have at least one data point of safety evaluation should be included in the dataset for safety evaluation. Participants who violate the protocol should not be excluded.

Dataset for full analysis (FAS): The dataset for full analysis is set according to the theory of intention-to-treat (ITT) analysis and includes an ideal population. All participants who have met the inclusion criteria but not the exclusion criteria, participated in randomization, received experimental vaccines (including the placebo), and had results of any blood biomarker measurements before immunization or between first inoculation and day 28 after the whole-course immunization should be included in the FAS for immunogenicity analysis.

Per-protocol set (PPS): PPS is a subset of FAS. The participants in this dataset are more compliant with the protocol, do not take any banned drugs during the study period, do not severely violate the protocol and inclusion criteria, are vaccinated according to the protocol, and have results of any biomarker measurements before immunization or between first inoculation and day 28 after the whole-course immunization.

① Phase 1 clinical trial

For the subset of PPS on the 4th,14th after the first inoculation as well as before the second inoculation,

participants should be included in PPS if they have finished the first inoculation, do not take banned drugs between the first two vaccinations, do not greatly violate the protocol, and meet the inclusion criteria.

For the subset of PPS on the 4th and 14th days after the second inoculation as well as before the third inoculation (or on day 28 after the second inoculation for the two-dose group), participants should be included in PPS if they have finished the second inoculation, do not take banned drugs between the first two inoculations, do not greatly violate the protocol, and meet the inclusion criteria.

For the subset of PPS on the 4th and 28th days after the third inoculation, participants should be included in PPS if they have finished the third inoculation, do not take banned drugs between the first two vaccinations, do not greatly violate the protocol, and meet the inclusion criteria.

② Phase 2 clinical trial

The principle of PPS is similar to that of phase 1 clinical trial, except that the 4th and 14th days after each inoculation are exempted.

The following participants are excluded from the PPS: those not meeting the inclusion criteria; those meeting the exclusion criteria; those with no or severely missing follow-up data or information after vaccination; those meeting the termination or withdrawal criteria but remaining in the trial; those receiving wrong vaccination or dose; those who are judged by the researchers to be excluded.

(3) **Selection of analysis data set after unblinding (for immune durability)**

Dataset for full analysis (FAS): All participants who meet the inclusion criteria but not the exclusion criteria, participate in randomization, receive experimental vaccines, and have results of any blood biomarker measurements on days 90, 180, and 360 after the whole-course immunization.

Per-protocol set (PPS): PPS is a subset of FAS. The participants in this dataset are more compliant with the protocol, do not take any banned drugs during the study period, do not severely violate the protocol and

inclusion criteria, are vaccinated according to the protocol, and have results of any biomarker measurements before immunization or on days 90, 180, and 360 after the whole-course immunization.

The following participants are excluded from the PPS: those not meeting the inclusion criteria; those meeting the exclusion criteria; those with no or severely missing follow-up data or information after vaccination; those meeting the termination or withdrawal criteria but remaining in the trial; those receiving wrong vaccination or dose; those who are judged by the researchers to be excluded.

PPS is used as the main dataset when analyzing humoral and cellular immune parameters. However, FAS is also used for analysis, and any different results from PPS and FAS would be discussed in the report.

10.2.2 Statistical analysis content

(1) Trial completion assessment

- The numbers and reasons of people who participate in the trial, complete the trial, drop out of trial, and violate/deviate the protocol;
- Demographic characteristics: sex composition and mean age.

(2) Safety evaluations

Safety evaluation mainly includes the total incidence of adverse reactions and the incidence of topical and systemic adverse reactions after each inoculation.

Adverse event (0-7 and 0-28/30 days) in the vaccine group and placebo group:

- The total incidence of adverse events/reactions;
- Adverse reactions after each inoculation;
- The incidence of topical or systemic adverse reactions;
- The incidence of abnormal laboratory parameters;

- Lymphocyte subsets and cytokine measures (including IL-1β, IL-2, IL-4, IL-5, IL-6, IL-8, IL-10, IL-12p70, IL-17A, IL-17F, IL-21, IFN -γ, TFN -α, TFN -β) before each injection, on day 14 after the first and second injection, before the third injection, and on days 28, 128, and 360 after the third injection.
- The composition of adverse reactions of different levels.

Adverse reaction symptoms in the vaccine group and placebo group:

- The incidence of symptom-specific adverse reactions and composition of adverse reactions of different levels;

Events causing withdrawal and SAE:

- Withdrawal due to adverse events and/or serious SAE cases should be specially noted, and the incidence of these events should be compared between groups.
- For non-solicited adverse events and SAE, the SOC level and PT level are analyzed after coding with MedDRA.

(3) Analysis of humoral immune tests

The incidence of four-fold increase of antibody as well as GMT, GMI and 95% CI of antibody in each age group and dose group are described.

Seroconversion (four-fold increase of antibody) = the number of participants with 4-fold increase of antibody after vaccination/total participants× 100%.

10.2.3 Statistical analysis method

(1) Baseline demographic characteristics

The χ^2 test is used to compare the sex composition in the vaccine and placebo groups. Two independent samples t-test is used to compare the mean age in each age-specific intervention group and control group.

(2) Participant compliance

The proportions of participation, loss to follow-up (and the reasons), exclusion, blood sample collection, use of concomitant drugs, and use of other vaccines in vaccine and placebo groups are described. χ^2 test, modified χ^2 test, or Fisher's exact test is used to compare the proportion of loss to follow-up and use of concomitant drugs between groups.

(3) Safety evaluation

Safety evaluation is based on SS dataset. Parameters used for safety evaluation in immune-procedure-specific groups are analyzed in phase 1 and 2 trials separately, which are further merged for analysis.

Adverse events in vaccine and placebo groups are counted, and the incidence rates (95% confidence interval) are calculated. The proportion of different levels of adverse events is also described. The χ^2 test, modified χ^2 test, or Fisher's exact test is used to compare the incidence of adverse events, adverse events of level 3, and SAE between vaccine and placebo groups. The χ^2 test or Fisher's exact test is used to compare the incidence of adverse events, adverse events of level 3, and SAE across the low-dose, medium-dose, high-dose and placebo groups. Rank sum test is used to compare the mean severity level of the adverse events between vaccine and placebo groups.

(4) Cellular immune safety measures in the phase 1 trial

FAS and PPS datasets are used for analysis.

Analysis of cytokine: ① Two independent samples t-test (when meeting normal distribution and homogeneity of variance) or modified t-tests (when meeting normal distribution but violating homogeneity of variance) or ANOVA is used to compare the differences of mean levels of cytokines, including IL-2, IL-4, IL-6, IL-10, TNF, and IFN, etc., before the first inoculation and each time point after the first inoculation among different groups. ② Analysis of variance of repeated measurement data is used to explore the difference of mean levels of cytokines in three dose groups on each time point after inoculation.

Analysis of lymphocyte count: ① Two independent samples t-test (when meeting normal distribution and homogeneity of variance) or modified t-tests (when meeting normal distribution but violating homogeneity of variance) is used to compare the differences of lymphocyte count among vaccine and placebo groups as well as different dose groups before the first inoculation and on different time points after inoculation. ② Analysis of variance of repeated measurement data is used to explore the differences of lymphocyte count in three dose groups on each time point after inoculation.

(5) Humoral immunogenicity assessment

FAS and PPS datasets are respectively used for analyses. For groups vaccinated on days 0, 28, and 56 in phase 1 and 2 clinical trials, blood samples are collected before each inoculation and on day 28 after whole-course immunization, thus we could merge the antibody measures of the same time point in phase 1 and 2 clinical trials. Blood samples are also collected on days 4 and 14 after each inoculation and on day 21 after the first inoculation in the phase 1 trial, thus the results are also reported for these time points.

Statistical description: The antibody titer should be logarithmically transformed, and the minimum, maximum, median, interquartile range, and GMT (95% confidence interval) would be calculated.

Comparison of serum antibody GMT before inoculation: Antibody titer is logarithmically transformed, and two independent samples t-tests (when meeting normal distribution and homogeneity of variance) or modified t-tests (when meeting normal distribution but violating homogeneity of variance) are used to compare serum antibody GMT before vaccination between vaccine and placebo groups, and ANOVA was used to compare the differences between different dose groups. The χ^2 test, modified χ^2 test, or Fisher's exact test is used to compare the positive rate of antibodies among vaccine and placebo groups, as well as different dose groups.

Comparison of antibodies at each time point after inoculation: ① antibody titer is logarithmically

transformed. For those receiving inoculation on days 0, 28, and 56, two independent samples t-test (when meeting normal distribution and homogeneity of variance) or modified t-tests (when meeting normal distribution but violating homogeneity of variance) are used to compare the antibody GMT after each vaccination between vaccine and placebo groups, and ANOVA was used to compare the differences across different dose groups. ② χ^2 test, modified χ^2 test, or Fisher's exact test is used to compare the seroconversion rate among vaccine and placebo groups, as well as different dose groups. ③ The antibody GMI and its 95% confidence interval are calculated in each group. ④ Analysis of variance of repeated measurement data is used to examine the differences of antibody GMT at different time points in different dose groups.

If necessary, the above statistical analysis and statistical report can be divided into two parts: (1) after the partial unblinding, data on safety evaluation and immunogenicity could be analyzed, and the results of the analysis could be used to write the first part of statistical report, which could provide evidence for setting dosage and immune procedure for the phase 3 clinical trial. (2) After the results of antibody tests on days 90, 180, and 360 are obtained, the second part of statistical report (immune durability analysis) could be finished.

11. Monitoring of clinical trials

11.1 Sponsor

The sponsor should establish a vaccine clinical trial quality management system to monitor, check, and control the whole process of the trial. The sponsor should also ensure the trial would be carried out as required, and data, records, and reports should meet the requirements of GCP and other regulations as well as the protocol. Before the registration of participants, the sponsor should ensure that investigators and

inspectors have known all clinical protocol and procedures related to the trial, including the information about the vaccines for the trial, the procedure for obtaining informed consent, the procedure for reporting adverse reactions/events, and the procedure for completing the eCRF, etc.

11.2 Investigators and researchers

The investigators in charge of vaccine clinical trials should establish a complete vaccine clinical trial organization management system and quality management system, have the management mechanism and measures to prevent and deal with emergencies in vaccine clinical trials, have the emergency treatment expert team for SAEs who are able to deal with serious adverse events, and have perfect cold chain equipment for vaccine delivery and storage to ensure the safe storage and delivery of vaccines and samples.

The clinical sites of vaccine clinical trial should have the vaccination qualification approved by the health administration department. They should also have relatively fixed and sufficient clinical trial researchers and standard operating procedures related to vaccine clinical trial. Related training should be carried out and recorded. Also, special VIP channels should be established with the aid of local hospitals to handle SAEs during the clinical trial. According to different vaccination and visit procedures, the trial site should set reception area, informed consent room, physical examination and consultation room, biological specimen collection room, vaccination room, emergency room, medical observation room, vaccine storage room, archive room, sample processing and preservation room, case screening laboratory, and place for temporary storage of medical waste, etc. Special VIP emergency channel should be established, and ambulance, rescue personnel, and first-aid items should be equipped with on site.

The tasks of all researchers should be confirmed by the main investigators to ensure that all researchers involved in the project are qualified. After training, each researcher should know their work task and master and conduct relevant standard operating procedures. Researchers in the field should be trained in capacities

related to GCP and vaccine clinical trial, and the training should be recorded. Auxiliary personnel should also participate in training, and the training should be recorded.

11.3 DSMB

DSMB which is independent of the sponsor could provide professional consultation and review clinical trial according to the protocol in order to protect the rights and interests of the participants. DSMB should also evaluate the safety of the vaccine and monitor adverse events during the trial. When there are significant safety risks for the participants, or the possibility of completion of the trial is reduced, DSMB should promptly notify the sponsor and hold a meeting with the sponsor and investigators to decide whether to suspend or terminate the clinical trial in advance.

The specific operations are as follows.

- Data monitoring and safety assessment of clinical trials at all stages should be performed by DSMB. The report evaluating the vaccines' safety on days 0 to 7 after the first vaccination and on day 28/30 after the whole-course vaccination in each age group in phase 1 clinical trial should be submitted to DSMB for review.
- After the safety observation on days 0 to 7 after the first dose in each dose group in phase 1 clinical trial is completed, the sponsor should send the safety assessment report to DSMB to facilitate DSMB to track the safety in phase 1 clinical trial. If DSMB does not send out questions on safety within 24 hours after receiving the report, the sponsor could acquiesce in that the safety assessment result in this stage meet the requirements of the protocol and could carry out the trial in the next group.
- During the next phase of the study, if there is a safety problem in the subsequent vaccination in the previous phase, DSMB would evaluate it in time and give suggestions (whether to continue the

trial after modifying the protocol or to terminate or suspend the trial in one or all groups) to the sponsor.

- In each phase of the trial, i.e. at any time during phase 1 clinical trial, the sponsor and investigators could propose questions related to safety or other aspects and hold an unplanned meeting with the DSMB. DSMB should timely evaluate the problem and give suggestions (whether to continue the trial after modifying the protocol or to terminate or suspend the trial in one or all groups) to the sponsor.

11.4 Personnel training

Before the trial, the sponsors and main investigators/researchers should train personnel involved in the trial. The training contents should include the brief scheme of the clinical trial, trial implementation procedure, time arrangement, operation precautions, test data filling, etc. During the trial, if there are new researchers or personnel, they should be trained separately. Additional training could be conducted if necessary. Each training should be recorded.

11.5 Inspectors

According to the *Technique Guideline for Clinical Trials of Vaccines*, the sponsor should appoint a sufficient number of inspectors to conduct whole-process inspection of clinical trials. The inspectors should have the education background and working experience of medicine, pharmacy, or related majors. The number of inspectors designated by the sponsor should be determined according to the frequency of the supervision and the complexity of the trial design. Inspectors should carry out the inspection of the clinical trial according to the requirements of the inspection plan and submit the inspection report.

11.6 Safety of the participants

11.6.1 General precautions

The clinical trial is carried out in the Wuzhi County Center for Disease Control and Prevention. The sponsor has examined the research site strictly in accordance with the requirements of GCP before the trial, focusing on whether the environmental facilities meet the requirements of the *Management Specifications for Vaccination* and *Technique Guideline for Clinical Trials of Vaccines*. The facilities and equipment in the first-aid room should be sufficient and effective, and the first-aid doctors are qualified. *Special VIP channel agreement* should be signed by the research field and local hospitals. The first-aid personnel (including doctors, nurses, drivers, etc.) should be trained and familiar with the transfer route and procedures of the hospital. They should be on standby in the research field during vaccinations. An ambulance should be equipped with and parked in a fixed position. The ambulance should be in good condition and an emergency state, and it should be prepared to respond to the emergency teams at any time. During vaccinations, the hospital should prepare for medical personnel, instruments and equipment, and first-aid drugs every day to ensure that the participants can receive timely treatment. The emergency plan should be formulated in the research field, which specified personnel responsibility, contact number, rescue route, etc. to ensure that adverse events could be handled timely and effective contact between the participants and researchers to timely address serious adverse events.

11.6.2 Risk prevention measures related to COVID-19

(1) Considerations for the clinical trial site

During the epidemics of COVID-19 in Wuzhi County, 5 confirmed cases were all imported from Wuhan. Most communities/villages had no confirmed cases and are considered as low-risk areas. The clinical trial plans to recruit participants from communities or villages without confirmed COVID-19 cases (verified through infectious disease surveillance system). Recruited participants do not have close contact with individuals from Wuhan or confirmed cases, and they do not travel outside the county before the trial.

Researchers inquire volunteers about whether they have been to Hubei province, overseas, or villages/communities with confirmed cases since December 2019, whether they had close contact with confirmed or suspected COVID-19 cases, whether they are in quarantine, and whether there were COVID-19 confirmed cases or suspected cases in their village/community through investigations or inquiries. Symptoms of fever (axillary temperature >37.0 °C), dry cough, fatigue, nasal obstruction, runny nose, sore throat, myalgia, diarrhea, shortness of breath, and dyspnea are also inquired.

(2) Strengthening the management and personal protection of participants during the trial

The research field should be disinfected strictly according to the regulations, and the windows should be opened regularly for ventilation. Independent areas and special channels are strictly set and applied, and the volunteers and those accompanying them should wait in different areas to avoid contact with personnel other than research doctors and nurses. Each functional area and public site should be equipped with hand disinfectants and temperature measuring devices, and the volunteers and those accompanying them should wear masks, get hand disinfected, and measure temperature before entering the research facilities.

During the first inoculation, researchers should remind the participants to strengthen self-protection. Necessary personal protective equipment such as masks and disinfectants should be provided to the participants. Besides, researchers should closely monitor the health status of the participants, especially for symptoms related to COVID-19.

(3) Novel coronavirus IgM/IgG antibody tests

The SARS-CoV-2 IgM/IgG antibody would be tested before the first inoculation. If the antibody test results are positive, the volunteers would not be eligible to participate in the clinical trial. Besides, doctors would ask the volunteers whether they have recent symptoms such as fever, dry cough, or fatigue, etc. If the volunteers have these symptoms, the participants should be arranged to the hospital and to receive chest

imaging tests according to the requirements of prevention of COVID-19. Samples would be collected for etiology detection on SARS-CoV-2 if chest imaging indicates that there is a possibility of SARS-CoV-2 infection.

(4) In phase 1 trial, sentinel monitoring is used to fully protect the safety of the participants

In the phase 1 trial, researchers will stay in the village health center or community health service center in the research field on days 0 to 7 after the first inoculation. The participants will be actively followed (daily face-to-face visits).

(5) COVID-19 disease process monitoring

SARS-CoV-2 or other coronavirus infection will be monitored among the participants if they are confirmed cases to find out potential populations who might be at risk of getting ADE or VED after receiving the vaccine. Timely treatment and intervention measures should be taken to reduce the risk of disease progression.

The research field should sign medical treatment agreements with local second-class hospitals or above to set up the special VIP channels, in order to ensure that the participants could get timely treatment after being infected with SARS-CoV-2. Researchers should inform the hospital that the participant has received an inactivated vaccine for SARS-CoV-2 to assist doctors to learn about the participants' condition and potential risks. Hospitals would collect nasopharynx swab, alveolar fluid, and blood samples for novel coronavirus etiological measurements. Blood samples are also used for antigen tests. Lead researchers and hospitals should closely observe patients' disease progression. They should also contact other hospitals or institutions who could test IL-2, IL-6, IL-4, TNF-α, and IFN-γ, etc. and prepare for the measures. When the patients' disease progresses rapidly and has the tendency of ADE or VED, blood samples should be collected and sent to the hospitals or institutions that can carry out the measures to figure out whether there is ADE or

VED. Zhengzhou Jinyu Clinical Laboratory Center Co., Ltd. is selected for the measurements of those cytokines.

After unblinding, if the participants from intervention groups get diseases related to SARS-CoV-2 infection and the disease progression is quick, it indicates that there is a higher possibility of ADE or VED. Doctors should be suggested to adopt immunosuppressive measures to avoid the formation of inflammatory storm. The participants could also be transferred to a higher-class hospital in time to prevent more serious injuries due to improper treatment of ADE or VED.

(6) Antibody durability monitoring and long-term safety follow-up

According to the protocol, the participants should be followed up for one year. Information on serious adverse events including disease caused by SARS-CoV-2 infection should be collected through phone follow-up and active report from the participants. Meanwhile, blood samples should be collected in the third, sixth, and 12th months after whole-course immunization for immune durability test. Results could be used to decide whether to prolong the observation of immune persistence and long-term safety.

11.6.3 Handling and reporting serious adverse events

The monitoring and report of adverse events during clinical trials are jointly conducted by the participants, researchers in the field, investigators, sponsor, and other relevant organizations at different stages.

The sponsor is the main body responsible for the safety information monitoring and evaluation as well as reporting SAE in clinical trials. Staff would be designated to monitor the safety of vaccines and report SAE during the clinical trial, and they should work with researchers to formulate the standard operating procedures for clinical trial safety information monitoring and SAE report. They should learn about the latest status of safety information of the whole clinical trial and timely report it to all clinical trial

institutions/researchers and regulatory authorities.

The sponsor should not change the investigator's judgment on the correlation between serious adverse events and vaccines. The sponsor should report the serious adverse events that may be related to or are related to the investigational vaccine to the chairman of DSMB by telephone or fax within 48 hours, and the sponsor should also submit the follow-up information as soon as possible. If the sponsor, investigators, and DSMB could not reach in an agreement, their opinions should be stated in detail in the report according to the regulation. DSMB's review of serious adverse events should not exceed the deadline of reporting to regulatory authorities, if required.

When it is difficult to determine the correlation between SAEs and vaccines, or the reported correlations between SAEs and vaccines are questioned and need re-evaluation, a panel of experts in the relevant fields could be invited to discuss these issues and to reach a final conclusion.

(1) Measures in the field

Emergency plan for SAE treatment should be established in the research field, and all relevant personnel should be trained. If there are serious adverse events, researchers should take appropriate measures and record them. Researchers should try to learn about any clinically significant diseases or events after vaccinations among the participants and make sure that the participants receive appropriate treatment in the designated hospital in time according to the relevant regulations.

Serious adverse events/reactions should be followed up until symptoms disappear or remain stable. The progressions and outcomes of all symptoms should be recorded in detail, and all drug treatment and medical treatment should be recorded during follow-up. Serious adverse events should be accurately recorded on eCRF and evaluated and discussed in the final report after the completion or termination of the trial.

During the whole observation process, if there are any injuries caused by the serious adverse reactions

related to vaccines which are confirmed by the expert investigation team, the sponsor should give corresponding compensations according to the *Regulations on the Administration of Vaccine Circulation and Vaccination* and *Basic Insurance Compensation Measures for Abnormal Reactions of Vaccination in Henan Province (Interim Version)*.

(2) Procedure for reporting serious adverse events

① Researchers' reporting procedure

For any serious adverse events no matter whether they are related to the tested vaccines or not, researchers must submit the first report of the *Serious Adverse Event Report* form to the provincial drug administration, the sponsor, and the ethics committee by fax, email, EDC system, or personal delivery within 24 hours after being informed. Follow-up reports of *Serious Adverse Event Report* form should be submitted regularly until the end of the event. All information should be reported in the *Serious Adverse Event Report* form, including the description of adverse reactions/events, attack time and type, duration, intensity, causal relationship with vaccination, results, treatment methods (symptomatic treatment), and other relevant clinical and laboratory data.

When receiving the serious adverse event/reaction report, the investigators and the sponsor should comprehensively evaluate the duration, scope, intensity, outcome, and the willingness of participants to determine whether the participants could continue to participate in the trial or terminate the trial in advance.

③ Sponsor's reporting process

During the clinical trial, the sponsor should report the adverse response related to vaccines and suspected unexpected serious adverse reactions (SUSAR) in the form of individual safety report according to the *Standards and Procedures for Rapid Reporting of Safety Data during the Clinical Trial of Drugs*. When the investigators and the sponsor fail to reach an agreement in the judgment of the causal relationship

between adverse events and vaccines, if either of them could not definitely reject the causal relationship between vaccines and adverse events, the sponsor should also report them quickly.

For a fatal or life-threatening SUSAR, the sponsor should report it as soon as possible (no more than 7 days after being informed) and report and complete the follow-up information within the next 8 days (Note: the day when the sponsor is informed is day 0). When the SUSAR is nonfatal or not life-threatening, the sponsor should report it as soon as possible (no more than 15 days after being informed). For other potential serious safety risk information, the sponsor should also report it to the national drug review agency as soon as possible and make medical and scientific judgment for each event. After the first report, the sponsor should continue to follow up the serious adverse reactions and timely submit the follow-up report of new relevant information or changes of information (no more than 15 days after being informed).

11.6.4 Outcome of serious adverse events

The outcomes of serious adverse events include (1) symptom disappearance (with sequelae); (2) symptom disappearance (without sequelae); (3) symptom persistence; (4) symptom worsening; (5) death.

11.7 Measures for improving compliance

- According to the clinical trial protocol, a simple and clear volunteer recruitment form and informed consent form are developed;
- Researchers and doctors will receive training on how to communicate with volunteers with lay language to make them fully informed;
- Participants are selected according to the inclusion and exclusion criteria;
- Personnel involved in follow-up should have a high sense of responsibility and professionalism. Relevant training would be held to improve their communication skills and affinity. During the follow-up of safety, measures should be taken to ensure the effective contact between the

participants and researchers, timely handling adverse reactions, and providing relevant health consultation.

11.8 Blood sample management and numbering rules

11.8.1 Blood sample management

Humoral immunity

Four mL of venous blood is collected from the participants. Within 24 hours after collection, serum is separated in the laboratory in the research field. Serum will be separated and divided into three tubes (tube A and tube B for measurements, each tube contains no less than 0.5 ml of serum, and tube C for storage). The serum samples should be stored at - 20 °C or below and managed by staff, and sample storage files and temperature and humidity records should be established. The samples for humoral immunity are frozen and transported to National Institutes for Food and Drug Control and Wuhan Institute of Virology, Chinese Academy of Sciences. The tube C should not be transported at the same time with the tube A and tube B. Repeated freezing and thawing should be avoided during transportation. Tube C should be properly kept by the investigators until the clinical trial report is completed, and tube C should be processed and recorded after being confirmed by the sponsor.

Cellular immune safety biomarkers:

About 4 mL of venous blood added anticoagulant and 3 mL of venous blood not added anticoagulant are collected and stored at 2-8 °C. The samples are transferred to Zhengzhou Jinyu Clinical Laboratory Center, and tests should be completed within 24h after collection.

11.8.2 Blood sample numbering rules

Table 21. Antibody test and cellular immune biomarker blood sample number

Sampling time	Humoral immune (antibody)	Phase 1 cellular immune

	blood sample No.	safety biomarker blood sample No.
Before the 1st dose	Study No. - T-0	Study No. - S-0
4 days after the 1st dose	Study No. - T-1	-
14 days after the 1st dose	Study No. - T-2	Study No. - S-2
21 days after the 1st dose	Study No. - T-3	-
Before the 2nd dose (28 days after the 1st dose)	Study No. - T-4	Study No. - S-4
4 days after the 2nd dose	Study No. - T-5	-
14 days after the 2nd dose	Study No. - T-6	Study No. - S-6
Before the 3rd dose (28 days after the 2nd dose)	Study No. - T-7	Study No. - S-7
4 days after the 3rd dose	Study No. - T-8	-
14 days after the 3rd dose	Study No. - T-9	
28 days after the 3rd dose	Study No. - T-10	Study No. - S-10
90 days after vaccination	Study No. - T-11	-
180 days after vaccination	Study No. - T-12	Study No. - S-12
360 days after vaccination	Study No. - T-13	Study No. - S-13

11.9 Vaccine management

The investigator should guide the research field to establish the management system of the investigational vaccines. The management of vaccines including the receipt, storage, preparation, recycle,

and return/destruction should meet the requirements of relevant laws and regulations. The investigator and the research field should designate staffs who received GCP and relevant training to manage the vaccines.

Vaccine delivery: The whole process of vaccine management should meet the requirements of cold chain, and there should be corresponding equipment and facilities used for vaccine transportation and storage. During the vaccine delivery, there should be a delivery record and temperature monitoring. When the vaccines arrive at research field, the packaging situation and the unpacking temperature should be recorded. After receiving the vaccine, the designated personnel should sign the delivery record which would be later faxed or copied to the sender, and both parties should keep the delivery record properly.

Storage, distribution, and use of vaccine: The investigational vaccine should be stored in a separate locked area and managed by designated personnel. Those who receive the vaccines should check and record the batch number, expiry date, and delivery status of the vaccine, create a form to record the handover, registration, use, and recycle of the vaccine. The form should be filled in as required and kept in the work record.

Vaccine handover record: The sponsor should provide the investigational vaccine and placebo, and vaccine handover sheet. Researchers should check the name, batch number, and quantity of the vaccines when receiving them.

Vaccine registration and use records: Researchers should create vaccine registration and use records, and they should distribute investigational vaccine and placebo according to the protocol.

Vaccine recycle record: The abandoned, expired, and remaining vaccines in this trial should be returned to the sponsor. The sponsor should check the batch number and quantity of the vaccines when receiving them, they should also fill in the vaccine handover form and make relevant records, which should be later signed by the vaccine administrator and the sponsor.

The investigational vaccine and placebo should not be used for other people out of this clinical trial.

11.10 Calibration and standardization of instruments and equipment

— The refrigerator should be calibrated and within expiry dates. The temperature should be monitored and recorded for at least three days before use;

— Thermometers and other measuring instruments should be standardized;

— The centrifuge should operate normally and be within expiry dates. The usage should be recorded during use;

— The injectors for vaccination and blood collection should be disposable and sterile. The manufacturer should possess the national manufacturing license and record the batch numbers and expiry dates.

11.11 Quality control management of clinical trial data

11.11.1 Original data

The original data should include the demographic data, medical history information, physical examination results, vaccination records, information on uses of other drugs, and adverse events/reactions and their treatment and outcome, etc. All information should have the original records which are stored in a special room. The original data should be filed at the research field.

Researchers should carefully, accurately, and timely complete the original records on the day when acquiring the information. Black signing pens should be used to fill in the form, and the wrong part should not be covered, but should be marked with a line, and the correct content should be filled in the side with a signature and a date. The original record should include the following data.

Trial name, random sequence of the participant

Informed consent

Demographic data

Inclusion/exclusion criteria

Vaccination record

Date of follow-up and date of termination

Adverse events/reactions and their treatment and outcome

Accompanying medical treatment and other drugs/vaccinations

11.11.2 Electronic case report form (eCRF) and electronic database

All data recorded in the electronic case report form database should be from the original record.

The trial inspector authorized by the sponsor has the right to access all data at any time.

11.11.3 Management and storage of clinical trial materials

Materials in clinical trials should be kept in accordance with Appendix 2 of GCP, and researchers should keep these materials for at least 5 years after the termination of clinical trials. The sponsor should keep these materials for at least 5 years after the drug is put on the market.

(1) After the completion of the trial, clinical trial folders should be sorted out according to the requirements of GCP and respectively stored in the provincial CDC and research field. The materials delivered to the sponsor should be sorted out according to the requirements of this protocol, and all parties should record data handover;

(2) File management should be carried out in accordance with SOP, and the signboard including project name, completion date, sponsor, and storage period should be made. Safety measures such as insect prevention, moisture-proof, fire prevention, and anti-theft should be made;

(3) The use and access of materials/documents in this trial are limited to the relevant personnel from CDC, the sponsor (including the project inspectors), and inspectors from NMPA. Researchers should keep the clinical trial materials for at least 5 years after the termination of the clinical trial. After five years,

researchers should inform the sponsor, and no one would be allowed to dispose of the materials without the written permission from the sponsor.

11.12 Relevant documents

Relevant documents should be collected and filed according to the requirements of *Good Clinical Practice of Pharmaceutical Products* (Appendix 2).

11.12.1 Before clinical trial

(1) The following documents should be submitted to Ethics Committee for approval.

- Application form to the ethics committee
- Introduction of clinical trial (provided by the sponsor)
- Test report of experimental vaccine or certificate of batch issuance of biological products (provided by the sponsor)
- Letter of authorization from the sponsor (provided by the sponsor)
- Clinical trial protocol (signed by sponsor and investigators)
- Sample informed consent (sponsor/investigator)
- Sample recruitment advertisement (sponsor/investigator)
- Investigator's manual (sponsor)
- Sample electronic case report form (eCRF) (sponsor/investigator)
- Sample diary card/contact card (sponsor/investigator)
- Resume of main investigators (investigator)

The IRB approval certificate should be provided in quadruplicate, one for ethics committee, one for investigator, and two for the sponsor.

(2) Registration in SFDA

- Relevant approval documents of clinical trial
- Test report of experimental vaccine or certificate of batch issuance of biological products
- Clinical trial protocol (signed)
- Approval certificate of Ethics Committee (investigator)
- Informed consent (sample)
- Resume of main investigators

(3) Trial contract (investigators and the sponsor): in quadruplicate, two originals for each party.

11.12.2 After the end of the trail

(1) Investigators keep

- List of participants
- Random selection table
- Informed consent (signed)
- Diary card/contact card (followed up)
- Electronic case report form
- Original record form
- Original data from the research field
- Test reports of biological samples
- Statistical analysis report
- Clinical trial summary report

(2) Sponsor folder

The investigator should provide clinical trial data to the sponsor in accordance with the *Measures for the Administration of Drug Registration*.

- Approval of ethics committee
- Sample informed consent
- Information and qualification of clinical research units (practice license of medical institutions, legal person certificate of public institutions, etc.)
- Resume and authorization form of main investigators
- Clinical trial research protocol, modification contents of the protocol, and approval document of the Ethics Committee for the modification contents
- Case report form, diary card, contact card sample form
- eCRF
- Test report of the experimental vaccine or certificate of batch issuance of biological products
- Instructions for vaccines
- Laboratory qualification and quality control certificate (technical service unit)
- List of participants
- Serious adverse event report form and relevant information
- Serum test report
- Statistical analysis plan
- Statistical analysis report
- Clinical trial summary report

11.12.3 Protocol and clinical trial report

The responsibilities of all parties to the clinical trial should be stipulated in the agreement, which should come into force after being signed and sealed by all parties concerned. The original agreement is in quadruplicate, two for the sponsor and two for the investigators.

Clinical summary report: 6 copies, 2 copies kept by investigators, 4 copies used by the sponsor to apply for new drug certificate and approval number.

12. Ethics committee

12.1 Review and approval

This clinical trial protocol should be approved by the local IRB. The main investigator should submit the clinical trial protocol and all necessary additional documents to the Ethics Committee (specific document list could be obtained according to the requirements of the local ethics committee). After the ethics committee reviews and approves the application, an approval certificate will be provided.

12.2 Inspection in the research field

During the whole process of the trial, the ethics committee should inspect whether there are ethical problems that might harm the participants and whether the participants get treatment, compensations, and corresponding measures when they are harmed by the experiment. The ethics committee should also evaluate the risk that the participants might face.

12.2.1 Informed consent

The methods of selecting participants and providing relevant information to participants should be complete and easy to understand; the ethics committee should check whether the methods of obtaining informed consent are appropriate. During the whole process of the trial, the ethics committee should regularly review the progress of the trial and assess the risks and benefits for the participants.

12.2.2 Confidentiality

Ensure that the privacy of the participants should not be disclosed during the trial, in the report and any publications. The blood samples should only be identified by the participants' ID code, blood sample number,

blood sampling time, and test items.

12.2.3 Potential hazards and hazard minimization

The inspection could be performed to check whether the adverse reactions related to vaccination (such as abscess at the vaccination site and rash after vaccination) are treated in time according to relevant regulations, and whether those who have life-threatening events are sent to the hospital for treatment and report immediately, and whether there are measures to ensure that experienced and trained medical personnel could collect venous blood according to the specified procedures under supervision, which could minimize the agony (including pain and infection of venipuncture site with low probability) of the participants.

13. References

1. Wu A, Peng Y, Huang B, et al. Genome Composition and Divergence of the Novel Coronavirus (2019-nCoV) Originating in China. *Cell Host Microbe.* 2020;27(3):325-328.

2. Li F. Structure, Function, and Evolution of Coronavirus Spike Proteins. *Annu Rev Virol.* 2016;3(1):237-261.

3. The 2019-nCoV Outbreak Joint Field Epidemiology Investigation Team, Li Q. Notes from the field: an outbreak of NCIP (2019-nCoV) infection in China — Wuhan, Hubei Province, 2019–2020. *China CDC Weekly.* 2020;2:79-80.

4. Chan JF-W, Yuan S, Kok K-H, et al. A familial cluster of pneumonia associated with the 2019 novel coronavirus indicating person-to-person transmission: a study of a family cluster. *The Lancet.* 2020;395(10223):514-523.

5. Yu F, Du L, Ojcius DM, Pan C, Jiang S. Measures for diagnosing and treating infections by a novel coronavirus responsible for a pneumonia outbreak originating in Wuhan, China. *Microbes and Infection.* 2020;22(2):74-79.

6. Shanghai Clinical Treatment Expert Group for Corona Virus Disease 2019. Comprehensive treatment and management of corona virus disease 2019: expert consensus statement from Shanghai. *Chin J Infect Dis.* 2020;38.

7. World Health Organization. WHO Coronavirus Disease (COVID-19) Dashboard. 2020; https://covid19.who.int/. Accessed July 3, 2020.

8. Shi Y, Wang N, Zou Q. Progress and Challenge of Vaccine Development Against 2019 Novel Coronavirus (2019-nCoV). *Zhonghua Yu Fang Yi Xue Za Zhi.* 2020;54(0):E029.

9. Zhu N, Zhang D, Wang W, et al. A Novel Coronavirus from Patients with Pneumonia in China, 2019. *N*

Engl J Med. 2020;382(8):727-733.

10. Lu R, Zhao X, Li J, et al. Genomic characterisation and epidemiology of 2019 novel coronavirus: implications for virus origins and receptor binding. *The Lancet.* 2020;395(10224):565-574.
11. Li Q, Guan X, Wu P, et al. Early Transmission Dynamics in Wuhan, China, of Novel Coronavirus-Infected Pneumonia. *N Engl J Med.* 2020;382(13):1199-1207.
12. Wrapp D, Wang N, Corbett KS, et al. Cryo-EM structure of the 2019-nCoV spike in the prefusion conformation. *Science (New York, NY).* 2020;367(6483):1260-1263.
13. Chinese Thoracic Society, Chinese Society of General Practice, Chinese Association of Chest Physician, et al. Expert Recommendations for the Prevention and Control of COVID-19 Infections in Primary Care (First Edition). *Chin J Gen Pract.* 2020;19.
14. National Health Commission & National Administration of Traditional Chinese Medicine. Diagnosis and Treatment Protocol for Novel Coronavirus Pneumonia (Trial Version 7). *Chin Med J.* 2020;133(9):1087-1095.
15. Zhu F-C, Li Y-H, Guan X-H, et al. Safety, tolerability, and immunogenicity of a recombinant adenovirus type-5 vectored COVID-19 vaccine: a dose-escalation, open-label, non-randomised, first-in-human trial. *The Lancet.* 2020;395(10240):1845-1854.
16. Ababneh M, Alrwashdeh M, Khalifeh M. Recombinant adenoviral vaccine encoding the spike 1 subunit of the Middle East Respiratory Syndrome Coronavirus elicits strong humoral and cellular immune responses in mice. *Veterinary world.* 2019;12(10):1554-1562.
17. Businesswire. Merck’s ERVEBO® [Ebola Zaire Vaccine (rVSVΔG-ZEBOV-GP) live] Granted Conditional Approval in the European Union. 2019; https://www.businesswire.com/news/home/20191111005601/en. Accessed July 3, 2020.

18. McKee S. US approves Merck's Ebola vaccine. 2019; http://www.pharmatimes.com/news/us_approves_mercks_ebola_vaccine_1320832. Accessed July 3, 2020.

19. EN-CPhI.CN®. The first recombinant Ebola virus vaccine was approved by CFDA. 2017; https://www.en-cphi.cn/news/show-39703.html. Accessed July 3, 2020.

20. China Daily. Nations progressing with COVID-19 vaccine tests. 2020; http://ex.chinadaily.com.cn/exchange/partners/45/rss/channel/www/columns/2n8e04/stories/WS5e71a3c7a3101282172800f8.html. Accessed July 3, 2020.

21. Loftus P. Drugmaker Moderna Delivers First Experimental Coronavirus Vaccine for Human Testing. 2020; https://www.wsj.com/articles/drugmaker-moderna-delivers-first-coronavirus-vaccine-for-human-testing-11582579099?mod=hp_lead_pos3. Accessed July 3, 2020.

14. Accessory: informed consent

14.1 Informed consent for the phase 1 trial

Informed consent for the phase 1 trial of an inactivated vaccine (Vero cell) for COVID-19

No.:____________

The purpose of this informed consent is to invite you to take part in a clinical trial which will investigate the safety and immunogenicity of an inactivated vaccine (Vero cell) for the COVID-19 developed by the Wuhan Biological Products Institute Co., Ltd. Before you decide whether you will participate in the trial, this informed consent will give you a detailed explanation of the whole research process, risks, benefits, and other related issues, and please read the following information carefully. If you have any questions, please consult the doctors or researchers of the study.

Study title: A phase 1 trial of an inactivated vaccine (Vero cell) for COVID-19

Sponsor: Wuhan Biological Products Institute Co., Ltd

Investigators:

Henan Provincial Center for Disease Control and Prevention

Wuzhi County Center for Disease Control and Prevention, Jiaozuo City, Henan Province

This informed consent includes two parts: the first part is the general information of the study, including the disease, vaccine, the study procedures, risks and benefits of participation in the study; and the second part is the signature part if you agree to participate in the study. The informed consent is made in duplicate and you will keep one copy for your reference.

1. Introduction of the disease and vaccine

COVID-19 has quickly spread around the world and become a global pandemic. As an acute respiratory infectious disease, the disease has been included in the class B infectious disease according to the Law of the People's Republic of China on the Prevention and Treatment of Infectious Diseases and is managed as class A infectious disease. The disease has been named as Corona Virus Disease 2019 (COVID-19), and the virus causing the disease was named as SARS-CoV-2. There is currently no specific treatment drugs or prevention vaccines available for the COVID-19, and thus development of a safe and effective vaccine is important to combat the COVID-19 pandemic.

The main route of transmission is through respiratory droplets and close contact. When exposed to high concentration aerosols in a relatively closed environment, it is possible to propagate through aerosols. Because the virus can be isolated from feces and urine, attention should also be paid to the spread of aerosols or contacts caused by environmental pollution by feces and urine. All people are susceptible to the infection. The elderly and those with chronic diseases are at a higher risk of poor prognosis. The symptoms of children are relatively mild.

This inactivated vaccine (Vero cell) for COVID-19, developed by the Wuhan Biological Products Institute Co., Ltd., was approved and examined by the National Medical Products Administration according to *Drug Administration Law of the People's Republic of China*, *Vaccine Administration Law of the People's Republic of China*, and other related regulations. The inactivated vaccine (Vero cell) was produced by inoculating COVID-19 WIV04 strain in Vero cells, which was then cultured, harvested, inactivated, clarified, concentrated, secondary inactivated, purified, and finally made with the adjuvant of aluminum hydroxide. The inactivated vaccines have been commonly used for other infections or diseases, such as the DTP (Diphtheria/Tetanus/Pertussis) vaccine, enterovirus 71 inactivated vaccine (Vero cell), and are in general safe

and effective. The investigational inactivated vaccine (Vero cell) for COVID-19 has been tested in the preclinical studies and shown promising effectiveness. The study was reviewed and approved by medical ethics committee of Henan Provincial Center for Disease Control and Prevention. All vaccines and placebos have passed the examination of National Institute for Food and Drug Control, China, and obtained the examination approval certificate.

2. Expected number of participants: 96

3. Inclusion and Exclusion criteria

3.1 Inclusion criteria

- Healthy people aged ≥18 years old;
- General good health as confirmed by medical history and physical examination;
- Not confirmed or suspected COVID-19 cases or asymptomatic cases.
- Since December 2019, the participant has not gone to Hubei province, overseas, or to a village/community where there were reported COVID-19 cases; or has not closely contacted with confirmed or suspected cases, are currently not in quarantine, and are not from a village/community where there have been confirmed or suspected cases.
- Women who have reached menarche are not pregnant (negative urine pregnancy test), are not breastfeeding, have no birth plan within three months after enrollment, and have taken effective contraceptive measures two weeks before enrollment;
- Able and willing to complete the whole research process of about 14 months;
- Participants have the ability to understand the research procedures, sign the informed consent voluntarily after informed consent, and can comply with the requirements of the clinical research program.

3.2 Exclusion criteria

- Those with positive antibody tests of the COVID-19;
- History of SARS virus infection (identified through self-report or on-site inquiry)
- Those with congenital malformation, developmental disorder, genetic defect, or severe malnutrition, etc;
- Those with confirmed or suspected serious respiratory diseases, serious cardiovascular diseases, malignant tumors, severe hepatorenal diseases, uncontrolled hypertension (systolic blood pressure ≥140 mmHg, diastolic blood pressure ≥90 mmHg), diabetes complications, or various acute or chronic diseases (acute attack stage);
- Those diagnosed with congenital or acquired immunodeficiency, HIV infection, lymphoma, leukemia, or other autoimmune diseases;
- Those with history or family history of convulsion, epilepsy, encephalopathy, or mental illness;
- Those with a history of abnormal coagulation (such as lack of coagulation factors or coagulation diseases);
- Those receiving anti-TB treatment;
- Those receiving immunoenhancement or inhibitor treatment (p.o. or gtt.) over 14 days within the past three months (continuous oral or infusion for more than 14 days);
- Those receiving live attenuated vaccines within one month before inoculation or other vaccines within 14 days before inoculation;
- Those with fever (axillary temperature >37.0 °C), dry cough, fatigue, nasal obstruction, runny nose, sore throat, myalgia, diarrhea, shortness of breath, and dyspnea within 14 days before inoculation;
- Those with clinically abnormal parameters from blood biochemical, blood routine, and urine

routine before inoculation;

- Axillary temperature >37.0 °C before inoculation;
- Those who have experienced severe allergic reactions (such as acute anaphylaxis, urticaria, eczema, dyspnea, neurovascular edema, or abdominal pain), or those who are allergic to the known gradients of COVID-19 inactivated vaccine;
- Those receiving blood products within 3 months before inoculation;
- Those receiving other study drugs in a clinical trial within 6 months before inoculation;
- Those who are not suitable for the clinical trial as evaluated by the researchers.

4. Research procedure

Your consent will be obtained prior to any research-related activities.

(1) Informed consent: The physician will introduce the contents of this study to you. After fully understanding the process and requirements, if you agree to participate in this study, please sign this informed consent voluntarily.

(2) After signing the informed consent form, the physician will conduct physical examination and ask some questions about you, including:

- Temperature, height, weight, blood pressure, heart and lung auscultation, skin and throat examination, etc;
- Women who have reached menarche need to be tested for urine pregnancy test;
- Inquiry and record your health information (including medical history and current disease status);
- Fasting blood samples (9 mL) will be collected for blood routine test, blood biochemical test, novel coronavirus antibody screening, and antibody measurements. An additional 7 mL of blood will be collected for cellular immune test.

(3) If you pass the physical examination and meet the inclusion criteria but not the exclusion criteria, you will be randomly assigned a study number.

(4) Vaccination: according to the study number, you will receive a vaccine or a placebo, which will be randomly decided by the computer program and no one including the researchers will know in advance. The vaccine will be injected three times on days 0, 28, and 56. There are three dose groups for the vaccine: low-dose, medium-dose, and high-dose groups. Therefore, you will be randomly assigned to one of the four groups: placebo, low-dose, medium-dose, and high-dose groups.

(5) 30 minutes stay: after inoculation, you need to be observed for the immediate response in the stay room for 30 minutes. At the same time, the physician will give you a thermometer, a scale, a pen, and a diary card, and explain how to correctly measure the temperature, observe the physical discomfort, and record any adverse events on the diary card.

(6) Biospecimen sample collection: blood and urine will be collected for laboratory tests (hepatorenal function test, blood routine test, and urine routine test), antibody test, cellular immunity, and immune durability observation. The specific blood collection time points and blood volumes are as follows.

- Laboratory tests (hepatorenal function test, blood routine test and urine routine test): 5 mL of venous blood will be collected for hepatorenal function test and blood routine test before each inoculation and on the fourth day after each inoculation. Appropriate amount of urine will be collected for urine routine test at the same time.
- Antibody test: about 4 mL of venous blood will be collected before each inoculation, on days 4 and 14 after each inoculation, on day 21 after the first inoculation, and on day 28 after the whole course.
- Cellular immunity biomarkers: about 7 mL of venous blood will be collected before each

inoculation, on day 14 after each inoculation, and on day 28 after the whole-course immunization.

- Observation of immune durability: about 4 mL of venous blood will be collected on days 90, 180, and 360 after the whole-course immunization to evaluate immune durability. About 7 mL of venous blood will be collected for on days 180 and 360 after the whole-course immunization to evaluate cellular immune.

(7) Follow up after inoculations: researchers will visit you once a day in the village/community on days 0 to 7 after the first inoculation to inquire about the occurrence of adverse events and collect the diary cards. After the second and third vaccination, a phone call will be made within 24 hours, a face-to-face visit will be made within 3 days, and another phone call will be made within 4 to 7 days to inquire about the occurrence of adverse events and information on the diary cards. On the 8th day after each inoculation, researchers will collect and review diary cards and give out contact cards. Between the 8th and 28th/30th days after each inoculation, weekly phone calls will be made, and contact cards will be collected on 28th/30th day.

From the first inoculation to 12 months after the last inoculation, we will make regular phone-call follow-ups, and if you have anything that you want to let us know, please call us anytime. If you are hospitalized due to illness or other conditions during the study, please do call our follow-up physicians. In addition, if you have any other questions, please feel free to contact us.

5. The total research period is about 14 months.

6. Related risks

Some common reactions may occur during the inoculation process, including pain, tenderness, redness, induration, and pruritus at the injection site; systemic reactions such as fever, headache, fatigue, nausea and vomiting, diarrhea, cough, allergy, myalgia, arthralgia. Generally, the symptoms will subside by themselves.

If your symptoms persist or become worse, you will be given symptomatic treatment by the physicians. Occasionally, there might be severe allergic reactions, and you will receive timely treatment. We will take care of all the treatments.

Very rarely, infection enhancement effect might occur in very few people who once received this vaccine and later get infected with COVID-19. We will provide you and your family with knowledge about prevention of getting infected by the SARS-CoV-2, and provide necessary personal protective equipment such as facial masks and disinfectants if there are confirmed COVID-19 cases locally. If you is infected during the trial, the researchers will immediately contact the local hospital, and the hospital will give priority to your hospitalization and timely treatment.

After venous blood collection, a few people may have skin bruises at the injection site.

In this study, the participants who will be injected with placebo cannot be protected from SARS-CoV-2 infection. However, no one, including the researchers, will know who gets vaccine and who gets placebo.

7. Benefits

This is a pre-market safety study of vaccine, which cannot guarantee that you will benefit from it. The inactivated vaccines may protect you from getting COVID-19 if infected by the SARS-CoV-2, which will depend on the immune response. Due to the individual differences of immune system, some participants may not get immune protection.

You do not have to pay for vaccination, medical examination, or all other research related processes.

8. Compensation

If you arrive at the research field by yourself, you will be paid a transportation allowance. There will also be small gifts given to you for participating in the trial.

If you re vaccinated with placebo after we unblind the group assignment at the end of the trial, we will

provide you with this vaccine free of charge after the approval of market launch of this study vaccine. During the whole observation process, if you have any physical injury caused by serious adverse reactions that are judged by expert investigation team to be related to the vaccine, the sponsor will give corresponding compensation according to the national *Regulations on the Administration of Vaccine Circulation and Vaccination* and *Basic Insurance Compensation Measures for Abnormal Reactions of Vaccination in Henan Province (Interim version)*.

9. Alternative vaccines

At present, there is no licensed COVID-19 vaccine on the market in China or any other country.

10. Confidentiality agreement

All personal information related to you will be strictly confidential. You will be assigned a study number to identify you the study. The original data will be kept by the Centers for Disease Prevention and Control of Henan province and Wuzhi county. The government authorities, the inspectors and monitors appointed by the sponsor (Wuhan Institute of Biological Products Co., Ltd.), and the ethics committee of Henan province Center for Disease Prevention and Control may examine the original material with your information to verify the accuracy of the data according to the regulations. When the investigators submit the research material to the sponsor, your personally information will be hidden. The results may be published in the scientific journals, but will not list any identity information or privacy about you. The data collected during the trial will be used for analysis in this study only and will not be used for other purposes without your approval.

11. Right to withdraw from our study

We hope that you will participate in the whole process, but your participation is completely voluntary, and you have the right to request to withdraw at any time. No matter how you decide, your withdrawal will not be subject to any penalty or loss of interest. Your medical treatment and rights will not be affected, and

you will not be required to pay any fees. If you decide not to participant in the study or to withdraw from the study at any time, please contact us.

Of course, the study physician may also ask you to withdraw from the study at any time. This may be due to a number of different reasons, such as concerns about your health status, your failure to follow the study's protocol, or the study being suspended or terminated.

If any serious adverse events (including those need hospitalizations and are considered to be related to the study vaccine) occur during the study period, the study may be terminated immediately. The researchers will inform you of the study suspension and the reason for the suspension and give you necessary instructions. Once it is confirmed that it is safe to continue the study, you can also decide whether to continue the study based on the information provided.

12. Other matters

Your blood and urine samples will be properly stored for laboratory measures according to the trial protocol and relevant regulations. The results may not be actively fed back to you if there is no abnormal value that needs immediate medical attention. After the trial is ended and if you need the feedback of your results, we will provide you the results you need at appropriate time. Your data and biospecimen samples will be stored for at least 5 years according to the trial regulations.

If there is any new information that may affect your decision to continue to participate in the study, we will inform you in a timely manner and communicate with you again.

13. Contact information

If you have any questions that need consultation and help, you can call the following number at any time:

Investigators:

Henan province Center for Disease Control and Prevention (vaccine clinical research center); Director Xia,

Tel: XXX;

Wuzhi county Center for Disease Control and Prevention; Director Li, Tel: XXX;

For questions about your rights as a participant, please contact:

Medical ethics committee of Henan Center for Disease Control and Prevention; Director Xu, Tel: XXX.

No.:_______________

14. Sign the informed consent

14.1 Signature of the participant

Please read out the following boxes:

I have read and understood all the above contents in detail, I have understood and recognized all the requirements of the study, and the doctor has explained and answered all my questions in detail to me; after full consideration, I voluntarily to participate in the study, and sign my name on the informed consent.

Participant's name:______________________ (filled in by investigator)

Participant's signature:_______________ Time: MM/DD/YYYY/H/M

14.2 Signature of witness (if any, please read out the following box):

Because the participant could not read the contents of the informed consent correctly, the doctor has informed all the contents on site, and the participant has understood all the contents of the informed consent and agreed to participate in this study. As a third-party witness, I witnessed the whole informed consent process.

Name of witness:_________ Contact information of witness:________ (filled in by investigator)

Signature of witness:________________ Date: MM/DD/YYYY/H/M

14.3 Signature of the Investigator

I promise that I have introduced the relevant information of this study to the recipients and made full communication. I have given full and satisfactory answers to any questions raised by the recipients. I guarantee that the recipients sign the informed consent under the condition of full notification, full understanding, and independent choice, and will give a copy of the signed informed consent to the recipient.

Signature of investigator: _______________ Time: MM/DD/YYYY/H/M

14.2 Informed consent for the phase 2 trial

Informed consent for the phase 2 trial of an inactivated vaccine (Vero cell) for COVID-19

No.:____________

The purpose of this informed consent is to invite you to take part in a clinical trial which will investigate the safety and immunogenicity of an inactivated vaccine (Vero cell) for the COVID-19 developed by the Wuhan Biological Products Institute Co., Ltd. Before you decide whether you will participate in the trial, this informed consent will give you a detailed explanation of the whole research process, risks, benefits, and other related issues, and please read the following information carefully. If you have any questions, please consult the doctors or researchers of the study.

Study title: A phase 2 trial of an inactivated vaccine (Vero cell) for COVID-19

Sponsor: Wuhan Biological Products Institute Co., Ltd

Investigators:

Henan Provincial Center for Disease Control and Prevention

Wuzhi County Center for Disease Control and Prevention, Jiaozuo City, Henan Province

This informed consent includes two parts: the first part is the general information of the study, including the disease, vaccine, the study procedures, risks and benefits of participation in the study; and the second part is the signature part if you agree to participate in the study. The informed consent is made in duplicate and you will keep one copy for your reference.

1. Introduction of the disease and vaccine

COVID-19 has quickly spread around the world and become a global pandemic. As an acute respiratory infectious disease, the disease has been included in the class B infectious disease according to the Law of the People's Republic of China on the Prevention and Treatment of Infectious Diseases and is managed as class A infectious disease. The disease has been named as Corona Virus Disease 2019 (COVID-19), and the virus causing the disease was named as SARS-CoV-2. There is currently no specific treatment drugs or prevention vaccines available for the COVID-19, and thus development of a safe and effective vaccine is important to combat the COVID-19 pandemic.

The main route of transmission is through respiratory droplets and close contact. When exposed to high concentration aerosols in a relatively closed environment, it is possible to propagate through aerosols. Because the virus can be isolated from feces and urine, attention should also be paid to the spread of aerosols or contacts caused by environmental pollution by feces and urine. All people are susceptible to the infection. The elderly and those with chronic diseases are at a higher risk of poor prognosis. The symptoms of children are relatively mild.

This inactivated vaccine (Vero cell) for COVID-19, developed by the Wuhan Biological Products Institute Co., Ltd., was approved and examined by the National Medical Products Administration according to *Drug Administration Law of the People's Republic of China*, *Vaccine Administration Law of the People's Republic of China*, and other related regulations. The inactivated vaccine (Vero cell) was produced by inoculating COVID-19 WIV04 strain in Vero cells, which was then cultured, harvested, inactivated, clarified, concentrated, secondary inactivated, purified, and finally made with the adjuvant of aluminum hydroxide. The inactivated vaccines have been commonly used for other infections or diseases, such as the DTP (Diphtheria/Tetanus/Pertussis) vaccine, enterovirus 71 inactivated vaccine (Vero cell), and are in general safe

and effective. The investigational inactivated vaccine (Vero cell) for COVID-19 has been tested in the preclinical studies and shown promising effectiveness. The study was reviewed and approved by medical ethics committee of Henan Provincial Center for Disease Control and Prevention. All vaccines and placebos have passed the examination of National Institute for Food and Drug Control, China, and obtained the examination approval certificate.

2. Number of subjects: 1270

3. Inclusion and Exclusion criteria

3.1 Inclusion criteria

- healthy children aged 3-17 years;
- General good health as confirmed by medical history and physical examination;
- Not confirmed or suspected COVID-19 cases or asymptomatic cases.
- Since December 2019, the participant has not gone to Hubei province, overseas, or to a village/community where there were reported COVID-19 cases; or has not closely contacted with confirmed or suspected cases, are currently not in quarantine, and are not from a village/community where there have been confirmed or suspected cases.
- Women who have reached menarche are not pregnant (negative urine pregnancy test), are not breastfeeding, have no birth plan within three months after enrollment, and have taken effective contraceptive measures two weeks before enrollment;
- Able and willing to complete the whole research process of about 14 months;
- Participants have the ability to understand the research procedures, sign the informed consent voluntarily after informed consent, and can comply with the requirements of the clinical research program.

3.2 Exclusion criteria

- Those with positive antibody tests of the COVID-19;
- History of SARS virus infection (identified through self-report or on-site inquiry);
- Those with congenital malformation, developmental disorder, genetic defect, or severe malnutrition, etc;
- Those with confirmed or suspected serious respiratory diseases, serious cardiovascular diseases, malignant tumors, severe hepatorenal diseases, uncontrolled hypertension (systolic blood pressure ≥140 mmHg, diastolic blood pressure ≥90 mmHg), diabetes complications, or various acute or chronic diseases (acute attack stage);
- Those diagnosed with congenital or acquired immunodeficiency, HIV infection, lymphoma, leukemia, or other autoimmune diseases;
- Those with history or family history of convulsion, epilepsy, encephalopathy, or mental illness;
- Those with a history of abnormal coagulation (such as lack of coagulation factors or coagulation diseases);
- Those receiving anti-TB treatment;
- Those receiving immunoenhancement or inhibitor treatment (p.o. or gtt.) over 14 days within the past three months (continuous oral or infusion for more than 14 days);
- Those receiving live attenuated vaccines within one month before inoculation or other vaccines within 14 days before inoculation;
- Those with fever (axillary temperature >37.0 °C), dry cough, fatigue, nasal obstruction, runny nose, sore throat, myalgia, diarrhea, shortness of breath, and dyspnea within 14 days before inoculation;
- Those with clinically abnormal parameters from blood biochemical, blood routine, and urine routine

before inoculation;

- Axillary temperature >37.0 °C before inoculation;
- Those who have experienced severe allergic reactions (such as acute anaphylaxis, urticaria, eczema, dyspnea, neurovascular edema, or abdominal pain), or those who are allergic to the known gradients of COVID-19 inactivated vaccine;
- Those receiving blood products within 3 months before inoculation;
- Those receiving other study drugs in a clinical trial within 6 months before inoculation;
- Those who are not suitable for the clinical trial as evaluated by the researchers.

4. Research procedure

Your consent will be obtained prior to any research-related activities.

(1) Informed consent: The physician will introduce the contents of this study to you. After fully understanding the process and requirements, if you agree to participate in this study, please sign this informed consent voluntarily.

(2) After signing the informed consent form, the physician will conduct physical examination and ask some questions about you, including:

- Temperature, height, weight, blood pressure, heart and lung auscultation, skin and throat examination, etc;
- Women who have reached menarche need to be tested for urine pregnancy test;
- Inquiry and record your health information (including medical history and current disease status);
- Fasting blood samples (9 mL) will be collected for blood routine test, blood biochemical test, novel coronavirus antibody screening, and antibody measurements. An additional 7 mL of blood will be collected for cellular immune test.

(3) If you pass the physical examination and meet the inclusion criteria but not the exclusion criteria, you will be randomly assigned a study number.

(4) Vaccination: according to the study number, you will receive a vaccine or a placebo, which will be randomly decided by the computer program and no one including the researchers will know in advance. The vaccine will be injected three times on days 0, 28, and 56. There are three dose groups for the vaccine: low-dose, medium-dose, and high-dose groups. Therefore, you will be randomly assigned to one of the four groups: placebo, low-dose, medium-dose, and high-dose groups.

(5) 30 minutes stay: after inoculation, you need to be observed for the immediate response in the stay room for 30 minutes. At the same time, the physician will give you a thermometer, a scale, a pen, and a diary card, and explain how to correctly measure the temperature, observe the physical discomfort, and record any adverse events on the diary card.

(6) Biospecimen sample collection: blood and urine will be collected for laboratory tests (hepatorenal function test, blood routine test, and urine routine test), antibody test, cellular immunity, and immune durability observation. About 4 mL of venous blood will be collected for antibody tests before each inoculation as well as 28, 90, 180 and 360 days after the whole-course immunization.

(7) Follow up after vaccination: a phone call will be made within 24 hours, a face-to-face visit will be made within 3 days, and another phone call will be made within 4 to 7 days to inquire about the occurrence of adverse events and information on the diary cards. On the 8th day after each inoculation, researchers will collect and review diary cards and give out contact cards. Between the 8th and 28th / 30th days after each inoculation, weekly phone calls will be made, and contact cards will be collected on 28th / 30th day.

From the first inoculation to 12 months after the last inoculation, we will make regular phone-call follow-ups, and if you have anything that you want to let us know, please call us anytime. If you are

hospitalized due to illness or other conditions during the study, please do call our follow-up physicians. In addition, if you have any other questions, please feel free to contact us.

5. The total research period is about 14 months.

6. Related risks

Some common reactions may occur during the inoculation process, including pain, tenderness, redness, induration, and pruritus at the injection site; systemic reactions such as fever, headache, fatigue, nausea and vomiting, diarrhea, cough, allergy, myalgia, arthralgia. Generally, the symptoms will subside by themselves. If your symptoms persist or become worse, you will be given symptomatic treatment by the physicians. Occasionally, there might be severe allergic reactions, and you will receive timely treatment. We will take care of all the treatments.

Very rarely, infection enhancement effect might occur in very few people who once received this vaccine and later get infected with COVID-19. We will provide you and your family with knowledge about prevention of getting infected by the SARS-CoV-2, and provide necessary personal protective equipment such as facial masks and disinfectants if there are confirmed COVID-19 cases locally. If you are infected during the trial, the researchers will immediately contact the local hospital, and the hospital will give priority to your hospitalization and timely treatment.

After venous blood collection, a few people may have skin bruises at the injection site.

In this study, the participants who will be injected with placebo cannot be protected from SARS-CoV-2 infection. However, no one, including the researchers, will know who gets vaccine and who gets placebo.

7. Benefits

This is a pre-market safety study of vaccine, which cannot guarantee that you will benefit from it. The inactivated vaccines may protect you from getting COVID-19 if infected by the SARS-CoV-2, which will

depend on the immune response. Due to the individual differences of immune system, some participants may not get immune protection. You do not have to pay for vaccination, medical examination, or all other research related processes.

8. Compensation

If you arrive at the research field by yourself, you will be paid a transportation allowance. There will also be small gifts given to you for participating in the trial.

If you are vaccinated with placebo after we unblind the group assignment at the end of the trial, we will provide you with this vaccine free of charge after the approval of market launch of this study vaccine. During the whole observation process, if you have any physical injury caused by serious adverse reactions that are judged by expert investigation team to be related to the vaccine, the sponsor will give corresponding compensation according to the national *Regulations on the Administration of Vaccine Circulation and Vaccination* and *Basic Insurance Compensation Measures for Abnormal Reactions of Vaccination in Henan Province (Interim version)*.

9. Alternative vaccines

At present, there is no licensed COVID-19 vaccine on the market in China or any other country.

10. Confidentiality agreement

All personal information related to you will be strictly confidential. You will be assigned a study number to identify you in the study. The original data will be kept by the Centers for Disease Prevention and Control of Henan province and Wuzhi county. The government authorities, the inspectors and monitors appointed by the sponsor (Wuhan Institute of Biological Products Co., Ltd.), and the ethics committee of Henan province Center for Disease Prevention and Control may examine the original material with your information to verify the accuracy of the data according to the regulations. When the investigators submit the research material to

the sponsor, your personally information will be hidden. The results may be published in the scientific journals, but will not list any identity information or privacy about you. The data collected during the trial will be used for analysis in this study only and will not be used for other purposes without your approval.

11. Right to withdraw from our study

We hope that you will participate in the whole process, but your participation is completely voluntary, and you have the right to request to withdraw at any time. No matter how you decide, your withdrawal will not be subject to any penalty or loss of interest. Your medical treatment and rights will not be affected, and you will not be required to pay any fees. If you decide not to participant in the study or to withdraw from the study at any time, please contact us.

Of course, the study physician may also ask you to withdraw from the study at any time. This may be due to a number of different reasons, such as concerns about your health status, your failure to follow the study's protocol, or the study being suspended or terminated.

If any serious adverse events (including those need hospitalizations and are considered to be related to the study vaccine) occur during the study period, the study may be terminated immediately. The researchers will inform you of the study suspension and the reason for the suspension and give you necessary instructions. Once it is confirmed that it is safe to continue the study, you can also decide whether to continue the study based on the information provided.

12. Other matters

Your blood and urine samples will be properly stored for laboratory measures according to the trial protocol and relevant regulations. The results may not be actively fed back to you if there is no abnormal value that needs immediate medical attention. After the trial is ended and if you need the feedback of your results, we will provide you the results you need at appropriate time. Your data and biospecimen samples will be stored

for at least 5 years according to the trial regulations.

If there is any new information that may affect your decision to continue to participate in the study, we will inform you in a timely manner and communicate with you again.

13. Contact information

If you have any questions that need consultation and help, you can call the following number at any time:

Investigators:

Henan province Center for Disease Control and Prevention (vaccine clinical research center); Director Xia, Tel: XXX;

Wuzhi county Center for Disease Control and Prevention; Director Li, Tel: XXX;

For questions about your rights as a participant, please contact:

Medical ethics committee of Henan Center for Disease Control and Prevention; Director Xu, Tel: XXX.

No.:_______________

14. Sign the informed consent

14.1 Signature of the participant

Please read out the following boxes:

I have read and understood all the above contents in detail, I have understood and recognized all the requirements of the study, and the doctor has explained and answered all my questions in detail to me; after full consideration, I voluntarily to participate in the study, and sign my name on the informed consent.

Participant's name:_____________________ (filled in by investigator)

Participant's signature:______________ Time: MM/DD/YYYY/H/M

14.2 Signature of witness (if any, please read out the following box):

Because the participant could not read the contents of the informed consent correctly, the doctor has informed all the contents on site, and the participant has understood all the contents of the informed consent and agreed to participate in this study. As a third-party witness, I witnessed the whole informed consent process.

V[illegible]

Name of witness:_________ Contact information of witness:________ (filled in by investigator)

Signature of witness:_______________ Date: MM/DD/YYYY/H/M

14.4 Signature of the Investigator

I promise that I have introduced the relevant information of this study to the recipients and made full communication. I have given full and satisfactory answers to any questions raised by the recipients. I guarantee that the recipients sign the informed consent under the condition of full notification, full understanding, and independent choice, and will give a copy of the signed informed consent to the recipient.

Signature of investigator: ______________ Time: MM/DD/YYYY/H/M
