# Supplementary material for: Safety and Immunogenicity of an Inactivated COVID-19 Vaccine, WIBP-CorV, in Healthy Children: Interim Analysis of a Randomized, Double-Blind, Controlled, Phase 1/2 Trial
Source: Front Immunol. 2022 Jun 24;13:898151. doi: 10.3389/fimmu.2022.898151 (PMC9265248; doi:10.3389/fimmu.2022.898151)
Supplement: Supplementary file 2 [file DataSheet_2.pdf]

Supplemental Online Content

Supplement to: Guo W, Duan K, Zhang Y, et al. Safety and immunogenicity of an inactivated COVID-19 vaccine, WIBP-CorV, in healthy children: interim analysis of 2 randomized clinical trials up to 180 days after full vaccination.

Supplement 2. Statistical analysis plan

This supplemental material has been provided by the authors to give readers additional information about their work.

Section 2: **Statistical analysis plan**

Protocol title: Evaluation of the safety and immunogenicity of an inactivated vaccine (Vero cell) for the COVID-19 in the healthy children aged 3-17 years : randomized, double-blind, placebo-controlled phase 1/2 clinical trials

Product name: An inactivated vaccine (Vero cell) for the COVID-19

Protocol No.: WIBP2020001SQ

Sponsor: Wuhan Institute of Biological Products Co., Ltd;

Investigator: Henan Provincial Center for Disease Control and Prevention;

Researcher: Wuzhi county Center for Disease Control and Prevention, Jiaozuo, Henan

Statistical Party: Teaching and Research Unit of Biostatistics, School of Public Health, Zhengzhou University, Zhengzhou, Henan Province, China

Version: Version 3.1

Date: June 14, 2020

1. List of abbreviations

AE	adverse event
CI	confidence interval
CDC	Centers for Disease Control and Prevention
CoV	coronavirus
DSMB	Data Safety and Monitoring Board
FAS	full analysis set
GMT	geometric mean titer
GMI	geometric mean fold increase
PPS	per-protocol set
SAE	serious adverse events
SAP	statistical analysis plan
SS	safety set

2. Introduction

The purpose of this statistical analysis plan (SAP) is to present the statistical methodology that will be used for the final analysis of the phase 1/2 clinical trials of an inactivated novel coronavirus vaccine (Vero cell) in healthy children aged 3-17 years old (sponsor: Wuhan Institute of Biological Products Co., Ltd, Protocol number: WIBP2020001SQ). It is based on the protocol version 3.0 dated 20 May 2020. In case of differences in terms of descriptions or explanations between the SAP and the clinical trial protocol, the SAP will supersede the protocol.

This statistical analysis plan includes an overview of the trial design (related to statistics), definition and measurement of the endpoints, definition of data sets for analysis, definition of missing data and outliers, statistical analysis methods, statistical software, references and so on.

3. Study description

3.1 Study objective

Major objective

To evaluate the safety of an inactivated vaccine (Vero cells) for the COVID-19 with different doses and different immune procedures in healthy children aged 3-17 years.

Secondary objective

To investigate the immunogenicity and safety of an inactivated vaccine (Vero cells) for the COVID-19 with different doses and different immune procedures in healthy children aged 3-17 years.

3.2 Study design

Randomized, double-blind, placebo-controlled phase 1/2 trials.

In the phase 1/2 clinical trials, participants are divided into three age groups (i.e. 13-17-year-old group ,6-12-year-old group and 3-5-year-old group), each age group is further divided into three dose groups, i.e. low-dose, medium-dose, and high-dose groups. In the phase 1 trial, 3 doses of vaccine or placebo are administered to participants on day 0, day 28, and day 56, and the number of participants in each dose group is three times as many as the number of participants in the placebo group (3:1 ratio). In the phase 2 trial, three doses of vaccine (in low, medium, and high doses, respectively) or placebo are administered to participants on day 0, day 28, and day 56 with increased sample sizes.

The vaccines are administered to the low-dose group first, followed by the medium-dose and high-dose group. Among all participants in the phase 1 trial, laboratory routine tests are conducted before each injection and on the 4th day after the injection. After the 7-day post-injection safety assessment on the 8th day after the first dose, the next higher dose in phase 1 trial as well as the corresponding dose group in phase 2 trial could be injected if the preliminary assessment of safety meets the criteria of the protocol. Given the urgency, the phase 2 trial can be timely carried out according to the real situation at the clinical site.

3.3 Sample size

According to the *Technique Guideline for Clinical Trials of Vaccines* issued by the China FDA, the sample size of each vaccine dose group is about 20-30 participants for the phase 1 trial. In phase 1 clinical trial, participants are respectively enrolled in the low-dose, medium-dose, and high-dose groups, 36 participants are enrolled in the placebo control group in each of the dose

groups thus there are a total of 36 participants in the placebo group in the cohorts aged 6-17 years. 24 participants are respectively enrolled in the low-dose, medium-dose, and high-dose groups, and eight participants are enrolled in the placebo control group in each of the dose groups thus there are a total of 24 participants in the placebo group in the cohorts aged 3-5 years. The detailed sample size is shown in the Table 1.

Table 1. Sample size and procedure of the phase 1 trial

Age group	Group	Dose	N	Timing of injection
6-17	A1	Low dose	36	Days 0, 28, 56
	A2	Placebo	12	
	A3	Medium dose	36	
	A4	Placebo	12	
	A5	High dose	36	
	A6	Placebo	12	
3-5	E1	Low dose	24	
	E2	Placebo	8	
	E3	Medium dose	24	
	E4	Placebo	8	
	E5	High dose	24	
	E6	Placebo	8	

In the phase 2 trial, three doses of vaccine (in low, medium, and high doses, respectively)

or placebo are administered to participants on days 0, 28, and 56 with increased sample sizes (60 participants in the vaccine and 20 participants in the placebo in the cohorts aged 6-17 years. 84 participants in the vaccine and 28 participants in the placebo in the cohorts aged 3-5 years). The detailed sample size in the phase 2 trial is shown in the Table 2.

Table 2. Sample size and procedure of the phase 2 trial

Age group	Group	Dose	N	Timing of injection
6-17	A1	Low dose	84	Days 0, 28, and 56
	A2	Placebo	28	
	A3	Medium dose	84	
	A4	Placebo	28	
	A5	High dose	84	
	A6	Placebo	28	
3-5	E1	Low dose	60	Days 0, 28, and 56
	E2	Placebo	20	
	E3	Medium dose	60	
	E4	Placebo	20	
	E5	High dose	60	
	E6	Placebo	20	

3.4 Inclusion and Exclusion criteria

Inclusion criteria

— Age range: healthy children aged 3-17 years

- General good health as established by medical history and physical examination;
- Since December 2019, the participant has not gone to Hubei province, overseas, or to a village/community where there had been COVID-19 cases, has not contacted with confirmed or suspected cases, are not in the quarantine period, and are not from a village/community where there were confirmed or suspected cases.
- Women who have reached menarche are not pregnant (negative urine pregnancy test), are not breastfeeding, do not have pregnancy plan within the three months after enrollment, and have already taken effective contraceptive measures two weeks before enrollment;
- For potential participants under 18 years of age, a parent/legal guardian (referred to subsequently as “parent”) are able and willing to complete the whole research procedure in about 14 months;
- For potential participants under 18 years of age, a parent/legal guardian (referred to subsequently as “parent”) have the ability to understand the research procedures, to sign the informed consent voluntarily after explanation, and can comply with the requirements of the clinical research program.

Exclusion criteria for the first dose

- Confirmed, suspected, or asymptomatic COVID-19 cases;
- Those with positive antibody tests of the COVID-19;
- History of SARS virus infection (identified through self-report or on-site inquiry)
- Those with fever (axillary temperature >37.0 ℃), dry cough, fatigue, nasal obstruction, runny nose, sore throat, myalgia, diarrhea, shortness of breath, and dyspnea within 14 days before

injection;

- Those with clinically abnormal parameters from blood biochemical, blood routine, and urine routine before injection (only for stage I clinical trial);
- Axillary temperature >37.0 ℃ before injection;
- Those who have experienced severe allergic reactions (such as acute anaphylaxis, urticaria, eczema, dyspnea, neurovascular edema, or abdominal pain), or those who are allergic to the known gradients of COVID-19 inactivated vaccine;
- Those with history or family history of convulsion, epilepsy, encephalopathy, or mental illness;
- Those with congenital malformation, developmental disorder, genetic defect, or severe malnutrition, etc.;
- Those with severe hepatorenal diseases, uncontrolled hypertension (systolic blood pressure ≥140 mmHg, diastolic blood pressure ≥90 mmHg), diabetes complications, malignant tumors, or various acute or chronic diseases (acute attack stage);
- Those diagnosed with congenital or acquired immunodeficiency, HIV infection, lymphoma, leukemia, or other autoimmune diseases;
- Those with confirmed or suspected serious respiratory diseases, serious cardiovascular diseases, hepatorenal diseases, and malignant tumors;
- Those with a history of abnormal coagulation (such as lack of coagulation factors or coagulation diseases);
- Those receiving anti-TB treatment;
- Those receiving immunoenhancement or inhibitor treatment (p.o. or gtt.) over 14 days within 3 months (continuous oral or infusion for more than 14 days);

- Those receiving live attenuated vaccines within one month before injection or other vaccines within 14 days before injection;
- Those receiving blood products within 3 months before injection;
- Those receiving other study drugs within 6 months before injection;
- Those under other conditions not suitable for the clinical trial (evaluated by researchers)

Exclusion criteria for second and third doses

- Women who have reached menarche with positive urine pregnancy tests;
- Those with high fever (axillary temperature ≥39.0 ℃) lasting for three days or severe allergic reaction after the previous injection;
- Serious adverse reactions related to the previous injection;
- If inconformity with inclusion criteria or conformity with exclusion criteria for the first dose occurs or is newly found after the previous injection , researchers should decide whether the participants could continue to participate in the study;
- Other reasons for exclusion evaluated by researchers.

Participants are not required to complete the trial if

- non-specific immunoglobulins are used during the study;
- steroid hormones have been given orally or intravenously for 14 days.

4. Endpoints

4.1 Endpoints of the phase 1 trial

Safety endpoints:

- The primary safety endpoint is the incidence of adverse reactions observed within 7 days after each injection;

The secondary safety endpoints include:

- Incidence of any adverse reactions/events within 30 minutes after each injection;
- Incidence of abnormal hepatorenal function and abnormal parameters of blood and urine routine on the 4th day after each injection;
- Lymphocyte subset distribution and cytokines before each injection, on day 14 after the first and second injection, and on days 28, 180, and 360 after the whole-course immunization.
- Incidence of adverse events observed from day 8 to 28/30, and from days 0 to 28/30 after each injection;
- Incidence of serious adverse events (SAE) within 12 months.

Humoral immunogenicity endpoints:

The neutralizing antibody amounts against live SARS-CoV-2 virus and specific ELISA antibody titers to whole SARS-CoV-2 virus.

- Seroconversion and antibody titers (GMT, GMI) before each injection, on days 4 and 14 (except for the third injection among those aged 3-17 years) after each injection on days 28, 90, 180, and 360 after the whole-course immunization. A positive antibody response (seroconversion) is defined as at least a four-fold increase in post-injection titer from baseline.

4.2 Endpoints of the phase 2 trial

Safety endpoints:

- The primary safety endpoint is the incidence of adverse reactions observed within 7 days after each injection;

The secondary safety endpoints include:

- Incidence of any adverse reactions/events within 30 minutes after each injection;
- Incidence of adverse events observed from day 8 to 14/21/28/30, and from days 0 to 14/21/28/30 after each injection;
- Incidence of serious adverse events (SAE) within 12 months.

Humoral immunogenicity endpoints:

- Antibody titers (GMT, GMI) and seroconversion before each injection, and on days 28, 90, 180, and 360 after the whole-course immunization in all groups.

For uncertain values: when calculating GMT, GMI and seroconversion of antibodies, if the antibody level is below the lower detection limit, the lower detection limit will be taken as the value; if the antibody level is greater than the maximum detection limit, the maximum dilution will be taken as the value.

5. Statistical analysis population

Participants who were not eligible for randomization but who have been erroneously randomized into the study will be excluded from all analysis populations.

5.1 Selection of datasets for analysis before full unblinding (for safety analysis and immunogenicity analysis on day 28 after the whole-course immunization)

Dataset for safety evaluation (SS): All participants who have received at least one vaccine and have at least one data point of safety evaluation should be included in the dataset for safety evaluation. Participants who violate the protocol should not be excluded.

Dataset for full analysis (FAS): The dataset for full analysis is set according to the theory of

intention-to-treat (ITT) analysis and includes an ideal population. All participants who have met the inclusion criteria but not the exclusion criteria, participated in randomization, received at least one experimental vaccine, and had results of any blood biomarker measurements before immunization or between first injection and day 28 after the whole-course immunization should be included in the FAS for immunogenicity analysis.

Per-protocol set (PPS): PPS is a subset of FAS. The participants in this dataset are more compliant with the protocol, do not take any banned drugs during the study period, do not severely violate the protocol and inclusion criteria, are vaccinated according to the protocol, and have results of any biomarker measurements before immunization or between first injection and day 28 after the whole-course immunization.

① Phase 1 clinical trial

For the subset of PPS on the 4th, 14th after the first injection as well as before the second injection, participants should be included in PPS if they have finished the first injection, do not take banned drugs between the first two vaccinations, do not greatly violate the protocol, and meet the inclusion criteria.

For the subset of PPS on the 4th and 14th days after the second injection as well as before the third injection (or on day 28 after the second injection for the two-dose group), participants should be included in PPS if they have finished the second injection, do not take banned drugs between the first two injections, do not greatly violate the protocol, and meet the inclusion criteria.

For the subset of PPS on the 4th, 14th, and 28th days after the third injection, participants should be included in PPS if they have finished the third injection, do not take banned drugs between the first two vaccinations, do not greatly violate the protocol, and meet the inclusion criteria.

② Phase 2 clinical trial

The principle of PPS is similar to that of phase 1 clinical trial, except that the 4th, 14th after each injection are exempted.

The following participants are excluded from the PPS: those not meeting the inclusion criteria; those meeting the exclusion criteria; those with no or severely missing follow-up data or information after vaccination; those meeting the termination or withdrawal criteria but remaining in the trial; those receiving wrong vaccination or dose; those who are judged by the researchers to be excluded.

(3) **Selection of analysis data set after unblinding at the end of the trial (for immune durability)**

Dataset for full analysis (FAS): All participants who meet the inclusion criteria but not the exclusion criteria, participate in randomization, receive experimental vaccines, and have results of any blood biomarker measurements on days 90, 180, and 360 after the whole-course immunization.

Per-protocol set (PPS): PPS is a subset of FAS. The participants in this dataset are more compliant with the protocol, do not take any banned drugs during the study period, do not severely violate the protocol and inclusion criteria, are vaccinated according to the protocol, and have results of any biomarker measurements before immunization or on days 90, 180, and 360 after the whole-course immunization.

The following participants are excluded from the PPS: those not meeting the inclusion criteria; those meeting the exclusion criteria; those with no or severely missing follow-up data or information after vaccination; those meeting the termination or withdrawal criteria but remaining in the trial; those receiving wrong vaccination or dose; those who are judged by the researchers to be excluded.

FAS is used as the main dataset when analyzing humoral immune parameters and laboratory test results. However, PPS is also used for analysis, and any different results from PPS and FAS

would be discussed in the report.

6. Statistical methods

6.1 General principles

In this study, the statistical analysis will be completed by SPSS 25.0 ®software. The statistical analysis plan is formulated by biostatisticians and key researchers according to the research plan, and the final document is refined and formed before the data is locked.

- Continuous variables are described by mean, standard deviation, median, quartile, minimum, maximum, and coefficient of variation. The categorical and rating data are described by frequency and percentage.
- The continuous variables are analyzed by t-test and analysis of variance, and the categorical variables are analyzed by χ^2 test, modified χ^2 test, or Fisher's exact test. The rank sum test is used for the data of unknown or non-normal distribution.
- Statistical significance is considered if $P \leq 0.05$ when the two-sided test level is $\alpha = 0.05$.
- In the PPS analysis, missing values during the follow-up were imputed using the last observation carried forward method.

6.2 Trial completion assessment

- The numbers and reasons of people who participate in the trial, complete the trial, drop out of trial, and violate/deviate the protocol;
- Statistics on the number of people included in each dataset and a detailed list of datasets.
- Draw the flow chart of participant selection and distribution.
- The proportions of use of concomitant drugs, and use of other vaccines in vaccine and

placebo groups are described. The χ^2 test, modified χ^2 test, or Fisher's exact test is used to compare the proportion of use of concomitant drugs between groups.

- Baseline demographic characteristics: sex composition and age distribution. The χ^2 test is used to compare the sex composition in the vaccine and placebo groups. Two independent samples t-test is used to compare the mean age between each vaccine and placebo group, and ANOVA is used to compare different groups.

6.3 Safety evaluations

Safety evaluation mainly includes the total incidence of adverse reactions and the incidence of topical and systemic adverse reactions after each injection. Safety evaluation is based on SS dataset. Parameters used for safety evaluation in immune-procedure-specific groups are analyzed in phase 1 and 2 trials separately, which are further merged for analysis.

Adverse event (0-7 and 0-28/30 days) in the vaccine group and placebo group:

- The total incidence of adverse reactions and events;
- Adverse reactions after each injection;
- The incidence of topical or systemic adverse reactions;
- The incidence of abnormal laboratory parameters;
- The composition of adverse reactions of different levels.

Adverse reaction symptoms in the vaccine group and placebo group:

- The incidence of symptom-specific adverse reactions and composition of adverse reactions of different levels;

Events causing withdrawal and SAE:

- Withdrawal due to adverse events and/or serious SAE cases should be specially noted,

and the incidence of these events should be compared between groups.

The χ^2 test, modified χ^2 test, or Fisher's exact test is used to compare the incidence of adverse events, adverse events of level 3, and SAE between vaccine and placebo groups, as well as between different dose groups. Rank sum test is used to compare the mean level of the adverse events between vaccine and placebo groups.

6.4 Cellular immune safety analysis in the phase 1 trial

FAS and PPS datasets are used, and all values were logarithmically transformed before analysis.

Analysis of cytokines: ① Two independent samples t-test (when meeting normal distribution and homogeneity of variance) or modified t-tests (when meeting normal distribution but violating homogeneity of variance) is used to compare the differences of mean levels of cytokines, including IL-2, IL-4, IL-6, IL-10, TNF-α, and IFN-γ, etc., before the first injection and each time point after the first injection between vaccine and placebo groups, and ANOVA is used to compare among different vaccine groups. ② Analysis of variance of repeated measurement data is used to explore the difference of mean levels of cytokines in three dose groups on each time point after injection.

Analysis of lymphocyte count: ① Two independent samples t-test (when meeting normal distribution and homogeneity of variance) or modified t-tests (when meeting normal distribution but violating homogeneity of variance) is used to compare the differences of lymphocyte count between vaccine and placebo groups before the first injection and on different time points after injection, and ANOVA is used to compare among different vaccine groups. ② Analysis of variance of repeated measurement data is used to explore the differences of lymphocyte count in three dose groups on each time point after injection.

6.5 Humoral immunogenicity assessment

FAS and PPS datasets are respectively used for analyses. For groups vaccinated on days 0, 28, and 56 in phase 1 and 2 clinical trials, blood samples are collected before each injection and on day 28 after whole-course immunization, thus we could merge the antibody measures of the same time point in the phase 1 and 2 trials. Blood samples are also collected on days 4 and 14 (except for the third injection among those aged 3-17 years) after each injection in the phase 1 trial among those aged 3-17 years, thus the results are also reported for these time points.

Statistical description: The antibody titer should be logarithmically transformed, and the minimum, maximum, median, interquartile range, and GMT (95% confidence interval) would be calculated.

Comparison of serum antibody GMT before each injection: Antibody titer is logarithmically transformed, and two independent samples t-test (when meeting normal distribution and homogeneity of variance) or modified t-test (when meeting normal distribution but violating homogeneity of variance) is used to compare serum antibody GMT before vaccination between vaccine and placebo groups, and ANOVA is used for comparison among different dose groups. χ^2 test, modified χ^2 test, or Fisher's exact test is used to compare the seroconversion rate among vaccine and placebo groups, as well as different dose groups.

Comparison of antibodies at each time point after injection: ① antibody titers are logarithmically transformed. For those receiving injections on days 0, 28, and 56, two independent samples t-test (when meeting normal distribution and homogeneity of variance) or modified t-test (when meeting normal distribution but violating homogeneity of variance) is used to compare the antibody GMT after each vaccination between vaccine and placebo groups, and ANOVA is used for

comparison among different dose groups. ② χ^2 test, modified χ^2 test, or Fisher's exact test is used to compare the seroconversion rate among vaccine and placebo groups, as well as different dose groups. ③ The antibody GMI and its 95% confidence interval are calculated in each group. ④ Analysis of variance of repeated measurement data is used to examine the differences of antibody GMT at different time points in different groups.

7. Partial unblinding and interim analysis

Considering that the preclinical animal safety evaluation is still unfinished, current animal evidence only supports two-dose immune procedures in clinical trials. Whether three-dose immune procedures could be used in the clinical trial needs subsequent results of animal studies. Therefore, partial unblinding and interim analysis are planned for the phase 1 and 2 trials after getting the results of immunogenicity test on day 14 after the second injection and safety evaluation after 28 days from the second injection. In the phase 2 trial, partial unblinding and interim analysis are planned for the two-dose schedule groups using the medium dose after getting the results of immunogenicity test on day 28 after the second injection and safety evaluation after 28 days from the second injection. Main safety and immunogenicity evaluations should be conducted in these groups after unblinding.

Considering the urgent COVID-19 epidemic, partial unblinding could be conducted in certain dose groups. Whether to carry out partial unblinding in certain groups and the frequency of partial unblinding in certain groups should be decided according to the time when antibody test results are obtained. Unblinding could be conducted after inspection of blinding and data cleaning. Unblinding is carried out jointly by the sponsor, the main investigator, statisticians, DSMB

members, and data managers, etc. All personnel involved in unblinding should sign a confidentiality agreement, and researchers in the field are still blinding and should conduct immunogenicity test and safety evaluation after 12 months from the end of the whole-course immunization.

8. Interim analysis added on June 14, 2020

Considering the urgent COVID-19 epidemic, partial unblinding is added to the protocol on June 14. The data are available for humoral immunogenicity tests on day 14 after the second inoculation in the phase 1 and 2 trials. Partial unblinding in those groups can be carried out after inspection and data cleaning. The reasons for unblinding those groups are mainly because results from these groups could provide basic information about safety and immunogenicity of the inactivated vaccine for 3 different doses (100, 200 and 400 WU/dose). The information will be used for the decision of continuing the current trial and design for the phase 3 trial.
