# Supplementary material for: Safety and Immunogenicity of an Inactivated COVID-19 Vaccine, WIBP-CorV, in Healthy Children: Interim Analysis of a Randomized, Double-Blind, Controlled, Phase 1/2 Trial
Source: Front Immunol. 2022 Jun 24;13:898151. doi: 10.3389/fimmu.2022.898151 (PMC9265248; doi:10.3389/fimmu.2022.898151)
Supplement: Supplementary file 3 [file DataSheet_3.pdf]

Supplemental Online Content

Supplement to: Guo W, Duan K, Zhang Y, et al. Safety and immunogenicity of an inactivated COVID-19 vaccine, WIBP-CorV, in healthy children: interim analysis of 2 randomized clinical trials up to 180 days after full vaccination.

Supplement 3. Appendix pages

Appendix 1. Incidence of adverse events within 0-7 days (including 30 minutes) of the full vaccination in subjects aged 3-17 years in phase I clinical trial......................................1

Appendix 2. Incidence of adverse events within 30 days of the full vaccination in subjects aged 3-17 years in phase I clinical trial...2

Appendix 3. Incidence of adverse reactions within 0-7 days (including 30 minutes) of the whole vaccination for subjects aged 3-17 years in phase I clinical trial................................3

Appendix 4. Severity of adverse reactions of subjects aged 3-17 years in phase I clinical trial after the full vaccination (based on cases)..6

Appendix 5. Incidence of serious adverse events in subjects aged 3-17 years in phase I clinical trial..9

Appendix 6. Antibody GMT (95% CI) of phase I subjects aged 3-17 years at different time points（FAS） 12

Appendix 7. Antibody GMT (95% CI) of phase I subjects aged 3-17 years at different time points（PPS） 14

Appendix 8. Comparison of antibody levels of phase I subjects aged 3-17 years 28 days after the full vaccination（FAS）...16

Appendix 9. Comparison of antibody levels of phase I subjects aged 3-17 years 28 days after the full vaccination（PPS）...20

Appendix 10. Quadruple growth rate of antibody at different time points in subjects aged 3-17 years in phase I (95% CI)（PPS）..22

Appendix 11. Quadruple growth rate of antibody at different time points in subjects aged 3-17 years in phase I (95% CI)（FAS）..23

Appendix 12. Incidence of adverse events within 0-7 days (including 30 minutes) after the full vaccination in subjects aged 3-17 years years in phase II...24

Appendix 13. Incidence of adverse events within 30 days after whole course exemption in subjects aged 3-17 years in phase II...25

Appendix 14. Incidence of adverse reactions within 0-7 days (including 30 minutes) after the full vaccination in subjects aged 3-17 years in phase II clinical trial............................26

Appendix 15. Severity of adverse reactions of phase II subjects aged 3-17 years after whole course vaccination (based on cases)..28

Appendix 16. Incidence of serious adverse events in subjects aged 3-17 years years in phase II clinical trial..32

Appendix 17. Antibody GMT (95% CI) of subjects aged 3-17 years at different time points in phase II clinical trial（FAS）..37

Appendix 18. Antibody GMT (95% CI) of phase II subjects aged 3-17 years at different time points（PPS）..39

Appendix 19. Comparison of antibody levels of phase II subjects aged 3-17 years 28 days after the full vaccination（FAS）...42

Appendix 20. Comparison of antibody levels of subjects aged 3-17 years in phase II clinical trial 28 days after the full vaccination（PPS）...47

Appendix 21. Quadruple growth rate of antibody in subjects aged 3-17 years in phase II at different time points (95% CI)（FAS）...49

Appendix 22. Quadruple growth rate of antibody in subjects aged 3-17 years in phase II clinical trial at different time points (95% CI)（PPS）..50

Appendix 23. Incidence of adverse events within 30 days after phase I and II combined whole course vaccination for 3-17 years old...52

Appendix 24. Incidence of adverse reactions within 30 days after phase I and II combined whole course vaccination for 3-17 years old..54

Appendix 25. Antibody GMT of phase I and II subjects aged 3-17 years before the first dose of vaccination to 28 days after the third dose of vaccination（FAS）................52

Appendix 26. Antibody GMT of phase I and II subjects aged 3-17 years from before the first dose of vaccination to 28 days after the third dose of vaccination（PPS）................54

Appendix 27. Comparison of antibody levels of phase I and phase II subjects aged 3-17 years after the full vaccination（FAS）...56

Appendix 28. Comparison of antibody levels of phase I and phase II subjects aged 3-17 years after the full vaccination（PPS）...60

Appendix 29. Quadruple growth rate of antibody in phase I and phase II clinical trials combined with subjects aged 3-17 years from before the first dose of vaccination to 28 days after the third dose of vaccination（FAS）..63

Appendix 30. Quadruple growth rate of antibody in phase I and phase II clinical trials combined with subjects aged 3-17 years from before the first dose of vaccination to 28 days after the third dose of vaccination（PPS）..65

Appendix 31. The change trend of GMT of neutralizing antibody in phase I clinical trial at 90 days after the full vaccination（PPS）..67

Appendix 32. The change trend of GMT of neutralizing antibody in phase I clinical trial at 90 days after the full vaccination (FAS) 68

Appendix 33. Quadruple growth rate of neutralizing antibody in phase I clinical trial at 90 days after the third dose of vaccination（%）(PPS) 69

Appendix 34. Quadruple growth rate of neutralizing antibody in phase I clinical trial at 90 days after the third dose of vaccination（%）(FAS) 70

Appendix 35. The change trend of GMT of specific antibody in phase I clinical trial at 90 days after the full vaccination (PPS) 71

Appendix 36. The change trend of GMT of specific antibody in phase Iclinical trial at 90 days after the full vaccination (FAS) 72

Appendix 37. Quadruple growth rate of specific antibody in phase I clinical trial at 90 days after the third dose of vaccination（%）(PPS) 73

Appendix 38. Quadruple growth rate of specific antibody in phase I clinical trial at 90 days after the third dose of vaccination（%）(FAS) 74

Appendix 39. The change trend of GMT of neutralizing antibody in phase II clinical trial at 90 days after the full vaccination（PPS）..75

Appendix 40. The change trend of GMT of neutralizing antibody in phase II clinical trial at 90 days after the full vaccination (FAS) 76

Appendix 41. Quadruple growth rate of neutralizing antibody in phase II clinical trial at 90 days after the third dose of vaccination（%）(PPS) ……..77

Appendix 42. Quadruple growth rate of neutralizing antibody in phase II clinical trial at 90 days after the third dose of vaccination（%）(FAS) 78

Appendix 43. The change trend of GMT of specific antibody in phase II clinical trial at 90 days after the full vaccination (PPS) 79

Appendix 44. The change trend of GMT of specific antibody in phase II clinical trial at 90 days after the full vaccination (FAS) 80
Appendix 45. Quadruple growth rate of specific antibody in phase II clinical trial at 90 days after the third dose of vaccination（%）(PPS) 81
Appendix 46. Quadruple growth rate of specific antibody in phase II clinical trial at 90 days after the third dose of vaccination（%）(FAS) 82
Appendix 47. The change trend of GMT of neutralizing antibody in phase I and phase II clinical trial at 90 days after the full vaccination（PPS） 83
Appendix 48. The change trend of GMT of neutralizing antibody in phase I and phase II clinical trial at 90 days after the full vaccination (FAS) 84
Appendix 49. Quadruple growth rate of neutralizing antibody in phase I and phase II clinical trial at 90 days after the third dose of vaccination（%）(PPS) 85
Appendix 50. Quadruple growth rate of neutralizing antibody in phase I and phase II clinical trial at 90 days after the third dose of vaccination（%）(FAS) 86
Appendix 51. The change trend of GMT of specific antibody in phase I and phase II clinical trial at 90 days after the full vaccination (PPS) 87
Appendix 52. The change trend of GMT of specific antibody in phase I and phase II clinical trial at 90 days after the full vaccination (FAS) 88
Appendix 53. Quadruple growth rate of specific antibody in phase I and phase II clinical trial at 90 days after the third dose of vaccination（%）(PPS) 89
Appendix 54. Quadruple growth rate of specific antibody in phase I and phase II clinical trial at 190 days after the third dose of immunization（%）(FAS) 90
Appendix 55. The change trend of GMT of neutralizing antibody in phase I clinical trial at 180 days after the full vaccination（PPS） 91
Appendix 56. The change trend of GMT of neutralizing antibody in phase I clinical trial at 180 days after the full vaccination (FAS) 92
Appendix 57. Quadruple growth rate of neutralizing antibody in phase I clinical trial at 180 days after the third dose of vaccination（%）(PPS) 93
Appendix 58. Quadruple growth rate of neutralizing antibody in phase I clinical trial at 180 days after the third dose of vaccination（%）(FAS) 94

Appendix 59. The change trend of GMT of specific antibody in phase I clinical trial at 180 days after the full vaccination (PPS) 95

Appendix 60. The change trend of GMT of specific antibody in phase Iclinical trial at 180 days after the full vaccination (FAS) 96

Appendix 61. Quadruple growth rate of specific antibody in phase I clinical trial at 180 days after the third dose of vaccination（%）(PPS) 97

Appendix 62. Quadruple growth rate of specific antibody in phase I clinical trial at 180 days after the third dose of vaccination（%）(FAS) 98

Appendix 63. The change trend of GMT of neutralizing antibody in phase II clinical trial at 180 days after the full vaccination（PPS） 99

Appendix 64. The change trend of GMT of neutralizing antibody in phase II clinical trial at 180 days after the full vaccination (FAS) 100

Appendix 65. Quadruple growth rate of neutralizing antibody in phase II clinical trial at 180 days after the third dose of vaccination （%）(PPS) 101

Appendix 66. Quadruple growth rate of neutralizing antibody in phase II clinical trial at 180 days after the third dose of vaccination（%）(FAS) 102

Appendix 67. The change trend of GMT of specific antibody in phase II clinical trial at 180 days after the full vaccination (PPS) 103

Appendix 68. The change trend of GMT of specific antibody in phase II clinical trial at 180 days after the full vaccination (FAS) 104

Appendix 69. Quadruple growth rate of specific antibody in phase II clinical trial at 180 days after the third dose of vaccination（%）(PPS) 105

Appendix 70. Quadruple growth rate of specific antibody in phase II clinical trial at 180 days after the third dose of vaccination（%）(FAS) 106

Appendix 71. The change trend of GMT of neutralizing antibody in phase I and phase II clinical trial at 180 days after the full vaccination（PPS） 107

Appendix 72. The change trend of GMT of neutralizing antibody in phase I and phase II clinical trial at 180 days after the full vaccination (FAS) 108

Appendix 73. Quadruple growth rate of neutralizing antibody in phase I and phase II clinical trial at 180 days after the third dose of vaccination（%）(PPS) 109

Appendix 74. Quadruple growth rate of neutralizing antibody in phase I and phase II clinical trial at 180 days after the third dose of vaccination（%）(FAS) 110

Appendix 75. The change trend of GMT of specific antibody in phase I and phase II clinical trial at 180 days after the full vaccination (PPS) 111

Appendix 76. The change trend of GMT of specific antibody in phase I and phase II clinical trial at 180 days after the full vaccination (FAS) 112

Appendix 77. Quadruple growth rate of specific antibody in phase I and phase II clinical trial at 180 days after the third dose of vaccination（%）(PPS) 113

Appendix 78. Quadruple growth rate of specific antibody in phase I and phase II clinical trial at 1180 days after the third dose of vaccination（%）(FAS) 114

Appendix 79. Comparison of Neutralizing Antibody for Female and Male in Different Treatment Groups..115

Appendix 1. Incidence of adverse events within 0-7 days (including 30 minutes) of the full vaccination in subjects aged 3-17 years in phase I clinical trial

		Low dose				Medium dose				High dose				Placebo				*P*
		Case times	Number of cases	Rate(%)	95%CI	Case times	Number of cases	Rate(%)	95%CI	Case times	Number of cases	Rate(%)	95%CI	Case times	Number of cases	Rate(%)	95%CI	
Aged 13-17 (N=18)	Total adverse events	8	7	38.9	17.3~64.3	2	2	11.1	1.4~34.7	4	4	22.2	6.4~47.6	9	6	33.3	13.3~59.0	0.239
	Systemic adverse events	2	2	11.1	1.4~34.7	2	2	11.1	1.4~34.7	2	2	11.1	1.4~34.7	1	1	5.6	0.1~27.3	0.924
	Local adverse events	6	6	33.3	13.3~59.0	0	0	0.0	0.0~18.5	1	1	5.6	0.1~27.3	8	6	33.3	13.3~59.0	0.009
	Other adverse events	0	0	0.0	0.0~18.5	0	0	0.0	0.0~18.5	1	1	5.6	0.1~27.3	0	0	0.0	0.0~18.5	>0.999
Aged 6-12 (N=18)	Total adverse events	6	3	16.7	3.6~41.4	8	6	33.3	13.3~59.0	4	1	5.6	0.1~27.3	10	7	38.9	17.3~64.3	0.072
	Systemic adverse events	1	1	5.6	0.1~27.3	1	1	5.6	0.1~27.3	1	1	5.6	0.1~27.3	2	2	11.1	1.4~34.7	0.886
	Local adverse events	2	2	11.1	1.4~34.7	7	5	27.8	9.7~53.5	1	1	5.6	0.1~27.3	5	4	22.2	6.4~47.6	0.261
	Other adverse events	3	1	5.6	0.1~27.3	0	0	0.0	0.0~18.5	2	1	5.6	0.1~27.3	3	2	11.1	1.4~34.7	0.548
Aged 3-5 (N=24)	Total adverse events	14	10	41.7	22.1~63.4	10	9	37.5	18.8~59.4	14	8	33.3	15.6~55.3	9	7	29.2	12.6~51.1	0.823
	Systemic adverse events	4	4	16.7	4.7~37.4	6	5	20.8	7.1~42.2	11	6	25.0	9.8~46.7	4	3	12.5	2.7~32.4	0.713
	Local adverse events	6	5	20.8	7.1~42.2	1	1	4.2	0.1~21.1	1	1	4.2	0.1~21.1	4	4	16.7	4.7~37.4	0.155
	Other adverse events	4	3	12.5	2.7~32.4	3	3	12.5	2.7~32.4	2	2	8.3	1.0~27.0	1	1	4.2	0.1~21.1	0.718
	Level 3 adverse events	0	0	0.0	0.0~14.2	0	0	0.0	0.0~14.2	1	1	4.2	0.1~21.1	0	0	0.0	0.0~14.2	>0.999
Aged 3-17 (N=60)	Total adverse events	28	20	33.3	21.7~46.7	20	17	28.3	17.5~41.4	22	13	21.7	12.1~34.2	28	20	33.3	21.7~46.7	0.447
	Systemic adverse events	7	7	11.7	4.8~22.6	9	8	13.3	5.9~24.6	14	9	15.0	7.1~26.6	7	6	10.0	3.8~20.5	0.859
	Local adverse events	14	13	21.7	12.1~34.2	8	6	10.0	3.8~20.5	3	3	5.0	1.0~13.9	17	14	23.3	13.4~36.0	0.010
	Other adverse events	7	4	6.7	1.8~16.2	3	3	5.0	1.0~13.9	5	4	6.7	1.8~16.2	4	3	5.0	1.0~13.9	0.959
	Level 3 adverse events	0	0	0.0	0.0~6.0	0	0	0.0	0.0~6.0	1	1	1.7	0.1~8.9	0	0	0.0	0.0~6.0	>0.999

Note: ① total adverse events include adverse events at inoculation site (local), adverse events at non inoculation site (whole body) and other adverse events. The low, medium, and high doses represent 2.5μg, 5.0μg, and 10.0 μg/dose, respectively.

Appendix 2. Incidence of adverse events within 30 days of the full vaccination in subjects aged 3-17 years in phase I clinical trial

		Low dose				Medium dose				High dose				Placebo				*P*
		Case times	Number of cases	Rate(%)	95%CI	Case times	Number of cases	Rate(%)	95%CI	Case times	Number of cases	Rate(%)	95%CI	Case times	Number of cases	Rate(%)	95%CI	
Aged 13-17 (N=18)	Total adverse events	11	7	38.9	17.3~64.3	5	3	16.7	3.6~41.4	8	7	38.9	17.3~64.3	10	7	38.9	17.3~64.3	0.392
	Systemic adverse events	2	2	11.1	1.4~34.7	2	2	11.1	1.4~34.7	3	3	16.7	3.6~41.4	1	1	5.6	0.1~27.3	0.771
	Local adverse events	6	6	33.3	13.3~59.0	0	0	0.0	0.0~18.5	1	1	5.6	0.1~27.3	8	6	33.3	13.3~59.0	0.009
	Other adverse events	3	1	5.6	0.1~27.3	3	3	16.7	3.6~41.4	4	3	16.7	3.6~41.4	1	1	5.6	0.1~27.3	0.522
Aged 6-12 (N=18)	Total adverse events	12	5	27.8	9.7~53.5	13	7	38.9	17.3~64.3	5	2	11.1	1.4~34.7	18	9	50.0	26.0~74.0	0.077
	Systemic adverse events	2	2	11.1	1.4~34.7	3	3	16.7	3.6~41.4	2	2	11.1	1.4~34.7	5	4	22.2	6.4~47.6	0.758
	Local adverse events	2	2	11.1	1.4~34.7	7	5	27.8	9.7~53.5	1	1	5.6	0.1~27.3	5	4	22.2	6.4~47.6	0.261
	Other adverse events	8	3	16.7	3.6~41.4	3	1	5.6	0.1~27.3	2	1	5.6	0.1~27.3	8	4	22.2	6.4~47.6	0.330
	Level 3 adverse events	1	1	5.6	0.1~27.3	0	0	0.0	0.0~18.5	0	0	0.0	0.0~18.5	0	0	0.0	0.0~18.5	>0.999
Aged 3-5 (N=24)	Total adverse events	20	14	58.3	36.6~77.9	28	14	58.3	36.6~77.9	23	12	50.0	29.1~70.9	23	16	66.7	44.7~84.4	0.712
	Systemic adverse events	7	6	25.0	9.8~46.7	19	10	41.7	22.1~63.4	18	10	41.7	22.1~63.4	15	11	45.8	25.6~67.2	0.458
	Local adverse events	6	5	20.8	7.1~42.2	1	1	4.2	0.1~21.1	1	1	4.2	0.1~21.1	4	4	16.7	4.7~37.4	0.155
	Other adverse events	7	6	25.0	9.8~46.7	8	5	20.8	7.1~42.2	4	4	16.7	4.7~37.4	4	3	12.5	2.7~32.4	0.713
	Level 3 adverse events	0	0	0.0	0.0~14.2	0	0	0.0	0.0~14.2	1	1	4.2	0.1~21.1	0	0	0.0	0.0~14.2	>0.999
Aged 3-17 (N=60)	Total adverse events	43	26	43.3	30.6~56.8	46	24	40.0	27.6~53.5	36	21	35.0	23.1~48.4	51	32	53.3	40.0~66.3	0.221
	Systemic adverse events	11	10	16.7	8.3~28.5	24	15	25.0	14.7~37.9	23	15	25.0	14.7~37.9	21	16	26.7	16.1~39.7	0.562
	Local adverse events	14	13	21.7	12.1~34.2	8	6	10.0	3.8~20.5	3	3	5.0	1.0~13.9	17	14	23.3	13.4~36.0	0.010
	Other adverse events	18	10	16.7	8.3~28.5	14	9	15.0	7.1~26.6	10	8	13.3	5.9~24.6	13	8	13.3	5.9~24.6	0.947
	Level 3 adverse events	1	1	1.7	0.1~8.9	0	0	0.0	0.0~6.0	1	1	1.7	0.1~8.9	0	0	0.0	0.0~6.0	>0.999

Note: ① total adverse events include adverse events at inoculation site (local), adverse events at non inoculation site (whole body) and other adverse events. The low, medium, and high doses represent 2.5μg, 5.0μg, and 10.0 μg/dose, respectively.

Appendix 3. Incidence of adverse reactions within 0-7 days (including 30 minutes) of the whole vaccination for subjects aged 3-17 years in phase I clinical trial

		Low dose				Medium dose				High dose				Placebo				*P*
		Case times	Number of cases	Rate(%)	95%CI	Case times	Number of cases	Rate(%)	95%CI	Case times	Number of cases	Rate(%)	95%CI	Case times	Number of cases	Rate(%)	95%CI	
Aged 13-17 (N=18)	Total adverse reactions	8	7	38.9	17.3~64.3	2	2	11.1	1.4~34.7	3	3	16.7	3.6~41.4	9	6	33.3	13.3~59.0	0.169
	Systemic adverse reactions	2	2	11.1	1.4~34.7	2	2	11.1	1.4~34.7	2	2	11.1	1.4~34.7	1	1	5.6	0.1~27.3	0.924
	Hypersensitivity	0	0	0.0	0.0~18.5	0	0	0.0	0.0~18.5	0	0	0.0	0.0~18.5	0	0	0.0	0.0~18.5	-
	Fever	2	2	11.1	1.4~34.7	2	2	11.1	1.4~34.7	1	1	5.6	0.1~27.3	1	1	5.6	0.1~27.3	0.867
	Diarrhea	0	0	0.0	0.0~18.5	0	0	0.0	0.0~18.5	0	0	0.0	0.0~18.5	0	0	0.0	0.0~18.5	-
	Arthralgia	0	0	0.0	0.0~18.5	0	0	0.0	0.0~18.5	1	1	5.6	0.1~27.3	0	0	0.0	0.0~18.5	>0.999
	Cough	0	0	0.0	0.0~18.5	0	0	0.0	0.0~18.5	0	0	0.0	0.0~18.5	0	0	0.0	0.0~18.5	-
	Vomit	0	0	0.0	0.0~18.5	0	0	0.0	0.0~18.5	0	0	0.0	0.0~18.5	0	0	0.0	0.0~18.5	-
	Anorexia	0	0	0.0	0.0~18.5	0	0	0.0	0.0~18.5	0	0	0.0	0.0~18.5	0	0	0.0	0.0~18.5	-
	Headache	0	0	0.0	0.0~18.5	0	0	0.0	0.0~18.5	0	0	0.0	0.0~18.5	0	0	0.0	0.0~18.5	-
	Local adverse reaction	6	6	33.3	13.3~59.0	0	0	0.0	0.0~18.5	1	1	5.6	0.1~27.3	8	6	33.3	13.3~59.0	0.009
	Erythema	0	0	0.0	0.0~18.5	0	0	0.0	0.0~18.5	0	0	0.0	0.0~18.5	1	1	5.6	0.1~27.3	>0.999
	Pain	6	6	33.3	13.3~59.0	0	0	0.0	0.0~18.5	1	1	5.6	0.1~27.3	6	5	27.8	9.7~53.5	0.015
	Swelling	0	0	0.0	0.0~18.5	0	0	0.0	0.0~18.5	0	0	0.0	0.0~18.5	1	1	5.6	0.1~27.3	>0.999
	Other adverse reactions	0	0	0.0	0.0~18.5	0	0	0.0	0.0~18.5	0	0	0.0	0.0~18.5	0	0	0.0	0.0~18.5	-
	Laryngeal pain	0	0	0.0	0.0~18.5	0	0	0.0	0.0~18.5	0	0	0.0	0.0~18.5	0	0	0.0	0.0~18.5	-
	Blistering of oral mucosa	0	0	0.0	0.0~18.5	0	0	0.0	0.0~18.5	0	0	0.0	0.0~18.5	0	0	0.0	0.0~18.5	-
	runny nose	0	0	0.0	0.0~18.5	0	0	0.0	0.0~18.5	0	0	0.0	0.0~18.5	0	0	0.0	0.0~18.5	-
	Pharyngeal swelling	0	0	0.0	0.0~18.5	0	0	0.0	0.0~18.5	0	0	0.0	0.0~18.5	0	0	0.0	0.0~18.5	-
Aged 6-12 (N=18)	Total adverse reactions	6	3	16.7	3.6~41.4	8	6	33.3	13.3~59.0	2	1	5.6	0.1~27.3	7	6	33.3	13.3~59.0	0.123
	Systemic adverse reactions	1	1	5.6	0.1~27.3	1	1	5.6	0.1~27.3	1	1	5.6	0.1~27.3	2	2	11.1	1.4~34.7	0.886
	Hypersensitivity	0	0	0.0	0.0~18.5	0	0	0.0	0.0~18.5	0	0	0.0	0.0~18.5	1	1	5.6	0.1~27.3	>0.999
	Fever	0	0	0.0	0.0~18.5	1	1	5.6	0.1~27.3	0	0	0.0	0.0~18.5	0	0	0.0	0.0~18.5	>0.999
	Diarrhea	0	0	0.0	0.0~18.5	0	0	0.0	0.0~18.5	0	0	0.0	0.0~18.5	0	0	0.0	0.0~18.5	-
	Arthralgia	0	0	0.0	0.0~18.5	0	0	0.0	0.0~18.5	0	0	0.0	0.0~18.5	0	0	0.0	0.0~18.5	-
	Cough	1	1	5.6	0.1~27.3	0	0	0.0	0.0~18.5	1	1	5.6	0.1~27.3	1	1	5.6	0.1~27.3	>0.999
	Vomit	0	0	0.0	0.0~18.5	0	0	0.0	0.0~18.5	0	0	0.0	0.0~18.5	0	0	0.0	0.0~18.5	-
	Anorexia	0	0	0.0	0.0~18.5	0	0	0.0	0.0~18.5	0	0	0.0	0.0~18.5	0	0	0.0	0.0~18.5	-

	Headache	0	0	0.0	0.0~18.5	0	0	0.0	0.0~18.5	0	0	0.0	0.0~18.5	0	0	0.0	0.0~18.5	-
	Local adverse reaction	2	2	11.1	1.4~34.7	7	5	27.8	9.7~53.5	1	1	5.6	0.1~27.3	5	4	22.2	6.4~47.6	0.261
	Erythema	0	0	0.0	0.0~18.5	2	2	11.1	1.4~34.7	0	0	0.0	0.0~18.5	1	1	5.6	0.1~27.3	0.609
	Pain	2	2	11.1	1.4~34.7	5	5	27.8	9.7~53.5	1	1	5.6	0.1~27.3	4	4	22.2	6.4~47.6	0.261
	Swelling	0	0	0.0	0.0~18.5	0	0	0.0	0.0~18.5	0	0	0.0	0.0~18.5	0	0	0.0	0.0~18.5	-
	Other adverse reactions	3	1	5.6	0.1~27.3	0	0	0.0	0.0~18.5	0	0	0.0	0.0~18.5	0	0	0.0	0.0~18.5	>0.999
	Laryngeal pain	1	1	5.6	0.1~27.3	0	0	0.0	0.0~18.5	0	0	0.0	0.0~18.5	0	0	0.0	0.0~18.5	>0.999
	Blistering of oral mucosa	1	1	5.6	0.1~27.3	0	0	0.0	0.0~18.5	0	0	0.0	0.0~18.5	0	0	0.0	0.0~18.5	>0.999
	runny nose	0	0	0.0	0.0~18.5	0	0	0.0	0.0~18.5	0	0	0.0	0.0~18.5	0	0	0.0	0.0~18.5	-
	Pharyngeal swelling	1	1	5.6	0.1~27.3	0	0	0.0	0.0~18.5	0	0	0.0	0.0~18.5	0	0	0.0	0.0~18.5	>0.999
Aged 3-5 (N=24)	Total adverse reactions	11	9	37.5	18.8~59.4	7	6	25.0	9.8~46.7	9	6	25.0	9.8~46.7	7	6	25.0	9.8~46.7	0.708
	Systemic adverse reactions	4	4	16.7	4.7~37.4	6	5	20.8	7.1~42.2	8	5	20.8	7.1~42.2	3	3	12.5	2.7~32.4	0.853
	Hypersensitivity	0	0	0.0	0.0~14.2	0	0	0.0	0.0~14.2	0	0	0.0	0.0~14.2	0	0	0.0	0.0~14.2	-
	Fever	2	2	8.3	1.0~27.0	2	2	8.3	1.0~27.0	5	5	20.8	7.1~42.2	1	1	4.2	0.1~21.1	0.259
	Diarrhea	1	1	4.2	0.1~21.1	0	0	0.0	0.0~14.2	0	0	0.0	0.0~14.2	0	0	0.0	0.0~14.2	>0.999
	Arthralgia	0	0	0.0	0.0~14.2	0	0	0.0	0.0~14.2	0	0	0.0	0.0~14.2	0	0	0.0	0.0~14.2	-
	Cough	1	1	4.2	0.1~21.1	4	4	16.7	4.7~37.4	1	1	4.2	0.1~21.1	1	1	4.2	0.1~21.1	0.245
	Vomit	0	0	0.0	0.0~14.2	0	0	0.0	0.0~14.2	1	1	4.2	0.1~21.1	0	0	0.0	0.0~14.2	>0.999
	Anorexia	0	0	0.0	0.0~14.2	0	0	0.0	0.0~14.2	1	1	4.2	0.1~21.1	0	0	0.0	0.0~14.2	>0.999
	Headache	0	0	0.0	0.0~14.2	0	0	0.0	0.0~14.2	0	0	0.0	0.0~14.2	1	1	4.2	0.1~21.1	>0.999
	Local adverse reaction	6	5	20.8	7.1~42.2	1	1	4.2	0.1~21.1	1	1	4.2	0.1~21.1	4	4	16.7	4.7~37.4	0.155
	Erythema	1	1	4.2	0.1~21.1	0	0	0.0	0.0~14.2	0	0	0.0	0.0~14.2	1	1	4.2	0.1~21.1	>0.999
	Pain	5	4	16.7	4.7~37.4	1	1	4.2	0.1~21.1	1	1	4.2	0.1~21.1	3	3	12.5	2.7~32.4	0.346
	Swelling	0	0	0.0	0.0~14.2	0	0	0.0	0.0~14.2	0	0	0.0	0.0~14.2	0	0	0.0	0.0~14.2	-
	Other adverse reactions	1	1	4.2	0.1~21.1	0	0	0.0	0.0~14.2	0	0	0.0	0.0~14.2	0	0	0.0	0.0~14.2	>0.999
	Laryngeal pain	0	0	0.0	0.0~14.2	0	0	0.0	0.0~14.2	0	0	0.0	0.0~14.2	0	0	0.0	0.0~14.2	-
	Blistering of oral mucosa	0	0	0.0	0.0~14.2	0	0	0.0	0.0~14.2	0	0	0.0	0.0~14.2	0	0	0.0	0.0~14.2	-
	runny nose	1	1	4.2	0.1~21.1	0	0	0.0	0.0~14.2	0	0	0.0	0.0~14.2	0	0	0.0	0.0~14.2	>0.999
	Pharyngeal swelling	0	0	0.0	0.0~14.2	0	0	0.0	0.0~14.2	0	0	0.0	0.0~14.2	0	0	0.0	0.0~14.2	-
Aged 3-17 (N=60)	Total adverse reactions	25	19	31.7	20.3~45.0	17	14	23.3	13.4~36.0	14	10	16.7	8.3~28.5	23	18	30.0	18.8~43.2	0.216
	Systemic adverse reactions	7	7	11.7	4.8~22.6	9	8	13.3	5.9~24.6	11	8	13.3	5.9~24.6	6	6	10.0	3.8~20.5	0.934
	Hypersensitivity	0	0	0.0	0.0~6.0	0	0	0.0	0.0~6.0	0	0	0.0	0.0~6.0	1	1	1.7	0.0~8.9	>0.999

Fever	4	4	6.7	1.8~16.2	5	5	8.3	2.8~18.4	6	6	10.0	3.8~20.5	2	2	3.3	0.4~11.5	0.529
Diarrhea	1	1	1.7	0.0~8.9	0	0	0.0	0.0~6.0	0	0	0.0	0.0~6.0	0	0	0.0	0.0~6.0	>0.999
Arthralgia	0	0	0.0	0.0~6.0	0	0	0.0	0.0~6.0	1	1	1.7	0.0~8.9	0	0	0.0	0.0~6.0	>0.999
Cough	2	2	3.3	0.4~11.5	4	4	6.7	1.8~16.2	2	2	3.3	0.4~11.5	2	2	3.3	0.4~11.5	0.741
Vomit	0	0	0.0	0.0~6.0	0	0	0.0	0.0~6.0	1	1	1.7	0.0~8.9	0	0	0.0	0.0~6.0	>0.999
Anorexia	0	0	0.0	0.0~6.0	0	0	0.0	0.0~6.0	1	1	1.7	0.0~8.9	0	0	0.0	0.0~6.0	>0.999
Headache	0	0	0.0	0.0~6.0	0	0	0.0	0.0~6.0	0	0	0.0	0.0~6.0	1	1	1.7	0.0~8.9	>0.999
Local adverse reaction	14	13	21.7	12.1~34.2	8	6	10.0	3.8~20.5	3	3	5.0	1.0~13.9	17	14	23.3	13.4~36.0	0.010
Erythema	1	1	1.7	0.0~8.9	2	2	3.3	0.4~11.5	0	0	0.0	0.0~6.0	3	3	5.0	1.0~13.9	0.331
Pain	13	12	20.0	10.8~32.3	6	6	10.0	3.8~20.5	3	3	5.0	1.0~13.9	13	12	20.0	10.8~32.3	0.036
Swelling	0	0	0.0	0.0~6.0	0	0	0.0	0.0~6.0	0	0	0.0	0.0~6.0	1	1	1.7	0.0~8.9	>0.999
Other adverse reactions	4	2	3.3	0.4~11.5	0	0	0.0	0.0~6.0	0	0	0.0	0.0~6.0	0	0	0.0	0.0~6.0	0.247
Laryngeal pain	1	1	1.7	0.0~8.9	0	0	0.0	0.0~6.0	0	0	0.0	0.0~6.0	0	0	0.0	0.0~6.0	>0.999
Blistering of oral mucosa	1	1	1.7	0.0~8.9	0	0	0.0	0.0~6.0	0	0	0.0	0.0~6.0	0	0	0.0	0.0~6.0	>0.999
runny nose	1	1	1.7	0.0~8.9	0	0	0.0	0.0~6.0	0	0	0.0	0.0~6.0	0	0	0.0	0.0~6.0	>0.999
Pharyngeal swelling	1	1	1.7	0.0~8.9	0	0	0.0	0.0~6.0	0	0	0.0	0.0~6.0	0	0	0.0	0.0~6.0	>0.999

Note: total adverse reactions include inoculation site (local) adverse reactions, non inoculation site (systemic) adverse reactions and other adverse reactions. The low, medium, and high doses represent 2.5μg, 5.0μg, and 10.0 μg/dose, respectively.

Appendix 4. Severity of adverse reactions of subjects aged 3-17 years in phase I clinical trial after the full vaccination (based on cases)

Symptom	Group	Total	Level 1	Level 2	Level 3	*P*
Total adverse reactions	Low dose	25	19	6	0	0.465
	Medium dose	17	13	4	0	
	High dose	14	9	5	0	
	Placebo	23	20	3	0	
Local adverse reaction	Low dose	14	14	0	0	>0.999
	Medium dose	8	8	0	0	
	High dose	3	3	0	0	
	Placebo	17	16	1	0	
Erythema at vaccination site	Low dose	1	1	0	0	>0.999
	Medium dose	2	2	0	0	
	High dose	0	0	0	0	
	Placebo	3	2	1	0	
Pain at vaccination site	Low dose	13	13	0	0	-
	Medium dose	6	6	0	0	
	High dose	3	3	0	0	
	Placebo	13	13	0	0	
Swelling of vaccination site	Low dose	0	0	0	0	-
	Medium dose	0	0	0	0	
	High dose	0	0	0	0	
	Placebo	1	1	0	0	
Systemic adverse reactions	Low dose	7	5	2	0	0.893
	Medium dose	9	5	4	0	
	High dose	11	6	5	0	
	Placebo	6	4	2	0	
Hypersensitivity	Low dose	0	0	0	0	-
	Medium dose	0	0	0	0	
	High dose	0	0	0	0	
	Placebo	1	1	0	0	
Fever	Low dose	4	3	1	0	0.761
	Medium dose	5	3	2	0	

	High dose	6	2	4	0	
	Placebo	2	1	1	0	
Arthralgia	Low dose	0	0	0	0	-
	Medium dose	0	0	0	0	
	High dose	1	1	0	0	
	Placebo	0	0	0	0	
Diarrhea	Low dose	1	1	0	0	-
	Medium dose	0	0	0	0	
	High dose	0	0	0	0	
	Placebo	0	0	0	0	
Cough	Low dose	2	1	1	0	0.848
	Medium dose	4	2	2	0	
	High dose	2	2	0	0	
	Placebo	2	1	1	0	
Vomit	Low dose	0	0	0	0	-
	Medium dose	0	0	0	0	
	High dose	1	0	1	0	
	Placebo	0	0	0	0	
Anorexia	Low dose	0	0	0	0	-
	Medium dose	0	0	0	0	
	High dose	1	1	0	0	
	Placebo	0	0	0	0	
Headache	Low dose	0	0	0	0	-
	Medium dose	0	0	0	0	
	High dose	0	0	0	0	
	Placebo	1	1	0	0	
Other adverse reactions	Low dose	4	0	4	0	-
	Medium dose	0	0	0	0	
	High dose	0	0	0	0	
	Placebo	0	0	0	0	
Laryngeal pain	Low dose	1	0	1	0	-
	Medium dose	0	0	0	0	

	High dose	0	0	0	0	
	Placebo	0	0	0	0	
Blistering of oral mucosa	Low dose	1	0	1	0	-
	Medium dose	0	0	0	0	
	High dose	0	0	0	0	
	Placebo	0	0	0	0	
runny nose	Low dose	1	0	1	0	-
	Medium dose	0	0	0	0	
	High dose	0	0	0	0	
	Placebo	0	0	0	0	
Pharyngeal swelling	Low dose	1	0	1	0	-
	Medium dose	0	0	0	0	
	High dose	0	0	0	0	
	Placebo	0	0	0	0	

The low, medium, and high doses represent 2.5μg, 5.0μg, and 10.0 μg/dose, respectively.

Appendix 5. Incidence of serious adverse events in subjects 3-17 years years in phase I clinical trial

SOC	PT	Age group	Group	N	Case times	Number of cases	Rate(	95%CI)
**Infectious and infectious diseases**	Total	13-17 years	Low dose	18	0	0	0	0.0~18.5
			Medium dose	18	0	0	0	0.0~18.5
			High dose	18	0	0	0	0.0~18.5
			Placebo	18	0	0	0	0.0~18.5
		6-12 years	Low dose	18	1	1	5.6	0.1~27.3
			Medium dose	18	0	0	0	0.0~18.5
			High dose	18	0	0	0	0.0~18.5
			Placebo	18	0	0	0	0.0~18.5
		3-5 years	Low dose	24	0	0	0	0.0~14.2
			Medium dose	24	0	0	0	0.0~14.2
			High dose	24	0	0	0	0.0~14.2
			Placebo	24	0	0	0	0.0~14.2
		3-17 years	Low dose	60	1	1	1.7	0.0~8.9
			Medium dose	60	0	0	0	0.0~6.0
			High dose	60	0	0	0	0.0~6.0
			Placebo	60	0	0	0	0.0~6.0
	Infectious pneumonia	13-17 years	Low dose	18	0	0	0	0.0~18.5
			Medium dose	18	0	0	0	0.0~18.5
			High dose	18	0	0	0	0.0~18.5
			Placebo	18	0	0	0	0.0~18.5
		6-12 years	Low dose	18	1	1	5.6	0.1~27.3
			Medium dose	18	0	0	0	0.0~18.5
			High dose	18	0	0	0	0.0~18.5
			Placebo	18	0	0	0	0.0~18.5
		3-5 years	Low dose	24	0	0	0	0.0~14.2
			Medium dose	24	0	0	0	0.0~14.2
			High dose	24	0	0	0	0.0~14.2
			Placebo	24	0	0	0	0.0~14.2

		3-17 years	Low dose	60	1	1	1.7	0.0~8.9
			Medium dose	60	0	0	0	0.0~6.0
			High dose	60	0	0	0	0.0~6.0
			Placebo	60	0	0	0	0.0~6.0
**Gastrointestinal diseases**	Total	13-17 years	Low dose	18	0	0	0	0.0~18.5
			Medium dose	18	0	0	0	0.0~18.5
			High dose	18	0	0	0	0.0~18.5
			Placebo	18	0	0	0	0.0~18.5
		6-12 years	Low dose	18	0	0	0	0.0~18.5
			Medium dose	18	0	0	0	0.0~18.5
			High dose	18	0	0	0	0.0~18.5
			Placebo	18	0	0	0	0.0~18.5
		3-5 years	Low dose	24	0	0	0	0.0~14.2
			Medium dose	24	0	0	0	0.0~14.2
			High dose	24	1	1	4.2	0.1~21.1
			Placebo	24	0	0	0	0.0~14.2
		3-17 years	Low dose	60	0	0	0	0.0~6.0
			Medium dose	60	0	0	0	0.0~6.0
			High dose	60	1	1	1.7	0.0~8.9
			Placebo	60	0	0	0	0.0~6.0
	enteritis	13-17 years	Low dose	18	0	0	0	0.0~18.5
			Medium dose	18	0	0	0	0.0~18.5
			High dose	18	0	0	0	0.0~18.5
			Placebo	18	0	0	0	0.0~18.5
		6-12 years	Low dose	18	0	0	0	0.0~18.5
			Medium dose	18	0	0	0	0.0~18.5
			High dose	18	0	0	0	0.0~18.5
			Placebo	18	0	0	0	0.0~18.5
		3-5 years	Low dose	24	0	0	0	0.0~14.2
			Medium dose	24	0	0	0	0.0~14.2

	High dose	24	1	1	4.2	0.1~21.1
	Placebo	24	0	0	0	0.0~14.2
3-17 years	Low dose	60	0	0	0	0.0~6.0
	Medium dose	60	0	0	0	0.0~6.0
	High dose	60	1	1	1.7	0.0~8.9
	Placebo	60	0	0	0	0.0~6.0

The low, medium, and high doses represent 2.5μg, 5.0μg, and 10.0 μg/dose, respectively.

Appendix 6. Antibody GMT (95% CI) of phase I subjects aged 3-17 years at different time points（FAS）

Age group	point of time	Low dose N	Low dose GMT(95%CI)	Medium dose N	Medium dose GMT(95%CI)	High dose N	High dose GMT(95%CI)	Placebo N	Placebo GMT(95%CI)	*P*
13-17 years	**Neutralizing Antibodies to Live SARS-CoV-2**									
	Before 1st dose immunization	18	5(5~5)	18	5(5~5)	18	5(5~5)	18	5(5~5)	
	28 days after the second dose of immunization	18	83(53.5~129)	18	180.8(58.5~141)	18	130.7(84.2~203)	18	5(3.2~7.8)	<0.001
	28 days after the third dose of immunization	18	281.4(171.4~462.1)	18	167.1(101.8~274.4)	18	166.1(101.2~272.7)	18	5(3~8.2)	<0.001
	**Specific Antibody Responses to SARS-CoV-2**									
	Before 1st dose immunization	18	10(10~10)	18	10(10~10)	18	10(10~10)	18	10(10~10)	
	28 days after the second dose of immunization	18	137.2(101.8~184.9)	18	154(114.2~207.5)	18	154(114.2~207.5)	18	10(7.4~13.5)	<0.001
	28 days after the third dose of immunization	18	345.6(263.4~453.4)	18	274.3(209.1~359.9)	18	244.4(186.3~320.6)	18	10(7.6~13.1)	<0.001
6-12 years	**Neutralizing Antibodies to Live SARS-CoV-2**									
	Before 1st dose immunization	18	5(5~5)	18	5(5~5)	18	5(5~5)	18	5(5~5)	
	28 days after the second dose of immunization	18	102.5(70.3~149.2)	18	318.4(218.6~463.7)	18	298.1(204.6~434.1)	18	5(3.4~7.3)	<0.001
	28 days after the third dose of immunization	18	292.7(187.6~456.8)	18	447.3(286.6~698.1)	18	446.5(286.1~696.7)	18	5(3.2~7.8)	<0.001
	**Specific Antibody Responses to SARS-CoV-2**									
	Before 1st dose immunization	18	10(10~10)	18	10(10~10)	18	10(10~10)	18	10(10~10)	
	28 days after the second dose of immunization	18	154(117.7~201.4)	18	264(201.8~345.3)	18	264(201.8~345.3)	18	10(7.6~13.1)	<0.001
	28 days after the third dose of immunization	18	320(238.9~428.7)	18	548.6(409.5~735)	18	527.9(394~707.2)	18	10(7.5~13.4)	<0.001
3-5 years	**Neutralizing Antibodies to Live SARS-CoV-2**									
	Before 1st dose immunization	24	5(5~5)	24	5(5~5)	24	5(5~5)	24	5(5~5)	
	28 days after the second dose of immunization	24	177.9(124.6~253.8)	24	256.4(179.6~365.9)	24	356.8(250~509.2)	22	5(3.4~7.2)	<0.001
	28 days after the third dose of immunization	24	469.3(326.1~675.3)	24	782.2(543.5~1125.7)	24	845.7(587.7~1217.1)	22	5(3.4~7.3)	<0.001
	**Specific Antibody Responses to SARS-CoV-2**									
	Before 1st dose immunization	24	10(10~10)	24	10(10~10)	24	10(10~10)	24	10(10~10)	

	28 days after the second dose of immunization	24	246.8(189.3~321.6)	24	285.1(218.7~371.5)	24	329.4(252.7~429.3)	22	10(7.6~13.2)	<0.001
	28 days after the third dose of immunization	24	621.8(448.1~862.9)	24	1015.9(732.1~1409.8)	24	1805.1(652.2~1256)	22	10(7.1~14.1)	<0.001
3-17 years	**Neutralizing Antibodies to Live SARS-CoV-2**									
	Before 1st dose immunization	60	5(5~5)	60	5(5~5)	60	5(5~5)	60	5(5~5)	
	28 days after the second dose of immunization	60	119.9(94.4~152.3)	60	200.4(157.8~254.5)	60	250.1(196.9~317.6)	58	5(3.9~6.4)	<0.001
	28 days after the third dose of immunization	60	349.4(266.6~457.9)	60	416.3(317.7~545.5)	60	428.5(327~561.5)	58	5(3.8~6.6)	<0.001
	**Specific Antibody Responses to SARS-CoV-2**									
	Before 1st dose immunization	60	10(10~10)	60	10(10~10)	60	10(10~10)	60	10(10~10)	
	28 days after the second dose of immunization	60	179.6(151.8~212.5)	60	231.6(195.7~274)	60	245.3(207.3~2180.3)	58	10(8.4~11.9)	<0.001
	28 days after the third dose of immunization	60	427.1(348.8~523.2)	60	570.2(465.5~698.3)	60	519.8(424.4~636.7)	58	10(8.1~12.3)	<0.001

The low, medium, and high doses represent 2.5μg, 5.0μg, and 10.0 μg/dose, respectively.

Appendix 7. Antibody GMT (95% CI) of phase I subjects aged 3-17 years at different time points（PPS）

Age group	point of time	Low dose		Medium dose		High dose		Placebo		*P*
		N	GMT(95%CI)	N	GMT(95%CI)	N	GMT(95%CI)	N	GMT(95%CI)	
13-17 years	**Neutralizing Antibodies to Live SARS-CoV-2**									
	Before 1st dose immunization	18	5(5~5)	18	5(5~5)	18	5(5~5)	18	5(5~5)	
	28 days after the second dose of immunization	18	83(53.5~129)	18	180.8(58.5~141)	18	130.7(84.2~203)	18	5(3.2~7.8)	<0.001
	28 days after the third dose of immunization	18	281.4(171.4~462.1)	18	167.1(101.8~274.4)	18	166.1(101.2~272.7)	18	5(3~8.2)	<0.001
	**Specific Antibody Responses to SARS-CoV-2**									
	Before 1st dose immunization	18	10(10~10)	18	10(10~10)	18	10(10~10)	18	10(10~10)	
	28 days after the second dose of immunization	18	137.2(101.8~184.9)	18	154(114.2~207.5)	18	154(114.2~207.5)	18	10(7.4~13.5)	<0.001
	28 days after the third dose of immunization	18	345.6(263.4~453.4)	18	274.3(209.1~359.9)	18	244.4(186.3~320.6)	18	10(7.6~13.1)	<0.001
6-12 years	Neutralizing Antibodies to Live SARS-CoV-2									
	Before 1st dose immunization	18	5(5~5)	18	5(5~5)	18	5(5~5)	18	5(5~5)	
	28 days after the second dose of immunization	18	102.5(70.3~149.2)	18	318.4(218.6~463.7)	18	298.1(204.6~434.1)	18	5(3.4~7.3)	<0.001
	28 days after the third dose of immunization	18	292.7(189.9~451.3)	17	502.7(322~784.8)	18	446.5(289.6~688.3)	18	5(3.2~7.7)	<0.001
	**Specific Antibody Responses to SARS-CoV-2**									
	Before 1st dose immunization	18	10(10~10)	18	10(10~10)	18	10(10~10)	18	10(10~10)	
	28 days after the second dose of immunization	18	154(117.7~201.4)	18	264(201.8~345.3)	18	264(201.8~345.3)	18	10(7.6~13.1)	<0.001
	28 days after the third dose of immunization	18	320(238.7~428.9)	17	566.3(418.9~765.6)	18	527.9(393.8~707.6)	18	10(7.5~13.4)	<0.001
3-5 years	**Neutralizing Antibodies to Live SARS-CoV-2**									
	Before 1st dose immunization	24	5(5~5)	24	5(5~5)	24	5(5~5)	24	5(5~5)	
	28 days after the second dose of immunization	24	177.9(124.5~254.1)	24	256.4(179.4~366.3)	23	366.2(254.3~527.3)	22	5(3.4~7.3)	<0.001
	28 days after the third dose of	24	469.3(325.4~676.8)	24	782.2(542.4~1128)	23	848(583.4~1232.6)	22	5(3.4~7.3)	<0.001

	immunization									
	**Specific Antibody Responses to SARS-CoV-2**									
	Before 1st dose immunization	24	10(10~10)	24	10(10~10)	24	10(10~10)	24	10(10~10)	
	28 days after the second dose of immunization	24	246.8(1180.4~319.7)	24	285.1(220~369.4)	23	350.3(268.8~456.4)	22	10(7.6~13.1)	<0.001
	28 days after the third dose of immunization	24	621.8(447.4~864.2)	24	1015.9(731~1412)	23	918.8(656.4~1286.1)	22	10(7.1~14.1)	<0.001
3-17 years	**Neutralizing Antibodies to Live SARS-CoV-2**									
	Before 1st dose immunization	60	5(5~5)	60	5(5~5)	60	5(5~5)	60	5(5~5)	
	28 days after the second dose of immunization	60	119.9(94.4~152.4)	60	200.4(157.7~254.6)	59	251.1(197.3~319.8)	58	5(3.9~6.4)	<0.001
	28 days after the third dose of immunization	60	349.4(266.9~457.4)	59	430(327.7~564.2)	59	424(323.1~556.4)	58	5(3.8~6.6)	<0.001
	**Specific Antibody Responses to SARS-CoV-2**									
	Before 1st dose immunization	60	10(10~10)	60	10(10~10)	60	10(10~10)	60	10(10~10)	
	28 days after the second dose of immunization	60	179.6(151.9~212.4)	60	231.6(195.8~273.8)	59	250(211.1~296.1)	58	10(8.4~11.9)	<0.001
	28 days after the third dose of immunization	60	427.1(348.5~523.5)	59	575.8(469~706.9)	59	518(422~635.9)	58	10(8.1~12.3)	<0.001

The low, medium, and high doses represent 2.5μg, 5.0μg, and 10.0 μg/dose, respectively.

Appendix 8. Comparison of antibody levels of phase I subjects aged 3-17 years 28 days after the full vaccination（FAS）

Age group	Antibody type	Low dose (N =18)	Medium dose (N = 18)	High dose (N =18)	Placebo (N = 18)
13-17 years	**Neutralizing Antibodies to Live SARS-CoV-2**				
	GMT (95% CI)	281.4(171.4~462.1)	167.1(101.8~274.4)	166.1(101.2~272.7)	5(3~8.2)
	GMI (95% CI)	1	0.59(0.29~1.20)	0.59(0.29~1.19)	
	Antibody 4-fold growth rate— % (95% CI)	94.4(72.7~99.9)	94.4(72.7~99.9)	94.4(72.7~99.9)	0
	**Specific Antibody Responses to SARS-CoV-2**				
	GMT (95% CI)	345.6(263.4~453.4)	274.3(209.1~359.9)	244.4(186.3~320.6)	10(7.6~13.1)
	GMI (95% CI)	1	0.79(0.54~1.17)	0.79(0.54~1.17)	-
	Antibody 4-fold growth rate — % (95% CI)	100	100	100	0
**Age group**	**Antibody type**	**Low dose (N = 18)**	**Medium dose (N = 18)**	**High dose (N =18)**	**Placebo (N =18)**
6-12 years	**Neutralizing Antibodies to Live SARS-CoV-2**				
	GMT (95% CI)	292.7(187.6~456.8)	447.3(286.6~698.1)	446.5(286.1~696.7)	5(3.2~7.8)
	GMI (95% CI)	1	1.53(0.81~2.87)	1.53(0.81~2.86)	-
	Antibody 4-fold growth rate— % (95% CI)	94.4(72.7~99.9)	100	100	0
	**Specific Antibody Responses to SARS-CoV-2**				
	GMT (95% CI)	320(238.9~428.7)	548.6(409.5~735)	527.9(394~707.2)	10(7.5~13.4)
	GMI (95% CI)	1	1.71(1.13~2.59)	1.65(1.09~2.49)	-
	Antibody 4-fold growth rate — % (95% CI)	100	100	100	0
**Age group**	**Antibody type**	**Low dose (N =24)**	**Medium dose (N = 24)**	**High dose (N =24)**	**Placebo (N =22)**
3-5 years	**Neutralizing Antibodies to Live SARS-CoV-2**				
	GMT (95% CI)	469.3(326.1~675.3)	782.2(543.5~1125.7)	845.7(587.7~1217.1)	5(3.4~7.3)
	GMI (95% CI)	1	1.67(1~2.79)	1.8(1.08~3.02)	-
	Antibody 4-fold growth rate— % (95% CI)	100	100	100	0
	**Specific Antibody Responses to SARS-CoV-2**				

	GMT (95% CI)	621.8(448.1~862.9)	1015.9(732.1~1409.8)	1805.1(652.2~1256)	10(7.1~14.1)
	GMI (95% CI)	1	1.63(1.03~2.6)	1.46(0.92~2.31)	-
	Antibody 4-fold growth rate — % (95% CI)	100	100	100	0
Age group	Antibody type	Low dose (N =60)	Medium dose (N =60)	High dose (N =60)	Placebo (N =58)
3-17 years	**Neutralizing Antibodies to Live SARS-CoV-2**				
	GMT (95% CI)	349.4(266.6~457.9)	416.3(317.7~545.5)	428.5(327~561.5)	5(3.8~6.6)
	GMI (95% CI)	1	1.19(0.81~1.75)	1.23(0.84~1.8)	-
	Antibody 4-fold growth rate— % (95% CI)	96.7(88.5~99.6)	98.3(180.9~100.0)	98.3(180.9~100.0)	0
	**Specific Antibody Responses to SARS-CoV-2**				
	GMT (95% CI)	427.1(348.8~523.2)	570.2(465.5~698.3)	519.8(424.4~636.7)	10(8.1~12.3)
	GMI (95% CI)	1	1.33(1~1.78)	1.22(0.91~1.62)	-
	Antibody 4-fold growth rate — % (95% CI)	100	100	100	0

The low, medium, and high doses represent 2.5μg, 5.0μg, and 10.0 μg/dose, respectively.

Appendix 9. Comparison of antibody levels of phase I subjects aged 3-17 years 28 days after the full vaccination（PPS）

Age group	Antibody type	Low dose (N = 18)	Medium dose (N =18)	High dose (N =18)	Placebo (N =18)
13-17 years	**Neutralizing Antibodies to Live SARS-CoV-2**				
	GMT (95% CI)	281.4(171.4~462.1)	167.1(101.8~274.4)	166.1(101.2~272.7)	5(3~8.2)
	GMI (95% CI)	1	0.59(0.29~1.2)	0.59(0.29~1.19)	-
	Antibody 4-fold growth rate— % (95% CI)	94.4(72.7~99.9)	94.4(72.7~99.9)	94.4(72.7~99.9)	0
	**Specific Antibody Responses to SARS-CoV-2**				
	GMT (95% CI)	345.6(263.4~453.4)	274.3(209.1~359.9)	244.4(186.3~320.6)	10(7.6~13.1)
	GMI (95% CI)	1	0.79(0.54~1.17)	0.71(0.48~1.04)	-
	Antibody 4-fold growth rate — % (95% CI)	100	100	100	0
**Age group**	**Antibody type**	**Low dose (N = 18)**	**Medium dose (N =17)**	**High dose (N =18)**	**Placebo (N =18)**
6-12 years	**Neutralizing Antibodies to Live SARS-CoV-2**				
	GMT (95% CI)	292.7(189.9~451.3)	502.7(322~784.8)	446.5(289.6~688.3)	5(3.2~7.7)
	GMI (95% CI)	1	1.72(0.92~3.2)	1.53(0.83~2.81)	-
	Antibody 4-fold growth rate— % (95% CI)	94.4(72.7~99.9)	100	100	0
	**Specific Antibody Responses to SARS-CoV-2**				
	GMT (95% CI)	320(238.7~428.9)	566.3(418.9~765.6)	527.9(393.8~707.6)	10(7.5~13.4)
	GMI (95% CI)	1	1.77(1.16~2.69)	1.65(1.09~2.5)	-
	Antibody 4-fold growth rate — % (95% CI)	100	100	100	0
**Age group**	**Antibody type**	**Low dose (N = 24)**	**Medium dose (N =24)**	**High dose (N =23)**	**Placebo (N =22)**
3-5 years	**Neutralizing Antibodies to Live SARS-CoV-2**				
	GMT (95% CI)	469.3(325.4~676.8)	782.2(542.4~1128)	848(583.4~1232.6)	5(3.4~7.3)
	GMI (95% CI)	1	1.67(0.99~2.8)	1.81(1.07~3.05)	-
	Antibody 4-fold growth rate— % (95% CI)	100	100	100	0
	**Specific Antibody Responses to SARS-CoV-2**				
	GMT (95% CI)	621.8(447.4~864.2)	1015.9(731~1412)	918.8(656.4~1286.1)	10(7.1~14.1)

	GMI (95% CI)	1	1.63(1.03~2.6)	1.48(0.92~2.37)	-
	Antibody 4-fold growth rate — % (95% CI)	100	100	100	0
**Age group**	**Antibody type**	**Low dose (N = 60)**	**Medium dose (N =59)**	**High dose (N =59)**	**Placebo (N =58)**
3-17 years	**Neutralizing Antibodies to Live SARS-CoV-2**				
	GMT (95% CI)	349.4(266.9~457.4)	430(327.7~564.2)	424(323.1~556.4)	5(3.8~6.6)
	GMI (95% CI)	1	1.23(0.84~1.8)	1.21(0.83~1.78)	-
	Antibody 4-fold growth rate— % (95% CI)	96.7(88.5~99.6)	98.3(180.9~100.0)	98.3(180.9~100.0)	0
	**Specific Antibody Responses to SARS-CoV-2**				
	GMT (95% CI)	427.1(348.5~523.5)	575.8(469~706.9)	518(422~635.9)	10(8.1~12.3)
	GMI (95% CI)	1	1.35(1.01~1.8)	1.21(0.91~1.62)	-
	Antibody 4-fold growth rate — % (95% CI)	100	100	100	0

The low, medium, and high doses represent 2.5μg, 5.0μg, and 10.0 μg/dose, respectively.

Appendix 10. Quadruple growth rate of antibody at different time points in subjects aged 3-17 years in phase I (95% CI)（PPS）

Age group	point of time	Low dose N	Low dose Antibody 4-fold growth rate	Low dose Rate(95%CI)	Medium dose N	Medium dose Antibody 4-fold growth rate	Medium dose Rate(95%CI)	High dose N	High dose Antibody 4-fold growth rate	High dose Rate(95%CI)	Placebo N	Placebo Antibody 4-fold growth rate	Placebo Rate(95%CI)	*P*
13-17 years	**Neutralizing Antibodies to Live SARS-CoV-2**													
	28 days after the second dose of immunization	18	17	94.4(72.7~99.9)	18	17	94.4(72.7~99.9)	18	17	94.4(72.7~99.9)	18	0	0	<0.001
	28 days after the third dose of immunization	18	17	94.4(72.7~99.9)	18	17	94.4(72.7~99.9)	18	17	94.4(72.7~99.9)	18	0	0	<0.001
	**Specific Antibody Responses to SARS-CoV-2**													
	28 days after the second dose of immunization	18	18	100	18	17	94.4(72.7~99.9)	18	18	100	18	0	0	<0.001
	28 days after the third dose of immunization	18	18	100	18	18	100	18	18	100	18	0	0	<0.001
6-12 years	**Neutralizing Antibodies to Live SARS-CoV-2**													
	28 days after the second dose of immunization	18	17	94.4(72.7~99.9)	18	18	100	18	18	100	18	0	0	<0.001
	28 days after the third dose of immunization	18	17	94.4(72.7~99.9)	17	17	100	18	18	100	18	0	0	<0.001
	**Specific Antibody Responses to SARS-CoV-2**													
	28 days after the second dose of immunization	18	18	100	18	18	100	18	18	100	18	0	0	<0.001
	28 days after the third dose of immunization	18	18	100	17	17	100	18	18	100	18	0	0	<0.001
3-5 years	**Neutralizing Antibodies to Live SARS-CoV-2**													
	28 days after the second dose of immunization	24	23	95.8(78.9~99.9)	24	23	95.8(78.9~99.9)	23	23	100	22	0	0	<0.001

	28 days after the third dose of immunization	24	24	100	24	24	100	23	23	100	22	0	0	<0.001
	**Specific Antibody Responses to SARS-CoV-2**													
	28 days after the second dose of immunization	24	24	100	24	24	100	23	23	100	22	0	0	<0.001
	28 days after the third dose of immunization	24	24	100	24	24	100	23	23	100	22	0	0	<0.001
3-17 years	**Neutralizing Antibodies to Live SARS-CoV-2**													
	28 days after the second dose of immunization	60	57	95.0 (86.1~99.0)	60	58	96.7(88.5~99.6)	59	58	98.3(180.9~100.0)	58	0	0	<0.001
	28 days after the third dose of immunization	60	58	96.7(88.5~99.6)	59	58	98.3(180.9~100.0)	59	58	98.3(180.9~100.0)	58	0	0	<0.001
	**Specific Antibody Responses to SARS-CoV-2**													
	28 days after the second dose of immunization	60	60	100	60	59	98.3(91.1~100.0)	59	59	100	58	0	0	<0.001
	28 days after the third dose of immunization	60	60	100	59	59	100	59	59	100	58	0	0	<0.001

The low, medium, and high doses represent 2.5μg, 5.0μg, and 10.0 μg/dose, respectively.

Appendix 11. Quadruple growth rate of antibody at different time points in subjects aged 3-17 years in phase I (95% CI)（FAS）

Age group	point of time	Low dose			Medium dose			High dose			Placebo			*P*
		N	Antibody 4-fold growth rate	Rate(95%CI)	N	Antibody 4-fold growth rate	Rate(95%CI)	N	Antibody 4-fold growth rate	Rate(95%CI)	N	Antibody 4-fold growth rate	Rate(95%CI)	
13-17 years	**Neutralizing Antibodies to Live SARS-CoV-2**													
	28 days after the second dose of immunization	18	17	94.4(72.7~99.9)	18	17	94.4(72.7~99.9)	18	17	94.4(72.7~99.9)	18	0	0	<0.001
	28 days after the third dose of immunization	18	17	94.4(72.7~99.9)	18	17	94.4(72.7~99.9)	18	17	94.4(72.7~99.9)	18	0	0	<0.001
	**Specific Antibody Responses to SARS-CoV-2**													
	28 days after the second dose of immunization	18	18	100	18	17	94.4(72.7~99.9)	18	18	100	18	0	0	<0.001
	28 days after the third dose of immunization	18	18	100	18	18	100	18	18	100	18	0	0	<0.001
6-12 years	**Neutralizing Antibodies to Live SARS-CoV-2**													
	28 days after the second dose of immunization	18	17	94.4(72.7~99.9)	18	18	100	18	18	100	18	0	0	<0.001
	28 days after the third dose of immunization	18	17	94.4(72.7~99.9)	18	18	100	18	18	100	18	0	0	<0.001
	**Specific Antibody Responses to SARS-CoV-2**													
	28 days after the second dose of immunization	18	18	100	18	18	100	18	18	100	18	0	0	<0.001
	28 days after the third dose of immunization	18	18	100	18	18	100	18	18	100	18	0	0	<0.001
3-5 years	**Neutralizing Antibodies to Live SARS-CoV-2**													
	28 days after the second dose of immunization	24	23	95.8(78.9~99.9)	24	23	95.8(78.9~99.9)	24	24	100	22	0	0	<0.001

	28 days after the third dose of immunization	24	24	100	24	24	100	24	24	100	22	0	0	<0.001
	**Specific Antibody Responses to SARS-CoV-2**													
	Before 1st dose immunization	24			24			24			24			
	28 days after the second dose of immunization	24	24	100	24	24	100	24	24	100	22	0	0	<0.001
	28 days after the third dose of immunization	24	24	100	24	24	100	24	24	100	22	0	0	<0.001
3-17 years	**Neutralizing Antibodies to Live SARS-CoV-2**													
	28 days after the second dose of immunization	60	57	95(86.1~99.0)	60	58	96.7(88.5~99.6)	60	59	98.3(91.1~100.0)	58	0	0	<0.001
	28 days after the third dose of immunization	60	58	96.7(88.5~99.6)	60	59	98.3(91.1~100.0)	60	59	98.3(91.1~100.0)	58	0	0	<0.001
	**Specific Antibody Responses to SARS-CoV-2**													
	28 days after the second dose of immunization	60	60	100	60	59	98.3(91.1~100.0)	60	60	100	58	0	0	<0.001
	28 days after the third dose of immunization	60	60	100	60	60	100	60	60	100	58	0	0	<0.001

The low, medium, and high doses represent 2.5μg, 5.0μg, and 10.0 μg/dose, respectively.

Appendix 12. Incidence of adverse events within 0-7 days (including 30 minutes) after the full vaccination in subjects aged 3-17 years years in phase II

		Low dose				Medium dose				High dose				Placebo				*P*
		Case times	Number of cases	Rate(%）	95%CI	Case times	Number of cases	Rate(%)	95%CI	Case times	Number of cases	Rate(%)	95%CI	Case times	Number of cases	Rate(%)	95%CI	
13-17 years (N=42)	Total adverse events	9	7	16.7	7.0~31.4	9	7	16.7	7.0~31.4	12	9	21.4	10.3~36.8	13	11	26.2	13.9~42.0	0.654
	Systemic adverse events	2	2	4.8	0.6~12.6	4	4	9.5	2.7~22.6	5	4	9.5	2.7~22.6	4	4	9.5	2.7~22.6	0.817
	Local adverse events	6	5	11.9	4.0~25.6	5	4	9.5	2.7~22.6	7	7	16.7	7.0~31.4	8	7	16.7	7.0~31.4	0.715
	Other adverse events	1	1	2.4	0.1~12.6	0	0	0	0.0~8.4	0	0	0	0.0~8.4	1	1	2.4	0.1~12.6	>0.999
6-12 years (N=42)	Total adverse events	5	5	11.9	4.0~25.6	6	4	9.5	2.7~22.6	1	1	2.4	0.1~12.6	11	6	14.3	5.4~28.5	0.276
	Systemic adverse events	0	0	0	0.0~8.4	0	0	0	0.0~8.4	1	1	2.4	0.1~12.6	1	1	2.4	0.1~12.6	>0.999
	Local adverse events	4	4	9.5	2.7~22.6	5	3	7.1	1.5~19.5	0	0	0	0.0~8.4	8	4	9.5	2.7~22.6	0.242
	Other adverse events	1	1	2.4	0.1~12.6	1	1	2.4	0.1~12.6	0	0	0	0.0~8.4	2	2	4.8	0.6~12.6	0.562
3-5 years (N=60)	Total adverse events	10	9	15	7.1~26.6	14	12	20	10.8~32.3	11	8	13.3	5.9~24.6	13	10	16.7	8.3~28.5	0.784
	Systemic adverse events	7	7	11.7	4.8~22.6	7	6	10	3.8~20.5	7	6	10	3.8~20.5	8	7	11.7	4.8~22.6	0.982
	Local adverse events	2	2	3.3	0.4~11.5	3	3	5	1.0~13.9	2	2	3.3	0.4~11.5	4	4	6.7	1.8~16.2	0.7180
	Other adverse events	1	1	1.7	0.0~8.9	4	4	6.7	1.8~16.2	2	1	1.7	0.0~8.9	1	1	1.7	0.0~8.9	0.264
3-17 years (N=144)	Total adverse events	24	21	14.6	9.3~21.4	29	23	16	10.4~23.0	24	18	12.5	7.6~19.0	37	27	18.8	12.7~26.1	0.518
	Systemic adverse events	9	9	6.3	2.9~11.5	11	10	6.9	3.4~12.4	13	11	7.6	3.9~13.3	13	12	8.3	4.4~14.1	0.916
	Local adverse events	12	11	7.6	3.9~13.3	13	10	6.9	3.4~12.4	9	9	6.3	2.9~11.5	20	15	10.4	5.9~16.6	0.572
	Other adverse events	3	3	2.1	0.4~6.0	5	5	3.5	1.1~7.9	2	1	0.7	0.0~3.8	4	4	2.8	0.8~7.0	0.431

Note: total adverse events include adverse events at inoculation site (local), adverse events at non inoculation site (systemic) and other adverse events. The low, medium, and high doses represent 2.5μg, 5.0μg, and 10.0 μg/dose, respectively.

Appendix 13. Incidence of adverse events within 30 days after whole course exemption in subjects aged 3-17 years in phase II

		Low dose				Medium dose				High dose				Placebo				*P*
		Case times	Number of cases	Rate(%）	95%CI	Case times	Number of cases	Rate(%)	95%CI	Case times	Number of cases	Rate(%)	95%CI	Case times	Number of cases	Rate(%)	95%CI	
13-17 years (N=42)	Total adverse events	30	15	35.7	21.6~52.0	9	7	16.7	7.0~31.4	13	10	23.8	12.1~39.5	20	16	38.1	23.6~54.4	0.098
	Systemic adverse events	7	6	14.3	5.4~28.5	4	4	9.5	2.7~22.6	6	5	11.9	4.0~25.6	7	7	16.7	7.0~31.4	0.79
	Local adverse events	6	5	11.9	4.0~25.6	5	4	9.5	2.7~22.6	7	7	16.7	7.0~31.4	8	7	16.7	7.0~31.4	0.715
	Other adverse events	17	11	26.2	13.9~42.0	0	0	0	0.0~8.4	0	0	0	0.0~8.4	5	5	11.9	4.0~25.6	<0.001
6-12 years (N=42)	Total adverse events	19	14	33.3	19.6~49.5	13	9	21.4	10.3~36.8	4	4	9.5	2.7~22.6	18	9	21.4	10.3~36.8	0.07
	Systemic adverse events	5	5	11.9	4.0~25.6	2	2	4.8	0.6~16.2	3	3	7.1	1.5~19.5	3	2	4.8	0.6~16.2	0.541
	Local adverse events	4	4	9.5	2.7~22.6	5	3	7.1	1.5~19.5	0	0	0	0.0~8.4	8	4	9.5	2.7~22.6	0.242
	Other adverse events	10	8	19	8.6~34.1	6	5	11.9	4.0~25.6	1	1	2.4	0.1~12.6	7	5	11.9	4.0~25.6	0.118
3-5 years (N=60)	Total adverse events	42	26	43.3	30.6~56.8	49	29	48.3	35.2~61.6	39	24	40	27.6~53.5	46	20	33.3	21.7~46.7	0.401
	Systemic adverse events	26	17	28.3	17.5~41.4	32	21	35	23.1~48.4	23	20	33.3	21.7~46.7	27	16	26.7	16.1~39.7	0.722
	Local adverse events	2	2	3.3	0.4~11.5	3	3	5	1.0~13.9	2	2	3.3	0.4~11.5	4	4	6.7	1.8~16.2	0.79
	Other adverse events	14	13	21.7	12.1~34.2	14	11	18.3	9.5~30.4	14	12	20	10.8~32.3	15	8	13.3	5.9~24.6	0.669
3-17 years (N=144)	Total adverse events	91	55	38.2	30.2~46.7	71	45	31.3	23.8~39.5	56	38	26.4	19.4~34.4	84	45	31.3	23.8~39.5	0.195
	Systemic adverse events	38	28	19.4	13.3~26.9	38	27	18.8	12.7~26.1	32	28	19.4	13.3~26.9	37	25	17.4	11.6~24.6	0.965
	Local adverse events	12	11	7.6	3.9~13.3	13	10	6.9	3.4~12.4	9	9	6.3	2.9~11.5	20	15	10.4	5.9~16.6	0.572
	Other adverse events	41	32	22.2	15.7~29.9	20	16	11.1	6.5~17.4	15	13	9	4.9~14.9	27	18	12.5	7.6~19.0	0.006

Note: total adverse events include adverse events at inoculation site (local), adverse events at non inoculation site (systemic) and other adverse events. The low, medium, and high doses represent 2.5μg, 5.0μg, and 10.0 μg/dose, respectively.

Appendix 14. Incidence of adverse reactions within 0-7 days (including 30 minutes) after the full vaccination in subjects aged 3-17 years in phase II clinical trial

		Low dose				Medium dose				High dose				Placebo				*P*
		Case times	Number of cases	Rate(%)	95%CI	Case times	Number of cases	Rate(%)	95%CI	Case times	Number of cases	Rate(%)	95%CI	Case times	Number of cases	Rate(%)	95%CI	
13-17 years (N=42)	Total adverse reactions	8	6	14.3	(5.4~28.5)	9	7	16.7	(7.0~31.4)	12	9	21.4	(10.3~36.8)	12	10	23.8	(12.1~39.5)	0.672
	Systemic adverse reactions	2	2	4.8	(0.6~16.2)	4	4	9.5	(2.7~22.6)	5	4	9.5	(2.7~22.6)	4	4	9.5	(2.7~22.6)	0.817
	Fever	2	2	4.8	(0.6~16.2)	2	2	4.8	(0.6~16.2)	3	3	7.1	(1.5~19.5)	3	3	7.1	(1.5~19.5)	0.935
	Diarrhea	0	0	0	(0.0~8.4)	1	1	2.4	(0.1~12.6)	0	0	0	(0.0~8.4)	1	1	2.4	(0.1~12.6)	>0.999
	Myalgia	0	0	0	(0.0~8.4)	1	1	2.4	(0.1~12.6)	0	0	0	(0.0~8.4)	0	0	0	(0.0~8.4)	>0.999
	Anorexia	0	0	0	(0.0~8.4)	0	0	0	(0.0~8.4)	1	1	2.4	(0.1~12.6)	0	0	0	(0.0~8.4)	>0.999
	Headache	0	0	0	(0.0~8.4)	0	0	0	(0.0~8.4)	1	1	2.4	(0.1~12.6)	0	0	0	(0.0~8.4)	>0.999
	Local adverse reaction	6	5	11.9	(4.0~25.6)	5	4	9.5	(2.7~22.6)	7	7	16.7	(7.0~31.4)	8	7	16.7	(7.0~31.4)	0.715
	Pain	6	5	11.9	(4.0~25.6)	5	4	9.5	(2.7~22.6)	7	7	16.7	(7.0~31.4)	8	7	16.7	(7.0~31.4)	0.715
6-12 years (N=42)	Total adverse reactions	4	4	9.5	(2.7~22.6)	5	3	7.1	(1.5~19.5)	1	1	2.4	(0.1~12.6)	9	4	9.5	(2.7~22.6)	0.541
	Systemic adverse reactions	0	0	0	(0.0~8.4)	0	0	0	(0.0~8.4)	1	1	2.4	(0.1~12.6)	1	1	2.4	(0.1~12.6)	>0.999
	Fever	0	0	0	(0.0~8.4)	0	0	0	(0.0~8.4)	1	1	2.4	(0.1~12.6)	1	1	2.4	(0.1~12.6)	>0.999
	Local adverse reaction	4	4	9.5	(2.7~22.6)	5	3	7.1	(1.5~19.5)	0	0	0	(0.0~8.4)	8	4	9.5	(2.7~22.6)	0.242
	Erythema	1	1	2.4	(0.1~12.6)	2	2	4.8	(0.6~16.2)	0	0	0	(0.0~8.4)	1	1	2.4	(0.1~12.6)	0.562
	Pain	3	3	7.1	(1.5~19.5)	2	2	4.8	(0.6~16.2)	0	0	0	(0.0~8.4)	5	3	7.1	(1.5~19.5)	0.369
	Swelling	0	0	0	(0.0~8.4)	1	1	2.4	(0.1~12.6)	0	0	0	(0.0~8.4)	2	1	2.4	(0.1~12.6)	>0.999
3-5 years (N=60)	Total adverse reactions	10	9	15	(7.1~26.6)	8	8	13.3	(5.9~24.6)	7	7	11.7	(4.8~22.6)	12	10	16.7	(8.3~28.5)	0.877
	Systemic adverse reactions	7	7	11.7	(4.8~22.6)	5	5	8.3	(2.8~18.4)	5	5	8.3	(2.8~18.4)	8	7	11.7	(4.8~22.6)	0.864
	Fever	5	5	8.3	(2.8~18.4)	1	1	1.7	(0.0~8.9)	4	4	6.7	(1.8~16.2)	4	4	6.7	(1.8~16.2)	0.435
	Diarrhea	0	0	0	(0.0~6.0)	0	0	0	(0.0~6.0)	0	0	0	(0.0~6.0)	1	1	1.7	(0.0~8.9)	>0.999
	Cough	2	2	3.3	(0.4~11.5)	4	4	6.7	(1.8~16.2)	1	1	1.7	(0.0~8.9)	3	3	5	(1.0~13.9)	0.555
	Local adverse reaction	2	2	3.3	(0.4~11.5)	3	3	5	(1.0~13.9)	2	2	3.3	(0.4~11.5)	4	4	6.7	(1.8~16.2)	0.79
	Erythema	0	0	0	(0.0~6.0)	1	1	1.7	(0.0~8.9)	0	0	0	(0.0~6.0)	1	1	1.7	(0.0~8.9)	>0.999

	Pain	1	1	1.7	(0.0~8.9)	2	2	3.3	(0.4~11.5)	1	1	1.7	(0.0~8.9)	3	3	5	(1.0~13.9)	0.655
	Swelling	1	1	1.7	(0.0~8.9)	0	0	0	(0.0~6.0)	1	1	1.7	(0.0~8.9)	0	0	0	(0.0~6.0)	>0.999
	Other adverse reactions	1	1	1.7	(0.0~8.9)	0	0	0	(0.0~6.0)	0	0	0	(0.0~6.0)	0	0	0	(0.0~6.0)	>0.999
	Abdominal pain	1	1	1.7	(0.0~8.9)	0	0	0	(0.0~6.0)	0	0	0	(0.0~6.0)	0	0	0	(0.0~6.0)	>0.999
3-17 years (N=144)	Total adverse reactions	22	19	13.2	(8.1~19.8)	22	18	12.5	(7.6~19.0)	20	17	11.8	(7.0~18.2)	33	24	16.7	(11.0~23.8)	0.632
	Systemic adverse reactions	9	9	6.3	(2.9~11.5)	9	9	6.3	(2.9~11.5)	11	10	6.9	(3.4~12.4)	13	12	8.3	(4.4~14.1)	0.886
	Fever	7	7	4.9	(2.0~9.8)	3	3	2.1	(0.4~6.0)	8	8	5.6	(2.4~10.7)	8	8	5.6	(2.4~10.7)	0.434
	Diarrhea	0	0	0	(0.0~2.5)	1	1	0.7	(0.0~3.8)	0	0	0	(0.0~2.5)	2	2	1.4	(0.2~4.9)	0.623
	Myalgia	0	0	0	(0.0~2.5)	1	1	0.7	(0.0~3.8)	0	0	0	(0.0~2.5)	0	0	0	(0.0~2.5)	>0.999
	Cough	2	2	1.4	(0.2~4.9)	4	4	2.8	(0.8~7.0)	1	1	0.7	(0.0~3.8)	3	3	2.1	(0.4~6.0)	0.565
	Anorexia	0	0	0	(0.0~2.5)	0	0	0	(0.0~2.5)	1	1	0.7	(0.0~3.8)	0	0	0	(0.0~2.5)	>0.999
	Headache	0	0	0	(0.0~2.5)	0	0	0	(0.0~2.5)	1	1	0.7	(0.0~3.8)	0	0	0	(0.0~2.5)	>0.999
	Local adverse reaction	12	11	7.6	(3.9~13.3)	13	10	6.9	(3.4~12.4)	9	9	6.3	(2.9~11.5)	20	15	10.4	(5.9~16.6)	0.572
	Erythema	1	1	0.7	(0.0~3.8)	3	3	2.1	(0.4~6.0)	0	0	0	(0.0~2.5)	2	2	1.4	(0.2~4.9)	0.338
	Pain	10	9	6.3	(2.9~11.5)	9	8	5.6	(2.4~10.7)	8	8	5.6	(2.4~10.7)	16	13	9	(4.9~14.9)	0.59
	Swelling	1	1	0.7	(0.0~3.8)	1	1	0.7	(0.0~3.8)	1	1	0.7	(0.0~3.8)	2	1	0.7	(0.0~3.8)	>0.999
	Other adverse reactions	1	1	0.7	(0.0~3.8)	0	0	0	(0.0~2.5)	0	0	0	(0.0~2.5)	0	0	0	(0.0~2.5)	>0.999
	Abdominal pain	1	1	0.7	(0.0~3.8)	0	0	0	(0.0~2.5)	0	0	0	(0.0~2.5)	0	0	0	(0.0~2.5)	>0.999

Note: total adverse reactions include inoculation site (local) adverse reactions, non inoculation site (systemic) adverse reactions and other adverse reactions. The low, medium, and high doses represent 2.5μg, 5.0μg, and 10.0 μg/dose, respectively.

Appendix 15. Severity of adverse reactions of phase II subjects aged 3-17 years after whole course vaccination (based on cases)

Symptom	Age group	Group	Total	Level 1	Level 2	Level 3
Total adverse reactions	13-17 years	Low dose	8	8	0	0
		Medium dose	9	9	0	0
		High dose	12	9	3	0
		Placebo	12	12	0	0
	6-12 years	Low dose	4	4	0	0
		Medium dose	5	3	2	0
		High dose	1	0	1	0
		Placebo	9	8	1	0
	3-5 years	Low dose	10	4	6	0
		Medium dose	8	5	3	0
		High dose	7	5	2	0
		Placebo	12	9	3	0
	3-17 years	Low dose	22	16	6	0
		Medium dose	22	17	5	0
		High dose	20	14	6	0
		Placebo	33	29	4	0
Local adverse reaction						
	6-12 years	Low dose	1	1	0	0
		Medium dose	2	1	1	0
		High dose	0	0	0	0
		Placebo	1	0	1	0
	3-5 years	Low dose	0	0	0	0
		Medium dose	1	1	0	0
		High dose	0	0	0	0
		Placebo	1	1	0	0
	3-17 years	Low dose	1	1	0	0
		Medium dose	3	2	1	0
		High dose	0	0	0	0
		Placebo	2	1	1	0
Pain at vaccination site	13-17 years	Low dose	6	6	0	0
		Medium dose	5	5	0	0

		High dose	7	7	0	0
		Placebo	8	8	0	0
	6-12 years	Low dose	3	3	0	0
		Medium dose	2	2	0	0
		High dose	0	0	0	0
		Placebo	5	5	0	0
	3-5 years	Low dose	1	1	0	0
		Medium dose	2	2	0	0
		High dose	1	1	0	0
		Placebo	3	3	0	0
	3-17 years	Low dose	10	10	0	0
		Medium dose	9	9	0	0
		High dose	8	8	0	0
		Placebo	16	16	0	0
Swelling of vaccination site	6-12 years	Low dose	0	0	0	0
		Medium dose	1	0	1	0
		High dose	0	0	0	0
		Placebo	2	2	0	0
	3-5 years	Low dose	1	0	1	0
		Medium dose	0	0	0	0
		High dose	1	1	0	0
		Placebo	0	0	0	0
	3-17 years	Low dose	1	0	1	0
		Medium dose	0	0	0	0
		High dose	1	1	0	0
		Placebo	0	0	0	0
Systemic adverse reactions						
Fever	13-17 years	Low dose	2	2	0	0
		Medium dose	2	2	0	0
		High dose	3	2	1	0
		Placebo	3	3	0	0
	6-12 years	Low dose	0	0	0	0
		Medium dose	0	0	0	0
		High dose	1	0	1	0

		Placebo	1	1	0	0
	3-5 years	Low dose	5	1	4	0
		Medium dose	1	0	1	0
		High dose	4	2	2	0
		Placebo	4	1	3	0
	3-17 years	Low dose	7	3	4	0
		Medium dose	3	2	1	0
		High dose	8	4	4	0
		Placebo	8	5	3	0
	13-17 years	Low dose	0	0	0	0
		Medium dose	1	1	0	0
		High dose	0	0	0	0
		Placebo	1	1	0	0
Diarrhea	3-5 years	Low dose	0	0	0	0
		Medium dose	0	0	0	0
		High dose	0	0	0	0
		Placebo	1	1	0	0
	3-17 years	Low dose	0	0	0	0
		Medium dose	1	1	0	0
		High dose	0	0	0	0
		Placebo	2	2	0	0
	3-5 years	Low dose	2	1	1	0
		Medium dose	4	2	2	0
		High dose	1	1	0	0
		Placebo	3	3	0	0
Cough	3-17 years	Low dose	2	1	1	0
		Medium dose	4	2	2	0
		High dose	1	1	0	0
		Placebo	3	3	0	0
	13-17 years	Low dose	0	0	0	0
		Medium dose	1	1	0	0
Myalgia		High dose	0	0	0	0
		Placebo	0	0	0	0
	3-17 years	Low dose	0	0	0	0

		Medium dose	1	1	0	0
		High dose	0	0	0	0
		Placebo	0	0	0	0
	13-17 years	Low dose	0	0	0	0
		Medium dose	0	0	0	0
		High dose	1	0	1	0
Anorexia		Placebo	0	0	0	0
	3-17 years	Low dose	0	0	0	0
		Medium dose	0	0	0	0
		High dose	1	0	1	0
		Placebo	0	0	0	0
	13-17 years	Low dose	0	0	0	0
		Medium dose	0	0	0	0
		High dose	1	0	1	0
Headache		Placebo	0	0	0	0
	3-17 years	Low dose	0	0	0	0
		Medium dose	0	0	0	0
		High dose	1	0	1	0
		Placebo	0	0	0	0
Other adverse reactions						
	3-5 years	Low dose	1	1	0	0
		Medium dose	0	0	0	0
		High dose	0	0	0	0
Abdominal pain		Placebo	0	0	0	0
	3-17 years	Low dose	1	1	0	0
		Medium dose	0	0	0	0
		High dose	0	0	0	0
		Placebo	0	0	0	0

The low, medium, and high doses represent 2.5μg, 5.0μg, and 10.0 μg/dose, respectively.

Appendix 16. Incidence of serious adverse events in subjects aged 3-17 years years in phase II clinical trial

SOC	PT	Age group	Group	N	Case times	Number of cases	Rate	(95%CI)
**Infectious and infectious diseases**	Total	13-17 years	Low dose	42	0	0	0.0	(0.0~8.4)
			Medium dose	42	0	0	0.0	(0.0~8.4)
			High dose	42	0	0	0.0	(0.0~8.4)
			Placebo	42	0	0	0.0	(0.0~8.4)
		6-12 years	Low dose	42	0	0	0.0	(0.0~8.4)
			Medium dose	42	0	0	0.0	(0.0~8.4)
			High dose	42	0	0	0.0	(0.0~8.4)
			Placebo	42	0	0	0.0	(0.0~8.4)
		3-5 years	Low dose	60	0	0	0.0	(0.0~6.0)
			Medium dose	60	3	2	3.3	(0.4~11.5)
			High dose	60	2	2	3.3	(0.4~11.5)
			Placebo	60	1	1	1.7	(0.0~8.9)
		**3-17 years**	Low dose	144	0	0	0.0	(0.0~2.5)
			Medium dose	144	3	2	1.4	(0.2~4.9)
			High dose	144	2	2	1.4	(0.2~4.9)
			Placebo	144	1	1	0.7	(0.0~3.8)
	Infectious pneumonia	13-17 years	Low dose	42	0	0	0.0	(0.0~8.4)
			Medium dose	42	0	0	0.0	(0.0~8.4)
			High dose	42	0	0	0.0	(0.0~8.4)
			Placebo	42	0	0	0.0	(0.0~8.4)
		6-12 years	Low dose	42	0	0	0.0	(0.0~8.4)
			Medium dose	42	0	0	0.0	(0.0~8.4)
			High dose	42	0	0	0.0	(0.0~8.4)
			Placebo	42	0	0	0.0	(0.0~8.4)
		3-5 years	Low dose	60	0	0	0.0	(0.0~6.0)
			Medium dose	60	1	1	1.7	(0.0~8.9)
			High dose	60	1	1	1.7	(0.0~8.9)
			Placebo	60	1	1	1.7	(0.0~8.9)

	**3-17 years**	Low dose	144	0	0	0.0	(0.0~2.5)
		Medium dose	144	1	1	0.7	(0.0~3.8)
		High dose	144	1	1	0.7	(0.0~3.8)
		Placebo	144	1	1	0.7	(0.0~3.8)
Chronic tonsillitis	13-17 years	Low dose	42	0	0	0.0	(0.0~8.4)
		Medium dose	42	0	0	0.0	(0.0~8.4)
		High dose	42	0	0	0.0	(0.0~8.4)
		Placebo	42	0	0	0.0	(0.0~8.4)
	6-12 years	Low dose	42	0	0	0.0	(0.0~8.4)
		Medium dose	42	0	0	0.0	(0.0~8.4)
		High dose	42	0	0	0.0	(0.0~8.4)
		Placebo	42	0	0	0.0	(0.0~8.4)
	3-5 years	Low dose	60	0	0	0.0	(0.0~6.0)
		Medium dose	60	1	1	1.7	(0.0~8.9)
		High dose	60	0	0	0.0	(0.0~6.0)
		Placebo	60	0	0	0.0	(0.0~6.0)
	**3-17 years**	Low dose	144	0	0	0.0	(0.0~2.5)
		Medium dose	144	1	1	0.7	(0.0~3.8)
		High dose	144	0	0	0.0	(0.0~2.5)
		Placebo	144	0	0	0.0	(0.0~2.5)
Bronchitis	13-17 years	Low dose	42	0	0	0.0	(0.0~8.4)
		Medium dose	42	0	0	0.0	(0.0~8.4)
		High dose	42	0	0	0.0	(0.0~8.4)
		Placebo	42	0	0	0.0	(0.0~8.4)
	6-12 years	Low dose	42	0	0	0.0	(0.0~8.4)
		Medium dose	42	0	0	0.0	(0.0~8.4)
		High dose	42	0	0	0.0	(0.0~8.4)
		Placebo	42	0	0	0.0	(0.0~8.4)
	3-5 years	Low dose	60	0	0	0.0	(0.0~6.0)
		Medium dose	60	0	0	0.0	(0.0~6.0)

		High dose	60	1	1	1.7	(0.0~8.9)
		Placebo	60	0	0	0.0	(0.0~6.0)
	**3-17 years**	Low dose	144	0	0	0.0	(0.0~2.5)
		Medium dose	144	0	0	0.0	(0.0~2.5)
		High dose	144	1	1	0.7	(0.0~3.8)
		Placebo	144	0	0	0.0	(0.0~2.5)
Pharyngitis	13-17 years	Low dose	42	0	0	0.0	(0.0~8.4)
		Medium dose	42	0	0	0.0	(0.0~8.4)
		High dose	42	0	0	0.0	(0.0~8.4)
		Placebo	42	0	0	0.0	(0.0~8.4)
	6-12 years	Low dose	42	0	0	0.0	(0.0~8.4)
		Medium dose	42	0	0	0.0	(0.0~8.4)
		High dose	42	0	0	0.0	(0.0~8.4)
		Placebo	42	0	0	0.0	(0.0~8.4)
	3-5 years	Low dose	60	0	0	0.0	(0.0~6.0)
		Medium dose	60	1	1	1.7	(0.0~8.9)
		High dose	60	0	0	0.0	(0.0~6.0)
		Placebo	60	0	0	0.0	(0.0~6.0)
	**3-17 years**	Low dose	144	0	0	0.0	(0.0~2.5)
		Medium dose	144	1	1	0.7	(0.0~3.8)
		High dose	144	0	0	0.0	(0.0~2.5)
		Placebo	144	0	0	0.0	(0.0~2.5)
Various injuries, poisoning and surgical complications	13-17 years	Low dose	42	0	0	0.0	(0.0~8.4)
		Medium dose	42	0	0	0.0	(0.0~8.4)
		High dose	42	0	0	0.0	(0.0~8.4)
		Placebo	42	0	0	0.0	(0.0~8.4)
	6-12 years	Low dose	42	0	0	0.0	(0.0~8.4)
		Medium dose	42	0	0	0.0	(0.0~8.4)
		High dose	42	0	0	0.0	(0.0~8.4)
		Placebo	42	1	1	2.4	(0.1~12.6)

		3-5 years	Low dose	60	0	0	0.0	(0.0~6.0)
			Medium dose	60	0	0	0.0	(0.0~6.0)
			High dose	60	0	0	0.0	(0.0~6.0)
			Placebo	60	0	0	0.0	(0.0~6.0)
		3-17 years	Low dose	144	0	0	0.0	(0.0~2.5)
			Medium dose	144	0	0	0.0	(0.0~2.5)
			High dose	144	0	0	0.0	(0.0~2.5)
			Placebo	144	1	1	0.7	(0.0~3.8)
	Forearm fracture	13-17 years	Low dose	42	0	0	0.0	(0.0~8.4)
			Medium dose	42	0	0	0.0	(0.0~8.4)
			High dose	42	0	0	0.0	(0.0~8.4)
			Placebo	42	0	0	0.0	(0.0~8.4)
		6-12 years	Low dose	42	0	0	0.0	(0.0~8.4)
			Medium dose	42	0	0	0.0	(0.0~8.4)
			High dose	42	0	0	0.0	(0.0~8.4)
			Placebo	42	1	1	2.4	(0.1~12.6)
		3-5 years	Low dose	60	0	0	0.0	(0.0~6.0)
			Medium dose	60	0	0	0.0	(0.0~6.0)
			High dose	60	0	0	0.0	(0.0~6.0)
			Placebo	60	0	0	0.0	(0.0~6.0)
		**3-17 years**	Low dose	144	0	0	0.0	(0.0~2.5)
			Medium dose	144	0	0	0.0	(0.0~2.5)
			High dose	144	0	0	0.0	(0.0~2.5)
			Placebo	144	1	1	0.7	(0.0~3.8)
Kidney and urinary diseases	Summary	13-17 years	Low dose	42	0	0	0.0	(0.0~8.4)
			Medium dose	42	0	0	0.0	(0.0~8.4)
			High dose	42	0	0	0.0	(0.0~8.4)
			Placebo	42	0	0	0.0	(0.0~8.4)
		6-12 years	Low dose	42	0	0	0.0	(0.0~8.4)
			Medium dose	42	0	0	0.0	(0.0~8.4)

		High dose	42	0	0	0.0	(0.0~8.4)
		Placebo	42	0	0	0.0	(0.0~8.4)
	3-5 years	Low dose	60	0	0	0.0	(0.0~6.0)
		Medium dose	60	0	0	0.0	(0.0~6.0)
		High dose	60	1	1	1.7	(0.0~8.9)
		Placebo	60	0	0	0.0	(0.0~6.0)
	**3-17 years**	Low dose	144	0	0	0.0	(0.0~2.5)
		Medium dose	144	0	0	0.0	(0.0~2.5)
		High dose	144	1	1	0.7	(0.0~3.8)
		Placebo	144	0	0	0.0	(0.0~2.5)
Nephrotic syndrome	13-17 years	Low dose	42	0	0	0.0	(0.0~8.4)
		Medium dose	42	0	0	0.0	(0.0~8.4)
		High dose	42	0	0	0.0	(0.0~8.4)
		Placebo	42	0	0	0.0	(0.0~8.4)
	6-12 years	Low dose	42	0	0	0.0	(0.0~8.4)
		Medium dose	42	0	0	0.0	(0.0~8.4)
		High dose	42	0	0	0.0	(0.0~8.4)
		Placebo	42	0	0	0.0	(0.0~8.4)
	3-5 years	Low dose	60	0	0	0.0	(0.0~6.0)
		Medium dose	60	0	0	0.0	(0.0~6.0)
		High dose	60	1	1	1.7	(0.0~8.9)
		Placebo	60	0	0	0.0	(0.0~6.0)
	**3-17 years**	Low dose	144	0	0	0.0	(0.0~2.5)
		Medium dose	144	0	0	0.0	(0.0~2.5)
		High dose	144	1	1	0.7	(0.0~3.8)
		Placebo	144	0	0	0.0	(0.0~2.5)

The low, medium, and high doses represent 2.5μg, 5.0μg, and 10.0 μg/dose, respectively.

Appendix 17. Antibody GMT (95% CI) of subjects aged 3-17 years at different time points in phase II clinical trial（FAS）

Age group	point of time	Low dose		Medium dose		High dose		Placebo		*P*
		N	GMT(95%CI)	N	GMT(95%CI)	N	GMT(95%CI)	N	GMT(95%CI)	
13-17 years	**Neutralizing Antibodies to Live SARS-CoV-2**									
	Before 1st dose immunization	42	5(5~5)	42	5(5~5)	42	5(5~5)	42	5(5~5)	
	28 days after the second dose of immunization	42	67.4(50.1~180.8)	42	74(55~99.7)	41	105.6(78.1~142.7)	42	5(3.7~6.7)	<0.001
	28 days after the third dose of immunization	42	112.1(79~159)	42	125.1(88.2~177.4)	41	242.9(170.5~345.9)	42	5(3.5~7.1)	<0.001
	**Specific Antibody Responses to SARS-CoV-2**									
	Before 1st dose immunization	42	10(10~10)	42	10(10~10)	42	10(10~10)	42	10(10~10)	
	28 days after the second dose of immunization	42	124.9(105~148.6)	42	124.9(105~148.6)	41	162.7(136.5~194)	42	10(8.4~11.9)	<0.001
	28 days after the third dose of immunization	42	280.4(234.5~335.3)	42	254(212.4~303.7)	41	325.5(271.6~3180)	42	10(8.4~12)	<0.001
6-12 years	**Neutralizing Antibodies to Live SARS-CoV-2**									
	Before 1st dose immunization	42	5(5~5)	42	5(5~5)	42	5(5~5)	42	5(5~5)	
	28 days after the second dose of immunization	42	111.5(87.5~142)	42	192.1(150.8~244.7)	41	229.6(179.8~293.3)	42	5(3.9~6.4)	<0.001
	28 days after the third dose of immunization	42	340.1(269.9~428.7)	42	4180.7(389.4~618.4)	41	635.1(502.5~802.6)	42	5(4~6.3)	<0.001
	**Specific Antibody Responses to SARS-CoV-2**									
	Before 1st dose immunization	42	10(10~10)	42	10(10~10)	42	10(10~10)	42	10(10~10)	
	28 days after the second dose of immunization	42	241.7(205.5~284.3)	42	211.8(180.1~249.1)	42	320(271.5~377.1)	42	10(8.5~11.8)	<0.001
	28 days after the third dose of immunization	42	542.6(439.8~669.5)	42	460.1(372.9~567.6)	41	505.1(408.4~624.8)	42	10(8.1~12.3)	<0.001
3-5 years	**Neutralizing Antibodies to Live SARS-CoV-2**									
	Before 1st dose immunization	60	5(5~5)	60	5(5~5)	60	5(5~5)	60	5(5~5)	
	28 days after the second dose of immunization	60	267.2(219.9~324.6)	59	273.4(224.6~332.7)	57	3180.6(319.9~477)	58	5(4.1~6.1)	<0.001
	28 days after the third dose of immunization	60	980.5(818.8~1174.1)	59	954.4(795.8~1144.6)	58	793(660.2~952.5)	59	5(4.2~6)	<0.001
	**Specific Antibody Responses to SARS-CoV-2**									
	Before 1st dose immunization	60	10(10~10)	60	10(10~10)	60	10(10~10)	60	10(10~10)	

	28 days after the second dose of immunization	60	298.6(257.8~345.8)	59	291.3(251.2~337.8)	57	323.9(278.6~376.5)	58	10(8.6~11.6)	<0.001
	28 days after the third dose of immunization	60	815.7(658.7~1010.2)	59	640(515.9~794)	58	984.1(791.7~1223.2)	59	10(8.1~12.4)	<0.001
	**Neutralizing Antibodies to Live SARS-CoV-2**									
	Before 1st dose immunization	144	5(5~5)	144	5(5~5)	144	5(5~5)	144	5(5~5)	
	28 days after the second dose of immunization	144	138.6(118.3~162.3)	143	167.9(143.3~196.8)	139	227(193.3~266.7)	142	5(4.3~5.9)	<0.001
	28 days after the third dose of immunization	144	382.5(319.7~457.6)	143	432.2(361~517.5)	140	525.5(438.1~630.3)	143	5(4.2~6)	<0.001
3-17 years	**Specific Antibody Responses to SARS-CoV-2**									
	Before 1st dose immunization	144	10(10~10)	144	10(10~10)	144	10(10~10)	144	10(10~10)	
	28 days after the second dose of immunization	144	217.7(196.3~241.5)	143	206.9(186.4~229.5)	139	263.4(237.1~292.8)	142	10(9~11.1)	<0.001
	28 days after the third dose of immunization	144	530.5(464.1~606.3)	143	442.8(387.2~506.3)	140	585.4(511.2~670.4)	143	10(8.7~11.4)	<0.001

The low, medium, and high doses represent 2.5μg, 5.0μg, and 10.0 μg/dose, respectively.

Appendix 18. Antibody GMT (95% CI) of phase II subjects aged 3-17 years at different time points（PPS）

Age group	point of time	Low dose		Medium dose		High dose		Placebo		*P*
		N	GMT(95%CI)	N	GMT(95%CI)	N	GMT(95%CI)	N	GMT(95%CI)	
13-17 years	**Neutralizing Antibodies to Live SARS-CoV-2**									
	Before 1st dose immunization	42	5(5~5)	42	5(5~5)	42	5(5~5)	42	5(5~5)	
	28 days after the second dose of immunization	42	67.4(50.1~180.8)	42	74.0(55.0~99.7)	41	105.6(78.1~142.7)	42	5.0 (3.7~6.7)	<0.001
	28 days after the third dose of immunization	42	112.1(79.0~159.0)	42	125.1(88.2~177.4)	41	242.9(170.5~345.9)	42	5.0 (3.5~7.1)	<0.001
	**Specific Antibody Responses to SARS-CoV-2**									
	Before 1st dose immunization	42	10(10~10)	42	10(10~10)	42	10(10~10)	42	10(10~10)	
	28 days after the second dose of immunization	42	124.9(105.0~148.6)	42	124.9(105.0~148.6)	41	162.7(136.5~194.0)	42	10.0 (8.4~11.9)	<0.001
	28 days after the third dose of immunization	42	280.4(234.5~335.3)	42	254.0 (212.4~303.7)	41	325.5(271.6~3180.0)	42	10.0(8.4~12.0)	<0.001
6-12 years	**Neutralizing Antibodies to Live SARS-CoV-2**									
	Before 1st dose immunization	42	5(5~5)	42	5(5~5)	42	5(5~5)	42	5(5~5)	
	28 days after the second dose of immunization	42	111.5(87.5~142)	42	192.1(150.8~244.7)	41	229.6(179.8~293.3)	42	5(3.9~6.4)	<0.001
	28 days after the third dose of immunization	42	340.1(269.9~428.7)	42	4180.7(389.4~618.4)	41	635.1(502.5~802.6)	42	5(4~6.3)	<0.001
	**Specific Antibody Responses to SARS-CoV-2**									
	Before 1st dose immunization	42	10(10~10)	42	10(10~10)	42	10(10~10)	42	10(10~10)	
	28 days after the second dose of immunization	42	241.7(205.5~284.3)	42	211.8(180.1~249.1)	41	320(271.5~377.1)	42	10(8.5~11.8)	<0.001
	28 days after the third dose of immunization	42	542.6(439.8~669.5)	42	460.1(372.9~567.6)	41	505.1(408.4~624.8)	42	10(8.1~12.3)	<0.001
3-5 years	**Neutralizing Antibodies to Live SARS-CoV-2**									
	Before 1st dose immunization	60	5(5~5)	60	5(5~5)	60	5(5~5)	60	5(5~5)	
	28 days after the second dose of immunization	60	267.2(219.8~324.7)	59	273.4(224.5~332.8)	57	3180.6(319.7~477.2)	57	5(4.1~6.1)	<0.001
	28 days after the third dose of immunization	60	980.5(820.9~1171.1)	59	954.4(797.8~1141.6)	55	815.7(677.5~982)	59	5(4.2~6)	<0.001
	**Specific Antibody Responses to SARS-CoV-2**									
	Before 1st dose immunization	60	10(10~10)	60	10(10~10)	60	10(10~10)	60	10(10~10)	

	28 days after the second dose of immunization	60	298.6(257.7~345.9)	59	291.3(251.1~337.9)	57	323.9(278.6~376.7)	57	10(8.6~11.6)	<0.001
	28 days after the third dose of immunization	60	815.7(661.3~1006.2)	59	640(517.9~7180.9)	55	1059.5(850.9~1319.2)	59	10(8.1~12.4)	<0.001
3-17 years	**Neutralizing Antibodies to Live SARS-CoV-2**									
	Before 1st dose immunization	144	5(5~5)	144	5(5~5)	144	5(5~5)	144	5(5~5)	
	28 days after the second dose of immunization	144	138.6(118.3~162.3)	143	167.9(143.3~196.8)	139	227(193.3~266.7)	141	5(4.3~5.9)	<0.001
	28 days after the third dose of immunization	144	382.5(319.7~457.6)	143	432.2(361~517.4)	137	526.7(438.2~633)	143	5(4.2~6)	<0.001
	**Specific Antibody Responses to SARS-CoV-2**									
	Before 1st dose immunization	144	10(10~10)	144	10(10~10)	144	10(10~10)	144	10(10~10)	
	28 days after the second dose of immunization	144	217.7(196.3~241.5)	143	206.9(186.4~229.6)	139	263.4(237~292.8)	141	10(9~11.1)	<0.001
	28 days after the third dose of immunization	144	530.5(464.3~606.1)	143	442.8(387.4~506.1)	137	596.2(520.1~683.5)	143	10(8.7~11.4)	<0.001

The low, medium, and high doses represent 2.5μg, 5.0μg, and 10.0 μg/dose, respectively.

Appendix 19. Comparison of antibody levels of phase II subjects aged 3-17 years 28 days after the full vaccination（FAS）

Age group	Antibody type	Low dose (N =42)	Medium dose (N =42)	High dose (N =41)	Placebo (N =42)
13-17 years	**Neutralizing Antibodies to Live SARS-CoV-2**				
	GMT (95% CI)	112.1(79~159)	125.1(88.2~177.4)	242.9(170.5~345.9)	5(3.5~7.1)
	GMI (95% CI)	1	1.12(0.68~1.83)	2.17(1.32~3.56)	-
	Antibody 4-fold growth rate— % (95% CI)	85.7(71.5~94.6)	180.5(77.4~97.3)	97.6(87.1~99.9)	0.0
	**Specific Antibody Responses to SARS-CoV-2**				
	GMT (95% CI)	280.4(234.5~335.3)	254(212.4~303.7)	325.5(271.6~3180)	10(8.4~12)
	GMI (95% CI)	1	0.91(0.7~1.17)	1.16(0.9~1.5)	-
	Antibody 4-fold growth rate — % (95% CI)	100.0	100.0	100.0	0.0
6-12 years	Antibody type	Low dose (N =42)	Medium dose (N =42)	High dose (N = 41)	Placebo (N = 42)
	**Neutralizing Antibodies to Live SARS-CoV-2**				
	GMT (95% CI)	340.1(269.9~428.7)	4180.7(389.4~618.4)	635.1(502.5~802.6)	5(4~6.3)
	GMI (95% CI)	1	1.44(1.04~2)	1.87(1.34~2.59)	-
	Antibody 4-fold growth rate— % (95% CI)	97.6(87.4~99.9)	100.0	100.0	0.0
	**Specific Antibody Responses to SARS-CoV-2**				
	GMT (95% CI)	542.6(439.8~669.5)	460.1(372.9~567.6)	505.1(408.4~624.8)	10(8.1~12.3)
	GMI (95% CI)	1	0.85(0.63~1.14)	0.93(0.69~1.26)	-
	Antibody 4-fold growth rate — % (95% CI)	100.0	97.6(87.4~99.9)	100.0	0.0
3-5 years	Antibody type	Low dose (N =60)	Medium dose (N =59)	High dose (N =58)	Placebo (N =59)
	**Neutralizing Antibodies to Live SARS-CoV-2**				
	GMT (95% CI)	980.5(818.8~1174.1)	954.4(795.8~1144.6)	793(660.2~952.5)	5(4.2~6)

	GMI (95% CI)	1	0.97(0.75~1.26)	0.81(0.63~1.05)	-
	Antibody 4-fold growth rate— % (95% CI)	100.0	100.0	100.0	0.0
	**Specific Antibody Responses to SARS-CoV-2**				
	GMT (95% CI)	815.7(658.7~1010.2)	640(515.9~794)	984.1(791.7~1223.2)	10(8.1~12.4)
	GMI (95% CI)	1	0.78(0.58~1.06)	1.21(0.89~1.64)	-
	Antibody 4-fold growth rate — % (95% CI)	100.0	100.0	100.0	0.0

	Antibody type	Low dose (N = 144)	Medium dose (N =143)	High dose (N =140)	Placebo (N = 143)
3-17 years	**Neutralizing Antibodies to Live SARS-CoV-2**				
	GMT (95% CI)	382.5(319.7~457.6)	432.2(361~517.5)	525.5(438.1~630.3)	5(4.2~6)
	GMI (95% CI)	1	1.13(0.88~1.46)	1.37(1.06~1.77)	-
	Antibody 4-fold growth rate— % (95% CI)	95.1(180.2~98)	97.2(93~99.2)	99.3(96.1~100)	0.0
	**Specific Antibody Responses to SARS-CoV-2**				
	GMT (95% CI)	530.5(464.1~606.3)	442.8(387.2~506.3)	585.4(511.2~670.4)	10(8.7~11.4)
	GMI (95% CI)	1	0.83(0.69~1.01)	1.1(0.91~1.34)	-
	Antibody 4-fold growth rate — % (95% CI)	100.0	99.3(96.2~100)	100.0	0.0

The low, medium, and high doses represent 2.5μg, 5.0μg, and 10.0 μg/dose, respectively.

Appendix 20. Comparison of antibody levels of subjects aged 3-17 years in phase II clinical trial 28 days after the full vaccination（PPS）

Age group	Antibody type	Low dose (N = 42)	Medium dose (N = 42)	High dose (N = 41)	Placebo (N =42)
13-17 years	**Neutralizing Antibodies to Live SARS-CoV-2**				
	GMT (95% CI)	112.1(79.0~159.0)	125.1(88.2~177.4)	242.9(170.5~345.9)	5.0 (3.5~7.1)
	GMI (95% CI)	1	1.12(0.68~1.83)	2.17(1.32~3.56)	-
	Antibody 4-fold growth rate— % (95% CI)	85.7(71.5~94.6)	180.5(77.4~97.3)	97.6(87.1~99.9)	0.0
	**Specific Antibody Responses to SARS-CoV-2**				
	GMT (95% CI)	280.4(234.5~335.3)	254.0 (212.4~303.7)	325.5(271.6~3180.0)	10.0(8.4~12.0)
	GMI (95% CI)	1	0.91(0.7~1.17)	1.16(0.9~1.5)	-
	Antibody 4-fold growth rate — % (95% CI)	100.0	100.0	100.0	0.0
6-12 years	Antibody type	Low dose (N =42)	Medium dose (N =42)	High dose (N = 41)	Placebo (N =42)
	**Neutralizing Antibodies to Live SARS-CoV-2**				
	GMT (95% CI)	340.1(269.9~428.7)	4180.7(389.4~618.4)	635.1(502.5~802.6)	5(4~6.3)
	GMI (95% CI)	1	1.44(1.04~2)	1.87(1.34~2.59)	-
	Antibody 4-fold growth rate— % (95% CI)	97.6(87.4~99.9)	100.0	100.0	0.0
	**Specific Antibody Responses to SARS-CoV-2**				
	GMT (95% CI)	542.6(439.8~669.5)	460.1(372.9~567.6)	505.1(408.4~624.8)	10(8.1~12.3)
	GMI (95% CI)	1	0.85(0.63~1.14)	0.93(0.69~1.26)	-
	Antibody 4-fold growth rate — % (95% CI)	100.0	97.6(87.4~99.9)	100.0	0.0
3-5 years	Antibody type	Low dose (N =60)	Medium dose (N = 59)	High dose (N = 55)	Placebo (N =59)
	**Neutralizing Antibodies to Live SARS-CoV-2**				
	GMT (95% CI)	980.5(820.9~1171.1)	954.4(797.8~1141.6)	815.7(677.5~982)	5(4.2~6)
	GMI (95% CI)	1	0.97(0.76~1.25)	0.83(0.64~1.08)	-

	Antibody type	Low dose	Medium dose	High dose	Placebo
	Antibody 4-fold growth rate— % (95% CI)	100.0	100.0	100.0	0.0
	**Specific Antibody Responses to SARS-CoV-2**				
	GMT (95% CI)	815.7(661.3~1006.2)	640(517.9~7180.9)	1059.5(850.9~1319.2)	10(8.1~12.4)
	GMI (95% CI)	1	0.78(0.58~1.06)	1.3(0.96~1.76)	-
	Antibody 4-fold growth rate — % (95% CI)	100.0	100.0	100.0	0.0
	Antibody type	Low dose (N =144)	Medium dose (N =143)	High dose (N =137)	Placebo (N =143)
	**Neutralizing Antibodies to Live SARS-CoV-2**				
	GMT (95% CI)	382.5(319.7~457.6)	432.2(361~517.4)	526.7(438.2~633)	5(4.2~6)
	GMI (95% CI)	1	1.13(0.88~1.46)	1.38(1.07~1.78)	-
3-17 years	Antibody 4-fold growth rate— % (95% CI)	95.1(180.2~98.0)	97.2(93.0~99.2)	99.3(96.0~100.0)	0.0
	**Specific Antibody Responses to SARS-CoV-2**				
	GMT (95% CI)	530.5(464.3~606.1)	442.8(387.4~506.1)	596.2(520.1~683.5)	10(8.7~11.4)
	GMI (95% CI)	1	0.83(0.69~1.01)	1.12(0.93~1.36)	-
	Antibody 4-fold growth rate — % (95% CI)	100.0	99.3(96.2~100)	100.0	0.0

The low, medium, and high doses represent 2.5μg, 5.0μg, and 10.0 μg/dose, respectively.

\

Appendix 21. Quadruple growth rate of antibody in subjects aged 3-17 years in phase II at different time points (95% CI)（FAS）

Age group	point of time	Low dose			Medium dose			High dose			Placebo			*P*
		N	Antibody 4-fold growth rate	Rate(95%CI)	N	Antibody 4-fold growth rate	Rate(95%CI)	N	Antibody 4-fold growth rate	Rate(95%CI)	N	Antibody 4-fold growth rate	Rate(95%CI)	
13-17 years	**Neutralizing Antibodies to Live SARS-CoV-2**													
	28 days after the second dose of immunization	42	36	85.7(71.5~94.6)	42	37	88.1(74.4~96.0)	41	39	95.1(83.5~99.4)	42	0	0.0	<0.001
	28 days after the third dose of immunization	42	36	85.7(71.5~94.6)	42	38	180.5(77.4~97.3)	41	40	97.6(87.1~99.9)	42	0	0.0	<0.001
	**Specific Antibody Responses to SARS-CoV-2**													
	28 days after the second dose of immunization	42	42	100.0	42	40	95.2(83.8~99.4)	41	41	100.0	42	0	0.0	<0.001
	28 days after the third dose of immunization	42	42	100.0	42	42	100.0	41	41	100.0	42	0	0.0	<0.001
6-12 years	**Neutralizing Antibodies to Live SARS-CoV-2**													
	28 days after the second dose of immunization	42	40	95.2(83.8~99.4)	42	42	100.0	41	41	100.0	42	0.0	0.0	<0.001
	28 days after the third dose of immunization	42	41	97.6(87.4~99.9)	42	42	100.0	41	41	100.0	42	0.0	0.0	<0.001
	**Specific Antibody Responses to SARS-CoV-2**													
	28 days after the second dose of immunization	42	42	100.0	42	42	100	41	41	100.0	42	0.0	0.0	<0.001
	28 days after the third dose of immunization	42	42	100.0	42	41	97.6(87.4~99.9)	41	41	100.0	42	0.0	0.0	<0.001
3-5 years	**Neutralizing Antibodies to Live SARS-CoV-2**													
	28 days after the second dose of immunization	60	59	98.3(91.1~100,0)	59	58	98.3(180.9~100,0)	57	57	100.0	58	0.0	0.0	<0.001
	28 days after the third dose of immunization	60	60	100.0	59	59	100.0	58	58	100.0	59	0.0	0.0	<0.001
	**Specific Antibody Responses to SARS-CoV-2**													

	28 days after the second dose of immunization	60	60	100.0	59	59	100.0	57	57	100.0	58	0.0	0.0	<0.001
	28 days after the third dose of immunization	60	60	100.0	59	59	100.0	58	58	100.0	59	0.0	0.0	<0.001
3-17 years	**Neutralizing Antibodies to Live SARS-CoV-2**													
	28 days after the second dose of immunization	144	135	93.8(88.5~97.1)	143	137	95.8(91.1~98.4)	139	137	98.6(94.9~99.8)	142	0.0	0.0	<0.001
	28 days after the third dose of immunization	144	137	95.1(180.2~98)	143	139	97.2(93~99.2)	140	139	99.3(96.1~100)	143	0.0	0.0	<0.001
	**Specific Antibody Responses to SARS-CoV-2**													
	28 days after the second dose of immunization	144	144	100.0	143	141	98.6(95~99.8)	139	139	100.0	142	0.0	0.0	<0.001
	28 days after the third dose of immunization	144	144	100.0	143	142	99.3(96.2~100)	140	140	100.0	143	0.0	0.0	<0.001

The low, medium, and high doses represent 2.5μg, 5.0μg, and 10.0 μg/dose, respectively.

Appendix 22. Quadruple growth rate of antibody in subjects aged 3-17 years in phase II clinical trial at different time points (95% CI)（PPS）

Age group	point of time	Low dose			Medium dose			High dose			Placebo			*P*
		N	Antibody 4-fold growth rate	Rate(95%CI)	N	Antibody 4-fold growth rate	Rate(95%CI)	N	Antibody 4-fold growth rate	Rate(95%CI)	N	Antibody 4-fold growth rate	Rate(95%CI)	
13-17 years	**Neutralizing Antibodies to Live SARS-CoV-2**													
	28 days after the second dose of immunization	42	36	85.7(71.5~94.6)	42	37	88.1(74.4~96.0)	41	39	95.1(83.5~99.4)	42	0.0	0.0	<0.001
	28 days after the third dose of immunization	42	36	85.7(71.5~94.6)	42	38	180.5(77.4~97.3)	41	40	97.6(87.1~99.9)	42	0.0	0.0	<0.001
	**Specific Antibody Responses to SARS-CoV-2**													
	28 days after the second dose of immunization	42	42	100.0	42	40	95.2(83.8~99.4)	41	41	100.0	42	0.0	0.0	<0.001
	28 days after the third dose of immunization	42	42	100.0	42	42	100.0	41	41	100.0	42	0.0	0.0	<0.001
6-12 years	**Neutralizing Antibodies to Live SARS-CoV-2**													
	28 days after the second dose of immunization	42	40	95.2(83.8~99.4)	42	42	100.0	41	41	100.0	42	0.0	0.0	<0.001
	28 days after the third dose of immunization	42	41	97.6(87.4~99.9)	42	42	100.0	41	41	100.0	42	0.0	0.0	<0.001
	**Specific Antibody Responses to SARS-CoV-2**													
	28 days after the second dose of immunization	42	42	100.0	42	42	100	41	41	100.0	42	0.0	0.0	<0.001
	28 days after the third dose of immunization	42	42	100.0	42	41	97.6(87.4~99.9)	41	41	100.0	42	0.0	0.0	<0.001
3-5 years	**Neutralizing Antibodies to Live SARS-CoV-2**													
	28 days after the second dose of immunization	60	59	98.3(91.1~100.0)	59	58	98.3(180.9~100.0)	57	57	100.0	57	0.0	0.0	<0.001
	28 days after the third dose of immunization	60	60	100.0	59	59	100.0	55	55	100.0	59	0.0	0.0	<0.001
	**Specific Antibody Responses to SARS-CoV-2**													
	28 days after the second dose of	60	60	100.0	59	59	100.0	57	57	100.0	57	0.0	0.0	<0.001

	immunization													
	28 days after the third dose of immunization	60	60	100.0	59	59	100.0	55	55	100.0	59	0.0	0.0	<0.001
	**Neutralizing Antibodies to Live SARS-CoV-2**													
	28 days after the second dose of immunization	144	135	93.8(88.5~97.1)	143	137	95.8(91.1~98.4)	139	137	98.6(94.9~99.8)	141	0.0	0.0	<0.001
	28 days after the third dose of immunization	144	137	95.1(180.2~98.0)	143	139	97.2(93.0~99.2)	137	136	99.3(96.0~100.0)	143	0.0	0.0	<0.001
3-17 years	**Specific Antibody Responses to SARS-CoV-2**													
	28 days after the second dose of immunization	144	144	100.0	143	141	98.6(95.0~99.8)	139	139	100.0	141	0.0	0.0	<0.001
	28 days after the third dose of immunization	144	144	100.0	143	142	99.3(96.2~100)	137	137	100.0	143	0.0	0.0	<0.001

The low, medium, and high doses represent 2.5μg, 5.0μg, and 10.0 μg/dose, respectively.

Appendix 23. Incidence of adverse events within 30 days after phase I and II combined whole course vaccination for 3-17 years old

		Low dose				Medium dose				High dose				Placebo				*P*
		Case times	Number of cases	Rate(%)	95%CI	Case times	Number of cases	Rate(%)	95%CI	Case times	Number of cases	Rate(%)	95%CI	Case times	Number of cases	Rate(%)	95%CI	
13-17 years (N=60)	Total adverse events	41	22	36.7	24.6~50.1	14	10	16.7	8.3~28.5	21	17	28.3	17.5~41.4	30	23	38.3	26.1~51.8	0.038
	Systemic adverse events	9	8	13.3	5.9~24.6	6	6	10	3.8~20.5	9	8	13.3	5.9~24.6	8	8	13.3	5.9~24.6	0.928
	Local adverse events	12	11	18.3	9.5~30.4	5	4	6.7	1.8~16.2	8	8	13.3	5.9~24.6	16	13	21.7	12.1~34.2	0.111
	Other adverse events	20	12	20	10.8~32.3	3	3	5	1.0~13.9	4	3	5	1.0~13.9	6	6	10	3.8~20.5	0.019
6-12 years (N=60)	Total adverse events	31	19	31.7	20.3~45.03	26	16	26.7	16.1~39.7	9	6	10	3.8~20.5	36	18	30	18.8~43.2	0.022
	Systemic adverse events	7	7	11.7	4.8~22.6	5	5	8.3	2.8~18.4	5	5	8.3	2.8~18.4	8	6	10	3.8~20.5	0.912
	Local adverse events	6	6	10	3.8~20.5	12	8	13.3	5.9~24.6	1	1	1.7	0.0~8.9	13	8	13.3	5.9~24.6	0.098
	Other adverse events	18	11	18.3	9.5~30.4	9	6	10	3.8~20.5	3	2	3.3	0.4~11.5	15	9	15	7.1~26.6	0.059
	Level 3 adverse events	1	1	1.7	0.0~8.9	0	0	0	0.0~6.0	0	0	0	0.0~6.0	1	1	1.7	0.0~8.9	>0.999
3-5 years (N=84)	Total adverse events	62	40	47.6	36.6~58.8	77	43	51.2	40.0~62.3	62	36	42.9	32.1~54.1	69	36	42.9	32.1~54.1	0.645
	Systemic adverse events	33	23	27.4	18.2~38.2	51	31	36.9	26.6~48.1	41	30	35.7	25.6~46.9	42	27	32.1	22.4~43.2	0.555
	Local adverse events	8	7	8.3	3.4~16.4	4	4	4.8	1.3~11.7	3	3	3.6	0.7~10.1	8	8	9.5	4.2~17.9	0.347
	Other adverse events	21	19	22.6	14.2~33.0	22	16	19	11.3~29.1	18	16	19	11.3~29.1	19	11	13.1	6.7~22.2	0.456
	Level 3 adverse events	0	0	0	0.0~4.3	3	2	2.4	0.3~8.3	3	2	2.4	0.3~8.3	0	0	0	0.0~4.3	0.256
3-17 years (N=204)	Total adverse events	134	81	39.7	32.9~46.8	117	69	33.8	27.4~40.8	92	59	28.9	22.8~35.7	135	77	37.7	31.1~44.8	0.107
	Systemic adverse events	49	38	18.6	13.5~24.7	62	42	20.6	15.3~26.8	55	43	21.1	15.7~27.3	58	41	20.1	14.8~26.3	0.935
	Local adverse events	26	24	11.8	7.7~17.0	21	16	7.8	4.5~12.4	12	12	5.9	3.1~10.0	37	29	14.2	9.7~19.8	0.021
	Other adverse events	59	42	20.6	15.3~26.8	34	25	12.3	8.1~17.6	25	21	10.3	6.5~15.3	40	26	12.7	8.5~18.1	0.015
	Level 3 adverse events	1	1	0.5	0.0~2.7	3	2	1	0.1~3.5	3	2	1	0.1~3.5	1	1	0.5	0.0~2.7	0.880

Note: ① total adverse events include adverse events at inoculation site (local), adverse events at non inoculation site (whole body) and other adverse events. The low, medium, and high doses represent 2.5μg, 5.0μg, and 10.0 μg/dose, respectively.

Appendix 24. Incidence of adverse reactions within 30 days after phase I and II combined whole course vaccination for 3-17 years old

Age group	Symptom	Low dose				Medium dose				High dose				Placebo				*P*
		Case times	Number of cases	Rate(%)	95%CI	Case times	Number of cases	Rate(%)	95%CI	Case times	Number of cases	Rate(%)	95%CI	Case times	Number of cases	Rate(%)	95%CI	
13-17 years (N=60)	Total adverse reactions	16	13	21.7	12.1~34.2	11	9	15	7.1~26.6	15	12	20	10.8~32.3	21	16	26.7	16.1~39.7	0.471
	Systemic adverse reactions	4	4	6.7	1.8~16.2	6	6	10	3.8~20.5	7	6	10	3.8~20.5	5	5	8.3	2.8~18.4	0.1802
	Hypersensitivity	0	0	0	0.0~6.0	0	0	0	0.0~6.0	0	0	0	0.0~6.0	0	0	0	0.0~6.0	-
	Fever	4	4	6.7	1.8~16.2	4	4	6.7	1.8~16.2	4	4	6.7	1.8~16.2	4	4	6.7	1.8~16.2	>0.999
	Diarrhea	0	0	0	0.0~6.0	1	1	1.7	0.0~8.9	0	0	0	0.0~6.0	1	1	1.7	0.0~8.9	>0.999
	Arthralgia	0	0	0	0.0~6.0	0	0	0	0.0~6.0	1	1	1.7	0.0~8.9	0	0	0	0.0~6.0	>0.999
	Cough	0	0	0	0.0~6.0	0	0	0	0.0~6.0	0	0	0	0.0~6.0	0	0	0	0.0~6.0	-
	Myalgia	0	0	0	0.0~6.0	1	1	1.7	0.0~8.9	0	0	0	0.0~6.0	0	0	0	0.0~6.0	>0.999
	Vomit	0	0	0	0.0~6.0	0	0	0	0.0~6.0	0	0	0	0.0~6.0	0	0	0	0.0~6.0	-
	Anorexia	0	0	0	0.0~6.0	0	0	0	0.0~6.0	1	1	1.7	0.0~8.9	0	0	0	0.0~6.0	>0.999
	Headache	0	0	0	0.0~6.0	0	0	0	0.0~6.0	1	1	1.7	0.0~8.9	0	0	0	0.0~6.0	>0.999
	Local adverse reaction	12	11	18.3	9.5~30.4	5	4	6.7	1.8~16.2	8	8	13.3	5.9~24.6	16	13	21.7	12.1~34.2	0.111
	Erythema	0	0	0	0.0~6.0	0	0	0	0.0~6.0	0	0	0	0.0~6.0	1	1	1.7	0.0~8.9	>0.999
	Pain	12	11	18.3	9.5~30.4	5	4	6.7	1.8~16.2	8	8	13.3	5.9~24.6	14	12	20	10.8~32.3	0.159
	Swelling	0	0	0	0.0~6.0	0	0	0	0.0~6.0	0	0	0	0.0~6.0	1	1	1.7	0.0~8.9	>0.999
	Other adverse reactions	0	0	0	0.0~6.0	0	0	0	0.0~6.0	0	0	0	0.0~6.0	0	0	0	0.0~6.0	-
	Abdominal pain	0	0	0	0.0~6.0	0	0	0	0.0~6.0	0	0	0	0.0~6.0	0	0	0	0.0~6.0	-
	Laryngeal pain	0	0	0	0.0~6.0	0	0	0	0.0~6.0	0	0	0	0.0~6.0	0	0	0	0.0~6.0	-
	Blistering of oral mucosa	0	0	0	0.0~6.0	0	0	0	0.0~6.0	0	0	0	0.0~6.0	0	0	0	0.0~6.0	-
	runny nose	0	0	0	0.0~6.1	0	0	0	0.0~6.1	0	0	0	0.0~6.1	0	0	0	0.0~6.1	-
	Pharyngeal swelling	0	0	0	0.0~6.2	0	0	0	0.0~6.2	0	0	0	0.0~6.2	0	0	0	0.0~6.2	-
6-12 years (N=60)	Total adverse reactions	10	7	11.7	4.8~22.6	13	9	15	7.1~26.6	3	2	3.3	0.4~11.5	16	10	16.7	8.3~28.5	0.105

	Systemic adverse reactions	1	1	1.7	0.0~8.9	1	1	1.7	0.0~8.9	2	2	3.3	0.4~11.5	3	3	5	1.0~13.9	0.655
	Hypersensitivity	0	0	0	0.0~6.0	0	0	0	0.0~6.0	0	0	0	0.0~6.0	1	1	1.7	0.0~8.9	>0.999
	Fever	0	0	0	0.0~6.0	1	1	1.7	0.0~8.9	1	1	1.7	0.0~8.9	1	1	1.7	0.0~8.9	>0.999
	Diarrhea	0	0	0	0.0~6.0	0	0	0	0.0~6.0	0	0	0	0.0~6.0	0	0	0	0.0~6.0	-
	Arthralgia	0	0	0	0.0~6.0	0	0	0	0.0~6.0	0	0	0	0.0~6.0	0	0	0	0.0~6.0	-
	Cough	1	1	1.7	0.0~8.9	0	0	0	0.0~6.0	1	1	1.7	0.0~8.9	1	1	1.7	0.0~8.9	>0.999
	Myalgia	0	0	0	0.0~6.0	0	0	0	0.0~6.0	0	0	0	0.0~6.0	0	0	0	0.0~6.0	-
	Vomit	0	0	0	0.0~6.0	0	0	0	0.0~6.0	0	0	0	0.0~6.0	0	0	0	0.0~6.0	-
	Anorexia	0	0	0	0.0~6.0	0	0	0	0.0~6.0	0	0	0	0.0~6.0	0	0	0	0.0~6.0	-
	Headache	0	0	0	0.0~6.0	0	0	0	0.0~6.0	0	0	0	0.0~6.0	0	0	0	0.0~6.0	-
	Local adverse reaction	6	6	10	3.8~20.5	12	8	13.3	5.9~24.6	1	1	1.7	0.0~8.9	13	8	13.3	5.9~24.6	0.098
	Erythema	1	1	1.7	0.0~8.9	4	4	6.7	1.8~16.2	0	0	0	0.0~6.0	2	2	3.3	0.4~11.5	0.161
	Pain	5	5	8.3	2.8~18.4	7	7	11.7	4.8~22.6	1	1	1.7	0.0~8.9	9	7	11.7	4.8~22.6	0.155
	Swelling	0	0	0	0.0~6.0	1	1	1.7	0.0~8.9	0	0	0	0.0~6.0	2	1	1.7	0.0~8.9	>0.999
	Other adverse reactions	3	1	1.7	0.0~8.9	0	0	0	0.0~6.0	0	0	0	0.0~6.0	0	0	0	0.0~6.0	>0.999
	Abdominal pain	0	0	0	0.0~6.0	0	0	0	0.0~6.0	0	0	0	0.0~6.0	0	0	0	0.0~6.0	-
	Laryngeal pain	1	1	1.7	0.0~8.9	0	0	0	0.0~6.0	0	0	0	0.0~6.0	0	0	0	0.0~6.0	>0.999
	Blistering of oral mucosa	1	1	1.7	0.0~8.9	0	0	0	0.0~6.0	0	0	0	0.0~6.0	0	0	0	0.0~6.0	>0.999
	runny nose	0	0	0	0.0~6.0	0	0	0	0.0~6.0	0	0	0	0.0~6.0	0	0	0	0.0~6.0	-
	Pharyngeal swelling	1	1	1.7	0.0~8.9	0	0	0	0.0~6.0	0	0	0	0.0~6.0	0	0	0	0.0~6.0	>0.999
3-5 years (N=84)	Total adverse reactions	21	18	21.4	13.2~31.7	15	14	16.7	9.4~26.4	16	13	15.5	8.5~25.0	19	16	19	11.3~29.1	0.757
	Systemic adverse reactions	11	11	13.1	6.7~22.2	11	10	11.9	5.9~20.8	13	10	11.9	5.9~20.8	11	10	11.9	5.9~20.8	0.994
	Hypersensitivity	0	0	0	0.0~4.3	0	0	0	0.0~4.3	0	0	0	0.0~4.3	0	0	0	0.0~4.3	-
	Fever	7	7	8.3	3.4~16.4	3	3	3.6	0.7~10.1	9	9	10.7	5.0~19.4	5	5	6	2.0~13.3	0.309
	Diarrhea	1	1	1.2	0.0~6.5	0	0	0	0.0~4.3	0	0	0	0.0~4.3	1	1	1.2	0.0~6.5	>0.999

	Arthralgia	0	0	0	0.0~4.3	0	0	0	0.0~4.3	0	0	0	0.0~4.3	0	0	0	0.0~4.3	-
	Cough	3	3	3.6	0.7~10.1	8	8	9.5	4.2~17.9	2	2	2.4	0.3~8.3	4	4	4.8	1.3~11.7	0.162
	Myalgia	0	0	0	0.0~4.3	0	0	0	0.0~4.3	0	0	0	0.0~4.3	0	0	0	0.0~4.3	-
	Vomit	0	0	0	0.0~4.3	0	0	0	0.0~4.3	1	1	1.2	0.0~6.5	0	0	0	0.0~4.3	>0.999
	Anorexia	0	0	0	0.0~4.3	0	0	0	0.0~4.3	1	1	1.2	0.0~6.5	0	0	0	0.0~4.3	>0.999
	Headache	0	0	0	0.0~4.3	0	0	0	0.0~4.3	0	0	0	0.0~4.3	1	1	1.2	0.0~6.5	>0.999
	Local adverse reaction	8	7	8.3	3.4~16.4	4	4	4.8	1.3~11.7	3	3	3.6	0.7~10.1	8	8	9.5	4.2~17.9	0.347
	Erythema	1	1	1.2	0.0~6.5	1	1	1.2	0.0~6.5	0	0	0	0.0~4.3	2	2	2.4	0.3~8.3	0.567
	Pain	6	5	6	2.0~13.3	3	3	3.6	0.7~10.1	2	2	2.4	0.3~8.3	6	6	7.1	2.7~14.9	0.453
	Swelling	1	1	1.2	0.0~6.5	0	0	0	0.0~4.3	1	1	1.2	0.0~6.5	0	0	0	0.0~4.3	>0.999
	Other adverse reactions	2	2	2.4	0.3~8.3	0	0	0	0.0~4.3	0	0	0	0.0~4.3	0	0	0	0.0~4.3	0.248
	Abdominal pain	1	1	1.2	0.0~6.5	0	0	0	0.0~4.3	0	0	0	0.0~4.3	0	0	0	0.0~4.3	>0.999
	Laryngeal pain	0	0	0	0.0~4.3	0	0	0	0.0~4.3	0	0	0	0.0~4.3	0	0	0	0.0~4.3	-
	Blistering of oral mucosa	0	0	0	0.0~4.3	0	0	0	0.0~4.3	0	0	0	0.0~4.3	0	0	0	0.0~4.3	-
	runny nose	1	1	1.2	0.0~6.5	0	0	0	0.0~4.3	0	0	0	0.0~4.3	0	0	0	0.0~4.3	>0.999
	Pharyngeal swelling	0	0	0	0.0~4.3	0	0	0	0.0~4.3	0	0	0	0.0~4.3	0	0	0	0.0~4.3	-
3-17 years (N=204)	Total adverse reactions	47	38	18.6	13.5~24.7	39	32	15.7	11.0~21.4	34	27	13.2	8.9~18.7	56	42	20.6	15.3~26.8	0.209
	Systemic adverse reactions	16	16	7.8	4.5~12.4	18	17	8.3	4.9~13.0	22	18	8.8	5.3~13.6	19	18	8.8	5.3~13.6	0.982
	Hypersensitivity	0	0	0	0.0~1.8	0	0	0	0.0~1.8	0	0	0	0.0~1.8	1	1	0.5	0.0~2.7	>0.999
	Fever	11	11	5.4	2.7~9.4	8	8	3.9	1.7~7.6	14	14	6.9	3.8~11.2	10	10	4.9	2.4~8.8	0.606
	Diarrhea	1	1	0.5	0.0~2.7	1	1	0.5	0.0~2.7	0	0	0	0.0~1.8	2	2	1	0.1~3.5	0.570
	Arthralgia	0	0	0	0.0~1.8	0	0	0	0.0~1.8	1	1	0.5	0.0~2.7	0	0	0	0.0~1.8	>0.999
	Cough	4	4	2	0.5~4.9	8	8	3.9	1.7~7.6	3	3	1.5	0.3~4.2	5	5	2.5	0.8~5.6	0.412
	Myalgia	0	0	0	0.0~1.8	1	1	0.5	0.0~2.7	0	0	0	0.0~1.8	0	0	0	0.0~1.8	>0.999
	Vomit	0	0	0	0.0~1.8	0	0	0	0.0~1.8	1	1	0.5	0.0~2.7	0	0	0	0.0~1.8	>0.999

Anorexia	0	0	0	0.0~1.8	0	0	0	0.0~1.8	2	2	1	0.1~3.5	0	0	0	0.0~1.8	0.249
Headache	0	0	0	0.0~1.8	0	0	0	0.0~1.8	1	1	0.5	0.0~2.7	1	1	0.5	0.0~2.7	>0.999
Local adverse reaction	26	24	11.8	7.7~17.0	21	16	7.8	4.5~12.4	12	12	5.9	3.1~10.0	37	29	14.2	9.7~19.8	0.021
Erythema	2	2	1	0.1~3.5	5	5	2.5	0.8~5.6	0	0	0	0.0~1.8	5	5	2.5	0.8~5.6	0.107
Pain	23	21	10.3	6.5~15.3	15	14	6.9	3.8~11.2	11	11	5.4	2.7~9.4	29	25	12.3	8.1~17.6	0.056
Swelling	1	1	0.5	0.0~2.7	1	1	0.5	0.0~2.7	1	1	0.5	0.0~2.7	3	2	1	0.1~3.5	0.896
Other adverse reactions	5	3	1.5	0.3~4.2	0	0	0	0.0~1.8	0	0	0	0.0~1.8	0	0	0	0.0~1.8	0.062
Abdominal pain	1	1	0.5	0.0~2.7	0	0	0	0.0~1.8	0	0	0	0.0~1.8	0	0	0	0.0~1.8	>0.999
Laryngeal pain	1	1	0.5	0.0~2.7	0	0	0	0.0~1.8	0	0	0	0.0~1.8	0	0	0	0.0~1.8	>0.999
Blistering of oral mucosa	1	1	0.5	0.0~2.7	0	0	0	0.0~1.8	0	0	0	0.0~1.8	0	0	0	0.0~1.8	>0.999
runny nose	1	1	0.5	0.0~2.7	0	0	0	0.0~1.8	0	0	0	0.0~1.8	0	0	0	0.0~1.8	>0.999
Pharyngeal swelling	1	1	0.5	0.0~2.7	0	0	0	0.0~1.8	0	0	0	0.0~1.8	0	0	0	0.0~1.8	>0.999

Note: total adverse reactions include inoculation site (local) adverse reactions, non inoculation site (systemic) adverse reactions and other adverse reactions. The low, medium, and high doses represent 2.5μg, 5.0μg, and 10.0 μg/dose, respectively.

Appendix 25. Antibody GMT of phase I and II subjects aged 3-17 years before the first dose of vaccination to 28 days after the third dose of vaccination （FAS）

Age group	point of time	Low dose		Medium dose		High dose		Placebo		*P*
		N	GMT(95%CI)	N	GMT(95%CI)	N	GMT(95%CI)	N	GMT(95%CI)	
13-17 years	**Neutralizing Antibodies to Live SARS-CoV-2**									
	Before 1st dose immunization	60	5(5~5)	60	5(5~5)	60	5(5~5)	60	5(5~5)	
	28 days after the second dose of immunization	60	71.8(56.2~91.6)	60	78.7(61.6~100.5)	59	112.7(88.1~144.1)	60	5(3.9~6.4)	<0.001
	28 days after the third dose of immunization	60	147.7(110.7~197.2)	60	136.5(102.2~182.2)	59	216.3(161.6~289.5)	60	5(3.7~6.7)	<0.001
	**Specific Antibody Responses to SARS-CoV-2**									
	Before 1st dose immunization	60	10(10~10)	60	10(10~10)	60	10(10~10)	60	10(10~10)	
	28 days after the second dose of immunization	60	128.5(110.6~149.2)	60	133(114.5~154.4)	59	160(137.6~186)	60	10(8.6~11.6)	<0.001
	28 days after the third dose of immunization	60	298.6(257.3~346.5)	60	259.9(224~301.6)	59	298.2(256.7~346.5)	60	10(8.6~11.6)	<0.001
6-12 years	**Neutralizing Antibodies to Live SARS-CoV-2**									
	Before 1st dose immunization	60	5(5~5)	60	5(5~5)	60	5(5~5)	60	5(5~5)	
	28 days after the second dose of immunization	60	108.7(88.7~133.2)	60	223.6(182.4~274)	59	248.6(202.5~305.3)	60	5(4.1~6.1)	<0.001
	28 days after the third dose of immunization	60	325.2(264.2~400.2)	60	477.3(387.7~587.5)	59	570.3(462.5~703.3)	60	5(4.1~6.2)	<0.001
	**Specific Antibody Responses to SARS-CoV-2**									
	Before 1st dose immunization	60	10(10~10)	60	10(10~10)	60	10(10~10)	60	10(10~10)	
	28 days after the second dose of immunization	60	211.1(183.4~243.1)	60	226.3(196.5~260.5)	59	301.7(261.8~347.8)	60	10(8.7~11.5)	<0.001
	28 days after the third dose of immunization	60	463.1(389.9~550.2)	60	485(408.3~576.2)	59	512(430.3~609.1)	60	10(8.4~11.9)	<0.001
3-5 years	**Neutralizing Antibodies to Live SARS-CoV-2**									
	Before 1st dose immunization	84	5(5~5)	84	5(5~5)	84	5(5~5)	84	5(5~5)	
	28 days after the second dose of immunization	84	237.8(200.3~282.4)	83	268.4(225.8~318.9)	81	380.3(319.3~452.9)	80	5(4.2~6)	<0.001
	28 days after the third dose of immunization	84	794.3(671.7~939.4)	83	1801(761.1~1066.6)	82	808.1(681.9~957.6)	81	5(4.2~5.9)	<0.001
	**Specific Antibody Responses to SARS-CoV-2**									

	Before 1st dose immunization	84	10(10~10)	84	10(10~10)	84	10(10~10)	84	10(10~10)	
	28 days after the second dose of immunization	84	282.7(248.7~321.4)	83	289.5(254.5~329.3)	81	325.5(285.7~370.9)	80	10(8.8~11.4)	<0.001
	28 days after the third dose of immunization	84	754.8(630.9~1803.2)	83	731.5(610.7~876.2)	82	960.3(800.8~1151.5)	81	10(8.3~12)	<0.001
3-17 years	**Neutralizing Antibodies to Live SARS-CoV-2**									
	Before 1st dose immunization	204	5(5~5)	204	5(5~5)	204	5(5~5)	204	5(5~5)	
	28 days after the second dose of immunization	204	132.8(116.4~151.5)	203	176.9(155.1~201.9)	199	233.8(204.6~267.1)	200	5(4.4~5.7)	<0.001
	28 days after the third dose of immunization	204	372.4(320.9~432.3)	203	427.5(368.1~496.4)	200	494.3(425.2~574.6)	201	5(4.3~5.8)	<0.001
	**Specific Antibody Responses to SARS-CoV-2**									
	Before 1st dose immunization	204	10(10~10)	204	10(10~10)	204	10(10~10)	204	10(10~10)	
	28 days after the second dose of immunization	204	205.7(188.3~224.7)	203	213.9(195.8~233.7)	199	257.8(235.8~282)	200	10(9.1~10.9)	<0.001
	28 days after the third dose of immunization	204	497.7(445.1~556.6)	203	477.1(426.6~533.7)	200	564.9(504.6~632.4)	201	10(8.9~11.2)	<0.001

The low, medium, and high doses represent 2.5μg, 5.0μg, and 10.0 μg/dose, respectively.

Appendix 26. Antibody GMT of phase I and II subjects aged 3-17 years from before the first dose of vaccination to 28 days after the third dose of vaccination（PPS）

Age group	point of time	Low dose		Medium dose		High dose		Placebo		*P*
		N	GMT(95%CI)	N	GMT(95%CI)	N	GMT(95%CI)	N	GMT(95%CI)	
13-17 years	**Neutralizing Antibodies to Live SARS-CoV-2**									
	Before 1st dose immunization	60	5(5~5)	60	5(5~5)	60	5(5~5)	60	5(5~5)	
	28 days after the second dose of immunization	60	71.8(56.2~91.6)	60	78.7(61.6~100.5)	59	112.7(88.1~144.1)	60	5(3.9~6.4)	<0.001
	28 days after the third dose of immunization	60	147.7(110.7~197.2)	60	136.5(102.2~182.2)	59	216.3(161.6~289.5)	60	5(3.7~6.7)	<0.001
	**Specific Antibody Responses to SARS-CoV-2**									
	Before 1st dose immunization	60	10(10~10)	60	10(10~10)	60	10(10~10)	60	10(10~10)	
	28 days after the second dose of immunization	60	128.5(110.6~149.2)	60	133(114.5~154.4)	59	160(137.6~186)	60	10(8.6~11.6)	<0.001
	28 days after the third dose of immunization	60	298.6(257.3~346.5)	60	259.9(224~301.6)	59	298.2(256.7~346.5)	60	10(8.6~11.6)	<0.001
6-12 years	**Neutralizing Antibodies to Live SARS-CoV-2**									
	Before 1st dose immunization	60	5(5~5)	60	5(5~5)	60	5(5~5)	60	5(5~5)	
	28 days after the second dose of immunization	60	108.7(88.7~133.2)	60	223.6(182.4~274)	59	248.6(202.5~305.3)	60	5(4.1~6.1)	<0.001
	28 days after the third dose of immunization	60	325.2(264.8~399.3)	59	494.1(401.7~607.8)	59	570.3(463.7~701.6)	60	5(4.1~6.1)	<0.001
	**Specific Antibody Responses to SARS-CoV-2**									
	Before 1st dose immunization	60	10(10~10)	60	10(10~10)	60	10(10~10)	60	10(10~10)	
	28 days after the second dose of immunization	60	211.1(183.4~243.1)	60	226.3(196.5~260.5)	59	301.7(261.8~347.8)	60	10(8.7~11.5)	<0.001
	28 days after the third dose of immunization	60	463.1(389.8~550.3)	59	488.5(410.5~581.2)	59	512(430.2~609.2)	60	10(8.4~11.9)	<0.001
3-5 years	**Neutralizing Antibodies to Live SARS-CoV-2**									
	Before 1st dose immunization	84	5(5~5)	84	5(5~5)	84	5(5~5)	84	5(5~5)	
	28 days after the second dose of immunization	84	237.8(200.3~282.5)	83	268.4(225.7~319)	80	383.4(321.5~457.3)	79	5(4.2~6)	<0.001
	28 days after the third dose of immunization	84	794.3(672.4~938.4)	83	1801(761.9~1065.5)	78	825.1(694~980.9)	81	5(4.2~5.9)	<0.001
	**Specific Antibody Responses to SARS-CoV-2**									

	Before 1st dose immunization	84	10(10~10)	84	10(10~10)	84	10(10~10)	84	10(10~10)	
	28 days after the second dose of immunization	84	282.7(248.9~321.2)	83	289.5(254.6~329.1)	80	331.3(2180.7~377.5)	79	10(8.8~11.4)	<0.001
	28 days after the third dose of immunization	84	754.8(632.2~1801.3)	83	731.5(612~874.4)	78	1015.9(845.2~1221.2)	81	10(8.3~12)	<0.001
3-17 years	**Neutralizing Antibodies to Live SARS-CoV-2**									
	Before 1st dose immunization	204	5(5~5)	204	5(5~5)	204	5(5~5)	204	5(5~5)	
	28 days after the second dose of immunization	204	132.8(116.4~151.5)	203	176.9(155~201.9)	198	234(204.7~267.5)	199	5(4.4~5.7)	<0.001
	28 days after the third dose of immunization	204	372.4(320.9~432.3)	202	431.6(371.6~501.3)	196	493.4(423.8~574.4)	201	5(4.3~5.8)	<0.001
	**Specific Antibody Responses to SARS-CoV-2**									
	Before 1st dose immunization	204	10(10~10)	204	10(10~10)	204	10(10~10)	204	10(10~10)	
	28 days after the second dose of immunization	204	205.7(188.4~224.7)	203	213.9(195.8~233.7)	198	259.4(237.2~283.7)	199	10(9.1~10.9)	<0.001
	28 days after the third dose of immunization	204	497.7(445.1~556.5)	202	478.1(427.4~534.8)	196	571.5(510~640.5)	201	10(8.9~11.2)	<0.001

The low, medium, and high doses represent 2.5μg, 5.0μg, and 10.0 μg/dose, respectively.

Appendix 27. Comparison of antibody levels of phase I and phase II subjects aged 3-17 years after the full vaccination（FAS）

Age group	Antibody type	Low dose (N = 60)	Medium dose (N = 60)	High dose (N = 59)	Placebo (N =60)
13-17 years	**Neutralizing Antibodies to Live SARS-CoV-2**				
	GMT (95% CI)	147.7(110.7~197.2)	136.5(102.2~182.2)	216.3(161.6~289.5)	5(3.7~6.7)
	GMI (95% CI)	1	0.92(0.61~1.39)	1.46(0.97~2.21)	-
	Antibody 4-fold growth rate— % (95% CI)	88.3(77.4~95.2)	91.7(81.6~97.2)	96.6(88.3~99.6)	0.0
	**Specific Antibody Responses to SARS-CoV-2**				
	GMT (95% CI)	298.6(257.3~346.5)	259.9(224~301.6)	298.2(256.7~346.5)	10(8.6~11.6)
	GMI (95% CI)	1	0.87(0.71~1.07)	1(0.81~1.23)	-
	Antibody 4-fold growth rate — % (95% CI)	100.0	100.0	100.0	0.0
6-12 years	Antibody type	Low dose (N =60)	Medium dose (N =60)	High dose (N =59)	Placebo (N =60)
	**Neutralizing Antibodies to Live SARS-CoV-2**				
	GMT (95% CI)	325.2(264.2~400.2)	477.3(387.7~587.5)	570.3(462.5~703.3)	5(4.1~6.1)
	GMI (95% CI)	1	1.47(1.09~1.97)	1.75(1.31~2.36)	-
	Antibody 4-fold growth rate— % (95% CI)	96.7(88.5~99.6)	100.0	100.0	0.0
	Specific Antibody Responses to SARS-CoV-2				
	GMT (95% CI)	463.1(389.9~550.2)	485(408.3~576.2)	512(430.3~609.1)	10(8.4~11.9)
	GMI (95% CI)	1	1.05(0.82~1.34)	1.11(0.87~1.41)	-
	Antibody 4-fold growth rate — % (95% CI)	100.0	98.3(91.1~100.0)	100.0	0.0
3-5 years	Antibody type	Low dose (N =84)	Medium dose (N = 83)	High dose (N = 82)	Placebo (N =81)
	**Neutralizing Antibodies to Live SARS-CoV-2**				

	Antibody type	Low dose	Medium dose	High dose	Placebo
	GMT (95% CI)	794.3(671.7~939.4)	1801(761.1~1066.6)	808.1(681.9~957.6)	5(4.2~5.9)
	GMI (95% CI)	1	1.13(0.89~1.44)	1.02(0.8~1.29)	-
	Antibody 4-fold growth rate— % (95% CI)	100.0	100.0	100.0	0.0
	**Specific Antibody Responses to SARS-CoV-2**				
	GMT (95% CI)	754.8(630.9~1803.2)	731.5(610.7~876.2)	960.3(800.8~1151.5)	10(8.3~12)
	GMI (95% CI)	1	0.97(0.75~1.25)	1.27(0.99~1.64)	-
	Antibody 4-fold growth rate — % (95% CI)	100.0	100.0	100.0	0.0
3-17 years	Antibody type	Low dose (N =204)	Medium dose (N =203)	High dose (N =200)	Placebo (N =201)
	**Neutralizing Antibodies to Live SARS-CoV-2**				
	GMT (95% CI)	372.4(320.9~432.3)	427.5(368.1~496.4)	494.3(425.2~574.6)	5(4.3~5.8)
	GMI (95% CI)	1	1.15(0.93~1.42)	1.33(1.07~1.64)	-
	Antibody 4-fold growth rate— % (95% CI)	95.6(91.8~98.0)	97.5(94.3~99.2)	99.096.4~99.9)	0.0
	**Specific Antibody Responses to SARS-CoV-2**				
	GMT (95% CI)	497.7(445.1~556.6)	477.1(426.6~533.7)	564.9(504.6~632.4)	10(8.9~11.2)
	GMI (95% CI)	1	0.96(0.82~1.12)	1.14(0.97~1.33)	-
	Antibody 4-fold growth rate — % (95% CI)	100.0	99.5(97.3~100.0)	100.0	0.0

The low, medium, and high doses represent 2.5μg, 5.0μg, and 10.0 μg/dose, respectively.

Appendix 28. Comparison of antibody levels of phase I and phase II subjects aged 3-17 years after the full vaccination（PPS）

Age group	Antibody type	Low dose (N =60)	Medium dose (N = 60)	High dose (N = 59)	Placebo (N =60)
13-17 years	**Neutralizing Antibodies to Live SARS-CoV-2**				
	GMT (95% CI)	147.7(110.7~197.2)	136.5(102.2~182.2)	216.3(161.6~289.5)	5(3.7~6.7)
	GMI (95% CI)	1	0.92(0.61~1.39)	1.46(0.97~2.21)	-
	Antibody 4-fold growth rate— % (95% CI)	88.3(77.4~95.2)	91.7(81.6~97.2)	96.6(88.3~99.6)	0.0
	**Specific Antibody Responses to SARS-CoV-2**				
	GMT (95% CI)	298.6(257.3~346.5)	259.9(224~301.6)	298.2(256.7~346.5)	10(8.6~11.6)
	GMI (95% CI)	1	0.87(0.71~1.07)	1(0.81~1.23)	0.0
	Antibody 4-fold growth rate — % (95% CI)	100.0	100.0	100.0	0.0
		Low dose (N =60)	Medium dose (N =59)	High dose (N = 59)	Placebo (N =60)
6-12 years	**Neutralizing Antibodies to Live SARS-CoV-2**				
	GMT (95% CI)	325.2(264.8~399.3)	494.1(401.7~607.8)	570.3(463.7~701.6)	5(4.1~6.1)
	GMI (95% CI)	1	1.52(1.14~2.03)	1.75(1.31~2.35)	-
	Antibody 4-fold growth rate— % (95% CI)	96.7(88.5~99.6)	100.0	100.0	0.0
	**Specific Antibody Responses to SARS-CoV-2**				
	GMT (95% CI)	463.1(389.8~550.3)	488.5(410.5~581.2)	512(430.2~609.2)	10(8.4~11.9)
	GMI (95% CI)	1	1.05(0.83~1.35)	1.11(0.87~1.41)	-

	Antibody 4-fold growth rate — % (95% CI)	100.0	98.3(180.9~100)	100.0	0.0
		Low dose (N =84)	Medium dose (N =83)	High dose (N =78)	Placebo (N =81)
3-5 years	**Neutralizing Antibodies to Live SARS-CoV-2**				
	GMT (95% CI)	794.3(672.4~938.4)	1801(761.9~1065.5)	825.1(694~980.9)	5(4.2~5.9)
	GMI (95% CI)	1	1.13(0.9~1.44)	1.04(0.82~1.32)	-
	Antibody 4-fold growth rate— % (95% CI)	100.0	100.0	100.0	0.0
	**Specific Antibody Responses to SARS-CoV-2**				
	GMT (95% CI)	754.8(632.2~1801.3)	731.5(612~874.4)	1015.9(845.2~1221.2)	10(8.3~12)
	GMI (95% CI)	1	1.35(1.04~1.74)	0.97(0.75~1.25)	-
	Antibody 4-fold growth rate — % (95% CI)	100.0	100.0	100.0	0.0
		Low dose (N =204)	Medium dose (N =202)	High dose (N =196)	Placebo (N =201)
3-17 years	**Neutralizing Antibodies to Live SARS-CoV-2**				
	GMT (95% CI)	372.4(320.9~432.3)	431.6(371.6~501.3)	493.4(423.8~574.4)	5(4.3~5.8)
	GMI (95% CI)	1	1.16(0.94~1.43)	1.32(1.07~1.64)	-
	Antibody 4-fold growth rate— % (95% CI)	95.6(91.8~98.0)	97.5(94.3~99.2)	99.0(96.4~99.9)	0.0
	**Specific Antibody Responses to SARS-CoV-2**				

GMT (95% CI)	497.7(445.1~556.5)	478.1(427.4~534.8)	571.5(510~640.5)	10(8.9~11.2)
GMI (95% CI)	1	0.96(0.82~1.13)	1.15(0.98~1.35)	-
Antibody 4-fold growth rate — % (95% CI)	100.0	99.5(97.3~100.0)	100.0	0.0

The low, medium, and high doses represent 2.5μg, 5.0μg, and 10.0 μg/dose, respectively.

Appendix 29. Quadruple growth rate of antibody in phase I and phase II clinical trials combined with subjects aged 3-17 years from before the first dose of vaccination to 28 days after the third dose of vaccination（FAS）

Age group	point of time	Low dose			Medium dose			High dose			Placebo			*P*
		N	Antibody 4-fold growth rate	Rate(95%CI)	N	Antibody 4-fold growth rate	Rate(95%CI)	N	Antibody 4-fold growth rate	Rate(95%CI)	N	Antibody 4-fold growth rate	Rate(95%CI)	
13-17 years	**Neutralizing Antibodies to Live SARS-CoV-2**													
	28 days after the second dose of immunization	60	53	88.3(77.4~95.2)	60	54	180.0(79.5~96.2)	59	56	94.9(85.9~98.9)	60	0	0.0	<0.001
	28 days after the third dose of immunization	60	53	88.3(77.4~95.2)	59	55	91.7(81.6~97.2)	59	57	96.6(88.3~99.6)	60	0	0.0	<0.001
	**Specific Antibody Responses to SARS-CoV-2**													
	28 days after the second dose of immunization	60	60	100.0	60	57	95.0(86.1~99)	59	59	100.0	60	0	0.0	<0.001
	28 days after the third dose of immunization	60	60	100.0	59	58	98.3(180.9~100)	59	59	100.0	60	0	0.0	<0.001
6-12 years	**Neutralizing Antibodies to Live SARS-CoV-2**													
	28 days after the second dose of immunization	60	57	95.0(86.1~99.0)	60	60	100.0	59	59	100.0	60	0	0.0	<0.001
	28 days after the third dose of immunization	60	58	96.7(88.5~99.6)	59	59	100.0	59	59	100.0	60	0	0.0	<0.001
	**Specific Antibody Responses to SARS-CoV-2**													
	28 days after the second dose of immunization	60	60	100.0	60	60	100.0	59	59	100.0	60	0	0.0	<0.001
	28 days after the third dose of immunization	60	60	100.0	59	58	98.3(180.9~100)	59	59	100.0	60	0	0.0	<0.001
3-5 years	**Neutralizing Antibodies to Live SARS-CoV-2**													
	28 days after the second dose of immunization	84	82	97.6(91.7~99.7)	83	81	97.6(91.6~99.7)	80	80	100.0	79	0	0.0	<0.001
	28 days after the third dose of immunization	84	84	100	83	83	100.0	78	78	100.0	81	0	0.0	<0.001
	**Specific Antibody Responses to SARS-CoV-2**													

3-17 years	28 days after the second dose of immunization	84	84	100.0	83	83	100.0	80	80	100.0	79	0	0.0	<0.001
	28 days after the third dose of immunization	84	84	100.0	83	83	100.0	78	78	100.0	81	0	0.0	<0.001
	**Neutralizing Antibodies to Live SARS-CoV-2**													
	28 days after the second dose of immunization	204	192	94.1(180.0~96.9)	203	195	96.1(92.4~98.3)	198	195	98.5(95.6~99.7)	199	0	0.0	<0.001
	28 days after the third dose of immunization	204	195	95.6(91.8~98.0)	202	197	97.5(94.3~99.2)	196	194	99.0(96.4~99.9)	201	0	0.0	<0.001
	**Specific Antibody Responses to SARS-CoV-2**													
	28 days after the second dose of immunization	204	204	100.0	203	200	98.5(95.7~99.7)	198	198	100.0	199	0	0.0	<0.001
	28 days after the third dose of immunization	204	204	100.0	202	201	99.5(97.3~100.0)	196	196	100.0	201	0	0.0	<0.001

The low, medium, and high doses represent 2.5μg, 5.0μg, and 10.0 μg/dose, respectively.

Appendix 30. Quadruple growth rate of antibody in phase I and phase II clinical trials combined with subjects aged 3-17 years from before the first dose of vaccination to 28 days after the third dose of vaccination（PPS）

Age group	point of time	Low dose			Medium dose			High dose			Placebo			*P*
		N	Antibody 4-fold growth rate	Rate(95%CI)	N	Antibody 4-fold growth rate	Rate(95%CI)	N	Antibody 4-fold growth rate	Rate(95%CI)	N	Antibody 4-fold growth rate	Rate(95%CI)	
13-17 years	**Neutralizing Antibodies to Live SARS-CoV-2**													
	28 days after the second dose of immunization	60	53	88.3(77.4~95.2)	60	54	180.0(79.5~96.2)	59	56	94.9(85.9~98.9)	60	0	0.0	<0.001
	28 days after the third dose of immunization	60	53	88.3(77.4~95.2)	60	55	91.7(81.6~97.2)	59	57	96.6(88.3~99.6)	60	0	0.0	<0.001
	**Specific Antibody Responses to SARS-CoV-2**													
	28 days after the second dose of immunization	60	60	100.0	60	57	95.0(86.1~99.0)	59	59	100.0	60	0	0.0	0
	28 days after the third dose of immunization	60	60	100.0	60	60	100	59	59	100.0	60	0	0.0	0
6-12 years	**Neutralizing Antibodies to Live SARS-CoV-2**													
	28 days after the second dose of immunization	60	57	95.0(86.1~99.0)	60	60	100.0	59	59	100.0	60	0	0.0	<0.001
	28 days after the third dose of immunization	60	58	96.7(88.5~99.6)	59	59	100.0	59	59	100.0	60	0	0.0	<0.001
	**Specific Antibody Responses to SARS-CoV-2**													
	28 days after the second dose of immunization	60	60	100.0	60	60	100.0	59	59	100.0	60	0	0.0	<0.001
	28 days after the third dose of immunization	60	60	100.0	59	58	98.3(180.9~100)	59	59	100.0	60	0	0.0	<0.001
3-5 years	**Neutralizing Antibodies to Live SARS-CoV-2**													
	28 days after the second dose of immunization	84	82	97.6(91.7~99.7)	83	81	97.6(91.6~99.7)	80	80	100.0	79	0	0.0	<0.001
	28 days after the third dose of immunization	84	84	100.0	83	83	100.0	78	78	100.0	81	0	0.0	<0.001

	**Specific Antibody Responses to SARS-CoV-2**													
	28 days after the second dose of immunization	84	84	100.0	83	83	100.0	80	80	100.0	79	0	0.0	<0.001
	28 days after the third dose of immunization	84	84	100.0	83	83	100.0	78	78	100.0	81	0	0.0	<0.001
3-17 years	**Neutralizing Antibodies to Live SARS-CoV-2**													
	28 days after the second dose of immunization	204	192	94.1(180.0~96.9)	203	195	96.1(92.4~98.3)	198	195	98.5(95.6~99.7)	199	0	0.0	<0.001
	28 days after the third dose of immunization	204	195	95.6(91.8~98.0)	202	197	97.5(94.3~99.2)	196	194	99.0(96.4~99.9)	201	0	0.0	<0.001
	**Specific Antibody Responses to SARS-CoV-2**													
	28 days after the second dose of immunization	204	204	100.0	203	200	98.5(95.7~99.7)	198	198	100.0	199	0	0.0	<0.001
	28 days after the third dose of immunization	204	204	100.0	202	201	99.5(97.3~100.0)	196	196	100.0	201	0	0.0	<0.001

The low, medium, and high doses represent 2.5μg, 5.0μg, and 10.0 μg/dose, respectively.

Appendix 31. The change trend of GMT of neutralizing antibody in phase I clinical trial at 90 days after the full vaccination（PPS）

Age group	Index	Placebo	Low dose	Medium dose	High dose
13-17 years	N	18	18	18	18
	Min，MAX	5,5	5,447	5,513	5,358
	$M(P_{25},P_{75})$	5.0(5.0,5.0)	103.0(42.0,233.0)	115.5(76.0,166.0)	151.5(81.0,179.0)
	GMT（95%CI）	5.0(2.8~8.8)	69.6(39.4~122.8)	93.5(53.0~165.0)	88.8(50.3~156.7)
	GMT rate（95%CI）	-	1	1.34(0.60~3.00)	1.28(0.57~2.85)
6-12 years	N	18	17	18	18
	Min, Max	5,5	5,470	42,1336	5,782
	$M(P_{25},P_{75})$	5.0(5.0,5.0)	143.0(75.0,421.0)	265.5(168.0,429.0)	277.0(84.0,341.0)
	GMT（95%CI）	5.0(3.0~8.3)	121.2(72.1~203.8)	263.3(158.9~436.3)	156.4(94.4~259.2)
	GMTrate（95%CI）	-	1	2.17(1.05~4.49)	1.29(0.63~2.66)
3-5 years	N	22	24	24	23
	Min, Max	5,5	55,1136	42,1854	5,892
	$M(P_{25},P_{75})$	5.0(5.0,5.0)	235.5(115.5,467.0)	258.0(150.0,665.0)	223.0(129.0,319.0)
	GMT（95%CI）	5.0(3.5~7.1)	247.5(176.6~346.7)	294.1(209.9~412.1)	195.3(138.4~275.6)

Appendix 32. The change trend of GMT of neutralizing antibody in phase I clinical trial at 90 days after the full vaccination (FAS)

Age group	Index	Placebo	Low dose	Medium dose	High dose
13-17 years	N	18	18	18	18
	Min，MAX	5,5	5,447	5,513	5,358
	$M(P_{25},P_{75})$	5.0(5.0,5.0)	103.0(42.0,233.0)	115.5(76.0,166.0)	151.5(81.0,179.0)
	GMT（95%CI）	5.0(2.8~8.8)	69.6(39.4~122.8)	93.5(53.0~165.0)	88.8(50.3~156.7)
	GMTrate （95%CI）	-	1	1.34(0.60~3.00)	1.28(0.57~2.85)
6-12 years	N	18	18	18	18
	Min, Max	5,5	5,470	42,1336	5,782
	$M(P_{25},P_{75})$	5.0(5.0,5.0)	161.0(75.0,421.0)	265.5(168.0,429.0)	277.0(84.0,341.0)
	GMT（95%CI）	5.0(3.0~8.3)	128.9(77.8~213.6)	263.3(158.9~436.3)	156.4(94.4~259.2)
	GMTrate （95%CI）	-	1	2.04(1.00~4.17)	1.21(0.59~2.48)
3-5 years	N	22	24	24	24
	Min, Max	5,5	55,1136	42,1854	5,892
	$M(P_{25},P_{75})$	5.0(5.0,5.0)	235.5(115.5,467.0)	258.0(150.0,665.0)	216.5(143.5,317.0)
	GMT（95%CI）	5.0(3.5~7.1)	247.5(176.9~346.1)	294.1(210.3~411.4)	194.8(139.3~272.5)
	GMTrate （95%CI）	-	1	1.19(0.74~1.91)	0.79(0.49~1.27)

Appendix 33. Quadruple growth rate of neutralizing antibody in phase I clinical trial at 90 days after the third dose of vaccination（%）(PPS)

Age group	Index	Placebo	Low dose	Medium dose	High dose
13-17 years	N	18	18	18	18
	4-fold growth rate	0	14	16	15
	rate （95%）	0(0~18.5)	77.8(52.4~93.6)	88.9(65.3~98.6)	83.3(58.6~96.4)
6-12 years	N	18	17	18	18
	4-fold growth rate	0	15	18	16
	rate （95%）	0(0~18.5)	88.2(63.6~98.5)	100	88.9(65.3~98.6)
13-17 years	N	22	24	24	23
	4-fold growth rate	0	24	24	22
	rate （95%）	0(0~15.4)	100	100	95.7(78.1~99.9)

Appendix 34. Quadruple growth rate of neutralizing antibody in phase I clinical trial at 90 days after the third dose of vaccination（%）(FAS)

Age group	Index	Placebo	Low dose	Medium dose	High dose
13-17years	N	18	18	18	18
	4-fold growth rate	0	14	16	15
	rate （95%）	0(0~18.5)	77.8(52.4~93.6)	88.9(65.3~98.6)	83.3(58.6~96.4)
6-12years	N	18	18	18	18
	4-fold growth rate	0	16	18	16
	rate （95%）	0(0~18.5)	88.9(65.3~98.6)	100	88.9(65.3~98.6)
3-5years	N	22	24	24	24
	4-fold growth rate	0	24	24	23
	rate （95%）	0(0~15.4)	100	100	95.8(78.9~99.9)

Appendix 35. The change trend of GMT of specific antibody in phase I clinical trial at 90 days after the full vaccination (PPS)

Age group	Index	Placebo	Low dose	Medium dose	High dose
13-17years	N	18	18	18	18
	Min，MAX	10,10	80,640	80,1280	40,320
	$M(P_{25},P_{75})$	10.0(10.0,10.0)	160.0(160.0,320.0)	160.0(160.0,320.0)	160.0(160.0,320.0)
	GMT（95%CI）	10.0(7.8~12.8)	179.6(139.8~230.7)	194.0(151.0~249.2)	160.0(124.6~205.5)
	GMTrate （95%CI）	-	1	1.08(0.76~1.54)	0.89(0.63~1.27)
6-12years	N	18	17	18	18
	Min, Max	10,10	80,640	160,1280	160,1280
	$M(P_{25},P_{75})$	10.0(10.0,10.0)	160.0(160.0,640.0)	320.0(160.0,640.0)	320.0(160.0,640.0)
	GMT（95%CI）	10.0(7.6~13.2)	250.6(187.7~334.5)	345.6(261.0~457.6)	320.0(241.7~423.7)
	GMTrate （95%CI）	-	1	1.41(0.95~2.10)	1.31(0.88~1.94)
3-5years	N	22	24	24	23
	Min, Max	10,10	160,1280	160,2560	160,2560
	$M(P_{25},P_{75})$	10.0(10.0,10.0)	320.0(240.0,640.0)	640.0(320.0,1280.0)	640.0(640.0,1280.0)
	GMT（95%CI）	10.0(7.7~13.0)	369.7(287.0~476.3)	604.1(468.9~778.2)	766.8(592.0~993.3)
	GMTrate （95%CI）	-	1	1.63(1.14~2.34)	2.00(1.40~2.87)

Appendix 36. The change trend of GMT of specific antibody in phase Iclinical trial at 90 days after the full vaccination (FAS)

Age group	Index	Placebo	Low dose	Medium dose	High dose
13-17years	N	18	18	18	18
	Min，MAX	10,10	80,640	80,1280	40,320
	$M(P_{25},P_{75})$	10.0(10.0,10.0)	160.0(160.0,320.0)	160.0(160.0,320.0)	160.0(160.0,320.0)
	GMT（95%CI）	10.0(7.8~12.8)	179.6(139.8~230.7)	194.0(151.0~249.2)	160.0(124.6~205.5)
	GMTrate （95%CI）	-	1	1.08(0.76~1.54)	0.89(0.63~1.27)
6-12 years	N	18	18	18	18
	Min, Max	10,10	80,640	160,1280	160,1280
	$M(P_{25},P_{75})$	10.0(10.0,10.0)	160.0(160.0,640.0)	320.0(160.0,640.0)	320.0(160.0,640.0)
	GMT（95%CI）	10.0(7.6~13.2)	244.4(184.8~323.3)	345.6(261.3~457.2)	320.0(241.9~423.3)
	GMTrate （95%CI）	-	1	1.41(0.95~2.10)	1.31(0.88~1.94)
3-5 years	N	22	24	24	24
	Min, Max	10,10	160,1280	160,2560	160,2560
	$M(P_{25},P_{75})$	10.0(10.0,10.0)	320.0(240.0,640.0)	640.0(320.0,1280.0)	640.0(480.0,1280.0)
	GMT（95%CI）	10.0(7.7~13.0)	369.7(286.7~476.8)	604.1(468.4~779.1)	739.4(573.3~953.7)
	GMTrate （95%CI）	-	1	1.63(1.14~2.34)	2.00(1.40~2.87)

Appendix 37. Quadruple growth rate of specific antibody in phase I clinical trial at 90 days after the third dose of vaccination（%）(PPS)

Age group	Index	Placebo	Low dose	Medium dose	High dose
13-17years	N	18	18	18	18
	4-fold growth rate	0	18	18	18
	rate （95%）	0(0~18.5)	100	100	100
6-12 years	N	18	17	18	18
	4-fold growth rate	0	17	18	18
	rate （95%）	0(0~18.5)	100	100	100
3-5 years	N	22	24	24	23
	4-fold growth rate	0	24	24	23
	rate （95%）	0(0~15.4)	100	100	100

Appendix 38. Quadruple growth rate of specific antibody in phase I clinical trial at 90 days after the third dose of vaccination（%）(FAS)

Age group	Index	Placebo	Low dose	Medium dose	High dose
13-17 years	N	18	18	18	18
	4-fold growth rate	0	18	18	18
	rate （95%）	0(0~18.5)	100	100	100
6-12 years	N	18	18	18	18
	4-fold growth rate	0	18	18	18
	rate （95%）	0(0~18.5)	100	100	100
3-5 years	N	22	24	24	24
	4-fold growth rate	0	24	24	24
	rate （95%）	0(0~15.4)	100	100	100

Appendix 39. The change trend of GMT of neutralizing antibody in phase II clinical trial at 90 days after the full vaccination（PPS）

Age group	Index	Placebo	Low dose	Medium dose	High dose
13-17years	N	42	42	42	41
	Min，MAX	5,5	5,854	5,444	5,774
	$M(P_{25},P_{75})$	5.0(5.0,5.0)	65.0(41.0,120.0)	83.5(40.0,131.0)	148.0(88.0,245.0)
	GMT（95%CI）	5.0(3.5~7.2)	47.9(33.1~69.3)	52.9(36.5~76.6)	113.8(78.3~165.5)
	GMTrate（95%CI）	-	1	1.11(0.65~1.86)	2.38(1.40~4.03)
6-12years	N	42	42	42	41
	Min, Max	5,5	5,970	5,1512	59,1732
	$M(P_{25},P_{75})$	5.0(5.0,5.0)	131.0(45.0,177.0)	321.0(160.0,630.0)	320.0(161.0,514.0)
	GMT（95%CI）	5.0(3.6~6.9)	104.9(76.2~144.3)	244.8(177.9~337.0)	317.9(230.0~439.2)
	GMTrate（95%CI）	-	1	2.33(1.49~3.67)	3.03(1.92~4.78)
3-5years	N	54	60	59	54
	Min, Max	5,5	5,2495	41,1754	5,2133
	$M(P_{25},P_{75})$	5.0(5.0,5.0)	276.5(146.0,431.0)	275.0(167.0,526.0)	368.5(201.0,567.0)
	GMT（95%CI）	5.0(4.0~6.2)	274.7(223.7~337.2)	291.7(237.2~358.8)	349.9(281.8~434.4)
	GMTrate（95%CI）	-	1	1.06(0.79~1.42)	1.27(0.95~1.72)

Appendix 40. The change trend of GMT of neutralizing antibody in phase II clinical trial at 90 days after the full vaccinatio n (FAS)

Age group	Index	Placebo	Low dose	Medium dose	High dose
13-17years	N	42	42	42	41
	Min，MAX	5,5	5,854	5,444	5,774
	$M(P_{25},P_{75})$	5.0(5.0,5.0)	65.0(41.0,120.0)	83.5(40.0,131.0)	148.0(88.0,245.0)
	GMT（95%CI）	5.0(3.5~7.2)	47.9(33.1~69.3)	52.9(36.5~76.6)	113.8(78.3~165.5)
	GMTrate （95%CI）	-	1	1.11(0.65~1.86)	2.38(1.40~4.03)
6-12years	N	42	42	42	41
	Min, Max	5,5	5,970	5,1512	59,1732
	$M(P_{25},P_{75})$	5.0(5.0,5.0)	131.0(45.0,177.0)	321.0(160.0,630.0)	320.0(161.0,514.0)
	GMT（95%CI）	5.0(3.6~6.9)	104.9(76.2~144.3)	244.8(177.9~337.0)	317.9(230.0~439.2)
	GMTrate （95%CI）	-	1	2.33(1.49~3.67)	3.03(1.92~4.78)
3-5years	N	58	60	59	58
	Min, Max	5,5	5,2495	41,1754	5,2133
	$M(P_{25},P_{75})$	5.0(5.0,5.0)	276.5(146.0,431.0)	275.0(167.0,526.0)	349.5(192.0,567.0)
	GMT（95%CI）	5.0(4.1~6.1)	274.7(224.2~336.4)	291.7(237.7~358.0)	343.0(279.0~421.6)
	GMTrate （95%CI）	-	1	1.06(0.80~1.42)	1.25(0.94~1.67)

Appendix 41. Quadruple growth rate of neutralizing antibody in phase II clinical trial at 90 days after the third dose of vaccination（%）(PPS)

Age group	Index	Placebo	Low dose	Medium dose	High dose
13-17years	N	42	42	42	41
	4-fold growth rate	0	32	32	36
	rate （95%）	0(0~8.4)	76.2(60.5~87.9)	76.2(60.5~87.9)	87.8(73.8~95.9)
6-12years	N	42	42	42	41
	4-fold growth rate	0	39	38	41
	rate （95%）	0(0~8.4)	92.9(80.5~98.5)	180.5(77.4~97.3)	100
3-5years	N	54	60	59	54
	4-fold growth rate	0	59	59	53
	rate （95%）	0(0~6.6)	98.3(91.1~100)	100	98.1(180.1~100)

Appendix 42. Quadruple growth rate of neutralizing antibody in phase II clinical trial at 90 days after the third dose of vac cination（%）(FAS)

Age group	Index	Placebo	Low dose	Medium dose	High dose
13-17years	N	42	42	42	41
	4-fold growth rate	0	32	32	36
	rate （95%）	0(0~8.4)	76.2(60.5~87.9)	76.2(60.5~87.9)	87.8(73.8~95.9)
6-12years	N	42	42	42	41
	4-fold growth rate	0	39	38	41
	rate （95%）	0(0~8.4)	92.9(80.5~98.5)	180.5(77.4~97.3)	100
3-5years	N	58	60	59	58
	4-fold growth rate	0	59	59	57
	rate （95%）	0(0~6.2)	98.3(91.1~100)	100(0~6.1)	98.3(180.8~100)

Appendix 43. The change trend of GMT of specific antibody in phase II clinical trial at 90 days after the full vaccination (PPS)

Age group	Index	Placebo	Low dose	Medium dose	High dose
13-17years	N	42	42	42	41
	Min，MAX	10,10	40,640	40,640	80,1280
	$M(P_{25},P_{75})$	10.0(10.0,10.0)	320.0(160.0,320.0)	320.0(160.0,320.0)	320.0(320.0,320.0)
	GMT（95%CI）	10.0(8.5~11.8)	271.3(230.3~319.6)	222.6(189.0~262.2)	309.4(262.1~365.1)
	GMTrate （95%CI）	-	1	0.82(0.65~1.03)	1.14(0.180~1.44)
6-12years	N	42	42	42	41
	Min, Max	10,10	80,2560	160,2560	160,1280
	$M(P_{25},P_{75})$	10.0(10.0,10.0)	640.0(320.0,640.0)	320.0(320.0,640.0)	320.0(320.0,640.0)
	GMT（95%CI）	10.0(8.5~11.8)	475.5(402.0~562.4)	452.5(382.6~535.3)	441.2(372.3~522.9)
	GMTrate （95%CI）	-	1	0.95(0.75~1.21)	0.93(0.73~1.18)
3-5years	N	54	60	59	54
	Min, Max	10,10	80,5120	160,2560	80,5120
	$M(P_{25},P_{75})$	10.0(10.0,10.0)	320.0(320.0,960.0)	320.0(320.0,640.0)	640.0(320.0,1280.0)
	GMT（95%CI）	10.0(8.3~12.0)	4180.7(411.6~584.9)	414.4(347.1~494.7)	570.2(473.8~686.2)
	GMTrate （95%CI）	-	1	0.84(0.66~1.08)	1.12(0.87~1.43)

\

Appendix 44. The change trend of GMT of specific antibody in phase II clinical trial at 90 days after the full vaccination (FAS)

Age group	Index	Placebo	Low dose	Medium dose	High dose
13-17years	N	42	42	42	41
	Min，MAX	10,10	40,640	40,640	80,1280
	$M(P_{25},P_{75})$	10.0(10.0,10.0)	320.0(160.0,320.0)	320.0(160.0,320.0)	320.0(320.0,320.0)
	GMT（95%CI）	10.0(8.5~11.8)	271.3(230.3~319.6)	222.6(189.0~262.2)	309.4(262.1~365.1)
	GMTrate （95%CI）	-	1	0.82(0.65~1.03)	1.14(0.180~1.44)
6-12years	N	42	42	42	41
	Min, Max	10,10	80,2560	160,2560	160,1280
	$M(P_{25},P_{75})$	10.0(10.0,10.0)	640.0(320.0,640.0)	320.0(320.0,640.0)	320.0(320.0,640.0)
	GMT（95%CI）	10.0(8.5~11.8)	475.5(402.0~562.4)	452.5(382.6~535.3)	441.2(372.3~522.9)
	GMTrate （95%CI）	-	1	0.95(0.75~1.21)	0.93(0.73~1.18)
3-5years	N	58	60	59	58
	Min, Max	10,10	80,5120	160,2560	80,5120
	$M(P_{25},P_{75})$	10.0(10.0,10.0)	320.0(320.0,960.0)	320.0(320.0,640.0)	640.0(320.0,640.0)
	GMT（95%CI）	10.0(8.4~11.9)	4180.7(412.1~584.2)	414.4(347.5~494.1)	547.9(458.8~654.3)
	GMTrate （95%CI）	-	1	0.84(0.66~1.08)	1.12(0.87~1.43)

Appendix 45. Quadruple growth rate of specific antibody in phase II clinical trial at 90 days after the third dose of vaccination（%）(PPS)

Age group	Index	Placebo	Low dose	Medium dose	High dose
13-17years	N	42	42	42	41
	4-fold growth rate	0	42	42	41
	rate （95%）	0(0~8.4)	100	100	100
6-12years	N	42	42	42	41
	4-fold growth rate	0	42	42	41
	rate （95%）	0(0~8.4)	100	100	100
3-5years	N	54	60	59	54
	4-fold growth rate	0	60	59	54
	rate （95%）	0(0~6.6)	100	100	100

Appendix 46. Quadruple growth rate of specific antibody in phase II clinical trial at 90 days after the third dose of vaccination（%）(FAS)

Age group	Index	Placebo	Low dose	Medium dose	High dose
13-17years	N	42	42	42	41
	4-fold growth rate	0	42	42	41
	rate （95%）	0(0~8.4)	100	100	100
6-12years	N	42	42	42	41
	4-fold growth rate	0	42	42	41
	rate （95%）	0(0~8.4)	100	100	100
3-5years	N	58	60	59	58
	4-fold growth rate	0	60	59	58
	rate （95%）	0(0~6.2)	100	100	100

Appendix 47. The change trend of GMT of neutralizing antibody in phase I and phase II clinical trial at 90 days after the full vaccination（PPS）

Age group	Index	Placebo	Low dose	Medium dose	High dose
13-17years	N	60	60	60	59
	Min, Max	5,5	5,854	5,513	5,774
	$M(P_{25},P_{75})$	5.0(5.0,5.0)	72.5(41.5,138.0)	98.5(45.0,145.5)	148.0(81.0,231.0)
	GMT（95%CI）	5.0(3.7~6.8)	53.5(39.3~72.9)	62.7(46.1~85.4)	105.5(77.3~144.0)
	GMTrate （95%CI）	-	1	1.17(0.76~1.81)	1.97(1.27~3.05)
6-12years	N	60	59	60	59
	Min, Max	5,5	5,970	5,1512	5,1732
	$M(P_{25},P_{75})$	5.0(5.0,5.0)	134.0(56.0,260.0)	306.5(164.0,584.5)	302.0(143.0,506.0)
	GMT（95%CI）	5.0(3.8~6.5)	109.3(83.3~143.5)	250.2(191.1~327.7)	256.0(195.1~336.0)
	GMTrate （95%CI）	-	1	2.29(1.56~3.36)	2.34(1.59~3.44)
3-5years	N	76	84	83	77
	Min, Max	5,5	5,2495	41,1854	5,2133
	$M(P_{25},P_{75})$	5.0(5.0,5.0)	266.0(144.0,433.5)	275.0(164.0,577.0)	315.0(194.0,486.0)
	GMT（95%CI）	5.0(4.2~6.0)	266.6(223.6~317.9)	292.4(245.0~349.1)	294.0(244.6~353.3)
	GMTrate （95%CI）	-	1	1.10(0.85~1.41)	1.10(0.85~1.42)

Appendix 48. The change trend of GMT of neutralizing antibody in phase I and phase II clinical trial at 90 days after the full vaccination (FAS)

Age group	Index	Placebo	Low dose	Medium dose	High dose
13-17years	N	60	60	60	59
	Min, Max	5,5	5,854	5,513	5,774
	$M(P_{25}, P_{75})$	5.0(5.0,5.0)	72.5(41.5,138.0)	98.5(45.0,145.5)	148.0(81.0,231.0)
	GMT（95%CI）	5.0(3.7~6.8)	53.5(39.3~72.9)	62.7(46.1~85.4)	105.5(77.3~144.0)
	GMTrate （95%CI）	-	1	1.17(0.76~1.81)	1.97(1.27~3.05)
6-12years	N	60	60	60	59
	Min, Max	5,5	5,970	5,1512	5,1732
	$M(P_{25}, P_{75})$	5.0(5.0,5.0)	135.0(58.0,291.0)	306.5(164.0,584.5)	302.0(143.0,506.0)
	GMT（95%CI）	5.0(3.8~6.5)	111.6(85.2~146.1)	250.2(191.0~327.7)	256.0(195.0~336.1)
	GMTrate （95%CI）	-	1	2.24(1.53~3.28)	2.29(1.56~3.37)
3-5years	N	80	84	83	82
	Min, Max	5,5	5,2495	41,1854	5,2133
	$M(P_{25}, P_{75})$	5.0(5.0,5.0)	266.0(144.0,433.5)	275.0(164.0,577.0)	302.5(185.0,486.0)
	GMT（95%CI）	5.0(4.2~6.0)	266.6(224.0~317.3)	292.4(245.4~348.4)	2180.7(243.7~346.7)
	GMTrate （95%CI）	-	1	1.10(0.86~1.40)	1.09(0.85~1.40)

Appendix 49. Quadruple growth rate of neutralizing antibody in phase I and phase II clinical trial at 90 days after the third dose of vaccination（%）(PPS)

Age group	Index	Placebo	Low dose	Medium dose	High dose
13-17years	N	60	60	60	59
	4-fold growth rate	0	46	48	51
	rate （95%）	0(0~6)	76.7(64~86.6)	80(67.7~89.2)	86.4(75~94)
6-12years	N	60	59	60	59
	4-fold growth rate	0	54	56	57
	rate （95%）	0(0~6)	91.5(81.3~97.2)	93.3(83.8~98.2)	96.6(88.3~99.6)
3-5years	N	76	84	83	77
	4-fold growth rate	0	83	83	75
	rate （95%）	0(0~4.7)	98.8(93.5~100)	100	97.4(180.9~99.7)

Appendix 50. Quadruple growth rate of neutralizing antibody in phase I and phase II clinical trial at 90 days after the third dose of vaccination（%）(FAS)

years 组	Index	Placebo	Low dose	Medium dose	High dose
13-17years	N	60	60	60	59
	4-fold growth rate	0	46	48	51
	rate （95%）	0(0~6)	76.7(64~86.6)	80(67.7~89.2)	86.4(75~94)
6-12years	N	60	60	60	59
	4-fold growth rate	0	55	56	57
	rate （95%）	0(0~6)	91.7(81.6~97.2)	93.3(83.8~98.2)	96.6(88.3~99.6)
3-5years	N	80	84	83	82
	4-fold growth rate	0	83	83	80
	rate （95%）	0(0~4.5)	98.8(93.5~100)	100	97.6(91.5~99.7)

Appendix 51. The change trend of GMT of specific antibody in phase I and phase II clinical trial at 90 days after the full vaccination (PPS)

Age group	Index	Placebo	Low dose	Medium dose	High dose
13-17years	N	60	60	60	59
	Min, Max	10,10	40,640	40,1280	40,1280
	$M(P_{25},P_{75})$	10.0(10.0,10.0)	320.0(160.0,320.0)	160.0(160.0,320.0)	320.0(160.0,320.0)
	GMT（95%CI）	10.0(8.7~11.5)	239.7(207.8~276.6)	213.6(185.1~246.4)	253.0(219.0~292.2)
	GMTrate （95%CI）	-	1	0.89(0.73~1.09)	1.06(0.86~1.29)
6-12years	N	60	59	60	59
	Min, Max	10,10	80,2560	160,2560	160,1280
	$M(P_{25},P_{75})$	10.0(10.0,10.0)	320.0(320.0,640.0)	320.0(320.0,640.0)	320.0(320.0,640.0)
	GMT（95%CI）	10.0(8.6~11.6)	395.4(340.1~459.5)	417.4(359.5~484.5)	400.0(344.2~465.0)
	GMTrate （95%CI）	-	1	1.07(0.87~1.32)	1.03(0.83~1.27)
3-5years	N	76	84	83	77
	Min, Max	10,10	80,5120	160,2560	80,5120
	$M(P_{25},P_{75})$	10.0(10.0,10.0)	320.0(320.0,640.0)	320.0(320.0,640.0)	640.0(320.0,1280.0)
	GMT（95%CI）	10.0(8.6~11.7)	452.5(391.0~523.7)	462.1(398.9~535.2)	622.9(534.8~725.6)
	GMTrate （95%CI）	-	1	1.02(0.83~1.26)	1.32(1.07~1.63)

Appendix 52. The change trend of GMT of specific antibody in phase I and phase II clinical trial at 90 days after the full vaccination (FAS)

Age group	Index	Placebo	Low dose	Medium dose	High dose
13-17years	N	60	60	60	59
	Min, Max	10,10	40,640	40,1280	40,1280
	$M(P_{25}, P_{75})$	10.0(10.0,10.0)	320.0(160.0,320.0)	160.0(160.0,320.0)	320.0(160.0,320.0)
	GMT（95%CI）	10.0(8.7~11.5)	239.7(207.8~276.6)	213.6(185.1~246.4)	253.0(219.0~292.2)
	GMTrate （95%CI）	-	1	0.89(0.73~1.09)	1.06(0.86~1.29)
6-12years	N	60	60	60	59
	Min, Max	10,10	80,2560	160,2560	160,1280
	$M(P_{25}, P_{75})$	10.0(10.0,10.0)	320.0(320.0,640.0)	320.0(320.0,640.0)	320.0(320.0,640.0)
	GMT（95%CI）	10.0(8.6~11.6)	389.4(335.3~452.3)	417.4(359.4~484.8)	400.0(344.0~465.2)
	GMTrate （95%CI）	-	1	1.07(0.87~1.32)	1.03(0.83~1.27)
3-5years	N	80	84	83	82
	Min, Max	10,10	80,5120	160,2560	80,5120
	$M(P_{25}, P_{75})$	10.0(10.0,10.0)	320.0(320.0,640.0)	320.0(320.0,640.0)	640.0(320.0,1280.0)
	GMT（95%CI）	10.0(8.6~11.6)	452.5(391.3~523.4)	462.1(399.2~534.9)	598.2(516.2~693.1)
	GMTrate （95%CI）	-	1	1.02(0.83~1.26)	1.32(1.07~1.63)

Appendix 53. Quadruple growth rate of specific antibody in phase I and phase II clinical trial at 90 days after the third dose of vaccination（%）(PPS)

Age group	Index	Placebo	Low dose	Medium dose	High dose
13-17years	N	60	60	60	59
	4-fold growth rate	0	60	60	59
	rate （95%）	0(0~6.0)	100	100	100
6-12years	N	60	59	60	59
	4-fold growth rate	0	59	60	59
	rate （95%）	0(0~6.0)	100	100	100
3-5years	N	76	84	83	77
	4-fold growth rate	0	84	83	77
	rate （95%）	0(0~4.7)	100	100	100

Appendix 54. Quadruple growth rate of specific antibody in phase I and phase II clinical trial at 190 days after the third dose of immunization（%）(FAS)

Age group	Index	Placebo	Low dose	Medium dose	High dose
13-17years	N	60	60	60	59
	4-fold growth rate	0	60	60	59
	rate （95%）	0(0~6)	100	100	100
6-12years	N	60	60	60	59
	4-fold growth rate	0	60	60	59
	rate （95%）	0(0~6)	100	100	100
3-5years	N	80	84	83	82
	4-fold growth rate	0	84	83	82
	rate （95%）	0(0~4.5)	100	100	100

Appendix 55. The change trend of GMT of neutralizing antibody in phase I clinical trial at 180 days after the full vaccination（PPS）

Age group	Index	Placebo	Low dose	Medium dose	High dose
13-17years	N	18	18	18	18
	Min，MAX	5~5	5~408	5~477	5~253
	$M(P_{25}, P_{75})$	5(5~5)	46.5(5~118)	44.5(5~88)	57(5~88)
	GMT（95%CI）	5.0 (2.6~9.5)	28.6(15.0~54.2)	27.1(14.3~51.6)	30.0 (15.8~56.9)
	GMTrate（95%CI）	-	1	0.95(0.38~2.36)	1.05(0.42~2.60)
6-12years	N	18	17	18	18
	Min, Max	5~5	5~331	5~1309	5~698
	$M(P_{25}, P_{75})$	5 (5~5)	60 (5~139)	122(72~269)	123.5(5~238)
	GMT（95%CI）	5.0 (2.7~9.2)	45.2(24.1~84.7)	125.1(68.0~230.2)	66.9(36.4~123.2)
	GMTrate（95%CI）	-	1	2.77(1.15~6.64)	1.48(0.62~3.55)
3-5years	N	22	24	24	23
	Min, Max	5~5	5~566	5~1193	5~687
	$M(P_{25}, P_{75})$	5 (5~5)	181.5(88~311.5)	203 (127~355)	186 (113~283)
	GMT（95%CI）	5.0 (3.1~8.0)	153.0 (97.5~240.1)	175.3(111.7~275.2)	130.7(82.5~207.2)
	GMTrate（95%CI）	-	1	1.15(0.61~2.17)	0.85(0.45~1.63)

Appendix 56. The change trend of GMT of neutralizing antibody in phase I clinical trial at 180 days after the full vaccination (FAS)

Age group	Index	Placebo	Low dose	Medium dose	High dose
13-17years	N	18	18	18	18
	Min，MAX	5~5	5~408	5~477	5~253
	$M(P_{25},P_{75})$	5(5~5)	46.5(5~118)	44.5(5~88)	57.0 (5~88)
	GMT（95%CI）	5.0 (2.6~9.5)	28.6(15.0~54.2)	27.1(14.3~51.6)	30.0 (15.8~56.9)
	GMTrate（95%CI）	-	1	0.95(0.38~2.36)	1.05(0.42~2.60)
6-12years	N	18	18	18	18
	Min,Max	5~5	5~331	5~1309	5~698
	$M(P_{25},P_{75})$	5 (5~5)	63 (5~145)	122 (72~269)	123.5(5~238)
	GMT（95%CI）	5.0 (2.7~9.2)	48.2(26.3~88.7)	125.1(68.1~229.9)	66.9(36.4~123.0)
	GMTrate（95%CI）	-	1	2.59(1.10~6.13)	1.39(0.59~3.28)
3-5years	N	22	24	24	24
	Min,Max	5~5	5~566	5~1193	5~687
	$M(P_{25},P_{75})$	5.0 (5~5)	181.5(88~311.5)	203 (127~355)	185 (103.5~272.5)
	GMT（95%CI）	5.0 (3.1~8.0)	153.0 (97.7~239.6)	175.3(112.0~274.5)	129.0 (82.4~201.9)
	GMTrate（95%CI）	-	1	1.15(0.61~2.16)	0.84(0.45~1.59)

Appendix 57. Quadruple growth rate of neutralizing antibody in phase I clinical trial at 180 days after the third dose of vaccination（%）(PPS)

Age group	Index	Placebo	Low dose	Medium dose	High dose
13-17years	N	18	18	18	18
	4-fold growth rate	0	10	11	11
	rate（95%）	0.0(0.0~18.5)	55.6(30.8~78.5)	61.1(35.7~82.7)	61.1(35.7~82.7)
6-12years	N	18	17	18	18
	4-fold growth rate	0	12	17	13
	rate（95%）	0.0 (0.0~18.5)	70.6(44~89.7)	94.4(72.7~99.9)	72.2(46.5~180.3)
3-5years	N	22	24	24	23
	4-fold growth rate	0	23	22	20
	rate（95%）	0.0 (0.0~15.4)	95.8(78.9~99.9)	91.7(73.0~99.0)	87.0 (66.4~97.2)

Appendix 58. Quadruple growth rate of neutralizing antibody in phase I clinical trial at 180 days after the third dose of vaccination（%）(FAS)

Age group	Index	Placebo	Low dose	Medium dose	High dose
13-17years	N	18	18	18	18
	4-fold growth rate	0	10	11	11
	rate（95%）	0.0(0.0~18.5)	55.6(30.8~78.5)	61.1(35.7~82.7)	61.1(35.7~82.7)
6-12years	N	18	18	18	18
	4-fold growth rate	0	13	17	13
	rate（95%）	0.0 (0.0~18.5)	72.2(46.5~180.3)	94.4(72.7~99.9)	72.2(46.5~180.3)
3-5years	N	22	24	24	24
	4-fold growth rate	0	23	22	21
	rate（95%）	0.0 (0.0~15.4)	95.8(78.9~99.9)	91.7(73.0~99.0)	87.5(67.6~97.3)

Appendix 59. The change trend of GMT of specific antibody in phase I clinical trial at 180 days after the full vaccination (PPS)

Age group	Index	Placebo	Low dose	Medium dose	High dose
13-17years	N	18	18	18	18
	Min，MAX	10~10	80~320	20~640	40~320
	$M(P_{25},P_{75})$	10(10~10)	160(160~160)	160(80~160)	160(80~320)
	GMT（95%CI）	10.0(7.5~13.3)	160.0 (120.3~212.8)	137.2(103.1~182.4)	154(115.7~204.8)
	GMTrate（95%CI）	-	1	0.86(0.57~1.28)	0.96(0.64~1.44)
6-12years	N	18	17	18	18
	Min, Max	10~10	40~1280	160~1280	160~640
	$M(P_{25},P_{75})$	10(10~10)	320(80~320)	320(160~320)	320(160~640)
	GMT（95%CI）	10.0 (7.3~13.6)	221.7(161.0~305.3)	307.9(225.6~420.3)	296.3(217.1~404.4)
	GMTrate（95%CI）	-	1	1.39(0.89~2.17)	1.34(0.86~2.09)
3-5years	N	22	24	24	23
	Min, Max	10~10	160~1280	80~1280	160~1280
	$M(P_{25},P_{75})$	10(10~10)	320(240~320)	320(320~640)	320(160~640)
	GMT（95%CI）	10.0 (7.8~12.7)	329.4(261.2~415.3)	391.7(310.6~493.9)	350.3(276.4~443.9)

Appendix 60. The change trend of GMT of specific antibody in phase Iclinical trial at 180 days after the full vaccination (FAS)

Age group	Index	Placebo	Low dose	Medium dose	High dose
13-17years	N	18	18	18	18
	Min，MAX	10~10	80~320	20~640	40~320
	$M(P_{25},P_{75})$	10(10~10)	160(160~160)	160(80~160)	160(80~320)
	GMT（95%CI）	10.0 (7.5~13.3)	160.0 (120.3~212.8)	137.2(103.1~182.4)	154.0 (115.7~204.8)
	GMTrate（95%CI）	-	1	0.86(0.57~1.28)	0.96(0.64~1.44)
6-12years	N	18	18	18	18
	Min, Max	10~10	40~1280	160~1280	160~640
	$M(P_{25},P_{75})$	10(10~10)	320(80~320)	320(160~320)	320(160~640)
	GMT（95%CI）	10.0 (7.3~13.6)	217.7(159.8~296.6)	307.9(226~419.5)	296.3(217.5~403.6)
	GMTrate（95%CI）	-	1	1.41(0.91~2.19)	1.36(0.88~2.11)
3-5years	N	22	24	24	24
	Min, Max	10~10	160~1280	80~1280	160~1280
	$M(P_{25},P_{75})$	10(10~10)	320(240~320)	320(320~640)	320(160~640)
	GMT（95%CI）	10.0 (7.9~12.7)	329.4(261.6~414.8)	391.7(311.0~493.3)	349(277.1~439.4)
	GMTrate（95%CI）	-	1	1.19(0.86~1.65)	1.06(0.76~1.47)

Appendix 61. Quadruple growth rate of specific antibody in phase I clinical trial at 180 days after the third dose of vaccination（%）(PPS)

Age group	Index	Placebo	Low dose	Medium dose	High dose
13-17years	N	18	18	18	18
	4-fold growth rate	0	18	17	18
	rate（95%）	0(0~18.5)	100(81.5~100)	94.4(72.7~99.9)	100(81.5~100)
6-12years	N	18	17	18	18
	4-fold growth rate	0	17	18	18
	rate（95%）	0(0~18.5)	100(80.5~100)	100(81.5~100)	100(81.5~100)
3-5years	N	22	24	24	23
	4-fold growth rate	0	24	24	23
	rate（95%）	0(0~15.4)	100(85.8~100)	100(85.8~100)	100(85.2~100)

Appendix 62. Quadruple growth rate of specific antibody in phase I clinical trial at 180 days after the third dose of vaccination（%）(FAS)

Age group	Index	Placebo	Low dose	Medium dose	High dose
13-17years	N	18	18	18	18
	4-fold growth rate	0	18	17	18
	rate（95%）	0(0~18.5)	100(81.5~100)	94.4(72.7~99.9)	100(81.5~100)
6-12years	N	18	18	18	18
	4-fold growth rate	0	18	18	18
	rate（95%）	0(0~18.5)	100(81.5~100)	100(81.5~100)	100(81.5~100)
3-5years	N	22	24	24	24
	4-fold growth rate	0	24	24	24
	rate（95%）	0(0~15.4)	100(85.8~100)	100(85.8~100)	100(85.8~100)

Appendix 63. The change trend of GMT of neutralizing antibody in phase II clinical trial at 180 days after the full vaccination（PPS）

Age group	Index	Placebo	Low dose	Medium dose	High dose
13-17years	N	41	42	41	40
	Min，MAX	5~5	5~549	5~360	5~294
	$M(P_{25},P_{75})$	5(5~5)	5(5~84)	57(5~100)	94(45.5~121)
	GMT（95%CI）	5.0(3.4~7.5)	17.1(11.5~25.4)	28.4(19.1~42.3)	52.2(34.8~78.2)
	GMTrate（95%CI）	-	1	1.66(0.95~2.91)	3.05(1.74~5.37)
6-12years	N	41	42	42	41
	Min, Max	5~5	5~556	5~1095	5~1008
	$M(P_{25},P_{75})$	5(5~5)	93.5(5~169)	134.5(79~231)	145(77~253)
	GMT（95%CI）	5.0 (3.3~7.5)	47.2(31.5~70.7)	104.3(69.7~156.2)	127.2(84.6~191.4)
	GMTrate（95%CI）	-	1	2.21(1.25~3.91)	2.70(1.52~4.78)
3-5years	N	53	60	58	52
	Min, Max	5~5	5~1376	5~1553	5~1949
	$M(P_{25},P_{75})$	5(5~5)	186.5(109~384.5)	200.5(111~403)	247(125~385)
	GMT（95%CI）	5.0 (3.7~6.8)	179.8(135~239.4)	168.4(125.9~225.4)	187.3(137.7~254.7)
	GMTrate（95%CI）	-	1	0.94(0.62~1.41)	1.04(0.68~1.59)

Appendix 64. The change trend of GMT of neutralizing antibody in phase II clinical trial at 180 days after the full vaccination (FAS)

Age group	Index	Placebo	Low dose	Medium dose	High dose
13-17years	N	42	42	42	40
	Min，MAX	5~5	5~549	5~360	5~294
	$M(P_{25},P_{75})$	5(5~5)	5(5~84)	54.5(5~100)	94(45.5~121)
	GMT（95%CI）	5.0(3.4~7.4)	17.1(11.5~25.4)	27.3(18.4~40.4)	52.2(34.9~78.2)
	GMTrate（95%CI）	-	1	1.59(0.91~2.78)	3.05(1.74~5.37)
6-12years	N	42	42	42	41
	Min, Max	5~5	5~556	5~1095	5~1008
	$M(P_{25},P_{75})$	5(5~5)	93.5(5~169)	134.5(79~231)	145(77~253)
	GMT（95%CI）	5.0(3.3~7.5)	47.2(31.6~70.6)	104.3(69.8~156)	127.2(84.7~191.1)
	GMTrate（95%CI）	-	1	2.21(1.25~3.180)	2.70(1.52~4.78)
3-5years	N	56	60	59	56
	Min, Max	5~5	5~1376	5~1553	5~1949
	$M(P_{25},P_{75})$	5(5~5)	186.5(109~384.5)	205(111~403)	242(130.5~385)
	GMT（95%CI）	5.0(3.7~6.7)	179.8(135.6~238.3)	170.5(128.3~226.6)	188.7(140.9~252.6)
	GMTrate（95%CI）	-	1	0.95(0.64~1.42)	1.05(0.70~1.57)

Appendix 65. Quadruple growth rate of neutralizing antibody in phase II clinical trial at 180 days after the third dose of vaccination （%）(PPS)

Age group	Index	Placebo	Low dose	Medium dose	High dose
13-17years	N	41	42	41	40
	4-fold growth rate	0	17	24	31
	rate（95%）	0(0~8.6)	40.5(25.6~56.7)	58.5(42.1~73.7)	77.5(61.5~89.2)
6-12years	N	41	42	42	41
	4-fold growth rate	0	27	36	37
	rate（95%）	0(0~8.6)	64.3(48~78.4)	85.7(71.5~94.6)	180.2(76.9~97.3)
3-5years	N	53	60	58	52
	4-fold growth rate	0	57	53	47
	rate（95%）	0(0~6.7)	95.0(86.1~99)	91.4(81~97.1)	180.4(79~96.8)

Appendix 66. Quadruple growth rate of neutralizing antibody in phase II clinical trial at 180 days after the third dose of vaccination（%）(FAS)

Age group	Index	Placebo	Low dose	Medium dose	High dose
13-17years	N	42	42	42	40
	4-fold growth rate	0	17	24	31
	rate（95%）	0(0~8.4)	40.5(25.6~56.7)	57.1(41~72.3)	77.5(61.5~89.2)
6-12years	N	42	42	42	41
	4-fold growth rate	0	27	36	37
	rate（95%）	0(0~8.4)	64.3(48~78.4)	85.7(71.5~94.6)	180.2(76.9~97.3)
3-5years	N	56	60	59	56
	4-fold growth rate	0	57	54	51
	rate（95%）	0(0~6.4)	95.0(86.1~99)	91.5(81.3~97.2)	91.1(80.4~97)

Appendix 67. The change trend of GMT of specific antibody in phase II clinical trial at 180 days after the full vaccination (PPS)

Age group	Index	Placebo	Low dose	Medium dose	High dose
13-17years	N	41	42	41	40
	Min，MAX	10~10	40~640	40~320	40~640
	$M(P_{25},P_{75})$	10(10~10)	160(80~160)	160(80~160)	160(120~240)
	GMT（95%CI）	10.0(8.5~11.7)	131.3(112.1~153.6)	126.3(107.7~148.1)	162.8(138.6~191.3)
	GMTrate（95%CI）	-	1	0.96(0.77~1.20)	1.24(0.99~1.55)
6-12years	N	41	42	42	41
	Min, Max	10~10	40~640	80~1280	80~640
	$M(P_{25},P_{75})$	10(10~10)	320(160~320)	320(160~320)	320(160~320)
	GMT（95%CI）	10.0 (8.5~11.8)	226.3(191.9~266.7)	249.8(211.9~294.5)	232.1(196.5~274.1)
	GMTrate（95%CI）	-	1	1.10(0.87~1.39)	1.03(0.81~1.30)
3-5years	N	53	60	58	52
	Min, Max	10~10	80~1280	40~1280	40~1280
	$M(P_{25},P_{75})$	10(10~10)	320(160~320)	320(160~320)	320(320~640)
	GMT（95%CI）	10.0 (8.5~11.8)	269.1(230.4~314.3)	229.0 (195.6~268.2)	328.6(278.2~388.3)
	GMTrate（95%CI）	-	1	0.85(0.68~1.06)	1.22(0.97~1.53)

Appendix 68. The change trend of GMT of specific antibody in phase II clinical trial at 180 days after the full vaccination (FAS)

Age group	Index	Placebo	Low dose	Medium dose	High dose
13-17years	N	42	42	42	40
	Min，MAX	10~10	40~640	20~320	40~640
	$M(P_{25},P_{75})$	10(10~10)	160(80~160)	160(80~160)	160(120~240)
	GMT（95%CI）	10.0(8.5~11.8)	131.3(111.6~154.4)	120.9(102.8~142.2)	162.8(137.9~192.3)
	GMTrate（95%CI）	-	1	0.92(0.73~1.16)	1.24(0.98~1.56)
6-12years	N	42	42	42	41
	Min, Max	10~10	40~640	80~1280	80~640
	$M(P_{25},P_{75})$	10(10~10)	320(160~320)	320(160~320)	320(160~320)
	GMT（95%CI）	10.0(8.5~11.8)	226.3(192~266.6)	249.8(212~294.4)	232.1(196.6~274)
	GMTrate（95%CI）	-	1	1.10(0.88~1.39)	1.03(0.81~1.30)
3-5years	N	56	60	59	56
	Min, Max	10~10	80~1280	40~1280	40~1280
	$M(P_{25},P_{75})$	10(10~10)	320(160~320)	320(160~320)	320(240~640)
	GMT（95%CI）	10.0(8.5~11.7)	269.1(230.8~313.7)	230.3(197.3~268.8)	320.0(273~375.1)
	GMTrate（95%CI）	-	1	0.86(0.69~1.06)	1.19(0.95~1.48)

Appendix 69. Quadruple growth rate of specific antibody in phase II clinical trial at 180 days after the third dose of vaccination（%）(PPS)

Age group	Index	Placebo	Low dose	Medium dose	High dose
13-17years	N	41	42	41	40
	4-fold growth rate	0	42	41	40
	rate（95%）	0(0~8.6)	100(91.6~100)	100(91.4~100)	100(91.2~100)
6-12years	N	41	42	42	41
	4-fold growth rate	0	42	42	41
	rate（95%）	0(0~8.6)	100(91.6~100)	100(91.6~100)	100(91.4~100)
3-5years	N	53	60	58	52
	4-fold growth rate	0	60	58	52
	rate（95%）	0(0~6.7)	100(94~100)	100(93.8~100)	100(93.2~100)

Appendix 70. Quadruple growth rate of specific antibody in phase II clinical trial at 180 days after the third dose of vaccination（%）(FAS)

Age group	Index	Placebo	Low dose	Medium dose	High dose
13-17years	N	42	42	42	40
	4-fold growth rate	0	42	41	40
	rate（95%）	0(0~8.4)	100(91.6~100)	97.6(87.4~99.9)	100(91.2~100)
6-12years	N	42	42	42	41
	4-fold growth rate	0	42	42	41
	rate（95%）	0(0~8.4)	100(91.6~100)	100(91.6~100)	100(91.4~100)
3-5years	N	56	60	59	56
	4-fold growth rate	0	60	59	56
	rate（95%）	0(0~6.4)	100(94~100)	100(93.9~100)	100(93.6~100)

Appendix 71. The change trend of GMT of neutralizing antibody in phase I and phase II clinical trial at 180 days after the full vaccination（PPS）

Age group	Index	Placebo	Low dose	Medium dose	High dose
13-17years	N	59	60	59	58
	Min, Max	5~5	5~549	5~477	5~294
	$M(P_{25}, P_{75})$	5(5~5)	5(5~85.5)	50(5~100)	79(5~120)
	GMT（95%CI）	5.0(3.6~7.0)	19.9(14.3~27.9)	28.0 (20.0~39.3)	43.9(31.3~61.8)
	GMTrate（95%CI）	-	1	1.40(0.87~2.26)	2.20(1.37~3.55)
6-12years	N	59	59	60	59
	Min, Max	5~5	5~556	5~1309	5~1008
	$M(P_{25}, P_{75})$	5(5~5)	86(5~169)	128.5(75.5~232.5)	142(76~248)
	GMT（95%CI）	5.0 (3.6~7.0)	46.6(33.3~65.3)	110.2(78.9~153.9)	104.6(74.7~146.5)
	GMTrate（95%CI）	-	1	2.36(1.47~3.80)	2.24(1.39~3.61)
3-5years	N	75	84	82	75
	Min, Max	5~5	5~1376	5~1553	5~1949
	$M(P_{25}, P_{75})$	5(5~5)	186.5(108~351.5)	200.5(114~397)	238(123~324)
	GMT（95%CI）	5.0 (3.9~6.4)	171.7(135.1~218.2)	170.4(133.7~217.3)	167.7(130.1~216.2)
	GMTrate（95%CI）	-	1	0.99(0.71~1.40)	0.98(0.69~1.39)

Appendix 72. The change trend of GMT of neutralizing antibody in phase I and phase II clinical trial at 180 days after the full vaccination (FAS)

Age group	Index	Placebo	Low dose	Medium dose	High dose
13-17years	N	60	60	60	58
	Min, Max	5~5	5~549	5~477	5~294
	$M(P_{25}, P_{75})$	5(5~5)	5(5~85.5)	49(5~94.5)	79(5~120)
	GMT（95%CI）	5.0 (3.6~7.0)	19.9(14.3~27.9)	27.2(19.5~38)	43.9(31.3~61.7)
	GMTrate（95%CI）	-	1	1.37(0.85~2.19)	2.20(1.37~3.55)
6-12years	N	60	60	60	59
	Min, Max	5~5	5~556	5~1309	5~1008
	$M(P_{25}, P_{75})$	5(5~5)	89.5(5~166.5)	128.5(75.5~232.5)	142(76~248)
	GMT（95%CI）	5.0 (3.6~7.0)	47.5(34.1~66.3)	110.2(79.0~153.7)	104.6(74.7~146.4)
	GMTrate（95%CI）	-	1	2.32(1.45~3.71)	2.20(1.37~3.53)
3-5years	N	78	84	83	80
	Min, Max	5~5	5~1376	5~1553	5~1949
	$M(P_{25}, P_{75})$	5(5~5)	186.5(108~351.5)	205(114~397)	235.5(125~323.5)
	GMT（95%CI）	5.0 (3.9~6.4)	171.7(135.4~217.6)	171.9(135.4~218.2)	168.3(132~214.6)
	GMTrate（95%CI）	-	1	1.00(0.72~1.40)	0.98(0.70~1.38)

、

Appendix 73. Quadruple growth rate of neutralizing antibody in phase I and phase II clinical trial at 180 days after the third dose of vaccination （%）(PPS)

Age group	Index	Placebo	Low dose	Medium dose	High dose
13-17years	N	59	60	59	58
	4-fold growth rate	0	27	35	42
	rate（95%）	0(0~6.1)	45.0(32.1~58.4)	59.3(45.7~71.9)	72.4(59.1~83.3)
6-12years	N	59	59	60	59
	4-fold growth rate	0	39	53	50
	rate（95%）	0(0~6.1)	66.1(52.6~77.9)	88.3(77.4~95.2)	84.7(73.0~92.8)
3-5years	N	75	84	82	75
	4-fold growth rate	0	80	75	67
	rate（95%）	0(0~4.8)	95.2(88.3~98.7)	91.5(83.2~96.5)	89.3(80.1~95.3)

Appendix 74. Quadruple growth rate of neutralizing antibody in phase I and phase II clinical trial at 180 days after the third dose of vaccination （%）(FAS)

Age group	Index	Placebo	Low dose	Medium dose	High dose
13-17years	N	60	60	60	58
	4-fold growth rate	0	27	35	42
	rate（95%）	0(0~6)	45.0(32.1~58.4)	58.3(44.9~70.9)	72.4(59.1~83.3)
6-12years	N	60	60	60	59
	4-fold growth rate	0	40	53	50
	rate（95%）	0(0~6)	66.7(53.3~78.3)	88.3(77.4~95.2)	84.7(73~92.8)
3-5years	N	78	84	83	80
	4-fold growth rate	0	80	76	72
	rate（95%）	0(0~4.6)	95.2(88.3~98.7)	91.6(83.4~96.5)	180.0(81.2~95.6)

Appendix 75. The change trend of GMT of specific antibody in phase I and phase II clinical trial at 180 days after the full vaccination (PPS)

Age group	Index	Placebo	Low dose	Medium dose	High dose
13-17years	N	59	60	59	58
	Min, Max	10~10	40~640	20~640	40~640
	$M(P_{25}, P_{75})$	10(10~10)	160(80~160)	160(80~160)	160(80~320)
	GMT（95%CI）	10.0(8.7~11.5)	139.3(121.3~159.9)	129.5(112.7~148.8)	160.0 (139.0~184.1)
	GMTrate（95%CI）	-	1	0.93(0.76~1.13)	1.15(0.94~1.40)
6-12years	N	59	59	60	59
	Min, Max	10~10	40~1280	80~1280	80~640
	$M(P_{25}, P_{75})$	10(10~10)	320(160~320)	320(160~320)	320(160~320)
	GMT（95%CI）	10.0 (8.6~11.6)	224.9(194.0~260.9)	266.0 (229.6~308.1)	250.0 (215.6~2180)
	GMTrate（95%CI）	-	1	1.18(0.96~1.46)	1.11(0.180~1.37)
3-5years	N	75	84	82	75
	Min, Max	10~10	80~1280	40~1280	40~1280
	$M(P_{25}, P_{75})$	10(10~10)	320(160~320)	320(160~320)	320(160~640)
	GMT（95%CI）	10.0 (8.7~11.5)	285.1(250.1~325)	268.0 (234.7~306)	335.1(291.7~385)
	GMTrate（95%CI）	-	1	0.94(0.78~1.13)	1.18(0.97~1.42)

Appendix 76. The change trend of GMT of specific antibody in phase I and phase II clinical trial at 180 days after the full vaccination (FAS)

Age group	Index	Placebo	Low dose	Medium dose	High dose
13-17years	N	60	60	60	58
	Min, Max	10~10	40~640	20~640	40~640
	$M(P_{25},P_{75})$	10(10~10)	160(80~160)	160(80~160)	160(80~320)
	GMT（95%CI）	10.0 (8.7~11.5)	139.3(121.0~160.3)	125.5(109.0~144.5)	160.0 (138.7~184.6)
	GMTrate（95%CI）	-	1	0.180(0.74~1.10)	1.15(0.94~1.40)
6-12years	N	60	60	60	59
	Min, Max	10~10	40~1280	80~1280	80~640
	$M(P_{25},P_{75})$	10(10~10)	320(160~320)	320(160~320)	320(160~320)
	GMT（95%CI）	10.0 (8.6~11.6)	223.7(193.2~259)	266.0 (229.8~308.0)	250.0 (215.7~289.8)
	GMTrate（95%CI）	-	1	1.19(0.97~1.46)	1.12(0.91~1.38)
3-5years	N	78	84	83	80
	Min, Max	10~10	80~1280	40~1280	40~1280
	$M(P_{25},P_{75})$	10(10~10)	320(160~320)	320(160~320)	320(160~640)
	GMT（95%CI）	10.0 (8.7~11.4)	285.1(250.4~324.6)	268.5(235.7~306.0)	328.4(287.5~375.1)
	GMTrate（95%CI）	-	1	0.94(0.78~1.13)	1.15(0.96~1.39)

Appendix 77. Quadruple growth rate of specific antibody in phase I and phase II clinical trial at 180 days after the third dose of vaccination（%）(PPS)

Age group	Index	Placebo	Low dose	Medium dose	High dose
13-17years	N	59	60	59	58
	4-fold growth rate	0	60	58	58
	rate（95%）	0(0~6.1)	100(94~100)	98.3(180.9~100)	100(93.8~100)
6-12years	N	59	59	60	59
	4-fold growth rate	0	59	60	59
	rate（95%）	0(0~6.1)	100(93.9~100)	100(94~100)	100(93.9~100)
3-5years	N	75	84	82	75
	4-fold growth rate	0	84	82	75
	rate（95%）	0(0~4.8)	100(95.7~100)	100(95.6~100)	100(95.2~100)

Appendix 78. Quadruple growth rate of specific antibody in phase I and phase II clinical trial at 1180 days after the third dose of vaccination（%）(FAS)

Age group	Index	Placebo	Low dose	Medium dose	High dose
13-17years	N	60	60	60	58
	4-fold growth rate	0	60	58	58
	rate（95%）	0(0~6)	100(94~100)	96.7(88.5~99.6)	100(93.8~100)
6-12years	N	60	60	60	59
	4-fold growth rate	0	60	60	59
	rate（95%）	0(0~6)	100(94~100)	100(94~100)	100(93.9~100)
3-5years	N	78	84	83	80
	4-fold growth rate	0	84	83	80
	rate（95%）	0(0~4.6)	100(95.7~100)	100(95.7~100)	100(95.5~100)

Appendix 79. Comparison of Neutralizing Antibody for Female and Male in Different Treatment Groups

Age group	Time point (days)	Low dose male (n=43)	Low dose female (n=41)	P	Medium dose male (n=41)	Medium dose female (n=43)	P	High dose male (n=43)	High dose female (n=41)	P	Control male (n=43)	Control female (n=41)	P
3-5 years	56	257.4(185.2~357.6)	219.0(166.2~288.4)	0.451	271.9(192.2~384.7)	265.1(192.3~365.3)	0.913	339.3(270.9~424.9)	436.1(357.0~532.8)	0.097	5.0(5.0~5.0)	5.0(5.0~5.0)	-
	84	936.7(704.0~1246.1)	668.4(502.7~888.7)	0.095	1015.7(719.9~1433.0)	805.8(613.2~1058.9)	0.287	813.0(641.2~1030.8)	837.3(649.3~1079.7)	0.864	5.0(5.0~5.0)	5.0(5.0~5.0)	-
	146	327.6(249.1~430.9)	214.8(159.3~289.5)	0.038	348.2(262.0~462.7)	248.6(194.2~318.1)	0.073	254.4(171.0~378.5)	338.4(261.8~437.3)	0.226	5.0(5.0~5.0)	5.0(5.0~5.0)	-
	236	221.6(161.9~303.3)	131.4(91.8~187.9)	0.029	178.7(111.2~287.1)	162.9(111.4~238.2)	0.759	140.1(85.5~229.6)	198.1(129.6~302.7)	0.282	5.0(5.0~5.0)	5.0(5.0~5.0)	-
6-12 years	56	101.1(66.4~153.9)	116.9(85.0~160.8)	0.575	191.6(142.9~256.9)	273.6(185.2~404.0)	0.133	268.0(195.0~368.3)	230.1(163.7~323.3)	0.505	5.0(5.0~5.0)	5.0(5.0~5.0)	-
	84	280.1(166.2~472.2)	377.5(272.8~522.5)	0.326	433.7(325.4~578.0)	590.1(446.2~780.3)	0.132	610.2(446.8~833.4)	531.8(394.4~717.0)	0.517	5.0(5.0~5.0)	5.0(5.0~5.0)	-
	146	88.4(47.9~163.0)	134.3(100.5~179.4)	0.213	197.6(115.3~338.9)	340.6(243.3~476.8)	0.086	277.4(196.6~391.4)	235.6(145.5~381.7)	0.573	5.0(5.0~5.0)	5.0(5.0~5.0)	-
	236	41.5(20.2~85.5)	52.2(29.5~92.1)	0.611	84.4(49.7~143.2)	156.2(103.1~236.5)	0.066	113.9(63.3~205.0)	95.8(56.8~161.6)	0.655	5.0(5.0~5.0)	5.0(5.0~5.0)	-
13-17 years	56	72.2(50.1~104.2)	70.7(39.3~127.1)	0.949	74.4(45.4~122.1)	82.1(56.1~120.2)	0.744	115.5(85.1~156.9)	108.1(64.3~181.7)	0.809	5.0(5.0~5.0)	5.0(5.0~5.0)	-
	84	172.1(104.1~284.6)	103.4(54.6~195.6)	0.240	125.4(70.3~223.5)	145.7(96.2~220.6)	0.659	256.0(181.2~361.7)	163.0(99.9~266.0)	0.120	5.0(5.0~5.0)	5.0(5.0~5.0)	-
	146	59.0(37.3~93.4)	42.6(20.1~90.1)	0.437	64.7(37.6~111.3)	61.3(37.0~101.5)	0.882	114.4(73.0~179.4)	92.1(51.0~166.2)	0.550	5.0(5.0~5.0)	5.0(5.0~5.0)	-
	236	22.0(13.2~36.6)	15.9(7.2~35.0)	0.486	30.9(17.7~53.8)	26.1(14.7~46.4)	0.677	54.1(34.1~85.7)	31.3(16.4~59.6)	0.154	5.0(5.0~5.0)	5.0(5.0~5.0)	-
3-17 years	56	126.8(100.7~159.7)	141.0(113.6~175.1)	0.517	172.4(136.8~217.1)	181.5(144.3~228.2)	0.753	219.7(183.4~263.2)	252.4(203.6~312.8)	0.324	5.0(5.0~5.0)	5.0(5.0~5.0)	-
	84	368.2(278.0~487.7)	377.9(293.7~486.3)	0.891	441.5(335.9~580.2)	422.2(332.3~536.3)	0.807	500.8(412.6~607.8)	484.9(386.5~608.3)	0.829	5.0(5.0~5.0)	5.0(5.0~5.0)	-
	146	124.9(94.2~165.6)	132.2(102.9~169.8)	0.766	185.4(139.9~245.8)	169.5(132.0~217.8)	0.637	196.8(154.7~250.4)	219.1(170.1~282.2)	0.544	5.0(5.0~5.0)	5.0(5.0~5.0)	-
	236	61.8(44.0~86.6)	62.8(45.1~87.5)	0.945	88.6(64.4~122.1)	87.5(64.2~119.4)	0.955	94.2(70.1~126.6)	99.8(72.4~137.7)	0.792	5.0(5.0~5.0)	5.0(5.0~5.0)	-
